# Deployment of personnel to military operations: impact on mental health and social functioning

**DOI:** 10.4073/csr.2018.6

**Published:** 2018-06-01

**Authors:** Martin Bøg, Trine Filges, Anne Marie Klint Jørgensen

## Abstract

This Campbell systematic review examines the effects of deployment on mental health. The review summarizes evidence from 185 studies. All studies used observational data to quantify the effect of deployment.

This review includes studies that evaluate the effects of deployment on mental health. A total of 185 studies were identified. However, only 40 of these were assessed to be of sufficient methodological quality to be included in the final analysis. The studies spanned the period from 1993 to 2017 and were mostly carried out in the USA, UK and Australia. The studies all had some important methodological weaknesses. None of the included studies used experimental designs (random assignment).

Deployment to military operations negatively affects the mental health functioning of deployed military personnel. For assessments taken more than 24 months since exposure, we consistently found adverse effects of deployment on all mental health domains (PTSD, depression, substance abuse/dependence, and common mental disorders), particularly on PTSD. For assessments taken less than 24 months (or a variable number of months since exposure) the evidence was less consistent and in many instances inconclusive.

**Plain language summary:**

**Deployment to military operations negatively affects the mental health functioning of deployed military personnel**

While additional research is needed, the current evidence strongly supports the notion that deployment negatively affects mental health functioning of deployed military personnel.

**What is this review about?**

When military personnel are deployed to military operations abroad they face an increased risk of physical harm, and an increased risk of adverse shocks to their mental health.

The primary condition under consideration is deployment to an international military operation. Deployment to a military operation is not a uniform condition; rather, it covers a range of scenarios. Military deployment is defined as performing military service in an operation at a location outside the home country for a limited time period, pursuant to orders.

The review included studies that reported outcomes for individuals who had been deployed. This review looked at the effect of deployment on mental health outcomes. The mental health outcomes are: post‐traumatic stress disorder (PTSD), major depressive disorder (MDD), common mental disorders (depression, anxiety and somatisation disorders) and substance‐related disorders.

By identifying the major effects of deployment on mental health and quantifying these effects, the review can inform policy development on deployment and military activity as well as post‐deployment support for veterans. In this way the review enables decision‐makers to prioritise key areas.

**What is the aim of this review?**

This Campbell systematic review examines the effects of deployment on mental health. The review summarizes evidence from 185 studies. All studies used observational data to quantify the effect of deployment.

**What are the main findings of this review?**

**What studies are included?**

This review includes studies that evaluate the effects of deployment on mental health. A total of 185 studies were identified. However, only 40 of these were assessed to be of sufficient methodological quality to be included in the final analysis. The studies spanned the period from 1993 to 2017 and were mostly carried out in the USA, UK and Australia. The studies all had some important methodological weaknesses. None of the included studies used experimental designs (random assignment).

**Does deployment have an effect on mental health?**

Deployment to military operations negatively affects the mental health functioning of deployed military personnel. For assessments taken more than 24 months since exposure, we consistently found adverse effects of deployment on all mental health domains (PTSD, depression, substance abuse/dependence, and common mental disorders), particularly on PTSD. For assessments taken less than 24 months (or a variable number of months since exposure) the evidence was less consistent and in many instances inconclusive.

**What do the findings of this review mean?**

The odds of screening positive for PTSD and depression were consistently high in the longer term. This suggests that efforts should be increased to detect and treat mental disorders, as effects may be long‐lasting.

Overall the risk of bias in the majority of included studies was high. While it is difficult to imagine a randomised study design to understand how deployment affects mental health, other matters such as changes to personnel policy, or unanticipated shocks to the demand for military personnel, could potentially be a rich source of quasi‐experimental variation.

**How up‐to‐date is this review?**

The review authors searched for studies up to 2017. This Campbell systematic review was published in March 2018.

**Executive summary:**

**BACKGROUND**

When military personnel are deployed to military operations abroad they face an increased risk of physical harm, and an increased risk of adverse shocks to their mental health. Research suggests that the increased risk to mental health is mainly due to the hazards of war, combat exposure: firing weapons, road side bombs, seeing fellow soldiers, friends, civilians, and enemies being injured, maimed or killed. These experiences may lead to severe mental stress. The adverse impact on mental health is the psychological cost of war, and it is of interest to policymakers to learn the magnitude of these effects. This review sets out to synthesise available evidence about the consequences of deployment for deployed military personnel in the mental health and social functioning domains.

**OBJECTIVES**

The objective of this review isto synthesise the consequences of deployment to military operation on the mental health and social functioning of deployed military personnel.

**SEARCH METHODS**

We searched electronic databases, grey literature, and references from primary studies and related reviews. No language or date restrictions were applied to the searches. We searched the following electronic databases: Academic Search Elite, Cochrane Library, EMBASE, ERIC, MEDLINE, PsycINFO, Science Citation Index, Social Science Citation Index, SocINDEX, as well as the Nordic platforms: bibliotek.dk, BIBSYS, and LIBRIS. The conclusions of this review are based on the most recent searches performed. The last search was performed in April 2017.

**SELECTION CRITERIA**

Primary studies had to meet the following inclusion criteria:

- Participants: The participants should be military personnel.
- Intervention: The condition should be deployment to a military operation.
- Comparison: The relevant comparisons were either comparing a) deployed military personnel to non‐deployed military personnel, b) deployed military personnel to military personnel deployed elsewhere, for example personnel deployed to non‐combat operations, c) military personnel deployed to the same operation but stratified by combat exposure.
- Outcomes: The study should report on one or more mental health outcomes, and/or social functioning for the deployed participants. In particular studies should report on one or more of the following mental health outcomes: PTSD, major depression, substance abuse or dependence (including alcohol), and common mental disorders (depression and anxiety disorders). The following social functioning outcomes were relevant: employment, and homelessness.
- Study Designs: Both experimental and quasi‐experimental designs with a comparison group were eligible for inclusion in the review.

Studies were excluded if they:

- Reported on deployments taking place before 1989.
- Used a within group pre‐post study design.
- Did not report on at least one of the mental health or social functioning outcomes.

**DATA COLLECTION AND ANALYSIS**

The total number of potentially relevant studies constituted31,049records. A total of 185 studies met the inclusion criteria and were critically appraised by the review authors. The final selection of 185 studies was from 13 different countries.

Forty eight of the 185 studies did not report effect estimates or provide data that would allow the calculation of an effect size and standard error. Fifty four studies were excluded because of overlapping samples. The majority of those studies were from USA but the main reason for not using studies from USA in the synthesis was lack of information to calculate an effect size. Nearly half the studies from the UK could not be used in the synthesis due to overlap of data samples. Forty three studies were judged to have a very high risk of bias (5 on the scale) and, in accordance with the protocol, we excluded these from the data synthesis on the basis that they would be more likely to mislead than inform., Thus a total of 40 studies, from five different countries, were included in the data synthesis.

Random effects models were used to pool data across the studies. We used the odds ratio. Pooled estimates were weighted with inverse variance methods, and 95% confidence intervals were calculated. The meta‐analyses were carried out by time since exposure (short, medium, long, and other time since exposure) and by type of comparison (deployed versus non‐deployed, all deployed but stratified by either combat operations versus non‐combat operations, or stratified by combat exposure). We performed single factor subgroup analysis. The assessment of any difference between subgroups was based on 95% confidence intervals. Funnel plots were used to assess the possibility of publication bias. Sensitivity analysis was used to evaluate whether the pooled effect sizes were robust across components of methodological quality.

**MAIN RESULTS**

The findings were mixed, depending on the outcome, the time since exposure and the approach (deployed versus non‐deployed termed absolute or stratified by extent of combat termed relative) used to investigate the effect. It was not possible to analyse the outcomes homelessness and employment. All studies that could be used in the data synthesis reported on the impact of deployment on mental health; PTSD, depression, substance use or common mental disorder.

For assessments taken less than 24 months since exposure the evidence was inconclusive either because too few studies reported results in the short and medium term and/or the degree of heterogeneity between studies was large.

For assessments taken at other time points (a variable number of months since exposure) the evidence was inconclusive for the relative comparisons due to either too few studies or a substantial degree of heterogeneity between studies. For the absolute comparison the analysis of common mental disorder was inconclusive, whereas the average effects of PTSD and depression were positive and statistically significant (PTSD odds ratio (OR) was 1.91 (95% confidence interval (CI): 1.28 to 2.85) and OR=1.98 (95% CI: 1.05 to 3.70) for depression). The analysis concerning substance use indicated that deployed participants did not have higher odds of screening positive for substance use compared to non‐deployed participants (OR=1.15 (95% CI: 0.98 to 1.36)).

For assessments taken more than 24 months post exposure, meta‐analyses indicated that the odds of screening positive for PTSD, depression, substance use and common mental disorder were higher for participants in the deployed group compared to participants in the group that were not deployed (PTSD OR=3.31 (95% CI: 2.69 to 4.07), OR=2.19 (95% CI: 1.58 to 3.03) for depression, OR=1.27 (95% CI: 1.15 to 1.39) for substance use, and OR=1.64 (95% CI: 1.38 to 1.96) for common mental disorder). Likewise, participants reporting high combat exposure had higher odds of screening positive for PTSD and depression than participants reporting lower exposure for long term assessments (PTSD OR=3.05 (95% CI: 1.94 to 4.80) and OR=1.81 (95% CI: 1.28 to 2.56) for depression). The analyses of substance use and common mental disorder were inconclusive due to too few studies.

On the basis of the prevalence of mental health problems in pre‐deployed or non‐deployed population based comparison sampleswe would therefore expect the long term prevalence of PTSD in post‐deployed samples to be in the range 6.1 – 14.9%, the long term prevalence of depression to be in the range from 7.6% to 18%, the long term prevalence of substance use to be in the range from 2.4% to 17.5% and the prevalence of common mental disorder to be in the range from 10% to 23%.

Sensitivity analyses resulted in no appreciable change in effect size, suggesting that the results are robust.

It was only possible to assess the impact of two types of personnel characteristics (branch of service and duty/enlistment status) on the mental health outcomes. We found no evidence to suggest that the effect of deployment on any outcomes differ between these two types of personnel characteristics.

**AUTHORS’ CONCLUSIONS**

Deployment to military operations negatively affects the mental health functioning of deployed military personnel. We focused on the effect of deployment on PTSD (post‐traumatic stress disorder), depression, substance abuse/dependence, and common mental disorders (depression and anxiety disorders). For assessments taken less than 24 months (or a variable number of months since exposure) the evidence was less consistent and in many instances inconclusive. For assessments taken more than 24 months since exposure, we consistently found adverse effects of deployment on all domains, particularly on PTSD. There is increased political awareness of the need to address post deployment mental health problems. The odds of screening positive for PTSD and depression were consistently high in the longer term. This suggests that efforts should be increased to detect and treat mental disorders, as effects may be long lasting. Mental illness is of particular concern in the military for operational reasons, but they may be hard to detect in the military setting because a military career is intimately linked with mental and physical strength.

It was not possible to examine a number of factors which we had reason to expect would impact on the magnitude of the effect. This would have been particularly relevant from a policy perspective because these are direct parameters that one could use to optimally “organize” deployment in order to minimize impacts on mental health functioning.

While additional research is needed, the current evidence strongly supports the notion that deployment negatively affects mental health functioning of deployed military personnel. The next step is to begin to examine preventive measures and policies for organizing deployment, in order to minimize the effects on mental health.

## 1 Background

### 1.1 THE PROBLEM, CONDITION OR ISSUE

A larger proportion of military personnel deployed to military operations now return from their deployments compared to earlier military cohorts (Sollinger, Fisher, & Metchser, 2008). This is in part due to the nature of the conflicts in the post‐Cold War era, but also innovations in modern warfare. These innovations concern both a shift towards advanced technological warfare with less reliance, historically speaking, on ground troops to carry out and complete missions, but also advancement in the protection of military personnel from the physical hazards of war and treatment of injuries. At the same time, the demands on military personnel have increased due to a secular trend towards smaller standing armies. As a result, military personnel find themselves facing longer and more frequent deployments, with shorter rest periods between deployments (Bruner, 2006).

Set against this change in the scope and nature of military deployments are some of the consequences of deployment. Returning personnel will, depending on mission type and/or assignment, have been exposed to a wide variety of stress factors, such as witnessing violent death, physical abuse, dead and/or decomposing bodies, maimed soldiers and civilians, been captured as prisoners of war, or witnessed movements of refugees as a result of civil war. Frequently, military personnel face deployment to theatres of insurgence or counter‐insurgency, where insurgents operate using guerrilla tactics such as suicide missions and road‐side bombings (for example, improvised explosive devices). Deployments to such theatres carry their own risk scenarios, including traumatic brain injury (TBI) and loss of limb (Tanielian & Jaycox, 2008). The lack of a real frontline of war implies that even support troops face increased risk of combat exposure (Street, Vogt, & Dutra, 2009). In addition, such experiences frequently take place within a context that is very different from everyday life: much of the social support network of the deployed is missing, as family and friends are far away, use of civilian communication systems may be difficult, there is often a lack of personal space, and living quarters may be unhygienic (MHAT‐IV, 2006).

The majority of deployed military personnel who return home will return and readjust successfully to either civilian life or life on a military base (Tanielian & Jaycox, 2008). However, combat exposure and other stressors increase the risk of physical and psychological trauma and, as a result, a substantial proportion of those returning from deployment to military operations abroad face the real risk of adverse effects to their mental health and social functioning (Hoge, Auchterlonie, & Milliken, 2006). These include increased risk of post‐traumatic stress disorder(PTSD), depression, anxiety, anger, and substance abuse (Andersen, 1998; Dohrenwend, Turner, Turse, Adams, Koenen, & Marshall, 2006; Helzer, Robins, and McEvoy, 1987; Hoge et al., 2004; Ishøy et al., 1999; Jacobson et al. 2008; Jakupcak et al., 2007; Larson, Highfill‐McRoy, & Booth‐Kewley2008; O'Brien & Hughes, 1991; O'Toole, Schureck, Marshall, Grayson, & Dobson, 1999; Pitman, Altman, and Macklin, 1989; Rona et al., 2009; Tanielian & Jaycox, 2008). Estimates presented in Tanielian and Jaycox (2008) on the prevalence of PTSD among US troops serving in Afghanistan and Iraq reports numbers ranging from 4‐45% of those deployed^1^. In contrast, prevalence in samples of pre‐deployed and non‐deployed personnel fall within a more narrow range. Sundin, Fear, Iversen, Rona, and Wessely (2010) review PTSD prevalence among personnel deployed to Iraq. They report PTSD prevalence in pre‐deployed and non‐deployed personnel in the range of 2% to 5%. The prevalence among personnel deployed to Iraq ranged from 1.4% to 31%. Prevalence measures tended to be larger in anonymous surveys, and in samples of US personnel as opposed to UK personnel. They also indicate that the prevalence of PTSD increases within 12 months of returning from deployment.

The severity and duration of PTSD symptoms can vary greatly between individuals. PTSD symptoms generally begin shortly after the trauma, however the onset may be delayed for months or years (Bisson, 2007; Friedman; 2006). Atkinson, Guetz, and Wein (2009) estimate that, due to the lag in diagnosing PTSD and the fact that, as a result, some service members with undetected PTSD are deployed to combat zones again, the cross‐sectional estimates may underestimate the true incidence of PTSD by as much as 100%. Their results suggest that as many as 300,000 US Army and Marine service members may be affected by PTSD.

Adverse mental health outcomes have a direct effect on individual wellbeing, but, in addition, have a number of indirect effects. For example, the families of deployed personnel who return with mental health problems may face strain in family relations, and mental health problems may be transferred between generations, particularly if the affliction is untreated (Davidson & Mellor, 2001; Dekel & Goldblatt, 2008; O'Toole, Outram, Catts, & Pierse, 2010). Furthermore, additional resources at the societal level must be allocated to screen, diagnose and treat the consequences of deployment.

These complicated issues raise important questions about the effects of deployment on those service members who return from military deployment. This review focuses on the effects of deployment on mental health and social functioning. One of the objectives of this review is that it should help inform current decision making and provide information regarding the type of deployments and operations and their present day consequences. We therefore believe it is timely and important to consider and assess the consequences of deployment on the mental health and social function on service memberssince the fall of the Iron Curtain in early 1989.

#### 1.1.1 Military operations in the post‐Cold War era

The landscape of military deployment has changed dramatically since 1989. While military deployment by its very nature carries substantial risk of exposure to events and experiences that may physically and mentally impair those directly engaged in combat and those engaged in support functions, there are a number of reasons why the extent of this issue is arguably of even greater importance now.

First, the types of security threats and military operations have changed substantially since 1989. The fall of the Iron Curtain released the tight grip of the former USSR on the Eastern Bloc, and in many developing nations across the globe where the Cold War had been fought by proxy. As a consequence, a dramatic increase in peacekeeping and peace‐enforcing operations ensued, many under the auspices of the United Nations. In the period from 1989 to 1994, the UN Security Council authorized a total of 20 new operations, raising the number of peacekeepers from 11,000 to 75,000 (United Nations [UN], 2014a). In addition, the post‐Cold War period is characterised by a shift away from more conventional conflicts between nations towards, for example, the War on Terror, support for insurgencies or counterinsurgencies, and operations to protect civilians in countries under threat of civil war. During this time ad hoc international military coalitions have been formed, including during the Gulf War (1990‐91) and in the recent operations in Iraq and Afghanistan.

Second, nations such as the US and UK have gone from having a large proportion of their military personnel in the Active Component, towards a strategy with increased reliance on the Reserve Component to meet operational needs (Department of Defense, 2008). In the US, reserves were deployed in large numbers during the Gulf War for the first time since the Korean War. This marked a shift in military personnel strategy where the reserve component was previously seen as a strategic reserve that could be activated in case of large scale war, and towards a conception of the reserves as an integral part of military operations (Klerman, 2008). Since the end of the Cold War the US Army consists of around 50% reserves (Feickert, & Kapp, 2014). In the UK a similar trend can be observed. While the total number of personnel in the UK Forces is shrinking, the volunteer reserve is on the increase, in line with the Future Reserves 2020 programme which aims to increase the size of the Reserve Force in the UK (Ministry of Defence, 2015). The shift in strategy towards viewing the reserve component as an operational force has affected the types of people who are deployed; reservists differ from personnel in the Active Component in terms of training received, physical and mental screening, frequency of civil careers, and potentiallyin unobserved ways as well. For example, as a consequence of the large scale commitment of the US, and in turn that of its allies, to military operations in Afghanistan and Iraq, an increasing share of those deployed consists of reservists and national guards. In January 2014, the UN had 15 peacekeeping missions worldwide^2^ and a special political mission in Afghanistan^3^ (UN, 2014b), and NATO hadfive operations internationally with multilateral participation^4^ (NATO, 2014). Furthermore, there are international coalitions with active military operations in other countries, for example Iraq.^5^ In the period 2001 to 2008, approximately 1.64 million US troops were deployed to Afghanistan or Iraq (Tanielian & Jaycox, 2008). As recently as 2011, Canada had over 3,500 military personnel deployed overseas (National Defence and Canadian Forces, 2011), Australia had approximately 2,900 soldiers deployed to international missions (Australian Department of Defence, 2011), and Denmark had approximately 1,500 military personnel deployed to international missions and has had approximately 50,000 military personnel deployed to international military missions since 1992 (Danish Armed Forces, 2011). As a consequence of the growing demand for international security, larger proportions of military personnel are being deployed, multiple deployments are becoming more frequent, and breaks between deployments are being shortened in order to meet demand (Hosek, Kavanagh, & Miller, 2006).

Third, technological advancements in body armour and other military technology, medical technologies, and military tactics, have increased chances of survival among those who face military deployment (Regan, 2004; Warden, 2006).

#### 1.1.2 The evidence

The nature of the issue makes it difficult to establish a causal link between deployment and mental health outcomes. For example randomised controlled trials where military personnel are explicitly randomly assigned to either face deployment to a combat zone or to not be deployed are not available. Instead we have found a large base of observational studies. Within the broad class of observational studies, a distinction can be drawn between (a) correlational studies which simply seek to measure incidence in the military population regarding the outcomes of interest (for example, PTSD), and its correlates (“risk” and “resilience” factors), and (b) studies that attempt to create a credible comparison group to the sample of deployed.

In the latter case, and provided that the comparison group is credible, we are likely to have the best available evidence, to shed light on the impact of deployment on the mental health and social functioning of deployed service members. A credible comparison group might consist of a sample from the military population that did not deploy at the time. If this group is balanced on important confounders, it may be credible to interpret the difference in outcomes between those deployed and those not deployed as the impact of deployment. In situations where military personnel face the possibility of deployment to several different operations, and where this assignment is conditionally independent, it may be reasonable to argue that combat exposure, and hence the risk of trauma, is conditionally independent. In this case a credible measure of the relative impact of deployment can be estimated. Even so, conclusions about causal effects must be very tentative.

### 1.2 THE CONDITION

The primary condition under consideration is deployment to an international military operation. Deployment to a military operation is not a uniform condition; rather, it covers a range of scenarios. Military deployment is defined as performing military service in an operation at a location outside the home country for a limited time period, pursuant to orders.

Staff at military bases across the globe experience varying degrees of extreme and challenging conditions. Military deployment differs from most civilian tasks due to the risk to loss of life, and more generally to the palette of risk factors that military personnel are exposed to while deployed. This review focuses on the effects of deployment on the mental health and social functioning of personnel returning from deployment. The multi‐faceted nature of deployment incorporates a wide variety of conditions and circumstances due to variation in the conflict and setting, type of operation, tasks, combat intensity, characteristics of those deployed, and duration of deployment itself.

#### 1.2.1 Type of mission

International military missions may be peacekeeping or peace‐enforcing missions under the UN, NATO, multilateral military coalitions or other international agencies. Such operations may involve the deployment of air, navy and army personnel as well as support staff who are not directly involved in military confrontation.

International military operations as studied in this review fall in the categories of low intensity conflicts (LIC) or conventional warfare. Conventional warfare is defined as warfare, other than guerrilla/counterinsurgent warfare, conducted without the use of nuclear, biological, or chemical weapons (Oxford University Press, 2001). Low intensity conflict is defined by the US Department of Defense: *“Low intensity conflict is a political‐military confrontation between contending states or groups below conventional war and above the routine, peaceful competition among states”* (Department of Defense, 2011). LIC can be classified in four major operational categories (Department of Defense, 2011; Global Security, 2011):

- support for insurgencies and counterinsurgencies
- combating terrorism
- peacekeeping operations
- peacetime contingency operations

LIC frequently involves protracted struggles between competing principles and ideologies. LIC ranges from subversion to the use of armed force. It is waged by a combination of means, employing political, economic, informational, and military instruments. Low intensity conflicts are often localized, generally in developing countries, but contain regional and global security implications (Global Security, 2011).

Within low intensity conflict and conventional war there can be great variation in levels of combat intensity. Peacekeeping missions may be non‐combat or low combat intensity deployments and relatively free from conflict exposure and be relatively stress free. An example of a non‐combat mission is the UN peacekeeping mission in Cyprus. At the other end of the range we find high combat intensity missions as seen in missions by NATO and coalition forces deployed to the Middle East. Military operations in Afghanistan and Iraq are examples of deployments that involve high and frequent combat exposure.

Military personnel may also be deployed to areas struck by natural disaster or military bases overseas not included in a military mission or operation, for example, US military bases in Europe. This type of deployment is outside the scope of the review.

#### 1.2.2 Type of service

Deployed personnel may come from different military populations, depending on country of origin and scale of operations. Some countries operate volunteer military such as the UK and the US, while other countries rely fully or partially on mandatory conscription to the military. As a consequence, the underlying characteristics of military personnel, including mental health characteristics, are not uniform. All deployed service members, regardless of the deploying nation, are included in the review.

Deployed personnel may derive from different military population pools including personnel from Active and Reserve Components, and national guards. Military reserve forces are members of the armed forces who combine a civil career with a military career. Reserve forces have a signed contract with the military and may be called in for active duty if needed. At any given time the demand for military personnel affects which subpopulation is at risk of deployment. For instance, at times of low demand only members of the Active Component face risk of deployment, whereas under times of high demand, such as for example under Operation Enduring Freedom to Afghanistan, large contingents of military reserve forces and national guards of the US face risk of deployment (Tanielian & Jaycox, 2008). Military personnel, regardless of type of service, are relevant to this review provided they have faced deployment to international military operations.

#### 1.2.3 Type of branch

All branches of deployed military personnel are relevant for this review. That is to say, personnel from army, navy, coast guard, Marine Corps, air force, Special Forces^6^ are all relevant. Both combat and non‐combat personnel face deployment to international missions and are thus relevant to the review. Deployed non‐combat personnel include personnel with transport/logistics functions, health and medical staff, information technology and communication staff, and general technical support personnel. As an example, the NATO mission to Afghanistan included components of deployed personnel overseeing training of local national police and military units (NATO, n.d.).

Deployed personnel regardless of military rank are relevant to the review. Military rank affects the types of operation‐specific tasks assigned to deployed personnel, and therefore rank can affect the mix of stress factors to which personnel are exposed. We therefore consider military rank to be an important control.

### 1.3 HOW DEPLOYMENT MIGHT AFFECT MILITARY PERSONNEL

Deployed personnel participate in military operations in order to fulfil a military objective. Military deployment itself involves increased risk of exposure to a number of stress and risk factors which are linked to mental health outcomes. Mental health problems may be long lasting, can influence the daily life of individuals profoundly, and can affect reintegration into civil society.

**Figure 1:**
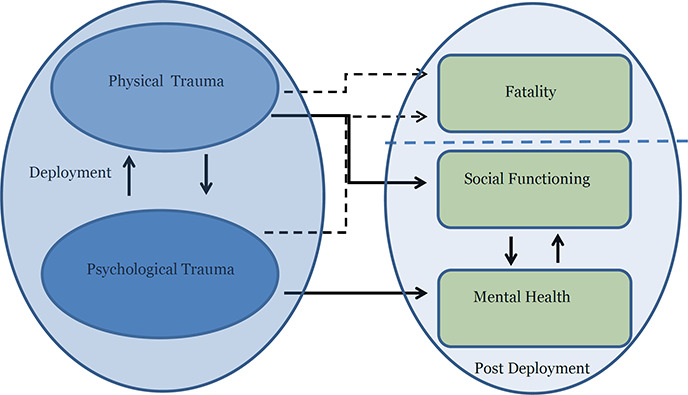
Military deployment and post‐deployment outcomes

To highlight these issues, Figure 1 presents a schematic overview linking events during deployments to outcomes. The events or series of events of interest to this review are events that may occur during deployment to war zones: the experience of physical and/or psychological trauma. *The Diagnostic and Statistical Manual of Mental Disorders ‐IV*(4^th^ edition); American Psychiatric Association, 1994) defines (psychological) *trauma* according to two criteria: (i) a person “experienced, witnessed or was confronted with an event or events that involved actual or threatened death or serious injury, or a threat to the physical integrity of self or others,” and (ii) “the person's response involved intense fear, helplessness, or horror” (p. 427‐28). It is note worthy that the experience of the event alone is not sufficient. In order to induce trauma the individual must have an emotional response of fear, horror, or helplessness^7^. The experience of trauma does not necessarily lead to any mental disorders, but, if it does, it can lead to a multitude of disorders such as: depression, specific phobias, panic disorder, personality disorders, and PTSD (Hyams, Wignall, & Roswell, 1996). The individual experience of physical trauma, such as loss of limbs, may also produce psychological trauma, but not necessarily.

Deployment entails an increased risk of exposure to such events compared to civilian life. Physical trauma may be experienced by the individual who is, for example, wounded in combat or injured by suicide bombers, improvised explosive devices (IEDs), and so on. The individual may also witness physical harm to others, such as the killing of civilians, mass graves, explosions, and watching fellow combatants sustain injury or death during combat. The witnessing of such events may cause psychological trauma to the individual.

The experience of physical and/or psychological trauma may have a number of consequences. First, the experience of personal trauma may directly lead to fatality. Such a dire consequence of trauma is outside the scope of this review. Second, the experience of trauma may affect the mental health and social functioning of those returning from service. Adverse mental health outcomes may in turn indirectly affect measures of social functioning that may include employment, homelessness, ability to sustain marital relations. The experience of physical trauma can have a direct effect on social functioning. For example, the loss of a limb may make it impossible to return to specific civil careers, which may in turn indirectly affect the mental well‐being of the veteran.

#### 1.3.1 Impact on mental health

The effects of deployment on mental health has received relatively little attention prior to World War I (WW1), although attempts at diagnosing war syndromes go at least back as far as the U.S. Civil War (Hyams et al., 1996). During WW1, deployed personnel suffered from what became known as “soldier's heart.” Affected soldiers reported, amongst other symptoms, fatigue, headache, confusion, and lack of concentration. An acute combat stress reaction (CSR) was also diagnosed (“shell shock”). In response, a clinical research program was developed during the war. After the Vietnam War, returning veterans were diagnosed with post‐traumatic stress disorder (PTSD), which was subsequently detected among veterans of the Korean Conflict and WW2 (Weisæth, 2002; Jones et al., 2002). Unlike acute combat stress reaction, which is an immediate result of psychological trauma, PTSD refers to the long term consequences of psychological stress (Hyams et al., 1996). PTSD is characterised by a range of symptoms: “[…] distressing thoughts, feelings, and images that recapitulate the traumatic event, avoidance of stimuli associated with the event, emotional numbing of responsiveness, […]” (Fairbank, Ebert, & Cadell, 2001, p. 184). PTSD is diagnosed only after symptoms have persisted for at least 30 days (Tanelian & Jaycox, 2008). Another comorbidity of deployment is major depressive disorder (MDD). MDD is a mood disorder that affects the everyday functioning of an individual. Amongst the common symptoms is disinterest in activities, significant weight gain/loss, insomnia/hypersomnia, feelings of worthlessness, thoughts of suicide, and/or suicide plans.

Deployment mainly affects the mental health of those deployed through increased risk of exposure to trauma (Cherry et al. 2001; Dohrenwendt et al., 2006; Larson et al., 2008). Exposure to trauma, either by participating in armed combat or as a witness to combat or the consequences of combat, for example, military medical teams and logistic officers, may adversely affect mental health. Deployed military personnel, particularly combat soldiers, experience risk of fatality, and some will see their peers wounded, maimed or killed.

Specific circumstances may represent specific stress factors. An example is the UN peacekeeping mission in former Yugoslavia. The mission operated under a limited UN mandate, which allowed only neutral surveillance and the prohibition of the use of force. While stationed in Yugoslavia, deployed soldiers witnessed extremely stressful situations when they observed direct attacks upon the civilian population. Another potentially contributing factor is the fact that deployment by its very nature implies the (temporary) removal of individuals from their everyday environment, including separation from family, friends, colleagues and support networks. Relations with friends and family during, and particularly after deployment, appear to be correlated with psychological stress reactions and affect social functioning (Christensen, 2001; King, King, Fairbank, Keane, & Adams, 1998). Similarly, Fontana and Rosenheck (1994) found that the homecoming experience of veterans played in important role in the development of PTSD.

The epidemiological literature on PTSD has revealed a number of risk factors for the development of PTSD following trauma in the general population as well as in military populations. Some of the relevant risk factors for this review are: being female, having a pre‐existing psychological disorder, having a family history of anxiety and depression, past exposure to trauma, degree and nature of war zone exposure (Fairbank et al., 2001). All else being equal, longer individual deployments can result in a higher risk of occurrence of at least one traumatic incident. Deployment length is therefore an important variable of interest. Deployment length varies by country and by type of service (Buckman et al., 2011). For example the UK Army typically deploys for 6 months, while the US Army typically deploys for a period of between 12 to 15 months. The UK Navy may face deployments of up to 22 months for every 36 month period (unless deployed on land), while the US Navy and Marines deploy for 7 months. The UK Air Force deploys for 9.3 months in every 24‐month period, while in the US deployments are of 4 month duration. Within these expected deployment lengths duration may vary considerably from sometimes only a few days to well above a year (US Army, 2011). Both troop demands and the nature of operations affect the length of deployment. Generally, however, deployment length varies between six and 12 months. Street et al. (2009) argue that mental “resilience” may decrease over time, therefore the period of time between multiple deployments and the number of deployments may also be important factors for predicting the experience of trauma. This is particularly relevant since, as a result of the large scale troop demands for recent operations in Iraq and Afghanistan, military personnel in general are deployed longer than before and have shorter rest‐periods between deployments (Tanielian & Jaycox, 2008).

Deployment may also lead to substance abuse or dependence, which is considered a mental disorder (American Psychiatric Association, 1994)^8^. Long term effects of deployment may be increased alcohol intake, especially linked to combat exposure, PTSD, and depression (Jacobson et al., 2008). Deployed military personnel are more likely to experience new onset heavy weekly drinking, binge drinking, and alcohol related problems, compared with non‐deployed military personnel (Hoge et al., 2004; Jacobson et al., 2008). This is particularly evident for deployed individuals with combat exposure (Fear et al., 2010; Jacobson et al., 2008). Deployed individuals diagnosed with PTSD and depression have increased the odds of new onset and continued alcohol‐related problems (Fear et al., 2010; Jacobson et al., 2008). A few studies suggest that drug use is an increasing concern, particularly for individuals deployed to Afghanistan and Iraq, where heroin is readily available (Karney, Ramchand, Osilla, Caldarone, & Burns, 2008; Thomsen et al., 2010).

Traumatic Brain Injury (TBI), more specifically the cognitive consequences of TBI, has received attention following the military operations in Iraq and Afghanistan. In those campaigns, insurgents have increasingly made use of IEDs (Improvised Explosive Devices) in their fight against international coalitional forces. Tanielian and Jaycox (2008) found that 19.5% of a representative sample of the US military population which had served in Iraq or Afghanistan had probable TBI.

For completeness, we note that the experience of trauma can also lead to post‐traumatic growth, understood as an individual sense of strength upon having successfully coped with trauma (Ramos & Leal, 2013). Tedeschi and Calhoun (1996, 2004) identify five domains where PTG may occur: “personal strength,” “new possibilities,” “relating to others,” “appreciation of life,” and “spiritual change.” In the context of military deployment, personnel have reported positive post‐deployment outcomes such as increased self‐esteem, personal development, and strong peer bonding (Danish Ministry of Defense, 2010; Rona et al., 2005; Thoresen, 2006). This review does not consider consequences of deployment for post‐traumatic growth.

#### 1.3.2 Impact on social functioning

There is little evidence for the direct link between deployment and social functioning. Nevertheless, we are interested in exploring the effects of deployment on social functioning, which may work through multiple channels. For example, social functioning may be affected both directly and indirectly by the traumas of war. Social functioning may be affected indirectly by psychological trauma experienced during deployment. Trauma may result in the development of mental afflictions which may, in turn, affect the social functioning of veterans. More direct channels in which deployment affects social functioning recognise that deployment to a combat zone leads to increased risk of loss of limbs, paralysis, TBI, and so on. Such factors may affect the chance of, for example, gaining employment upon returning from military service abroad, maintaining marital bonds and friendships. Deployment may also entail loss of firm specific human capital leading to adverse effects on salary or employment (Harvey et al., 2011).

We understand social functioning as the ability to undertake tasks and duties in civil society. Social functioning includes the ability to carry out work and home tasks, the degree to which financial concerns are experienced, relationships with family, satisfying sexual relationships, social contacts, and pleasure in spare time activities. Here we specifically focus on a narrow subset of objective indicators of social functioning namely: employment and homelessness. It is unclear whether these indicators of reintegration into civilian life are linked directly to deployment (Karney et al., 2008). Absence from the civil labour market during deployment (and military service in general) may affect employment after deployment. Employment status of veterans has been linked to their mental health. Deployed military personnel with PTSD are less likely to be employed than deployed military personnel without PTSD (Karney et al., 2008; Zatzick et al., 1997). Homelessness is more prevalent in post‐deployed military personnel compared to the general population (Perl, 2007; Perl, 2015).Tsai and Rosenheck (2015) review three decades of research on the risk factors for homelessness among US veterans. They identify substance use disorders and mental illness as the strongest factors associated with homelessness. These are also strong risk factors for the general population. Tsai and Rosenheck (2015) find that PTSD is not a particularly important risk factor for homelessness relative to other mental illnesses, in spite of the large prevalence in veteran populations. This could be explained by institutional arrangements whereby veterans with PTSD are more likely to be in contact with VA or DoD services that protect against homelessness. In as far as military deployment entails an increased risk of substance use and mental illness for example as a result of trauma caused by combat exposure, there is an indirect association between deployment and homelessness.

### 1.4 PREVIOUS REVIEWS

Two previous reviews have studied the mental health effects of deployment. The review most closely related to the topic of interest in the current protocol is a systematic review of psychiatric disorders in veterans of the Persian Gulf War of 1991 (Stimpson, Thomas, Weightman, Dunstan, & Lewis, 2003). The aim of Stimpson et al. (2003) was to review all studies comparing the prevalence of psychiatric disorders in Gulf War veterans with a group of service members not deployed to the Gulf War. The review authors identified 20 studies in which the desired comparison was present. Although heterogeneity between studies was significant, all studies reported increased prevalence of PTSD and common mental disorder in Gulf War veterans compared with the prevalence in other active service members not deployed to the Gulf War. For a description of the methods used in the primary studies, see section 1.5. The present review goes beyond the Stimpson review in scope in that it goes beyond studying veterans of the Gulf War only. One advantage is that this review will be able to go beyond the specifics of the Gulf War to uncover more general patterns of the effects of deployment. For example, the Gulf War was characterised by a number of unique stressors such as usage of uranium depleted shells and the burning of Kuwaiti oil wells.

The review by Buckman et al. (2011) examines the relationship between mental health and deployment length. Their review is broader than this review in that they consider all deployed personnel including private security personnel and journalists. In addition, they examined the effect of a discrepancy between expected deployment length and actual length on health and well‐being. A systematic search of studies measuring deployment length and the issue of ‘mismatch’ between expected and actual deployment length was conducted. Nine studies were reviewed. The review suggests that, as deployment length increases, the potential for personnel to suffer adverse health effects also increases. Our review will be broader in scope in that it is more broadly interested in measuring the effects on mental health of deployment. Deployment length may be an important factor in this respect in that it may moderate the effects of deployment.

### 1.5 WHY IT IS IMPORTANT TO DO THE REVIEW

Deployment to military missions affects many people across the globe with the increase of international military operations. For example in 2011, the missions to Afghanistan involved approximately 132,000 deployed from 48 nations and in Iraq 49,700 deployed (USF‐Iraq, 2011). To the best of our knowledge the evidence post‐dating the Gulf War has not been systematically reviewed. We seek to synthesise the existing research and systematically organise knowledge about important consequences of deployment.

In addition, we want to explore, evidence permitting, in a comprehensive way how important consequences of deployment co‐vary with policy instruments such as deployment length. This review may contribute to identifying whether the consequences of deployment for mental health and social functioning varies over time, and whether some service branches are more affected than others. It is also important to learn whether personnel from the Reserve Components are affected more or less severely than members from the Active Component.

Furthermore, we seek to identify gaps in existing knowledge in order to enhance future options for prioritising research in the field. This review is also conducted in order to inform potential next steps in policy development in the area of deployment and post‐deployment support. By identifying the major effects of deployment on mental health and quantifying these effects, the review can inform policy development on deployment and military activity as well as post deployment support for veterans. In this way the reviewenables decision makers to prioritise key areas.

## 2 Objectives

The objective of this review was to synthesise relevant studies in the research literature on the impact of deployment of military personnel to international military operations after 1989 with respect to the impact of deployment on:

Mental health:
  ∘ Post‐traumatic Stress Disorder (PTSD)
  ∘ Major Depressive Disorder
  ∘ Substance‐related abuse or dependence
  ∘ Common Mental Health Disorders (depression, anxiety, and somatization disorders)
Social functioning:
∘ Employment
∘ Homelessness

## 3 Methods

### 3.1 TITLE REGISTRATION AND REVIEW PROTOCOL

The title for this systematic review was approved by The Campbell Collaboration on 20 August 2010. The review protocol was approved on 3 November 2014. The title registration and protocol are available at: https://www.campbellcollaboration.org/library/impact‐of‐military‐deployment‐on‐mental‐health.html

### 3.2 CRITERIA FOR CONSIDERING STUDIES FOR THIS REVIEW

#### 3.2.1 Types of study designs

We expected the majority of studies to be based on observational study designs; it is difficult to imagine that a military decision‐maker would be willing to randomise between deploying and not deploying individual military personnel to an operation. In other words, even though assignment to military service may have derived from a draft lottery (such as for example the US Vietnam Draft Lottery), assignment to the deployment condition is unlikely to berandom. The decision procedures of military commanders typically involve a trade‐off between the benefits of deploying experienced and effective personnel, often based on group cohesion, to the cost of deployment on physical and mental stress (Wessely, 2006). In other words, individual assignment to deployment, even if that individual is eligible as per contract, is not random. Naturally, randomised studies would be eligible for this review should our search strategy uncover any such studies. It was more likely that there were studies where assignment locally produces high quality quasi‐experiments. Such studies may be available if the authors of primary studies have direct access to the decision rules of the decision maker responsible for assignment. For example, eligibility for individual deployment may be contingent on reaching a particular fitness score.^9^ If this is the case, and authors have access to this score, then it is possible to construct a valid comparison group by comparing individuals just above a pre‐specified cut‐off with those just below the cut‐off. In cases where the authors of primary studies do not have access to such information, or procedures do not allow authors to identify the relevant sub‐population, identification must rely on other strategies for constructing a credible comparison group. For example, Angrist, Chen, and Frandsen (2010) use the Vietnam Draft Lottery as an instrumental variable to identify the causal effect of exposure to the draft on labour force participation in the 1990s. Each such study was judged on how well it addressed the identification problem based on the risk of bias model outlined below in Section 3.3.3.

The study designs that were eligible for this review included:

- Controlled trials: Randomised controlled trials, quasi‐randomised trials (where participants are allocated by non‐random means such as alternate allocation, birth date, day of the week, case number or alphabetical order), non‐randomised trials (where participants are allocated by other actions controlled by the researcher).
- Non‐randomised studies: the decision to deploy or not deploy individuals is not under the control of the researcher. The study must use a credible comparison group to be eligible.

Studies that contained only deployed personnel and compared outcomes in this group before and after deployment (pre‐post design) were not eligible.

#### 3.2.2 Types of participants

The populations that were eligible for this review were military personnel, from any nation, who had faced deployment to international military operations since 1989. As detailed in section 1.1 there are several reasons for why we limited the time period to after 1989. First, 1989 marks the end of the Cold War period and as a consequence the types of military engagements have changed considerably since then. Second, modern military engagement relies heavily on activating reservist components to fill workforce demands. Because members of the Active and Reservist Component tend to be different in observable (and potentially unobservable) ways, deployment can be expected to have different effects on modern armies. Third, as a consequence of technological developments in body armour more military personnel now potentially survive military confrontations, which in earlier conflicts would have resulted in death. As a result, the composition of physical and psychological trauma in military populations has changed since the end of the Cold War.

Studies of deployment to military bases (abroad) not involved in active military operations were excluded. Likewise studies that focused on the deployment of civilian personnel to peace‐keeping or war zone operations were also excluded.

All types of service members were eligible. Specifically, members of the Active and Reserve Components, and national guards were eligible.

All types of military personnel were eligible. Specifically, personnel from army, air force, navy, Marine Corps, coast guard, special forces, were all eligible for inclusion in this review.

Military personnel regardless of age, gender, ethnic background, marital status, education, military rank, and country were eligible.

#### 3.2.3 Types of deployments

The “intervention” was deployment of soldiers to an international military operation. Any type of deployment of military personnel to a military operation, both combat and non‐combat operations were eligible for inclusion. For example deployment to peacekeeping operations (such as UN peacekeeping missions in the former Yugoslavia) and deployment to theatres of war (such as deployment to the First Gulf War, Operation Iraqi Freedom, and Operation Enduring Freedom in Afghanistan) were eligible deployments.

#### 3.2.4 Types of comparisons

Military populations can be expected to differ from the general population. This is particularly relevant to this review, since it affects what constitutes an appropriate comparison group for deployed military personnel. Military personnel are on average in better mental and physical health than the civilian population. Two factors contribute to this difference. First, individuals who select themselves into a military career are not a random sample of the general population. Second, military training of recruits acts as a selection device that tends to select those who are physically and mentally strong (Haley, 1998). Individuals who are unable to cope with the stress of military training separate from the military or are discharged and return to civilian life. In addition, deployed military personnel undergo additional physical and (sometimes) mental screening prior to being selected for deployment (Warner, Appenzeller, Parker, Warner, & Hoge, 2011). As a result, it can be expected that even within the subpopulation of military personnel, deployed and non‐deployed will differ in both observed and un‐observed characteristics. This effect is well known in occupational health studies, and is known as the “healthy worker effect” (see, for example, Li & Sung, 1999). We return to this point in Section 3.3.

To address the issues raised above only studies that used a well‐defined comparison group were eligible for inclusion. Such studies, for example:

- compared deployed military personnel to non‐deployed military personnel serving the same nation in the same era;
- compared military personnel deployed to high combat intensity missions with military personnel deployed to low combat intensity or non‐combat deployments serving the same nation;
- compared deployed personnel to non‐deployed personnel; or
- compared two groups of deployed personnel deployed who experienced different levels of combat exposure in the course of their deployment.

While we can expect that military decision makers select whom to deploy and whom not to deploy, there is evidence that the deployment of individual service members to either combat versus non‐combat is unrelated to individual characteristics (for example, Cesur, Sabia, & Tekin, 2012). We argue that a distinction must be made between non‐randomised studies that simply seek to document correlation between observable characteristics of participants and outcome(s), and non‐randomised studies that attempt to mimic an experimental situation by constructing and documenting a plausible comparison group. Only the latter type of study design was eligible for this review.

We now elaborate on the characteristics of plausible comparison conditions. As stated earlier, the military population differs in important ways from the civil population, not least because service members are frequently screened mentally and physically. Therefore, a credible comparison group to a deployed group must also be sampled from the military population. Even within the military population, selection procedures, including physical and mental screening, determine who does and who does not get deployed. Therefore restricting the comparison group to be sampled from the military population is not sufficient to remove selection bias, but goes some way toward resolving this problem. In addition, a comparison group must be balanced on important confounders viz‐a‐viz the deployed group, such that at least in observable characteristics, the deployed and the comparison group differ only in the assignment to having been deployed and not having been deployed. Some important confounders include: gender, age, rank, type of service, health. We elaborate on the set of confounders in Section 3.3.3.

An example of an eligible primary study is the study by Unwin et al. (1999). They assess mental health outcomes of UK service members who deployed to the Gulf War using a postal survey. As a comparison group, they survey UK service members who were serving during the Gulf War but did not deploy there. Another eligible study is Cesur et al. (2012). They compare personnel that were deployed to combat zones during recent military operations in Afghanistan and Iraq with non‐combat deployed personnel. They argue that assignment of personnel to either combat zone or non‐combat zone is orthogonal to individual characteristics. Their estimates therefore have a (plausible) causal interpretation.

It is also possible that mandated government policies assist researchers in creating a reasonable comparison group. For example, service members who would otherwise be eligible for deployment may not have been deployed at the time of the study because they were not *yet* eligible for another deployment. If the deployed group and the comparison group are balanced with regard to number of previous deployments then this may be a valid comparison group. An example of a study that appears to have this type of information available to the researchers is Hotopf et al. (2006).

Another type of eligible study compares two (or more) deployed groups that have (on average) been exposed to different intensities of combat exposure. If deployment mainly affects mental health and other outcomes through an increased risk of trauma, then a larger degree of combat exposure increases the likelihood of trauma, all else being equal. In other words, it may be reasonable to argue that the two groups have received different *dosages* of deployment. At this point caution must be exercised since commanding officers may use selection procedures to assign individuals to more or less severe combat exposures based on characteristics that are unobserved by researchers.

In summary, this review considered two types of comparisons: (1) absolute comparisons, and (2) relative comparisons. Studies that reported an absolute comparison compared a group of military personnel that were deployed to a military operation to a group of non‐deployed military personnel. Studies that reported a relative comparison compared either a) a group of military personnel deployed to combat operations to a group of personnel deployed to non‐combat operations, or b) compared a group of deployed military personnel with within group stratification in combat exposure, for example high and low combat exposure.

#### 3.2.5 Types of outcome measures

The review included studies that reported outcomes for *individuals who had been deployed*. Studies that reported only on the consequences, for example spouse and/or children, were not included. Studies that did not measure at least one outcome among the primary or secondary outcomes listed below were not included. We took a broad scope with respect to the types of participants and military operations under consideration. In order to ensure some degree of comparability between what studies are measuring, we narrowed the set of eligible outcomes in this manner.

##### 3.2.5.1 *Primary outcomes*

Of interest to this review is the effect of deployment on mental health outcomes. The mental health outcomes that are included in this review are:

- (Probable) post‐traumatic stress disorder (PTSD)
- (Probable) major depressive disorder (MDD)
- (Probable) common mental disorders (depression, anxiety and somatisation disorders)
- (Probable) substance‐related disorder

Probable is added here because we did not expect that the majority of studies measured mental health outcomes via structured clinical interviews, which are considered the “gold” standard in this literature. Instead we expected that questionnaires were used to screen for or indicate probable mental disorders, or indicate symptom severity.

Engelhard et al. (2007) and Kehle et al. (2011) are examples of studies where current PTSD is diagnosed following structured clinical interviews. Some studies may indirectly infer clinical diagnosis from register data. Also, in this case, the outcome will only be registered for those who were exposed to the diagnostic or sought medical assistance on their own. Such measures may therefore also be susceptible to bias. The most common form of detecting symptoms of mental disorders was via self‐reported questionnaires. The use of different instruments of detection may therefore be an important source of variation for the incidence of measured mental health outcomes. Smith et al. (2008) used self‐reported symptoms measured by DSM‐IV criteria using a 17‐item PTSD checklist, PCL‐C. The PTSD Checklist (PCL) (sensitivity: 1.0, specificity: .92) was used by Hoge et al. (2004) to detect (probable) PTSD. They also used a more stringent version of the same questionnaire (sensitivity: .60, specificity: .99). Hoge et al. used the 9‐item Patient Health Questionnaire (PHQ‐9) to detect (probable) MDD. A commonly used instrument for detecting alcohol abuse was the 10‐item WHO Alcohol Use Disorder Identification Test (for example, Harvey et al., 2011).

##### 3.2.5.2 *Secondary outcomes*

Social functioning outcomes were considered as secondary outcomes. The aim of including social functioning outcomes is to provide indications of the consequences of deployment for the experience of returning to civilian life. Social functioning is a multi‐dimensional concept which includes perceived social support from military and family/friends, social participation, sexual functioning, civilian work adjustment. Some of these measures are clearly more relevant for reservists and guards. For example, reservists will often leave civil employment when activated for duty. The time away and increased risk of trauma may lead to loss of specific and general human capital, and make it difficult to return to the civil career track they were on prior to deployment. Other measures, for example, family functioning, are expected to affect all deployed, due to the separation from regular life that deployment entails.

From amongst the broader set of outcomes that can be conceptually organised within the domain of social functioning we focused on a narrow set of secondary outcomes that may be particularly important for some of the sub‐populations we included. In particular the review focused on the effect of deployment on:

- employment
- homelessness

Due to the multi‐faceted nature of the concept of social functioning we expected primary studies to use a range of outcome variables to measure aspects of social functioning. Some possible measures might include self‐reported questionnaires (either dichotomous, multi‐scale or index), authorities, files and registry data.

#### 3.2.6 Time since exposure

Another important factor is the temporal aspect of particularly the measurement of mental health outcomes. The time at which surveys were administered may be important; some mental conditions develop over time, while other conditions may have already been treated if surveys are administered later (Bliese, Wright, Adler, Thomas, & Hoge, 2007; Castro & Hoge, 2005; Hotopf et al., 2006). Hence, studies may reveal a specific country's battery of treatments and their effectiveness, rather than the incidence of mental affliction. We therefore recorded the time at which outcomes were measured relative to the end of the relevant deployment spell. All relevant measures regardless of the time of measurement were eligible for inclusion in the review. See Section 3.4.3 for the specific categorisation of time since exposure used in the synthesis.

### 3.3 SEARCH METHODS FOR IDENTIFICATION OF STUDIES

#### 3.3.1 Electronic searches

Relevant studies were identified through electronic searches of bibliographic databases, government policy databanks and internet search engines. No language or date restrictions were applied to the searches (although studies focusing on deployments prior to 1989 were not included).

The following international bibliographic databases were searched:

- Academic Search Elite (EBSCO platform) ‐ Searched until April 2017
- Cochrane Library ‐Searched until April 2017
- EMBASE (EBSCO platform) ‐ Searched until April 2017
- ERIC (EBSCO platform) ‐ Searched until April 2017
- MEDLINE (Ovid platform) ‐ Searched until April 2017
- PsycINFO (EBSCO platform) ‐ Searched until April 2017
- Science Citation Index ‐ Searched until April 2017
- Social Science Citation Index ‐ Searched until April 2017
- SocINDEX (EBSCO platform) ‐ Searched until April 2017

The following Nordic bibliographic databases were searched:

- Bibliotek.dk ‐ The Danish National Library ‐ Searched until April 2017
- BIBSYS – The Norwegian National Library ‐ Searched until April 2017
- LIBRIS ‐ The Swedish National Library‐ Searched until April 2017

#### 3.3.2 Search terms

We report exact search strings and results for all electronic databases in Online Supplement 3 (see Chapter 11).

#### 3.3.3 Searching other resources

The review authors checked reference lists in relevant reviews and included primary studies for additional references.

OpenGrey was used to search for European grey literature (http://opengrey.eu/). Australian Centre for Posttraumatic Mental Health was used to search for Australian grey literature (www.acpmh.unimelb.edu.au/). Likewise we used Rand for US grey literature (www.rand.org) and US Department of Defense (www.dod.gov) and Walter Reed Army Institute of research (http://wrair‐www.army.mil/) for relevant US military grey literature. DTIC (http://www.dtic.mil/dtic/) was searched as well. Copies of relevant documents have been made recording the exact URL and date of access.

### 3.4 DATA COLLECTION AND ANALYSIS

#### 3.4.1 Selection of studies

Under the supervision of review authors, two members of the review team independently screened titles and abstracts and excluded studies that were clearly irrelevant. Studies considered relevant by at least one of the screeners was retrieved in full text. Review team members were not blind to the authors, institutions, or journals.

Full texts were appraised by two members of the review team, and each team member independently judged whether the study met the inclusion criteria for the review. All studies that were deemed relevant by both screeners were forwarded to the review authors for a final decision regarding eligibility for the review. In the case of any disagreement between screeners the study was also forwarded to review authors.

The final inclusion decision was made by one of the review authors. However, since both numerical coding and risk of bias assessment was conducted by two review authors working separately, effectively the final inclusion decision was made by two review authors. In all instances a consensus between the two review authors could be reached, and accordingly there was no need to consult with a third review author regarding eligibility.

Reason for exclusion of studies that were assessed by review authors against inclusion criteria is documented in the reference section of the review (Section 7.2). The overall search and screening process is illustrated in a flow‐diagram presented in Section 10.3.

#### 3.4.2 Data extraction and management

The information we extracted from reports were managed in a series of Microsoft Excel sheets. Separate sheets were developed for descriptive, numerical, risk of bias, effect size extraction coding. The internal reference ID's of study reports were used to link the information in each sheet to the relevant study.

##### Extraction of descriptive data

Descriptive study level data from included studies were extracted. Characteristics of the deployment (including mission location, type, command, duration, deploying country) were extracted. Participant characteristics such as study level summary information about gender composition, average age, ethnic composition, military rank, types of exposure (including types of combat exposure) were also extracted from included study reports.

Descriptive data were extracted by two members of the review team. Each report was coded by one team member, and subsequently the coding was carefully checked for errors by the other team member.

##### Extraction of numerical data

Numerical data were extracted by two review authors. The extraction followed the coding scheme outlined in our protocol. For each report we coded the type of outcomes domains considered, the type of instrument that was used to assess the outcome including whether the outcome was a dichotomous or a continuous measure. We recorded the time of deployment and the time at which measurements were takenin order to construct the exact time of measurement (time since exposure) in relation to the relevant deployment. Sample size was coded, along with the estimation method used by study authors to estimate the relevant effect. The type of comparison was coded in particular distinguishing between effects comparing deployed personnel against non‐deployed personnel and effects where the entire analysis sample faced deployment and effects compared relative combat exposure.

When effect sizes were reported directly in the reports, or sufficient information was present to permit us to calculate an effect size, we extracted effect sizes to a separate sheet. At the effect size level information relating to type of effect size (for example OR, SMD, and so on.), type of outcome (for example PTSD), instrument (such as how study authors assessed the outcome), subgroup (such as female participants, or reserves or guards only) was coded.

One review author extracted numerical data from each study report. The coding was checked by a second review author for accuracy. Members of the review team extracted effect size level numerical data. Each entry was checked by another member of the team.

##### Risk of bias assessment

Information pertaining to risk of bias assessment (see Section 3.4.1) was extracted by two review authors. One review author performed a risk of bias assessment on each study. A second review author then assessed the report using the risk of bias coding of the first author as a starting point. The two authors then met to reach consensus on the final coding. Additional data were added to the coding by the second author, to support the judgment. Initial disagreement on how to assess risk of bias items was not uncommon. In all instances the two review authors were able to reach a consensus judgment; hence a third opinion was not sought.

#### 3.4.3 Assessment of risk of bias in included studies

We assessed the methodological quality of studies using a risk of bias model developed by Prof. Barnaby Reeves in association with the Cochrane Non‐Randomised Studies Methods Group.^10^ This model is an extension of the Cochrane Collaboration's risk of bias tool and covers risk of bias in non‐randomised studies that have a well‐defined control group.

The extended model is organised and follows the same steps as the existing risk of bias model according to the Cochrane Handbook, chapter 8 (Higgins & Green, 2008). The extension to the model is explained in the three following points:

- The extended model specifically incorporates a formalised and structured approach for the assessment of selection bias in non‐randomised studies by adding an explicit item about confounding. This is based on a list of confounders considered to be important and defined in the review protocol. The assessment of confounding is made using a worksheet where, for each confounder, it is marked whether the confounder was considered by the researchers, the precision with which it was measured, the imbalance between groups, and the care with which adjustment was carried out (see Section 9.1). This assessment informed the final risk of bias score for confounding.
- Another feature of non‐randomised studies that make them at high risk of bias is that they need not have a protocol in advance of starting the recruitment process. The item concerning selective reporting therefore also requires assessment of the extent to which analyses (and potentially, other choices) could have been manipulated to bias the findings reported, for example, choice of method of model fitting, potential confounders considered/included. In addition, the model includes two separate yes/no items asking reviewers whether they think the researchers had a pre‐specified protocol and analysis plan.
- Finally, the risk of bias assessment is refined, making it possible to discriminate between studies with varying degrees of risk. This refinement is achieved with the addition of a 5‐point scale for certain items (see the following section, risk of bias judgment items for details).

The refined assessment is pertinent when thinking of data synthesis as it operationalizes the identification of studies (especially in relation to non‐randomised studies) with a very high risk of bias. The refinement increases transparency in assessment judgments.

##### 3.4.3.1 *Risk of bias judgment items*

The risk of bias model used in this review is based on nine items (see Section 9.1). The nine items refer to: sequence generation, allocation concealment, confounders, blinding, incomplete outcome data, selective outcome reporting, other potential threats to validity, a priori protocol, and a priori analysis plan.

##### 3.4.3.2 *Confounding*

An important part of the risk of bias assessment of non‐randomised studies is how the studies deal with confounding factors. Selection bias is understood as systematic baseline differences between groups and can therefore compromise comparability between groups. Baseline differences can be observable (for example age and gender) and unobservable (to the researcher; for example underlying “mental resilience”). There is no single non‐randomised study design that always deals adequately with the selection problem. Different designs represent different approaches to dealing with selection problems under different assumptions and require different types of data. It especially varies in relation to how different designs deal with selection on unobservable variables. The “adequate” method to control for selection depends on the model generating participation, i.e., assumptions about the nature of the process by which participants are selected into a program.

For this review, we identified the following observable confounding factors to be most relevant: mental health history, gender, age, ethnicity, military rank (enlisted, officer), branch of service (for example Army, Navy, and Air Force), Duty/Enlistment status (Active, Reserve/Guard), and number of previous deployments. All confounding variables should be measured pre‐deployment.

In addition, the presence of a “healthy worker” effect (Hotopf et al., 2006; Larson et al., 2008; Li & Sung, 1999) may confound results. The military have high induction standards followed by rigorous training to test physical and mental strength and resilience. While waiting for deployment, serving troops are continually observed (and sorted). Reservists and National Guards are subject to less rigorous screening. Troops can be discharged at several decision points if physical or mental health problems are noticed (and would therefore not be deployed; Hyams, 2006). As a result, the military population will be healthier than the general population, and in addition, we expect that the deployed may differ in systematic ways from the non‐deployed, although we do not expect this difference to be as stark as the difference between the military population and the general population.

We now discuss the rationale for including each of the confounding variables.

###### Mental health history

Several studies indicate that prior PTSD and other mental disorders may help predict the onset of new disorders (Himmelfarb, Yaeger, & Mintz, 2006; Vogt, King, & King, 2007). It is unclear whether a prior history of mental health problems increases the risk of for example PTSD, or simply reveals a latent variable (“mental resilience”).

###### Gender

In general, male and female service members are likely to experience different types of combat exposure. For example, US female service members who deployed to Iraq and Afghanistan were officially barred from serving in direct combat situations. But, those wars were essentially without a frontline; due to the nature of fighting, even support personnel were at an increased risk of combat exposure. Nevertheless, the nature of exposure may be different due to different assignments. In addition, female service members, as compared to males, are likely to report a higher incidence of sexual trauma, including sexual assault and harassment while deployed (Murdoch et al., 2006; National Defense Research Institute, 2014). Tanelian and Jaycox (2008) found that deployed female service members were more likely than their male counterparts to screen for probable PTSD. Given that the military is a male‐dominated occupation, there may also be differential selection procedures by gender into a military career. Finally, there is the possibility that “true” gender differences exist in the way that males and females process trauma exposure, leading to differential outcomes in the link between exposure and, for example, PTSD, although evidence is mixed (Street et al., 2009).

###### Age

Age has been shown to be a risk factor in some studies for the development of PTSD (Brewin, Andrews, & Valentine, 2000). In addition, age may also serve as proxy for the size social support networks.

###### Ethnicity

Ethnicity may partially determine how, and if, trauma is verbalised and processed. Ethnicity may also be a proxy for different networks of social support both prior to and after deployment. Tanelian and Jaycox (2008, p. 99) find that Hispanic ethnicity almost doubles the risk ratio (RR) for PTSD (adjusted RR of 1.881) in a representative sample of US service members deployed to Iraq.

###### Branch of service

Service branch is mainly a proxy for the exposure to trauma. A very basic difference between the typical airman and the typical combat soldier is the distance to wounded combatants, smell of decaying bodies, and so on; which all are known risk factors for trauma. In addition, pre‐deployment preparation may also vary depending on branch of service.

###### Military rank

Officers are a (self‐) selected group of military personnel and can be expected to be different from enlisted personnel in the amount of training received. More importantly, officers and enlisted personnel will likely differ in the ways in which they are exposed to potentially traumatic experiences in‐theatre.

###### Duty/Enlistment status

Deployed military personnel may come from three different military populations: Active Component, Reserve Component, and National Guard. Each population differs in the frequency with which they are observed and screened (physically and mentally). For example, service members from the Active Component who live on‐base will be screened much more frequently than reservists or guards. Thus, comparisons between these sub‐populations may be susceptible to the “healthy worker” effect (Li & Sung, 1999). Hotopf et al. (2006) found different effects for personnel from the Active Component compared to personnel from the Reserve Component.

###### Previous deployments

Previous deployments may be important as some studies suggest that mental resilience can be compared to a form of mental capital that depreciates and is expended under stressful situations (Atkinson, Guetz, & Wein, 2009; Street et al., 2009). When mental capital is depleted, individuals are more likely to suffer adverse consequences to mental health. Previous deployments may serve as a proxy for the amount of mental capital expended.

#### 3.4.4 Measures of treatment effect

Effect sizes were extracted from included studies by the methods described below. Included study reports provided measurements of treatment effects on both continuous scales (typically adjusted mean differences) and as dichotomous outcome data (typically odds ratios). The direction of all effect sizes was adjusted such that a larger effect size indicated worse mental health, or poorer social functioning.

Because a large majority of studies reported measurements of deployment effects in odds ratios we converted otherwise comparable continuous effect sizes, standardised mean differences, to log odds ratios using the procedures reported by Sánchez‐Meca, Marín‐Martínez, & Chacón‐Moscoso (2003). Details are provided in Section 3.4.2.3.

##### 3.4.4.1 *Continuous data*

For continuous outcomes, we calculated effect sizes and their standard error, where means, standard deviations, and sample sizes were available. We calculated Hedges’ (adjusted) *g* and its standard error (Lipsey & Wilson, 2001, p. 47‐49):

$$g=\left(1-\frac{3}{4N-9}\right)\times \left(\frac{{\bar{X}}_{1}-{\bar{X}}_{2}}{s_{p}}\right),\;SE_{g}=\sqrt{\frac{N}{n_{1}n_{2}}+\frac{g^{2}}{2N}}$$

where $N=n_{1}+n_{2}$ is the total sample size, $\bar{X}$ denotes the (adjusted) mean of a group, and is the pooled standard deviation defined as

$$s_{p}=\sqrt{\frac{(n_{1}-1)s_{1}^{2}+(n_{2}-1)s_{2}^{2}}{\left(n_{1}-1)+(n_{2}-1\right)}}$$

Here, *S*_1_ and *S*_2_ denotes the standard deviation of the two groups.

When means and standard deviations were not available, we used methods in Lipsey and Wilson (2001) to calculate standardized mean difference effect sizes from, for example, F‐ratios, t‐values, chi‐squared values, probit estimates, and correlation coefficients.

Examples of treatment effects that were reported on a continuous scale are: PTSD (for example PCL raw scores), Depression (for example BDI raw scores), alcohol misuse/abuse/dependence (for example AUDIT raw scores), and Common Mental Disorders (for example MCS raw scores).

A number of studies that reported effect measures on the relative comparison of combat exposure only reported marginal effects. That is, these studies reported the effect of an additional unit of “combat exposure” on the outcome variable. Since it is difficult to compare marginal effects to for example effects that compare high versus low exposure, we did not synthesise these types of effect estimates.

##### 3.4.4.2 *Discrete data*

For dichotomous outcomes, we calculated effect sizes as odds ratios and the standard error of the effect size, where this was possible. The log odds ratio (LOR) and its approximate standard deviation were calculated as (Lipsey & Wilson, 2001):

$$LOR=\log\left(\frac{ad}{bc}\right),\;SE_{LOR}=\sqrt{\frac{1}{a}+\frac{1}{b}+\frac{1}{c}+\frac{1}{d}}$$

where *a* is the frequency of “cases” in the treatment group, *b* is the frequency of “non‐cases” outcomes in the treatment group, and *c* and *d* are the number of cases and non‐cases in the control group, respectively.

Some studies only reported risk rates or marginal effects as an odds ratio (for example from a logistic regression of the effect of combat exposure, a continuous measure, on PTSD caseness, a dichotomous measure). Because these effect sizes are not comparable to odds ratios we did not synthesis results based on these measures.

##### 3.4.4.3 *Effect size conversions*

We used the Cox‐transformation to transform continuous scale effect sizes (Hedges’ *g*) to log odds ratio. The Cox‐transformation for the effect size and the associated standard error is (Sánchez‐Meca, Marín‐Martínez, & Chacón‐Moscoso, 2003):

$$LOR=l.65\times g,\;SE_{LOR}=1.65\times SE_{g}$$

#### 3.4.5 Unit of analysis issues

##### Multiple intervention and control groups

When a study reported multiple deployed groups and one controlgroup were pooled, we pooled groups if appropriate (if they included different individuals) and compared it to the control group. Multiple control groups were only pooled if appropriate (if they included different individuals).

A synthetic (average) effect size was calculated in order to avoid issues with dependence between effect sizes. This method provides an unbiased estimate of the mean effect size parameter but overestimates the standard error. Random effects models applied when synthetic effect sizes are involved actually perform better in terms of standard errors than do fixed effects models (Hedges, 2007). However, tests of heterogeneity when synthetic effect sizes are included are rejected less often than the nominal significance level.

When pooling was not appropriate, for example, when the multiple deployed groups and/or control groups included the same participants, only one deployed group was coded and compared to the control group to avoid double counting participants. The choice of which estimate to include was based on our risk of bias assessment and upon theoretical relevance. We chose the estimate that we judged to have the least risk of bias (primarily, confounding and in case of equal scoring the incomplete data item was used) and that was most likely to provide the best information about the outcome being assessed.

##### Multiple studies using the same sample of participants

In some cases, different studies used the same sample of participants and reported the same estimand, for example the relation between deployment and PTSD symptom severity. One such sample of participants is the Millennium Cohort Study (Ryan et al., 2007). Participants in the Millennium Cohort Study were the focus of analysis in several included study reports. When we encountered reports using identical groups of participants, and reporting on the same outcome construct, such as PTSD symptom severity, we reviewed all such studies, but only included one estimate of the outcome construct from each sample of participants in the meta‐analyses. The choice of which estimate to include was based on our risk of bias assessment of the studies. We chose the estimate that was judged to have the least risk of bias (primarily selection bias). Secondary parameters were the number of participants, and precision of estimates.

##### Time since exposure

When outcomes were measured at different times since deployment, each time specific outcome was analysed in a separate meta‐analysis with other comparable studies taking measures at a reasonably similar time since exposure.

We categorized time since exposure from the end of the analysed deployment spell. We used the following categorization: short‐term (0‐ <6 months since last possible exposure including measures taken during deployment), medium term (6‐ 24 months after deployment end), long term (at least 24 months after deployment end). Several studies did not fit this classification because they did not report when measurements were taken, or because recruitment (baseline measures) took place over several years and follow‐up measurements (post deployment) were taken over several years. For example this is the case with some studies based on the Millennium Cohort Study (Ryan et al., 2007), such that study members were recruited over a 3 year period and then followed up over a period of three years. Studies that did not fit the taxonomy given above were coded separately (as “Other”).

#### 3.4.6 Dealing with missing data

We recorded attrition data and response rates for all studies, and this information was used in our risk of bias assessment. We did not impute missing data when we performed moderator analysis..

#### 3.4.7 Assessment of heterogeneity

Heterogeneity among primary outcome studies was assessed with χ^2^ (Q‐statistic), and the *I* ^2^, and τ^2^ statistics (Higgins, Thompson, Deeks, & Altman, 2003). The Q‐statistic was used to represent the degree of variability in the treatment effect estimates due to heterogeneity:

$$I^{2}=(Q-df)/Q\;\;\;x\;100\%$$

where *Q* is the χ^2^ test‐statistic and *df* is its degrees of freedom (Higgins & Green, 2008). The value of I^2^ lies between 0% and 100%, with a value of 0% indicating no observed heterogeneity and larger values show increasing heterogeneity (Higgins et al., 2003). In addition we reported the between‐studies variance component (τ^2^)

#### 3.4.8 Assessment of reporting biases

We used funnel plots for information about possible publication bias (Higgins & Green, 2008). Publication bias is difficult to assess because asymmetric funnel plots are not necessarily caused by publication bias (and publication bias does not necessarily cause asymmetry in a funnel plot).

#### 3.4.9 Data synthesis

We restricted synthesis to those studies that did not receive a score of five on any item in the risk of bias assessment, as stated in the protocol for the review. Studies that were scored with a five on some risk of bias item were included in a sensitivity analysis, see Section 3.4.7.2.

We conducted separate meta‐analyses by types of outcomes (PTSD, Depression, Substance use, and CMD), types of comparison (“absolute”, “relative”), and time since exposure (“short”, “medium”, “long”, and “other”). For each analysis effect sizes were averaged as appropriate.

We carried out our meta‐analysis using odds ratios. Odds ratios were log transformed before being analysed. This was because ratio summary statistics all have the common feature that the lowest value that they can take is zero, that the value 1 corresponds to no intervention effect, and that the highest value an odds ratio can ever take is infinity. This number scale is not symmetric. The log transformation makes the scale symmetric: the log of zero is minus infinity, the log of 1 is zero, and the log of infinity is infinity.

All analyses were inverse variance weighted using random effects statistical models that incorporate both the sampling variance and between study variance components into the study level weights. Random effects weighted mean effect sizes were calculated using 95% confidence intervals. Measures of heterogeneity were assessed in each analysis.

Analysis was conducted in RevMan5. Graphical displays (forest plots) for meta‐analysis performed on ratio scales sometimes use a log scale, as the confidence intervals then appear symmetric. This is however not the case for the software Revman 5. The graphical displays using odds ratios and the mean effect size were reported as an odds ratio.

##### 3.4.9.1 *Investigation of heterogeneity*

We performed single factor subgroup analysis. The assessment of any difference between subgroups was based on 95% confidence intervals. No conclusions from subgroup analyses were drawn, and interpretation of relationships was cautious, as they were based on subdivision of studies and indirect comparisons.

##### 3.4.9.2 *Sensitivity analysis*

Sensitivity analysis was used to evaluate whether the pooled effect sizes were robust across components of methodological quality. Sensitivity analysis was carried out to explore the sensitivity of results to the inclusion of studies that had scored 5 on at least one risk of bias item. We also explored the sensitivity of results to the exclusion of studies with a score of four on the *incomplete outcome data* item, the *other risk of bias* item and the *confounding* item.

## 4 Results

### 4.1 DESCRIPTION OF STUDIES

#### 4.1.1 Results of the search

We ran the searches four times. July ‐ August 2011, August ‐ September 2013, in January 2015 and in April 2017.

We searched 13 international and Nordic bibliographic databases, searched for grey literature, and snowballed reference lists for further leads (see section 3.3 for more information).

The total number of potential relevant records was 31,049 after excluding duplicates from the database searches (databases: 30,025, grey and snowball: 1,024.)

All 31,049 records were screened based on title and abstract. The review team screened 3,401 in full text. 2206 studies were initially deemed to fit the inclusion criteria for the review. 35 of the studies did not upon closer inspection meet the inclusion criteria. The references to these studies and the justifications for exclusion are listed in section 7.2. In the end 185 study reports met the inclusion criteria and were data‐extracted by the review team.

Meta‐analysis was performed on40 study reports.

#### 4.1.2 Included studies

Table 1 presents the study characteristics of the 185 studies that met inclusion criteria. The statistics given in the table are at the study level, except where a study contains multiple groups of participants deployed to different operations. All studies use observational data to quantify the effect of deployment.

**Table 1:** Study characteristics of included studies (1,685 studies)

**Study Characteristics**					
**General Characteristics**	Mean	SD	**Participant Characteristics**	Mean	SD
Publication year	2008	6			
			Male (%)	83	21
Deploying country	K	%	*not reported in 30 studies*		
UK	20	11			
USA	137	74	Caucasian (%)	69	19
Other countries*	28	15	*not reported in 89 studies*		
*not reported in 1 study*			* *		
*Australia (7), Canada (6), Denmark (3), Germany (2), Italy (1), Kuwait (1), Netherlands (2), Norway (1),Poland (1), Saudi Arabia (1) and Sri Lanka (1)			Age (years)	32	6
			*not reported in 52 studies*		
Military Operation	k	%			
Gulf War	61	33	Married (%)	56	15
OIF (Iraq)	94	51	*not reported in 72 studies*		
OEF (Afghanistan)	69	37	* *		
Other locations*	16	9	Previously deployed (%)	30	27
*not reported in 11 studies*			*not reported in 141 studies*		
*Bougainville (2), Croatia (1), East Timor (1), Golan Heights (1), Haiti (1), Kosovo (3), Bosnia (4), Somalia (2), and Yugoslavia (1)			* *		
			Active Component (%)	54	40
Deployment type	k	%	*not reported in 67 studies*		
War zone	142	89	* *		
Civil conflict zone	17	11	Enlisted (%)	84	13
*not reported in 16 studies*			*not reported in 77 studies*		
			* *		
			Military service branches^ (%)		
			Army	62	37
			Navy	14	24
			Air Force	15	26
			Marine	9	23
* *			*not reported in 70 studies*		

Notes: *k = number of studies*. Per cents and averages taken only for studies reporting the characteristic in question.

^1^The United States Armed Forces consist of the main service branches: Army, Marine Corps, Navy, Air Force, and Coast Guard. The British Armed Forces consist of the main service branches: Royal Navy (Navy), Royal Marines (Marine), British Army (Army), and Royal Air Force (Air Force). The Australian Defence Force consists of the main service branches: Royal Australian Navy (Navy), Australian Army (Army), and Royal Australian Air Force (Air Force).

Averaged across all studies the mean study was published in 2008 with a standard deviation of six years.

94 studies (51%) assessed the effects of deployment to Iraq (“Operation Iraqi Freedom”) in the period after 9/11 2001, also known as the Global War on Terror. Operation Iraqi Freedom began on 20 March, 2003, and officially ended 18 December, 2011. The 2003 invasion of Iraq was led by U.S. Army General Tommy Franks, and codenamed “Operation Iraqi Freedom”. The UK codename was “Operation Telic”, and the Australian codename was “Operation Falconer”. 61 studies (33%) assessed the effects of deployment to the Gulf War (combat phase codenamed “Operation Desert Storm”). The combat phase of the Gulf War took place between 17 January 1991 and 28 February 1991. 69 studies (37%) considered military personnel deployed to Afghanistan (“Operation Enduring Freedom”). This US‐led operation began 10 October, 2001 and officially ended 31 December, 2014. 16 studies (9%) considered other deployments, mainly peacekeeping missions, in particular peacekeeping in the former Yugoslavia, under NATO‐command, to implement The General Framework Agreement for Peace in Bosnia and Herzegovina. Eleven studies did not report which operation participants had been deployed to. Table 2 gives a brief overview of the major operations considered in the included studies.

**Table 2:** Major military operations 1990 ‐ present day

Name of operation	Timespan of operation	Description
Operation Iraqi Freedom (Iraq)	2003‐2011	At 5:34 a.m. Baghdad time on 20 March 2003 the surprise military invasion of Iraq began. There was no declaration of war. The 2003 invasion of Iraq was led by U.S. Army General Tommy Franks, under the codename “Operation Iraqi Freedom”. The UK codename is “Operation Telic”, and the Australian codename is “Operation Falconer”. The war officially ended 18 December 2011. Civil conflict still on‐going. Part of the Global War on Terror.
Operation Enduring Freedom (Afghanistan)	2001‐2014	The US officially launched Operation Enduring Freedom on 7 October 2001 with the assistance of UK. The operation officially ended 31 December 2014. Part of the Global war on Terror.
The Gulf War (Kuwait, Iraq)	1990‐1991	The Gulf War, codenamed “Operation Desert Shield” (2 August 1990 – 17 January 1991) for operations leading to the build‐up of troops and defence of Saudi Arabia and “Operation Desert Storm” (17 January 1991 – 28 February 1991) in its combat phase, was a war ‐ in the Persian Gulf region ‐ waged by coalition forces from 34 nations led by the US against Iraq in response to Iraq's invasion and annexation of Kuwait.
United Nations Peacekeeping Missions^*^:		
Somalia	1992‐1995	The UN formed a peacekeeping mission to help bring stability to the region and allow relief supplies to reach those in desperate need of it. A further United States‐led multinational initiative was authorized by the UN in late 1992. Canada, along with more than 20 other nations, participated in this as well.
Bosnia‐Herzegovina	1995‐2000	The United Nations Mission in Bosnia and Herzegovina (UNMIBH) was an international organization formed under the United Nations Security Council Resolution 1035 on 21 December 1995. It completed its mandate on 31 December 2002, when it was succeeded by the European Union Police Mission in Bosnia and Herzegovina.
Kosovo	1999‐2002	The United Nations Interim Administration Mission in Kosovo (UNMIK) is the officially mandated mission of the United Nations in Kosovo. Currently, UNMIK describes its mandate as being to “help the Security Council achieve an overall objective, namely, to ensure conditions for a peaceful and normal life for all inhabitants of Kosovo and advance regional stability in the western Balkans.”

* *Only the most relevant for this review are shown*

74% of studies considered deployment of US troops, followed by the UK (11%). Other deploying countries in the sample were Australia, Canada, Denmark, Germany, Italy, Kuwait, Netherlands, Norway, Poland, Saudi Arabia and Sri Lanka (total of 28studies). One study did not report which country troops were deployed from.

Most studies (88%) considered deployment to war zones. The remaining studies (9%) considered deployment to civil conflict zones. Twenty studies did not report on the type of deployment.

62% of deployed participants was army personnel. A smaller share of deployed participants was personnel of other military branches: Navy (14%), Air Force (15%), and Marines (9%). 70 studies did not report on the service branch of participants.

At the study level 83% of the participants were males (30studies did not report on gender composition). Participants were 69% white Caucasian at the study level (not reported in 89studies), had an average age of 32 years (not reported in 52studies), and 56% were married (not reported in 72studies).

At the study level 30% of participants had previously been deployed (not reported in 141 studies). 54% of the deployed participants were from the Active Component (regulars). The remaining share of participants consisted either of members from the Reserve Component (reserves) or members of the National Guard (not reported in 67 studies). Finally, 84% of the participants (at the study level) were enlisted personnel (not reported in 77studies).

##### 4.1.2.1 *Study characteristics: synthesis sample*

Table 1 presents study characteristics for all included studies. Table 4 presents the study characteristics for those studies where an effect size could be calculated and used in the meta‐analysis. This sample of studies forms the basis for the synthesis. We therefore refer to this sample of studies as the synthesis sample. There are three reasons why a study may not be included in the synthesis sample. Some studies were not synthesised because the participants in the study were identical to those in another included study, and examined the same outcome construct. For example 9 studies were not in included in the synthesis sample because they all used an identical sample of participants from the Millennium Cohort Study (Ryan et al., 2007) and measured the same outcome. In such a case only one study was included in the synthesis sample to avoid double counting of participants. The second reason for studies not being synthesised was due to insufficient information to calculate an effect size. For example several studies provided an adjusted mean difference between treatment and control, but did not provide any raw standard deviation, or other statistics that would permit us to construct an effect size. The third reason for studies not being synthesised was receiving a score of 5 on any item in the risk of bias assessment. Studies that were scored with a 5 on some risk of bias item were included in a sensitivity analysis (where applicable).

In Table 3, we show the total number of studies that met the inclusion criteria for this review. The first column shows the total number of studies grouped by country. The second column gives the number of studies that were excluded from the data synthesis due to overlapping samples. The third column gives the number of studies that did not provide enough data to calculate an effect estimate. The fourth column shows the number of these studies that were coded with too high risk of bias to be included in the data synthesis (and could not be used in the sensitivity analysis). The fifth column gives the total number of studies used in the data synthesis, in total 40 studies. The last column gives the total number of studies coded with too high risk of bias used in the sensitivity analysis, in total 26 studies. Overall,54 studies could not be used in the synthesis due to overlapping data samples. The majority of those studies were from USA but the main reason for not using studies from USA in the synthesis was lack of information to calculate an effect size. Nearly half the studies from the UK could not be used in the synthesis due to overlapping data samples.

**Table 3:** Number of included studies

			**Reduction due to**			
**Country**	**Total**	**Overlap of data samples** ^2^	**Do not provide effect estimate** ^1^	**Too high risk of bias**	**Used in data synthesis** ^3^	**Too high risk of bias; used in sensitivity analysis**
Australia	7	3	2	1	1	
Canada	6	2	1	2		1
Denmark	3	2			1	
Germany	2	1			1	
Italy	1		1			
Kuwait	1					1
Netherlands	2		2			
Norway	1			1		
Poland	1					1
Saudi Arabia	1		1			
Sri Lanka	1			1		
UK	20	9	1		7	3
USA	139	37	40	12	30	20
***Total***	185	54	48	17	40	26

*Note: The reduction due tooverlap of data sample preceded the reduction due totoo high risk of bias*.

1 Or data that enable the calculation of an effect estimate.

2 The data samples used are identical to others used or are subgroups of data samples used (see Section 3.4.5 for this methodological issue).

The distribution of characteristics in the studies left for synthesis, shown in Table 4, was very similar to the distribution of study characteristics in the full sample of included studies. The table also provides information about outcome characteristics. A study may provide more than one effect size; for example reporting outcomes at different times since exposure or report outcomes based on a comparison of deployed to non deployed and relative combat exposure. In the mental health domain: 30 studies reported PTSD outcomes (34 effect sizes), 18 studies reported depression outcomes (19 effect sizes), nine studies reported outcomes in the domain of common mental disorders (11 effect sizes), and 14 studies reported outcomes related to substance use (16 effect sizes).

**Table 4:** Study characteristics of studies included in synthesis (40 studies)

**Study characteristics**					
**General characteristics**			**Participant characteristics**	Mean	SD
	Mean	SD			
Publication year	2007	5	Male (%)	86	17
			*not reported in 3studies*		
*Country of Study*	k	%			
UK	7	18	Caucasian (%)	72	15
USA	29	74	*not reported in 16studies*		
Other countries*	3	8			
			Age (years)	32	5
*Australia (1), Denmark (1), Germany (1)			*not reported in 18studies*		
					
*Mission location*	k	%	Married (%)	56	15
Gulf War	15	38	*not reported in 10studies*		
OIF	7	18			
OEF	2	5	Previously deployed (%)	35	31
OIF and OEF	14	36	*not reported in 28 studies*		
Other locations*	1	3			
*Bosnia (1)			Active Component (%)	50	41
			*not reported in 9 studies*		
*Deployment type*	k	%	* *		
War zone	35	90	Enlisted (%)	86	9
Civil conflict zone	1	3	*not reported in 10 studies*		
*not reported in 3studies*					
			**Outcome characteristics**	n	k
*Military service branches*^1^ (%)	Mean	SD	PTSD	34	30
Army	59	39	Depression	19	18
Navy	16	28	CMD	11	9
Air force	16	26	Substance use	16	14
Marine	10	25			
*not reported in 9 studies*					

Notes: *k = number of studies, n = number of effect sizes*. A study may provide more than one effect size (outcome, time since exposure or comparison non deployed and relative combat exposure). Per cents and averages taken only for studies reporting the characteristic in question.

1 The United States Armed Forces consist of the main service branches: Army, Marine Corps, Navy, Air Force, and Coast Guard. The British Armed Forces consist of the main service branches: Royal Navy (Navy), Royal Marines (Marine), British Army (Army), and Royal Air Force (Air Force). The Australian Defence Force consists of the main service branches: Royal Australian Navy (Navy), Australian Army (Army), and Royal Australian Air Force (Air Force).

##### 4.1.2.2 *Type of outcomes*

In this section we report how studies measured outcome constructs. The tables in section 9.2 describe which instruments studies used to identify relevant outcomes. Only a minority of studies used structured clinical interviews to diagnose disorders. Instead the majority instruments were self‐administered validated instruments where a score indicates the severity of symptoms. When instruments are validated it is possible to use a cutoff score to indicate probable diagnosis of the disorder (caseness). Below we present the major instruments used in the included studies, and we also describe what cutoff rules were applied in the studies to indicate caseness.

###### 4.1.2.2.1 *PTSD*

Sixteen studies in the synthesis sample used a version of the PTSD Checklist – PCL to assess severity of PTSD symptoms. The instrument gives a score which indicates the severity of PTSD symptoms; it is not a clinical diagnosis of PTSD. The PCL is a 17‐item screen developed and validated by Weathers, Litz, Harmn, Huska, &Keane (1993)on a sample of treatment seeking Vietnam veterans. The scale corresponds to the three clusters of DSM‐IV symptoms: re‐experiencing, avoiding stimuli, and hyper arousal. Responders answer whether they have been bothered by symptoms from not at all (1) to extremely (5). Accordingly the scale ranges from 17 to 85. Weathers, et al. (1993) recommended a cutoff of 50 for probable PTSD. In the synthesis sample 10 studies used this cutoff, 1 study used a more stringent cutoff consisting of a cutoff score of 50 and endorsement of one or more symptoms of PTSD. Two studies used a less stringent cutoff than 50 to indicate probable PTSD. Two studies reported an (adjusted) difference in the raw PCL score. The raw standard deviation and the adjusted difference score was used to construct Cohen's *d*, and was thereafter transformed to an odds ratio. One study did not report the cutoff rule.

Four studies used the Primary Care Posttraumatic Stress Disorder Screen (PC‐PTSD). The PC‐PTSD is a four item screen developed by Prins, et al. (2004). The recommended cutoff for probable PTSD is 2 or 3 (Prins et al., 2004; Bliese, Wright, Adler, Cabrera, Castro, & Hoge, 2008). Two studies used a cutoff of 2, one study used a cutoff of 3 and one study did not report the cutoff.

Two studies used hospitalization records; four studies used clinical structured interviews (three SCID, one CIDI). Remaining effect sizes used self‐developed questionnaires (2) and Mississippi scale (2).

###### 4.1.2.2.2 *Depression*

Six studies in the synthesis sample measured Depression with the Patient Health Questionnaire – PHQ (Kroenke, & Spitzer, 2002). Two different versions were used in the studies, a nine item version, PHQ‐9, and a two item version, the PHQ‐2. The PHQ‐2 uses the first two questions from the PHQ‐9. Major depressive disorder, as measured by nine items from the PHQ‐9, corresponds to the depression diagnosis from the DSM‐IV. Depression cannot be diagnosed with the PHQ, but it can be used to indicate possible depression. Participants use a 4‐point Likert scale to rate the severity of each depressive symptom from “not at all” to “nearly every day” during the previous two weeks prior to questionnaire completion. On the PHQ‐2 the responder is asked to rate the two questions: 1) little interest or pleasure in doing things, and 2) feeling, down, depressed, or hopeless, on the scale: not at all (0), several days (1), more than half the days (2), and nearly every day (3). Thus, the PHQ‐2 score can range from zero to six. Löwe, Kroenke, and Gräfe, K. (2005) recommend a cutoff score of 3. The PHQ‐9 was validated by Löwe et al. (2004). Several thresholds are recommended. Specifically they recommend cutoff scores of 9, 10, 11, as well as a categorical algorithm for detecting probable depression. The distribution of cutoffs for effect measures using the PHQ‐9: one reported using a cutoff score of 5, two reported using a standard cutoff, and one did not report how a case was detected. Two studies used the PHQ‐2.

Four studies used structured interviews (two SCID, two CIDI) for detecting depression, one study used the Beck Depression Inventory – BDI (Beck, Ward, Mendelson, Mock, & Erbaugh, 1961) where the effect measure was derived from raw scores, and two effect measures used a cutoff score of 9. The remaining studies measuring depression used: questionnaires (n=4),the CES depression scale (n=2), and the Primary Care Evaluation of Mental Disorders screening questionnaire for depressive symptoms (n=1).

###### 4.1.2.2.3 *Substance abuse or dependence*

Studies that measured and reported outcomes in this domainmost frequently used non‐validated instruments to assess probable substance abuse (four studies). Five studies used structured interviews. CIDI was used in four studies. Two of these used DSM‐IV criteria, and two did not report specific criteria. The SCID was used in one study (used DSM‐III‐R criteria). The alcohol abuse screen WHO‐AUDIT screen was used in four studies. Of these, two studies used a cutoff score of 10, and one used a cutoff score of 16 and one study stated that at‐risk drinking was defined as a score of at least 3 out of a possible 12. The remaining study used PRIME‐MD.

###### 4.1.2.2.4 *Common mental disorders*

Common mental disorders refer to any depression, anxiety and somatisation disorder. That is, studies that reported on this outcome aimed to identify a broader category of mental affliction. The most common instrument used was the GHQ. Five studies used the 12‐item GHQ‐12. The GHQ is a self‐report instrument for the detection of mentaldisorders in the community and among primary care patients. Scores per item range from zero to three, and are summed to produce a total score. Three studies used a cutoff of 4. One study used a lower threshold, and one study did not report the cutoff used. One study derived the effect measure from the BSI (Brief Symptom Inventory). A global severity index (GSI) can be created from the BSI, which was reported as a raw score.

Other measures used in this domain were: hospitalization (one study), MCS (one study), and the CIDI (one study).

### 4.2 RISK OF BIAS IN INCLUDED STUDIES

Studies were assessed for risk of bias^11^. Each study was rated in the following seven domains:

- Adequate sequence generation
- Allocation concealment
- Blinding
- Incomplete outcome data addressed
- Reporting bias
- Other bias
- Confounding

When a study was judged very high risk of bias (a score of five) on any of the four domains: incomplete data, reporting, other bias, and confounding it was excluded from synthesis, but included (where applicable) in sensitivity analysis. All included studies used observational data. Accordingly all included studies were rated with a high risk of bias on the adequate sequence generation and allocation concealment items.

On the Blinding item two studies were rated 2/5, nine studies were rated 3/5, 168 studies were rated 4/5 and one study was rated Unclear. No studies received a score of one or five on this item.

On the Incomplete outcome data item 14 studies were scored with a low risk of bias (1/5), 47 studies were rated 2/5, 47 studies were rated 3/5, 27 studies were rated 4/5 and one study was rated 5/5. 44 studies were rated with an unclear risk of bias on this item..

135 studies were rated 1/5 (low risk of bias) on the Reporting item, while 14 studies each received a rating of 2/5 or 4/5 and 15 studies received a rating of 3/5 (total of 43 studies). Two studies were rated with an unclear risk of bias and no studies were rated as 5/5.

126 studies were rated 1/5 on the Other bias item. 39 studies were rated 2/5, and six and four studies were rated 3/5 and 4/5 respectively. Five studies received an unclear risk of bias rating on this item and no studies were rated 5/5.

All studies used observational data to measure the effect of deployment. Accordingly all studies were assessed on the Confounding item. 95 studies were scored 5/5 on this item. As a result these studies were excluded from the main analysis, but were included in a sensitivity analysis (where appropriate). Fifty four studies were rated 4/5 on this item. Twenty one studies were rated 3/5, 9 studies were rated 2/5, and one study was rated 1/5.

**Figure 2:**
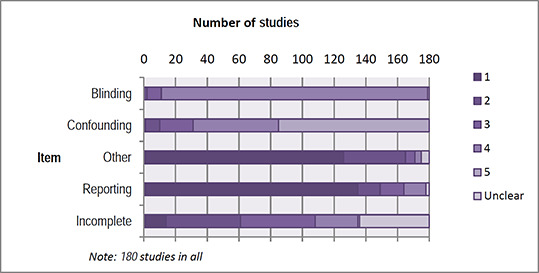
Risk of bias assessment

Figure 2 gives an overview of the risk of bias assessment by item.

### 4.3 SYNTHESIS OF RESULTS

The analyses presented in this section restrict attention to those studies that permitted extraction of an effect size, and were not rated 5/5in any of the four domains where risk of bias judgement was given on a scale 1 – 5. Forty studies were synthesised.

All effect sizes were synthesised as log odds ratios. Effect sizes were coded such that a larger effect size indicated worse outcomes for the deployed group, or the group with higher combat exposure (poorer mental health, poorer social functioning). Effect sizes that were derived from continuous measures were converted to log odds ratios using the methods outlined in section 3.4.2.3. In the graphical displays log odds ratios are back transferred.

We synthesise deffects separately by comparison, type of outcome, and time since exposure.

#### 4.3.1 Absolute comparison: Deployment versus non‐deployment

##### 4.3.1.1 *Post‐traumatic stress disorder*

**Figure 3:**
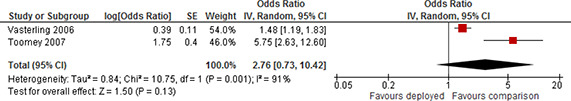
Forest plot, PTSD, deployment versus non‐deployment, 0‐6 months post deployment, odds ratio

**Figure 4:**
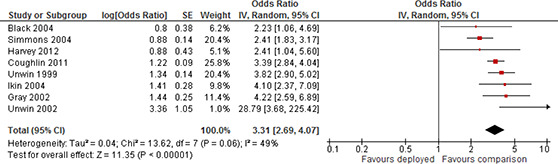
Forest plot, PTSD, deployment versus non‐deployment, more than 24 months post deployment, odds ratio

**Figure 5:**
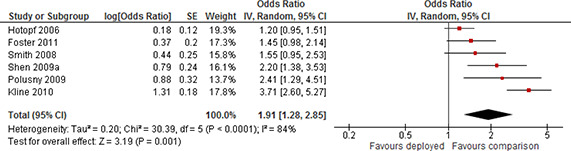
Forest plot, PTSD, deployment versus non‐deployment, variable number of months post deployment, odds ratio

Figures 3–5 present forest plots for the outcome: post‐traumatic stress disorder (PTSD) for the comparison between a group that deployed to a military operation and a group that did not deploy. Each study contributed at most one effect size^12^. For each study the figures show the effect size and the weight of the study in the average effect size. The average effect size with 95% confidence interval is shown as a diamond.

Two studies contributed effect sizes for assessments taken 0 to 6 months post deployment (“short”). Both studies reported an odds ratio greater than one which means that the deployed group faced an increased risk of screening positive for PTSD compared to the non‐deployed group. Both effect sizes were statistically significant. However, the effect sizes differed substantially in magnitude. The degree of heterogeneity was accordingly very large and the estimated τ^2^ of 0.84 and I^2^ (91%) indicated that the evidence was inconsistent. The evidence for assessments taken 0 to 6 months post deployment was inconclusive. The forest plot is displayed in Figure 3.

No studies contributed data to assessments 6 – 24 months post deployment (“medium”).

Eight studies contributed evidence to assessments taken more than 24 months since exposure (“long”). All reported results indicated a positive effect favouring the non‐deployed; all of the study‐level effects were statistically significant. This means that the deployed group had an increased risk of screening positive for PTSD compared to the non‐deployed group.

The weighted average was positive and statistically significant. The random effects weighted mean odds ratio was 3.31 (95% CI 2.69 to 4.07). The forest plot is displayed in Figure 4. There was a moderate degree of heterogeneity between the studies; the estimated τ^2^ was 0.04 and I^2^ was 49%.

There were six studies that contributed evidence at “other” times since exposure. Most of the studies in this category followed cohorts of military personnel who were not necessarily deployed at the same time. Accordingly the time since exposure ranged from short to long. All reported results indicated a positive effect favouring the non‐deployed; three of the study‐level effects were statistically non‐significant and three of the study‐level effects were statistically significant. The weighted average was positive and statistically significant. The random effects weighted mean odds ratio was 1.91 (95% CI 1.28 to 2.85). The forest plot is displayed in Figure 5. The degree of heterogeneity between the studies was large; the estimated τ^2^ was 0.20 and I^2^ was 84%.

In summary, meta‐analysis indicated that the risk of screening positive for PTSD increased for participants in the deployed group compared to participants in the group that did not deploy. For assessments taken more than 24 months past exposure the evidence consistently suggest large adverse effects of deployment on the risk of screening positive for PTSD.

Evidence was less consistent for assessments taken at other time points. The average effects were smaller in size to the average effect 24 months since exposure, and there was a high degree of heterogeneity between the studies. For assessments taken 0 to 6 months past exposure the evidence was inconclusive as only two studies contributed effect sizes and the degree of heterogeneity between studies was very large. No studies contributed data in the medium term.

##### 4.3.1.2 *Depression*

A total of 12 studies contributed data to the synthesis of the effect of deployment on depression for participants that deployed to a military operation compared to participants that did not deploy. When a study contributed more than one effect size, for example because participants were assessed at several time points since exposure, effect sizes were averaged to create a single synthetic effect size. Meta‐analysis was conducted separately by time since exposure.

**Figure 6:**
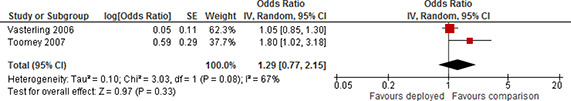
Forest plot, depression, deployed vs non‐deployed, 0‐6 months post deployment, odds ratio

Two studies screened participants for depression 0 to 6 months post deployment (“short”). Both studies reported an odds ratio greater than one; one of the study level effects was statistically significant and one was statistically non‐significant. The weighted average was positive and statistically non‐significant. The random effects weighted mean odds ratio was 1.29 (95% CI 0.77 to 2.15). The forest plot is displayed in Figure 6. The degree of heterogeneity between the studies was moderate; the estimated τ^2^ was 0.10 and I^2^ was 67%.

One study contributed assessments for depression 6 to 24 months post deployment. The odds ratio effect size was1.05, which was not statistically significant (95%CI: 0.83 to 1.33).

**Figure 7:**
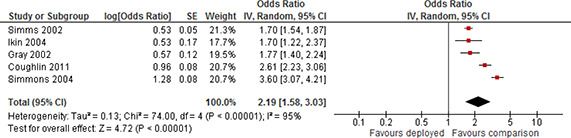
Forest plot, depression, deployed versus non‐deployed, more than 24 months post deployment, odds ratio

Five studies contributed data to the long term assessment for depression. All reported results indicated a positive effect favouring the non‐deployed; all the study‐level effects were statistically significant. The weighted average was positive and statistically significant. The random effects weighted mean odds ratio was 2.19 (95% CI 1.58 to 3.03). The forest plot is displayed in Figure 7. The degree of heterogeneity between the studies was large; the estimated τ^2^ was 0.13 and I^2^ was 95%.

**Figure 8:**
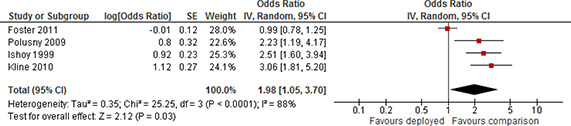
Forest plot, depression, deployment versus non‐deployment, variable number of months post deployment, odds ratio

Finally four studies contributed data at “other” times since exposure. The evidence was mixed; one study reported a negative non‐significant effect and three studies reported positive and statistically significant effects. The weighted average was positive and statistically significant. The random effects weighted mean odds ratio was 1.98 (95% CI 1.05 to 3.70). The forest plot is displayed in Figure 8. The degree of heterogeneity between the studies was large; the estimated τ^2^ was 0.35 and I^2^ was 88%.

In summary, meta‐analysis indicated that the risk of screening positive for depression increased for participants in the deployed group compared to participants in the group that did not deploy. However the random effects weighted mean effect was statistically significant only for participants assessed more than 24 months since exposure. For assessments taken more than 24 months past exposure the evidence consistently suggest large adverse effects of deployment on the risk of screening positive for depression. Evidence was less consistent for assessments taken at other time points. There was a high degree of heterogeneity between the studies and one study reported a negative, although non‐significant, point estimate. For assessments taken 0 to 6 months past exposure the evidence was inconclusive as only two studies contributed an effect size.

##### 4.3.1.3 *Substance use*

Primary studies measured both alcohol and illicit substance dependence in a variety of ways. Outcomes were pooled under “substance use”. A total of 10 studies contributed data to the synthesis of the effect of deployment on substance use for participants that deployed to a military operation compared to participants that did not deploy.

For both the short (0‐6 months since deployment) and medium term 6‐24 months since deployment) only one study each contributed an effect size. Both these effects suggested that risk of substance use is higher for participants that deployed to a military operation compared to participants that did not deploy, but effects are not statistically significant. The odds ratio for the short term was 1.15 (95% CI 0.61 to 2.15) and for the medium term the odds ratio was 1.07 (95% CI 0.44 to 2.59)

**Figure 9:**
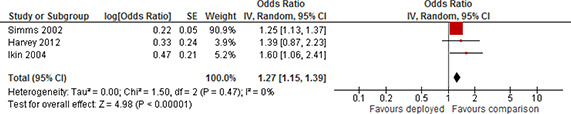
Forest plot, substance use, deployment versus non‐deployment, more than 24 months post deployment, odds ratio

Three studies contributed data to the long term assessment for substance use. All reported results indicated a positive effect favouring the non‐deployed; two study‐level effects were statistically significant and one was non‐significant. The weighted average was positive and statistically significant. The random effects weighted mean odds ratio was 1.27 (95% CI 1.15 to 1.39). The forest plot is displayed in Figure 9. There was no heterogeneity between the studies; the estimated τ^2^ was 0.00 and I^2^ was 0%.

**Figure 10:**
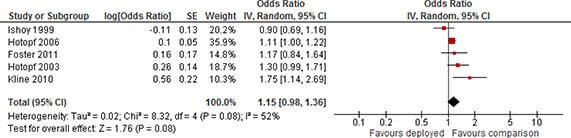
Forest plot, substance use, deployment versus non‐deployment, variable number of months post deployment, odds ratio

Finally five studies contributed data at “other” times since exposure. The evidence was mixed; one study reported a negative non‐significant effect and four studies reported positive effects; two were statistically significant and two were statistically non‐significant. The weighted average was positive and statistically non‐significant. The random effects weighted mean odds ratio was 1.15 (95% CI 0.98 to 1.36). The forest plot is displayed in Figure 10. The degree of heterogeneity between the studies was moderate; the estimated τ^2^ was 0.02 and I^2^ was 52%

In summary, the evidence of whether deployed participants have higher odds of screening positive for substance use compared to non‐deployed participants was mixed. For assessments up to 24 months past exposure (“short” and “medium”) no meta‐analysis could be performed. For assessments taken at least 24 months since exposure the evidence was stronger and showed deployed participants had higher odds of screening positive for substance use compared to non‐deployed participants. For assessments taken at “other” time points, the average effect was positive but statistically non‐significant and less consistent than the long term evidence.

##### 4.3.1.4 *Common mental disorders*

In this section we consider a broader category of mental disorders: common mental disorders. The outcome covers mood disorders (including depression), anxiety disorders (including PTSD)^13^, and somatoform disorders (ICD‐10 classifications: F32‐33, F40‐43, and F45).

Meta‐analysis was conducted separately by time since exposure. No studies contributed data to short term assessment and no studies contributed data to the medium term.

**Figure 11:**
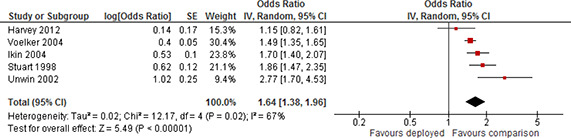
Forest plot, common mental disorder, deployment versus non‐deployment, more than 24 months post deployment, odds ratio

Five studies contributed data to long term outcomes. All studies contributed an effect size above 1, indicating that the deployed group fared worse than the comparison group; one of the effects was statistically non‐significant. The weighted average was positive and statistically significant. The random effects weighted mean odds ratio was 1.64 (95% CI 1.38 to 1.96). The forest plot is displayed in Figure 11. The degree of heterogeneity between the studies was moderate; the estimated τ^2^ was 0.02 and I^2^ was 67%.

**Figure 12:**
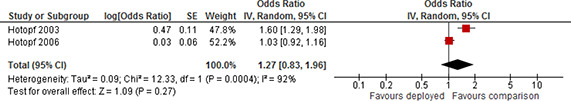
Forest plot, common mental disorder, deployment versus non‐deployment, variable number of months post deployment, odds ratio

Finally two studies contributed data at “other” times since exposure. Both studies reported a positive effect; one was statistically significant and one was statistically non‐significant. The weighted average was positive and statistically non‐significant. The random effects weighted mean odds ratio was 1.27 (95% CI 0.83 to 1.96). The forest plot is displayed in Figure 12. The degree of heterogeneity between the studies was large; the estimated τ^2^ was 0.09 and I^2^ was 92%.

In summary, only few studies reported on common mental disorders. No assessments were available 0 to 6 and 6 to 24 months past exposure (“short” and “medium”). The average effect size for assessments taken at “other” times was positive but not statistically significant. At 24 months since exposure the mean odds ratio was positive and statistically significant, implying on average an increased risk of screening positive for common mental disorders for deployed participants compared to non‐deployed participants.

##### 4.3.1.5 *Social functioning: employment and homelessness*

Two studies assessed the effect of deployment on employment comparing a group of deployed participants to a group of non‐deployed participants (Edwards, 2012; Horton, et al., 2013). Both studies were excluded from synthesis after risk of bias assessment. We did not identify any studies that reported on homelessness.

#### 4.3.2 Relative comparison: Deployed participants stratified by combat exposure

This section considers comparisons where all participants were deployed. The comparison is relative, because all participants were deployed, but participants differed in their exposure to risk of trauma. Exposure might be self‐reported for example via questionnaires where participants reported combat exposure. Alternatively, relative exposure was a result of studies comparing one group of participants that deployed to a combat‐zone to another group of participants that were deployed to a non‐combat zone for example deployment to a US military base in Germany.

We would expect substantial between study heterogeneity for these different comparisons, because there was substantial variation in how studies measured combat exposure, the time between potential exposure and reporting, and how participants were stratified by combat exposure (for example, low versus high, or low versus medium). Almost all studies assessed combat exposure via survey questions to participants. As such responses are prone to recall bias, and bias is likely to increase with time since exposure. Responses may also interact with the condition. For example part of the diagnosis of PTSD involves reliving traumatic experiences. As a result participants with the condition may be more likely to recall combat exposure compared to participants that had the same exposure, but no adverse reaction to it.

##### 4.3.2.1 *Post‐traumatic stress disorder*

**Figure 13:**
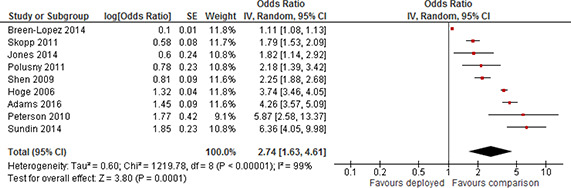
Forest plot, PTSD, high combat exposure versus low combat exposure, 0‐6 months post deployment, odds ratio

Nine studies contributed evidence to the comparison up to 6 months since exposure (“short”). All study level effects were positive and statistically significant. The mean odds ratio effect size was 2.74, which was statistically significant (95% CI: 1.63 to 4.61). This means that the group of participants who reported higher combat exposure hadhigher odds of screening positive for PTSD. The forest plot is displayed in Figure 13 There was substantial heterogeneity as indicated by both *I* ^2^ (99%) and τ^2^ (0.60).

Only one study contributed evidence for 6 to 24 months since exposure (“medium”). The odds ratio effect size was 36.97, which was statistically significant (95%CI: 2.07 to 659.28).

**Figure 14:**
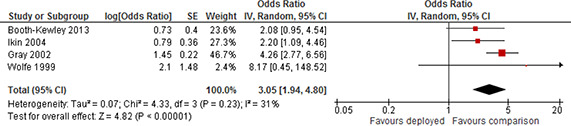
Forest plot, PTSD, high combat exposure versus low combat exposure, more than 24 months post deployment, odds ratio

Four studies contributed data to long term outcomes. All studies contributed an effect size above 1, indicating that the deployed group fared worse than the comparison group; one of the effects was statistically non‐significant. The weighted average was positive and statistically significant. The random effects weighted mean odds ratio was 3.05 (95% CI 1.94 to 4.80). The forest plot is displayed in Figure 14. The degree of heterogeneity between the studies was small; the estimated τ^2^ was 0.07 and I^2^ was 31%.

**Figure 15:**
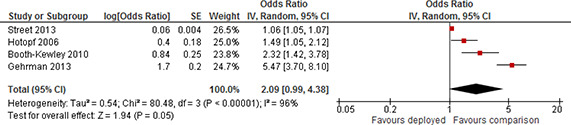
Forest plot, PTSD, high combat exposure versus low combat exposure, variable number of months post deployment, odds ratio

Four studies contributed data to other time point. All studies contributed an effect size above 1, indicating that the deployed group fared worse than the comparison group; all of the effects were statistically significant. However, the degree of heterogeneity between the studies was substantial; the estimated τ^2^ was 0.54 and I^2^ was 96%. The weighted average was positive but not statistically significant. The random effects weighted mean odds ratio was 2.09 (95% CI 0.99 to 4.38). The forest plot is displayed in Figure 15.

In summary, across the different time points mean odds ratio effect sizes all indicated that participants reporting high combat exposure had a higher odds of screening positive for PTSD than participants reporting lower exposure. The average odds ratio was however not statistically significant for assessments made for other time points and the degree of heterogeneity was substantial. For short term outcomes the degree of heterogeneity was also substantial. For long term assessments, more than 24 months since exposure, the average effect was statistically significant, and the evidence was consistent.

##### 4.3.2.2 *Depression*

Seven studies contributed evidence to this comparison.

**Figure 16:**
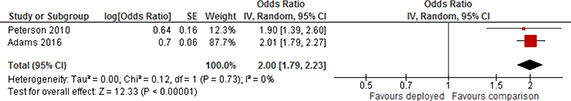
Forest plot, depression, high combat exposure versus low combat exposure, 0‐6 months post deployment, odds ratio

Two studies reported effects up to 6 months past exposure. The random effects weighted mean odds ratio was 2.00, which was statistically significant (95% CI: 1.79 to 2.23). The forest plot is displayed in Figure 16. There was no evidence between study heterogeneity; the estimated τ^2^ was 0.00 and I^2^ was 0%.

One study each reported effects 6‐24 months past exposure (odds ratio 6.62; 95% CI: 0.35 to 125.20) and for other time points since exposure (odds ratio 3.86; 95% CI: 2.18 to 6.81).

**Figure 17:**
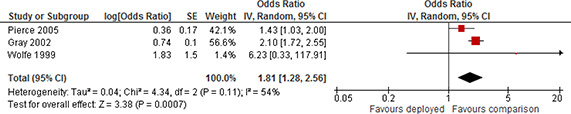
Forest plot, depression, high combat exposure versus low combat exposure, more than 24 months post deployment, odds ratio

Three studies reported effects more than 24 months since exposure. All effects were positive, two were statistically significant and one was statistically non‐significant. The weighted average was positive and statistically significant. The random effects weighted mean odds ratio was 1.81, which was statistically significant (95% CI: 1.28 to 2.56). The forest plot is displayed in Figure 17. There was moderate between study heterogeneity; the estimated τ^2^ was 0.04 and I^2^ was 54%.

In summary meta‐analysis could only be performed for short and long term outcomes. The average effects were positive indicating that participants who reported high combat exposure had an increased risk of screening positive for depression compared to participants reporting lower exposure.

##### 4.3.2.3 *Substance use*

**Figure 18:**
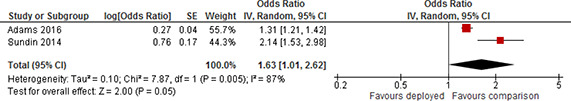
Forest plot, substance use, high combat exposure versus low combat exposure, 0‐6 months post deployment, odds ratio

Six studies contributed effect sizes to this comparison. Evidence could only be synthesised for short and “other” time periods, where two studies each contributed data. For the other comparisons only one study each contributed data (“medium” odds ratio 5.05, 95% CI: 0.26 to 99.40 and “long” odds ratio 1.60, 95% CI: 0.85 to 3.00). The weighted average for 0‐6 months post deployment was positive and statistically significant. The random effects weighted mean odds ratio was 1.63, which was statistically significant (95% CI: 1.01 to 2.62). However, the degree of heterogeneity between the studies was large; the estimated τ^2^ was 0.10 and I^2^ was 87%. The forest plot is displayed in Figure 18.

**Figure 19:**
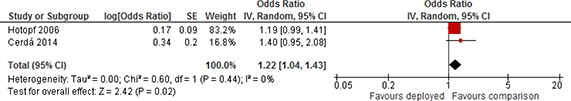
Forest plot, substance use, high combat exposure versus low combat exposure, variable number of months post deployment, odds ratio

The weighted average for other time points was positive and statistically significant. The random effects weighted mean odds ratio was 1.22, which was statistically significant (95% CI: 1.04 to 1.43). The forest plot is displayed in Figure 19. There was no indication of between study heterogeneity; the estimated τ^2^ was 0.00 and I^2^ was 0%.

In summary meta‐analysis could only be performed for short and other time points and only two studies each contributed data at these time points. The average effects were positive indicating that participants who reported high combat exposure had an increased risk of screening positive for substance use compared to participants reporting lower exposure.

##### 4.3.2.4 *Common mental disorders*

**Figure 20:**
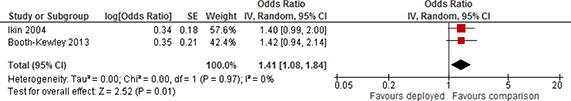
Forest plot, common mental disorder, high combat exposure versus low combat exposure, more than 24 months post deployment, odds ratio

Four studies contributed effect sizes to this comparison. Evidence could only be synthesised for long term outcomes, where two studies contributed data. No studies contributed data to the medium term. For the other comparisons only one study each contributed data (“short” odds ratio 1.00, 95% CI: 0.78 to 1.29; and “other” odds ratio 1.04, 95% CI: 0.86 to 1.27). The weighted average for assessments taken more than 24 months since exposure was positive and statistically significant. The random effects weighted mean odds ratio was 1.41, which was statistically significant (95% CI: 1.08 to 1.84). The forest plot is displayed in Figure 20. There was no indication of between study heterogeneity; the estimated τ^2^ was 0.00 and I^2^ was 0%.

In summary meta‐analysis could only be performed for long term outcomes and only two studies contributed data at this time point. The average effect was positive indicating that participants who reported high combat exposure had an increased risk of screening positive for common mental disorders compared to participants reporting lower exposure.

##### 4.3.2.5 *Social functioning: employment and homelessness*

We did not identify any studies that reported on employment outcomes for this comparison. One study assessed the effect of deployment on homelessness (Elbogen, Sullivan Wolfe, Wagner, & Beckham, 2014). The study was excluded from synthesis after risk of bias assessment.

#### 4.3.3 Summary

##### PTSD

Meta‐analysis indicated that the risk of screening positive for PTSD increased for participants in the deployed group compared to participants in the group that did not deploy for assessments taken more than 24 months past exposure. Likewise, participants reporting high combat exposure had a higher odds of screening positive for PTSD than participants reporting lower exposure for long term assessments. More than 24 months since exposure, the average effects were large and statistically significant, and the evidence was consistent for both types of comparisons.

Evidence was less consistent for assessments taken in the short term and at other time points. For assessments taken 0 to 6 months past exposure the evidence was inconclusive as the degree of heterogeneity between studies was very large for both types of comparisons. For assessments taken at other time points the evidence was inconclusive for the relative comparison as the degree of heterogeneity between studies was very large. For the absolute comparison the average effect was positive and statistically significant but there was a substantial degree of heterogeneity between the studies.

The evidence in the medium term was inconclusive as only one study contributed data in the medium term.

##### Depression

Meta‐analysis indicated that for long term outcomes the risk of screening positive for depression increased for participants in the deployed group compared to participants in the group that did not deploy. The evidence consistently suggested adverse effects of deployment on the risk of screening positive for depression, although there was a high degree of heterogeneity between studies. Likewise, meta‐analysis of long term outcomes indicated that participants who reported high combat exposure had an increased risk of screening positive for depression compared to participants reporting lower exposure, and there was only a moderate degree of heterogeneity between studies. For assessments taken at other time points the evidence suggested an increased risk for the deployed group compared to the non‐deployed group, although less consistently and with a high degree of heterogeneity between studies.

The remaining analyses were inconclusive due to too few studies.

##### Substance use

The evidence of whether deployed participants have a higher odds of screening positive for substance use compared to non‐deployed participants was scarce. For assessments taken at least 24 months since exposure the evidence consistently showed deployed participants had a higher odds of screening positive for substance use compared to non‐deployed participants. For assessments taken at “other” time points, the average effect was positive but statistically non‐significant and less consistent than the long term evidence.

The remaining analyses were inconclusive due to too few studies.

##### Common mental disorder

Only a few studies reported on common mental disorders. Deployed participants had an increased risk of screening positive for common mental disorders compared to non‐deployed participants, at least 24 months past deployment.

The remaining analyses were inconclusive due to too few studies.

#### 4.3.4 Moderator analysis

The included studies differed in terms of their sample characteristics, comparison conditions, and methodology. With between two and nine studies in a single meta‐analysis, the statistical power to detect heterogeneity of effects was quite low; nevertheless, evidence of statistical heterogeneity was found; in some analyses it was substantial.

With the aim of explaining observed heterogeneity, we investigated the following factors: Study‐level summaries of participant characteristics: mental health history, gender, age, ethnicity, military rank, branch of service, and duty/enlistment status.

Among the studies used in the data synthesis, only three studies reported mental health history. Only one study reported results separated by gender, two studies analysed females only and the majority of participants in the remaining studies were male; there was very little variation in this covariate. Ethnicity and age were not reported in many studies (16 and 18 studies respectively) and there was almost no variation in these covariates among the studies reporting them. Military rank was reported in all but ten studies but there was very little variation in this covariate among studies reporting it. Branch of service was reported in all but nine studies. Eleven studies used an Army only sample and in addition two studies reported separate estimates on branch of service. Duty/enlistment status was reported in all but nine studies. Nine studies used samples of Reserves/Guards only, six studies used samples of Regulars only; the remaining studies (reporting this covariate) used samples with on average 69% Regulars and in addition three studies reported separate estimates for Reserves/Guards and Regulars.

Where possible, subgroup analyses were performed using effect estimates separated by Army versus other branches of service and Reserves/Guards versus not Reserves/Guards. We only performed subgroup analysis if there were at least two studies in each subgroup.

Most of the results rely strictly on variation *between* studies, not within. Making inferences about different effect sizes among subgroups on the basis of between‐study differences entails a higher risk compared to inferences made on the basis of within study differences (Oxman & Guyatt, 1992). One should therefore be careful when interpreting estimates that rely on variation between studies.

We have drawn no overall conclusion because the analysis is based on a subset of the meta‐analyses. The assessment of any difference between the subgroups is based on 95% confidence intervals and interpretation of relationships is cautious.

##### 4.3.4.1 *Absolute comparison: Deployment versus non‐deployment*

###### 4.3.4.1.1 *Post‐traumatic stress disorder*

It was not possible to perform sub group analysis for short term outcomes.

Of the eight studies providing effect estimates for long term outcomes, one study reported results separated by Reserves/Guards and Regulars and one study used a sample of Reserves/Guards only.

**Figure 21:**
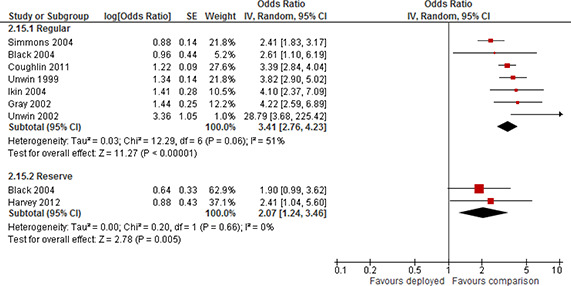
Forest plot, subgroup Reserve/Guard and Regular, PTSD, deployment versus non‐deployment, more than 24 months post deployment, odds ratio

The forest plot for the nine effect estimates is displayed in Figure 21. Pooled results for the two subgroups showed a statistically significant positive effect; odds ratio=3.41(95% CI 2.76 to 4.23) for Regulars and a statistically significant positive effect; odds ratio=2.07 (95% CI 1.24 to 3.46) for Reserves/Guards. There was a moderate degree of heterogeneity of effects among studies in the subgroup of Regulars (τ^2^=0.03, I^2^=51%) and no heterogeneity for Reserves/Guards (τ^2^=0.00, I^2^=0%). The confidence intervals of the subgroups overlapped. There was no evidence to support the hypothesis that the long term effect differs by duty/enlistment status (Reserves/Guards versus Regulars).

**Figure 22:**
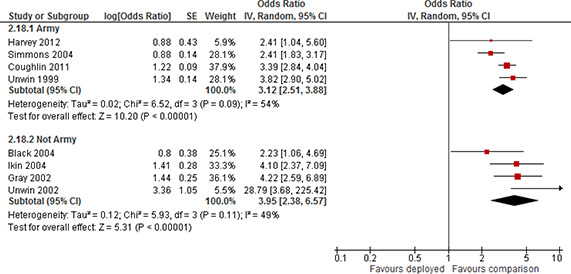
Forest plot, subgroup Army and Not Army, PTSD, deployment versus non‐deployment, more than 24 months post deployment, odds ratio

Four studies used a sample where the majority of participants were Army personnel (64% to 78%). The forest plot for the eight effect estimates is displayed in Figure 22. Pooled results for the two subgroups showed a statistically significant positive effect; odds ratio=3.12 (95% CI 2.51 to 3.88) for Army and a statistically significant positive effect; odds ratio=3.95 (95% CI 2.38 to 6.57) for not Army. There was a moderate degree of heterogeneity of effects among studies in the subgroup of Army (τ^2^=0.02, I^2^=54%) and in the sub group of not Army (τ^2^=0.12, I^2^=49%). The confidence intervals of the subgroups overlapped. There was no evidence to support the hypothesis that the long term effect differs by branch of service (Army versus other).

Of the six studies providing effect estimates for other time points, one study reported results separated by Reserves/Guards and Regulars and three studies used a sample of Reserves/Guards only.

**Figure 23:**
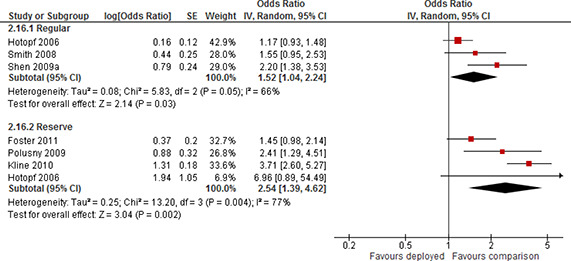
Forest plot, subgroup Reserve/Guard and Regular, PTSD, deployment versus non‐deployment, variable number of months post deployment, odds ratio

The forest plot for the seven effect estimates is displayed in Figure 23. Pooled results for the two subgroups showed a statistically significant positive effect; odds ratio=1.52(95% CI 1.04 to 2.24) for Regulars and a statistically significant positive effect; odds ratio=2.54 (95% CI 1.39 to 4.62) for Reserves/Guards. There was a moderate degree of heterogeneity of effects among studies in the subgroup of Regulars (τ^2^=0.08, I^2^=66%) and a high degree of heterogeneity for Reserves/Guards (τ^2^=0.25, I^2^=77%). The confidence intervals of the subgroups overlapped. There was no evidence to support the hypothesis that the effect at other time points differs by duty/enlistment status (Reserves/Guards versus Regulars).

**Figure 24:**
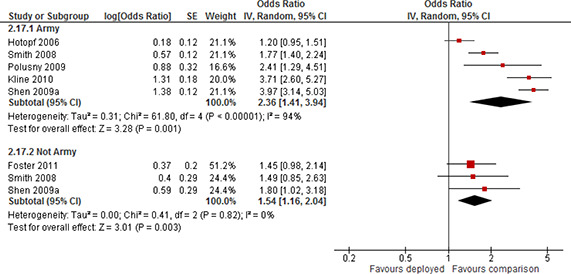
Forest plot, subgroup Army and Not Army, PTSD, deployment versus non‐deployment, variable number of months post deployment, odds ratio

Two studies reported results separated on branch of service, one study used a sample where the majority of the participants were Army personnel (64%) and two studies used a sample where all participants were Army personnel. The forest plot for the eight effect estimates is displayed in Figure 24. Pooled results for the two subgroups showed a statistically significant positive effect; odds ratio=2.36(95% CI 1.41 to 3.94) for Army and a statistically significant positive effect; odds ratio=1.54 (95% CI 1.16 to 2.04) for not Army. There was a very high degree of heterogeneity of effects among studies in the subgroup of Army (τ^2^=0.31, I^2^=94%) and no heterogeneity in the sub group of not Army (τ^2^=0.00, I^2^=0%). The confidence intervals of the subgroups overlapped. There was no evidence to support the hypothesis that the effect at other time points differs by branch of service (Army versus other).

###### 4.3.4.1.2 *Depression*

It was not possible to perform sub group analyses for Reserves/Guards/Regulars for any time points for the absolute comparison. For the absolute comparison it only possible to perform Army sub group analyses for long term and other time points.

**Figure 25:**
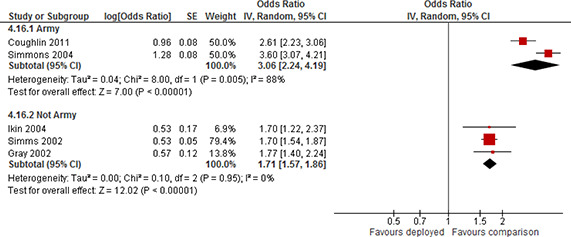
Forest plot, subgroup Army and Not Army, Depression, deployment versus non‐deployment, more than 24 months post deployment, odds ratio

Of the five studies providing effect estimates for long term outcomes, two studies used a sample where all participants were Army personnel. The forest plot for the five effect estimates is displayed in Figure 25. Pooled results for the two subgroups showed a statistically significant positive effect; odds ratio=3.06 (95% CI 2.24 to 4.19) for Army and a statistically significant positive effect; odds ratio=1.71 (95% CI 1.57 to 1.86) for not Army. There was a high degree of heterogeneity of effects among studies in the subgroup of Army (τ^2^=0.04, I^2^=88%) and no heterogeneity in the sub group of not Army (τ^2^=0.00, I^2^=0%). The confidence intervals of the subgroups did not overlap. The available evidence suggested that the long term effect may differ by branch of service in the sense that deployed Army personnel are worse off than deployed military personnel from other branches. Notice however, that the result relied strictly on variation *between* studies, not within; and only two of the three studies were included in the sub groups. One should be careful when interpreting results that rely on variation between studies and especially when very few studies are available.

**Figure 26:**
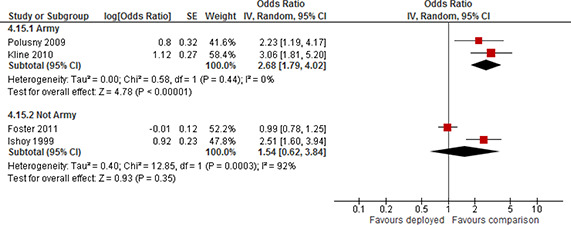
Forest plot, subgroup Army and Not Army, Depression, deployment versus non‐deployment, variable number of months post deployment, odds ratio

Two studies used a sample where all participants were Army personnel. The forest plot for the four effect estimates is displayed in Figure 26. Pooled results showed a statistically significant positive effect; odds ratio=2.68 (95% CI 1.79 to 4.02) for Army and a statistically non‐significant effect; odds ratio=1.54 (95% CI 0.62 to 3.84) for not Army. There was no heterogeneity of effects among studies in the subgroup of Army (τ^2^=0.00, I^2^=0%) and a high degree of heterogeneity in the sub group of not Army (τ^2^=0.40, I^2^=92%). The confidence intervals of the subgroups overlapped. There was no evidence to support the hypothesis that the effect at other time points differs by branch of service (Army versus other).

###### 4.3.4.1.3 *Substance use*

It was only possible to perform sub group analyses for other time points.

Of the five studies providing effect estimates for other time points, one study reported results separated by Reserves/Guards and Regulars and two studies used a sample of Reserves/Guards only.

**Figure 27:**
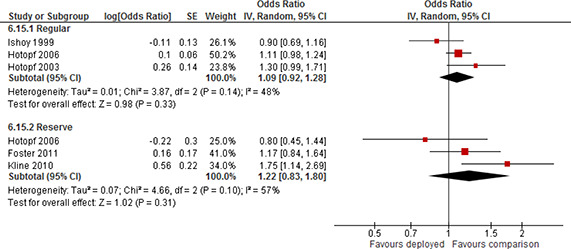
Forest plot, subgroup Reserve/Guard and Regular, Substance use, deployment versus non‐deployment, variable number of months post deployment, odds ratio

The forest plot for the six effect estimates is displayed in Figure 27. Pooled results for the two subgroups showed a statistically non‐significant effect; odds ratio=1.09 (95% CI 0.92 to 1.28) for Regulars and a statistically non‐significant effect; odds ratio=1.22 (95% CI 0.83 to 1.80) for Reserves/Guards. There was a low degree of heterogeneity of effects among studies in the subgroup of Regulars (τ^2^=0.01, I^2^=48%) and a moderate degree of heterogeneity for Reserves/Guards (τ^2^=0.07, I^2^=57%). The confidence intervals of the subgroups overlapped. There was no evidence to support the hypothesis that the effect at other time points differs by duty/enlistment status (Reserves/Guards versus Regulars).

**Figure 28:**
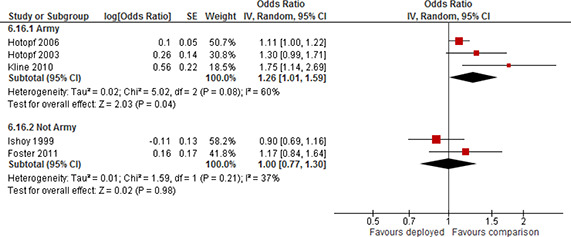
Forest plot, subgroup Army and Not Army, Substance use, deployment versus non‐deployment, variable number of months post deployment, odds ratio

Three studies used a sample where all participants were Army personnel. The forest plot for the five effect estimates is displayed in Figure 28. Pooled results showed a statistically significant positive effect; odds ratio=1.26 (95% CI 1.01 to 1.59) for Army and a statistically non‐significant effect; odds ratio=1.00 (95% CI 0.77 to 1.30) for not Army. There was a moderate degree of heterogeneity of effects among studies in the subgroup of Army (τ^2^=0.02, I^2^=60%) and a low degree of heterogeneity in the sub group of not Army (τ^2^=0.01, I^2^=37%). The confidence intervals of the subgroups overlapped. There was no evidence to support the hypothesis that the effect at other time points differs by branch of service (Army versus other).

###### 4.3.4.1.4 *Common mental disorder*

It was not possible to perform sub group analyses for Army at any time points, and it was only possible to perform Reserve/Regular sub group analysis for long term outcomes.

Of the five studies providing effect estimates for long term outcomes, two studies used a sample of Reserves/Guards only.

**Figure 29:**
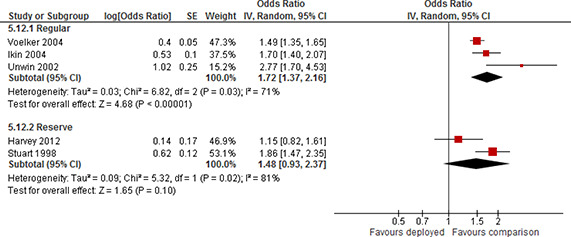
Forest plot, subgroup Reserve/Guard and Regular, Common mental disorder, deployment versus non‐deployment, more than 24 months post deployment, odds ratio

The forest plot for the five effect estimates is displayed in Figure 29. Pooled results for the two subgroups showed a statistically significant positive effect; odds ratio=1.72(95% CI 1.37 to 2.16) for Regulars and a statistically non‐significant effect; odds ratio=1.48 (95% CI 0.93 to 2.37) for Reserves/Guards. There was a moderate degree of heterogeneity of effects among studies in the subgroup of Regulars (τ^2^=0.03, I^2^=71%) and a high degree of heterogeneity for Reserves/Guards (τ^2^=0.09, I^2^=81%). The confidence intervals of the subgroups overlapped. There was no evidence to support the hypothesis that the long term effect differs by duty/enlistment status (Reserves/Guards versus Regulars).

#### 4.3.5 Relative Comparison: Deployed participants stratified by combat exposure

##### 4.3.5.1.1 *Post‐traumatic stress disorder*

It was not possible to perform sub group analyses for Reserves/Guards and Regulars for any time points. It was only possible to perform Army sub group analyses for short term outcomes.

Of the nine studies providing effect estimates for short term outcomes, four studies used a sample of Army only and one study used a sample of 84% Army personnel.

**Figure 30:**
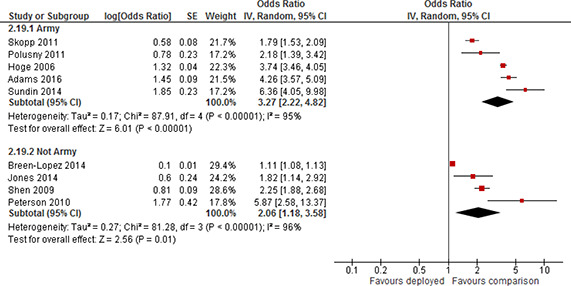
Forest plot, subgroup Army and Not Army, PTSD, high combat exposure versus low combat exposure, 0‐6 months post deployment, odds ratio

The forest plot for the nine effect estimates is displayed in Figure 30. Pooled results for the two subgroups showed a statistically significant positive effect; odds ratio=3.27(95% CI 2.22 to 4.82) for Army and a statistically significant positive effect; odds ratio=2.06 (95% CI 1.18 to 3.58) for not Army. There was a very high degree of heterogeneity of effects among studies in the subgroup of Army (τ^2^=0.17, I^2^=95%) and a very high degree of heterogeneity for not Army (τ^2^=0.27, I^2^=96%). The confidence intervals of the subgroups overlapped. There was no evidence to support the hypothesis that the short term effect differs by branch of service (Army versus other).

##### 4.3.5.1.2 *Depression*

It was not possible to perform any sub group analyses for depression at any time point.

##### 4.3.5.1.3 *Substance use*

It was not possible to perform any sub group analyses for substance use at any time point.

##### 4.3.5.1.4 *Common mental disorder*

It was not possible to perform any sub group analyses for common mental disorder at any time point.

#### 4.3.6 Sensitivity analysis

Sensitivity analyses were planned to evaluate whether the pooled effect sizes were robust across study design and components of methodological quality. Due to the fact that we found no randomised controlled trials, we could not evaluate the impact of study design. For methodological quality, we carried out sensitivity analyses for the confounding, incomplete data, and other risk of bias components of the risk of bias checklists, respectively. Two sets of sensitivity analyses were performed. First, we examined the robustness of conclusions when we included studies that were excluded due to a score of 5 (too high risk) on the confounding item. Ninety five studies were excluded from synthesis following risk of bias assessment. All of these studies were excluded due to a score of 5 (too high risk) on the confounding item for non‐randomised studies. We were able to extract effect sizes without participant overlap from 26 of the 95 studies. In the sensitivity analysis we pooled these studies with the studies from the synthesis sample. The analyses are performed separately by comparison, outcome, and time since exposure, essentially replicating the meta‐analyses conducted in 4.3.1 and 4.3.2.

Secondly, we examined the robustness of our conclusions when we excluded studies with risk of bias scores of 4 on confounding, incomplete data, or other risk of bias.

##### 4.3.6.1 *Absolute comparison: Meta‐analyses on combined sample*

Table 5 presents the results of meta‐analyses on the combined sample of studies for the comparison between participants that were deployed and participants that were not deployed. Forest plots are displayed in Section 10.1. Note that for some outcomes there were no excluded studies due to a score of 5 (too high risk) on the confounding item for some of the time points. These outcomes and time points are therefore not presented in table 5.

**Table 5:** Sensitivity analysis. Inclusion of studies with a score of 5 (too high risk of bias). Comparison between deployed and not deployed. Separately by time since exposure. Odds ratio with 95% confidence interval (CI).

				**Mean**	**95% CI**	
**Outcome**	**Time**	**Studies with a score of 5 (too high risk)**	**k**	**OR**	**Lower**	**Upper**
PTSD	Long	Not included	8	3.31	2.69	4.07
		Included	10	2.88	2.21	3.75
	Other	Not included	6	1.91	1.28	2.85
		Included	12	1.78	1.43	2.21
Depression	Short	Not included	2	1.29	0.77	2.15
		Included	4	0.98	0.6	1.59
	Medium	Not included	1	1.05	0.83	1.33
		Included	4	1.29	0.91	1.82
	Long	Not included	5	2.19	1.58	3.03
		Included	7	1.91	1.39	2.63
	Other	Not included	4	1.98	1.05	3.7
		Included	9	1.51	1.19	1.92
Substance use	Short	Not included	1	1.15	0.61	2.15
		Included	2	1.18	0.92	1.52
	Other	Not included	5	1.15	0.98	1.36
		Included	9	1.16	1.04	1.29
Common mental disorder	Long	Not included	5	1,64	1,38	1,96
		Included	6	1,55	1,28	1,88
	Other	Not included	2	1,27	0,83	1,96
		Included	4	1,31	0,98	1,74

Notes: k = number of studies.

There were no appreciable changes in the results following the inclusion of studies that were excluded due to a score of 5 (too high risk) on the confounding item for any outcome measure at any time point.

The results of excluding studies with a score of 4 on the confounding, incomplete data, or other risk of bias items are provided in Table 6 and displayed in forest plots in Section 10.1.

**Table 6:** Sensitivity analysis. Exclusion of studies with a score of 4 on the confounding, incomplete data, or other risk of bias items. Comparison between deployed and not deployed. Separately by time since exposure. Odds ratio with 95% confidence interval (CI).

				**Mean**	**95% CI**	
**Outcome**	**Time**	**Studies excluded**	**k**	**OR**	**Lower**	**Upper**
PTSD		Long	8	3.31	2.69	4.07
		Confounding score of 4	2	3.19	1.77	5.74
		Incomplete data score of 4	7	3.57	2.99	4.28
		Other score of 4	7	3.37	2.71	4.2
	Other		6	1.91	1.28	2.85
		Confounding score of 4	3	1.53	1.06	2.22
Depression		Other score of 4	5	2.03	1.25	3.31
	Long		5	2.19	1.58	3.03
		Incomplete data score of 4	4	1.93	1.51	2.46
	Other		4	1.98	1.05	3.7
		Other score of 4	3	2.6	1.93	3.52
Substance use	Other		5	1.15	0.98	1.36
		Confounding score of 4	2	1.13	1.01	1.28
		Other score of 4	4	1.16	0.95	1.41
Common mental disorder	Long		5	1.64	1.38	1.96
		Incomplete data score of 4	4	1.59	1.29	1.97
		Other score of 4	4	1.74	1.45	2.08

Notes: k = number of studies.

There were no appreciable changes in the results following removal of studies with a score of 4 on the confounding, incomplete data, or other bias components of the risk of bias checklists.

In summary, the conclusions of the main syntheses do not change.

##### 4.3.6.2 *Relative comparison: Meta‐analyses on combined sample*

Table 7 presents the results of meta‐analyses on the combined sample of studies for the comparison between deployed with high combat exposure and deployed with low combat exposure. Forest plots are displayed in Section 10.1 Note that for some outcomes there were no excluded studies due to a score of 5 (too high risk) on the confounding item for some of the time points. These outcomes and time points are therefore not presented in Table 7.

**Table 7:** Sensitivity analysis. Inclusion of studies with a score of 5 (too high risk of bias). Comparison between deployed stratified by combat exposure. Separately by time since exposure. Odds ratio with 95% confidence interval (CI).

				**Mean**	**95% CI**	
**Outcome**	**Time**	**Studies with a score of 5 (too high risk)**	**k**	**OR**	**Lower**	**Upper**
PTSD	Short	Included	9	2.74	1.73	4.61
		Not included	13	2.49	2.03	3.06
	Long	Included	4	3.05	1.94	4.8
		Not included	5	3.03	2.08	4.41
	Other	Included	4	2.09	0.99	4.38
		Not included	5	1.41	1.21	1.63
Depression	Short	Included	2	2.00	1.79	2.23
		Not included	4	1.46	0.93	2.30
	Long	Included	3	1.81	1.28	2.56
		Not included	5	1.83	1.38	2.43
	Other	Included	1	3.86	2.18	6.81
		Not included	2	1.95	0.55	6.96
Substance use	Short	Included	2	1.63	1.01	2.62
		Not included	3	1.4	1.05	1.85
	Long	Included	1	1.6	0.85	3
		Not included	2	1.53	1.12	2.08
	Other	Included	2	1.2	1	1.46
		Not included	3	1.09	0.94	1.27
Common mental disorder	Short	Included	1	1	0,78	1,29
		Not included	2	1,09	0,81	1,47

Notes: k = number of studies.

There were no appreciable changes in the results following the inclusion of studies that were excluded due to a score of 5 (too high risk) on the confounding item for PTSD and common mental disorder at any time point. For depression there was no appreciable change in the result following the inclusion of studies that were excluded due to a score of 5 (too high risk) on the confounding item in the long term, but short term outcomes and outcomes measured at other time points lost statistical significance. However, the main analyses of depression (presented in Section 4.3.2) relied on one study each at these time points. For substance use the inclusion of studies that were excluded due to a score of 5 (too high risk) on the confounding item altered the statistically significance for all time points but the main analyses of substance use (presented in Section 4.3.2) relied on one or two studies each at any time point.

The results of excluding studies with a score of 4 on the confounding or incomplete data items^14^ are provided in Table 8 and displayed in forest plots in Section 10.1.

**Table 8:** Sensitivity analysis. Exclusion of studies with a score of 4 on the confounding or incomplete data items. Comparison between deployed stratified by combat exposure. Separately by time since exposure. Odds ratio with 95% confidence interval (CI).

				**Mean**	**95% CI**	
**Outcome**	**Time**	**Studies excluded**	**k**	**OR**	**Lower**	**Upper**
PTSD	Short		9	2.74	1.63	4.61
		Confounding score of 4	5	3.37	1.93	5.87
	Long		4	3.05	1.94	4.8
		Confounding score of 4	2	3.26	1.73	6.15
	Other		4	2.09	0.99	4.38
		Confounding score of 4	2	2.85	0.8	10.19
Depression	Long		3	1.81	1.28	2.56
		Incomplete data score of 4	2	2.11	1.73	2.56

Notes: k = number of studies.

There were no appreciable changes in the results following removal of studies with a score of 4 on the confounding or incomplete data components of the risk of bias checklists.

In summary, the conclusions of the main syntheses do not change.

#### 4.3.7 Publication bias

We assessed the possibility of publication bias visually by examining funnel plots. Only analyses with at least five studies included were examined. The six funnel plots are displayed in Section 10.2. There are too few studies and insufficient variation in the standard errors to assess whether the funnel plots are symmetric. However, there is no striking asymmetry visible in any of the funnel plots.

## 5 Discussion

### 5.1 SUMMARY OF MAIN RESULTS

This review focused on the impact on mental health and social functioning of deployment of military personnel to military operations. Two types of comparisons were investigated. The absolute impact was investigated using a comparison between a group of military personnel that deployed to a military operation and a group of military personnel that did not deploy. The other comparison was relative, because all military personnel were deployed, but differed in their exposure to combat.

The findings are mixed, depending on the outcome, the time since exposure and the approach (absolute or relative) used to investigate the effect. We did not synthesise the effect of deployment on the outcomes homelessness and employment. Three studies reported on these outcomes, but they were excluded from synthesis following risk of bias assessment. Thus, all studies that were synthesised reported on the impact of deployment on mental health; PTSD, depression, substance use or common mental disorder.

For assessments taken 0 to 6 months past exposure the evidence was inconclusive either because too few studies reported results in the short term and/or the degree of heterogeneity between studies was very large.

No conclusion could be made in the medium term (6‐24 months since exposure) as too few studies reported results at this time point.

For assessments taken at other time points (a variable number of months since exposure) the evidence was inconclusive for the relative comparisons due to either too few studies or a substantial degree of heterogeneity between studies. For the absolute comparison the analysis of common mental disorder was inconclusive, whereas the average effects of PTSD and depression were positive and statistically significant (PTSD odds ratio (OR) was 1.91 (95% confidence interval (CI): 1.28 to 2.85) and OR=1.98 (95% CI: 1.05 to 3.70) for depression);but there was a high degree of heterogeneity between the studies in both analyses. The analysis concerning substance use indicated that deployed did not havehigher odds of screening positive for substance use compared to non‐deployed participants (OR=1.15 (95% CI: 0.98 to 1.36)).

Overall, the results of mental health outcomes measured more than 24 months since exposure were more consistent than at any other time point. For assessments taken more than 24 months past exposure, meta‐analyses indicated that the odds of screening positive for PTSD, depression, substance use and common mental disorder were higher for participants in the deployed group compared to participants in the group that did not deploy (PTSD OR=3.31 (95% CI: 2.69 to 4.07), OR=2.19 (95% CI: 1.58 to 3.03) for depression, OR=1.27 (95% CI: 1.15 to 1.39) for substance use and OR=1.64 (95% CI: 1.38 to 1.96) for common mental disorder). Overall the results were consistent, except in the analysis of depression were there was a high degree of heterogeneity between the studies. Likewise, participants reporting high combat exposure had higher odds of screening positive for PTSD and depression than participants reporting lower exposure for long term assessments (PTSD OR=3.05 (95% CI: 1.94 to 4.80) and OR=1.81 (95% CI: 1.28 to 2.56) for depression) and there was a low degree of heterogeneity between studies. The analyses of substance use and common mental disorder were inconclusive due to too few studies.

To understand what our findings mean for the mental health in post deployed populations we can rely on evidence about the prevalence of mental health problems in pre‐deployed or non‐deployed population based comparison samples. Such evidence should be informative about the likely extent of the issue. Sundin, Fear, Iversen, Rona, and Wessely (2010) reviewed the prevalence of PTSD in population‐based studies among personnel deployed to Iraq. They report PTSD prevalence in pre‐deployed comparison military personnel samples in the range of 2% to 5.6% (summary estimate 3.83%). Using this range and the estimate for the effect of deployment on PTSD in the long term from our meta‐analyses, we would expect the prevalence of PTSD in post‐deployed samples to be in the range 6.1 – 14.9%^15^. Blore, Sim, Forbes, Creamer, & Kelsall (2015) similarly review prevalence estimates for major depression in Gulf War veterans. When we restrict attention to studies using population‐based samples, prevalence measures are in the range 3.9% to 11.3%. For this range the estimate for the effect of deployment on depression in the long termfrom our meta‐analyses implies that in post‐deployed samples the prevalence of depression range from 7.6% to 18%. Kelsall et al. (2015) review studies of alcohol and/or substance use for both the Gulf War and for Iraq and Afghanistan (OEF/OIF). For population‐based studies they report prevalence in the range 2% to 13.1% for the Gulf War and 2.2% to 21.1% for OEF/OIF. Using this range of estimates we find that prevalence in the long term in post‐deployed samples is in the range 2.4% to 17.5%. Stimpson et al. (2003) review the Gulf War evidence for common mental disorder and report prevalence estimates in the range 7.3% to 24% in pre‐deployed comparison samples. For this outcome the long term odds ratio estimate implies prevalence in the post‐deployed samples ranging between 10% and 23%.

It was possible to assess the impact of two types of personnel characteristics (branch of service and duty/enlistment status) on the mental health outcomes. Where possible, subgroup analyses were performed using effect estimates separated by Army versus other branches of service and Reserves/Guards versus Regulars. We only performed subgroup analysis if there were at least two studies in each subgroup. We found no evidence to suggest that the effect of deploymenton any outcomes differed between these two types of personnel characteristics.

### 5.2 OVERALL COMPLETENESS AND APPLICABILITY OF EVIDENCE

In this review we included 40 studies in the data synthesis. This number is relatively low compared to the large number of studies (185) meeting the inclusion criteria. The reduction was caused by three different factors. Forty eight of the 185 studies did not report effect estimates or provide data that would allow the calculation of an effect size and standard error. Fifty four studies were excluded because of overlapping samples. Forty three studies were judged to have a very high risk of bias (5 on the scale) and, in accordance with the protocol, we excluded these from the data synthesis on the basis that they would be more likely to mislead than inform^16^.

More useable studies would have added additional weight and evidence to the synthesis and would have provided a more robust literature on which to base conclusions.

The 40 studies used in the data synthesis covered the US, UK, Australia, Denmark and Germany (5 countries), whereas 13 countries were represented by the 185 studies.

It was not possible to examine the impact on deployment of mental health history, gender, age, ethnicity and military rank. It was possible to study the impact of two types of personnel characteristic (branch of service and duty/enlistment status).

We could not synthesise the effect of deployment on the secondary outcomes homelessness and employment. Three studies reported on these outcomes, but they were excluded from synthesis following the risk of bias assessment.

All studies used military samples of participants, but there was substantial variation in sampling methods, ranging from full population cohorts to random representative sampling to convenience samples. Mental health outcomes were measured in many different ways, overwhelmingly with screening tools, where possible caseness was constructed in the studies, frequently by using a validated threshold, or for example by designating participants scoring in the upper quartile of the distribution of answers as cases. Even when studies used validated thresholds there was substantial variation in the thresholds applied. This reflects the different objectives of primary studies, but results should be interpreted in this light. We found no strong indication of publication bias.

### 5.3 QUALITY OF THE EVIDENCE

All included studies used a non‐randomised study design with a control group drawn from a military population. Studies either used statistical controls, pre‐processing statistical matching, or longitudinal variation, to identify the consequences of deployment on mental health and social functioning.

Overall the risk of bias in the majority of included studies was high. The risk of bias was examined using a tool for assessing risk of bias incorporating non‐randomised studies. We attempted to enhance the quality of the evidence in this review by excluding studies judged to be at very high risk of bias using this tool. We believe this process excluded those studies that are more likely to mislead than inform. The main synthesis was conducted on 40 studies. Ninety five studies were judged to be at very high risk of bias. In all95 studies the concern of too high risk of bias was on the confounding item. Most of these studies failed to establish a comparison group that was balanced on important confounders. Many of the studies did not have impact measurement of deployment as a primary research question. They may use an adequate research design to answer their primary research question, but the comparison of interest to this review may not be identified without substantial bias. One such example was the interest in co‐morbidity: for example the question whether PTSD and depression tend to be present at the same time (Breslau, Davis, Peterson, & Schultz, 2000). For example investigators addressed this question by controlling for depression, post‐deployment, as an additional variable in a regression with PTSD as the dependent variable. The effect estimate can then be interpreted as a measure of the risk of PTSD conditional on depression symptoms. While this type of modellingraises fundamental issues, for example with respect to reverse causality, the specification does not identify the same effect as a specification that is not conditional on depression symptoms. Additionally some studies also controlled for outcomes that occur post‐deployment. This may bias results if deployment has a direct effect on these variables such for example marriage or employment. We note that primary studies may have another parameter of interest in mind, but they do not identify the parameter of interest to this review.

Furthermore, we performed a number of sensitivity analyses to check whether the obtained results are robust across methodological quality. Two sets of sensitivity analyses were performed. First, we examined the robustness of conclusions when we included studies that were excluded due to a score of 5 (too high risk) on the confounding item. Second, we examined the robustness of our conclusions when we excluded studies with risk of bias scores of 4 on confounding, incomplete data, or other risk of bias. The overall conclusions did not change on the basis of these analyses.

There was overall consistency in the direction of effects; deployment to military operations adversely affects the mental health functioning of military personnel compared to non‐deployed personnel or personnel deployed with low or no combat exposure.

### 5.4 LIMITATIONS AND POTENTIAL BIASES IN THE REVIEW PROCESS

We performed a comprehensive electronic database search, combined with grey literature searching, and hand searching of key journals. No studies are awaiting classification, nor are we aware of any on‐going studies. All citations were screened by two independent screeners, and review authors assessed all included studies against inclusion criteria.

We believe that there are no other potential biases in the review process as two members of the review team^17^ independently coded the included studies. Any disagreements were resolved by discussion. Further, decisions about inclusion of studies and assessment of study quality were made by two review authors independently and minor disagreements resolved by discussion. Numeric data extraction was made by one review author and was checked by a second review author and member of the review team.

### 5.5 AGREEMENTS AND DISAGREEMENTS WITH OTHER STUDIES OR REVIEWS

Stimpson et al. (2003) conducted a systematic review and meta‐analysis of PTSD, common mental disorders (depression and anxiety), and alcohol misuse in Gulf War veterans. Similar to this review, they compared deployed military personnel to non‐deployed personnel, or personnel deployed elsewhere. Their review included 20 studies, and like this review excluded a number of studies because they repeated results already included. Their summary estimates (odds ratios) were: 3.16 (95% CI: 2.14 to 4.65) for PTSD, 2.04 (95% CI: 1.94 to 2.15) for common mental disorder, and found only limited evidence for alcohol misuse. In terms of summary estimates for PTSD and common mental disorders the findings of this review is in line with that evidence, even if this review also included more recent evidence than that from the Gulf War.

Magruder and Yeager (2009) conducted a systematic review and meta‐analysis on the effect of deployment on PTSD for U.S. service personnel serving in the Iraq War (OIF/OEF), the Gulf War, and for the Vietnam War. Synthesis was conducted by war era. They found only two studies for OIF/OEF with a summary odds ratio for PTSD of 1.42 (95% CI: 1.31 to 1.53) for deployed personnel compared to non‐deployed personnel. Since the publication of their review a substantial number of research studies have been published on personnel deployed to OIF/OEF as reflected by the number of included studies in our review. Magruder and Yeager (2009) included 12 studies in the synthesis for the Gulf War, with a summary odds ratio for PTSD of 2.74 (95% CI: 2.47 to 3.03), echoing the finding of this review.

Blore, Sim, Forbes, Creamer, &Kelsall (2015) conducted a systematic review and meta‐analysis of depression in Gulf War veterans. The review included 14 studies. The summary odds ratio for depression in Gulf War veterans was 2.28 (95% CI: 1.88 to 2.76) compared to non‐deployed military personnel. We found a similar summary odds ratio for depression in the long term

Kellsall et al. (2015) conducted a systematic review and meta‐analysis of the effect of deployment on alcohol use and substance use disorders of military personnel compared to non‐deployed personnel. In total 18 studies were included in their review. They found a summary odds ratio of 1.33 (95% CI: 1.22 to 1.46) for alcohol and 2.13 (95% CI: 0.96 to 4.72) for substance use among Gulf War veterans compared to non‐deployed personnel. For Iraq/Afghanistan veterans they found a summary odds ratio of 1.36 (95% CI: 1.11 to 1.66) for alcohol and 1.14 (95% CI: 1.04 to 1.25) for substance. In the meta‐analyses conducted in this review we pooled alcohol and substance use. The results are therefore not directly comparable but still of same order of magnitude in the long term.

## 6 Authors’ conclusions

### 6.1 IMPLICATIONS FOR PRACTICE AND POLICY

Deployment to military operations may adversely affect the mental health functioning of military personnel compared to non‐deployed personnel or personnel deployed with low or no combat exposure. This review synthesised effects of deployment on PTSD (post‐traumatic stress disorder), depression, substance use, and common mental disorder (defined as depression or anxiety).

It is important for practitioners and policymakers to understand the extent of mental health conditions in post‐deployed samples. This can inform on‐going policy development in addressing mental health issues in post deployed personnel (for example, Obama, 2010). We found substantial adverse effects of deployment on PTSD in the long run (more than 24 months since exposure), more moderate effects of deployment on depression and common mental disorder in the long run and only a small effect on substance use in the long term.

For assessments taken less than 24 months or a variable number of months since exposure the evidence was less consistent and in many instances inconclusive either because too few studies reported results and/or the degree of heterogeneity between studies was very large.

Studies varied widely in terms of instruments used and in the types of thresholds applied. This may partly explain the observed heterogeneity between studies and may reflect the different purpose of studies. For example, if detecting all cases is important, studies may use a more sensitive, but less specific screening tool. The detection of possible PTSD is a case in point. Few studies used clinical assessment; indeed most studies used either the PCL or the PC‐PTSD screening tools for detecting PTSD. Bliese, Wright, Adler, Cabrera, Castro, and Hoge (2008) validated the PCL and the PC‐PTSD screen in a sample of active duty military personnel three months after their return from a yearlong deployment to Iraq. They found that a cut‐off in the range 30 to 34 on the PCL and recommended a cut‐off of either 2 or 3 on the PC‐PTSD. Yet many studies in our sample used a threshold of 50 on the PCL. This threshold was validated by Weathers et al. (1993) on a sample of treatment seeking Vietnam War veterans. Other studies used a more stringent combined criterion requiring both a cut‐off of 50 on the PCL and endorsement of functional impairment. While the two criteria have been validated separately in military samples, they have not been validated jointly. A stringent criterion is likely to miss sub threshold cases (“hidden cases”) that likely benefit from early interventions before their mental condition develops into a full‐blown case. From a preventive perspective it might be desirable to apply lower thresholds to identify at‐risk personnel.

Psychological disorders are common, disabling, and burdensome in military populations. As such they constitute an important cost of war. It is worth emphasizing that this review has established that while PTSD is the signature mental health problem in deployed military populations, a range of other mental health problems follow in its wake. To identify and treat these issues requires screening tools sufficiently broad to be able to detect the multifaceted set of risks that deployed personnel are exposed to, and screening tool thresholds must be adjusted to the purpose of the screening. Mental illness is of particular concern in the military for operational reasons, but they may be hard to detect as military careers are intimately linked with mental and physical strength. The literature has suggested that stigma reduction, and reducing barriers to careare important implications of such findings. While these are certainly important improvements, breaking the fundamental link between career opportunities and mental fitness may be just as important. This could for example imply creating and actively promoting alternative career paths in the military.

The risk of mental health problems in the domains examined here was consistently high in the longer term. An implication is that veteran populations are likely to be characterized by high prevalence even long after their separation from the military. This is an important implication for practitioners working in primary care. It also suggests a double dividend of early detection. Screening tools that identify not only cases, but also personnel at‐risk, is likely to be beneficial both for affected personnel, as early treatment may be possible and the failure to detect mental health problems early may also lead to a substantial increase in future treatment costs.

It appears that the odds of experiencing mental health problems is about the same when we compare a group that were deployed to a non‐deployed comparison group and when we compare a group that were deployed with high intensity combat exposure to a deployed group with low intensity or no exposure. This is perhaps surprising, and is consistent with combat exposure being the main stressor in terms of increased risk for mental health problems. These additional risk factors for mental health problems can potentially be incorporated into screening protocols.

We were only able to examine a subset of moderators of the effect of deployment on mental health functioning; Army versus other branches and reservists versus regulars.

Based on the low number of studies we were only able to perform subgroup analyses and not for all outcome/time since exposure combinations. We have drawn no overall conclusion because the analysis is based on a subset of the meta‐analyses. It is an important shortcoming of the current evidence that such moderators of effects have not yet been fully investigated.

We were particularly interested in examining the potential role of duration of deployment and “downtime”^18^ (time between deployments) for mental health functioning. Given the present context where active duty personnel are likely to see several tours during their active career this is particularly important. From a policy perspective because these are direct parameters that one could use to optimally “organize” deployment in order to minimize impact on mental health functioning. Unfortunately very few studies reported on these measures for the comparisons relevant to this review and we were unable to examine this further. Indeed because the evidence strongly supports the notion that deployment affects mental health functioning, the next step is to begin to examine preventive measures, which may include policies for organizing deployment in order to minimize impact on mental health functioning.

The review aimed to examine effects on civilian life as well, in particular, the effect of deployment on subsequent employment and homelessness. Unfortunately there was insufficient evidence available and consequently this could not be examined.

### 6.2 IMPLICATIONS FOR RESEARCH

All studies included in this review used a non‐randomised study design. The complete lack of evidence of a quasi‐randomised nature is a concern. While it is difficult to imagine a randomised study design to understand how deployment affects mental health of military personnel, for example policy changes to personnel policy, or unanticipated shocks to the demand for military personnel could potentially be a rich source of quasi‐experimental variation.

The strongest non‐randomised study designs were those that used longitudinal designs, in particular studies that were able to statistically control for mental health status prior to deployment. Indeed a near ideal design would match treatment and control groups on some measure of prior mental health status, since prior mental health is likely to be the strongest predictor of current mental health. Furthermore current mental health is an important characteristic for the deployment decision, which makes it an important balancing variable for observational study designs. In the absence of randomised or quasi‐randomised evidence it is important that studies attempt to establish balance between the deployed group and the comparison. Lyk‐Jensen, Weatherall, and Jepsen (2016) use administrative panel data on Danish military personnel to assess the effect of deployment on mental health up to nine years after deployment. They apply a difference‐in‐difference study design with a matched control group, and controlling for mental health prior to deployment. The design allows them to circumvent many potential biases including attrition bias. Observational study designs that mimic randomization give greater credibility to the evidence. For instance studies may use change in deployment policies in a difference‐in‐difference framework, or contractual constraints to try and identify effects. Another potential approach would be to have a greater focus on decision making procedures of military commanders. Both physical and mental health and resilience are important factors when commanders decide whether and where to deploy personnel. It may be possible to look at a subset of the relevant population namely those that are on the margin between either being deployed/not deployed, and the type of deployment. Such data have been used in other research contexts to identify effects because it is more credible to argue that assignment is “as good as random” for individuals who are on the margin.

We found substantial between study heterogeneity in some meta‐analyses. We were not able to explain this heterogeneity with study characteristics. Overall, this indicates that we do not yet have sufficient knowledge about moderators of effect sizes. Therefore more studies are needed in order to examine moderators of effect sizes further.

The military samples included in this review consisted both of population based samples and convenience samples. A drawback of convenience samples is that they may not be representative of the population of military personnel. For example anecdotal evidence of particularly adverse effects on mental health for a particular brigade may prompt a study. While this study may demonstrate a true relationship between deployment and adverse mental health effects, the strength of the relationship is likely to be exaggerated because of the manner in which the sample was recruited. In order to better inform practice and policy about the effect of deployment it is desirable that investigators use representative sampling of participants. On the other hand studies that used population based samples typically did not report the time since exposure. This may be related to the type of sampling where personnel who may have been deployed at different time points and to different military operations are sampled to reflect the current composition of personnel. Because this review demonstrated that veteran populations are likely characterized by high prevalence even long after their separation from the military, studies that use population based samples should either control for time since exposure in their analysis or report the mean time since exposure.

A related point is selective attrition in samples. Selective attrition is present if for example deployed personnel with mental health problems are more likely to separate from service than personnel in the comparison group. That is, there is a correlation between attrition and deployment status. Similarly many studies rely on self‐report, or recruit participants via telephone, which may introduce bias. Selective attrition of this type could challenge the internal validity of a study. Investigators should demonstrate that selective attrition is not likely to influence results. This can for example be demonstrated by actively recruiting separated service members, and possibly by increasing follow up efforts of personnel that have separated, or by controlling for the possible correlation between separation from service and deployment status in the analyses. Another way to overcome this issue is to rely on administrative dataset, making it possible to systematically follow up personnel who have separated from the military. This will also help in assessing the external validity of findings, for example if a similar relationship can be demonstrated in samples of personnel that have separated from the military.

The studies included in this review frequently used different instruments to assess the same outcome and even when studies used the same instrument the cut‐off used varied. Not all studies used validated cut‐offs. For example defining a case as the combination of at least 50 on the PCL and endorsement of functional impairment has been validated separately but not jointly. Studies varied in the type of research questions they posed, which may have motivated the use different cut‐off values. We were unable to investigate such relations further due to the small number of studies. Regardless, it will be beneficial if studies also report effects using validated commonly used cut‐off values, such that it is easier to compare studies.

## 8 Information about this review

### 8.1 ACKNOWLEDGEMENTS

The review authors would like to thank both the Campbell Education Group, Social Welfare Group, and Methods Group for their assistance with this review.

Thanks to former Head of SFI Campbell, Ph. D. Mette Deding, for continued support and efforts to realise this review. Without the joint efforts of the entire review team, not just the review authors, this review would not be possible.

### 8.2 REVIEW AUTHORS

**Lead review author:**

**Table:** 

**Name:**	**Martin Bøg**
Title:	PhD
Country:	Denmark
Email:	martin.bog@gmail.com
**Co‐authors:**	
**Name:**	**Trine Filges**
Title:	PhD
Affiliation:	VIVE Campbell
Address:	Herluf Trollesgade 11
Postal Code:	DK‐1052 Copenhagen K
Country:	Denmark
Email:	tif@vive.dk
	
**Name:**	**Anne‐Marie Klint Jørgensen**
Title:	Information specialist
Country:	Denmark
	

### 8.3 ROLES AND RESPONSIBILITIES

- Content: Martin Bøg, Trine Filges
- Systematic review methods: Trine Filges, Martin Bøg
- Statistical analysis: Trine Filges, Martin Bøg, Tróndur Møller Sandoy
- Information retrieval: Anne‐Marie Klint Jørgensen, Pia Vang Hansenand Bjørn Christian Viinholt Nielsen
- Coding:
  ∘ Descriptive: Tróndur Møller Sandoy, Sjúrdur Zachariasson, Rasmus Henriksen Klokker, Ulrik Højmark Pedersen
  ∘ Numerical: Martin Bøg, Trine Filges, Tróndur Møller Sandoy, Sjúrdur Zachariasson
  ∘ Risk of Bias: Martin Bøg, Trine Filges
- Screening: Martin Bøg, Trine Filges, Tróndur Møller Sandoy, Sjúrdur Zachariasson, Rasmus Henriksen Klokker, Julie Marie Nielsen, Freja Jørgensen, Ulrik Højmark Pedersen, Ida Scheel Rasmussen, Pia Vang Hansen, Therese Lucia Friis, Marcel Mirzaei‐Fard, Asta Breinholt Lund, Stine Lian Olsen, Anne‐Sofie Due Knudsen and Bjørn Christian Viinholt Nielsen

### 8.4 SOURCES OF SUPPORT

SFI (VIVE) Campbell.

### 8.5 DECLARATIONS OF INTEREST

The authors have no vested interest in the outcomes of this review, nor any incentive to represent findings in a biased manner.

### 8.6 PLANS FOR UPDATING THE REVIEW

Martin Bøg will be responsible for updating the review, as funding becomes available.

### 8.7 DEVIATIONS FROM THE PROTOCOL

In the protocol we stated that we would contact international experts to identify unpublished and on‐ going studies, and provide them with the inclusion criteria for the review along with the list of included studies, asking for any other published, unpublished or ongoing studies relevant for the review. We only consulted with our in‐house content expert.

We stated in the protocol that when there was insufficient numerical data in a report to calculate the type of effect size used for synthesis we would contact investigators requesting the necessary data. In the cases where this occurred we did not contact investigators, as this would have required that they perform additional statistical analyses to answer our enquiry.

### 8.8 AUTHOR DECLARATION

**Authors’ responsibilities**

By completing this form, you accept responsibility for maintaining the review in light of new evidence, comments and criticisms, and other developments, and updating the review at least once every five years, or, if requested, transferring responsibility for maintaining the review to others as agreed with the Coordinating Group. If an update is not submitted according to agreed plans, or if we are unable to contact you for an extended period, the relevant Coordinating Group has the right to propose the update to alternative authors.

**Publication in the Campbell Library**

The Campbell Collaboration places no restrictions on publication of the findings of a Campbell systematic review in a more abbreviated form as a journal article either before or after the publication of the monograph version in *Campbell Systematic Reviews*. Some journals, however, have restrictions that preclude publication of findings that have been, or will be, reported elsewhere, and authors considering publication in such a journal should be aware of possible conflict with publication of the monograph version in *Campbell Systematic Reviews*. Publication in a journal after publication or in press status in *Campbell Systematic Reviews* should acknowledge the Campbell version and include a citation to it. Note that systematic reviews published in *Campbell Systematic Reviews* and co‐registered with the Cochrane Collaboration may have additional requirements or restrictions for co‐publication. Review authors accept responsibility for meeting any co‐publication requirements.

**I understand the commitment required to update a Campbell review, and agree to publish in the Campbell Library. Signed on behalf of the authors:**

**Form completed by: Martin Bøg**

**Date: 29 December 2015**

## 9 Tables

### 9.1 ASSESSMENT OF RISK OF BIAS IN INCLUDED STUDIES

#### 9.1.1 Risk of bias table

**Table:** 

**Item**	**Judgement** ^a^	**Description** (quote from paper, or describekey information)
1. Sequence generation		
2. Allocation concealment		
3. Confounding^b^ ^,^ ^c^		
4. Blinding?^b^		
5. Incomplete outcome data addressed?^b^		
6. Free of selective reporting?^b^		
7. Free of other bias?		
*8. A priori* protocol?^d^		
*9. A priori* analysis plan?^e^		

a Some items on low/high risk/unclear scale (double‐line border), some on 5 point scale/unclear (single line border), some on yes/no/unclear scale (dashed border). For all items, record “unclear” if inadequate reporting prevents a judgement being made.

b For each outcome in the study.

c This item is only used for QESs. It is based on list of confounders considered important at the outset and defined in the protocol for the review (*assessment against worksheet*).

d Did the researchers write a protocol defining the study population, intervention and comparator, primary and other outcomes, data collection methods, etc. in advance of starting the study?

e Did the researchers have an analysis plan defining the primary and other outcomes, statistical methods, subgroup analyses, etc. in advance of starting the study?

#### 9.1.2 Risk of bias tool

##### 9.1.2.1 *Studies for which RoB tool is intended*

The risk of bias model is developed by Prof. Barnaby Reeves in association with the Cochrane Non‐Randomised Studies Methods Group.^19^ This model, an extension of the Cochrane Collaboration's risk of bias tool, covers both risk of bias in randomised controlled trials (RCTs and QRCTs), but also risk of bias in non‐randomised studies (QES).

The point of departure for the risk of bias model is the Cochrane Handbook for Systematic Reviews of interventions (Higgins & Green, 2008). The existing Cochrane risk of bias tool needs elaboration when assessing non‐randomised studies because, for non‐randomised studies, particular attention should be paid to selection bias / risk of confounding. Additional item on confounding is used only for non‐randomised studies (QESs) and is not used for randomised controlled trials (RCTs and QRCTs).

##### 9.1.2.2 *Assessment of risk of bias*

Issues when using modified RoB tool to assess included non‐randomised studies:

- Use existing principle: score judgment and provide information (preferably direct quote) to support judgment
- QESs.
- 5‐point scale for some items (distinguish “unclear” from intermediate risk of bias).
- Keep in mind the general philosophy – assessment is not about whether researchers could have done better but about risk of bias; the assessment tool must be used in a standard way whatever the difficulty / circumstances of investigating the research question of interest and whatever the study design used.
- Anchors: “1/No/low risk” of bias should correspond to a high quality RCT. “5/high risk” of bias should correspond to a risk of bias that means the findings should not be considered (too risky, too much bias, more likely to mislead than inform)

1. Sequence generation
  - Low/high/unclear RoB item
  - Always high RoB (not random) for a non‐randomised study
  - Might argue that this item redundant for QES since always high – but important to include in RoB table (‘level playing field’ argument)
2. Allocation concealment
  - Low/high/unclear RoB item
  - Potentially low RoB for a non‐randomised study, e.g. quasi‐randomised (so high RoB to sequence generation) but concealed (reviewer judges that the people making decisions about including participants didn't know how allocation was being done, e.g. odd/even date of birth/hospital number)
3. RoB from confounding (additional item for QES; assess for each outcome)
  - Assumes a pre‐specified list of potential confounders defined in the protocol
  - Low(1) / 2 / 3 / 4 / high(5) / unclear RoB item
  - Judgment needs to factor in:
    ∘ proportion of confounders (from pre‐specified list) that were considered
    ∘ whether most important confounders (from pre‐specified list) were considered
    ∘ resolution/precision with which confounders were measured
    ∘ extent of imbalance between groups at baseline
    ∘ care with which adjustment was done (typically a judgment about the statistical modeling carried out by authors)
  - Low RoB requires that all important confounders are balanced at baseline (not primarily/not only a statistical judgment) OR measured ‘well’ and ‘carefully’ controlled for in the analysis.

Assess against pre‐specified worksheet. Reviewers will make a RoB judgment about each factor first and then ‘eyeball’ these for the judgment RoB table.

4. RoB from lack of blinding (assess for each outcome, as per existing RoB tool)
  - Low(1) / 2 / 3 / 4 / high(5) / unclear RoB item
  - Judgment needs to factor in:
    ∘ nature of outcome (subjective / objective; source of information)
    ∘ who was / was not blinded and the risk that those who were not blinded could introduce performance or detection bias
    ∘ see Ch.8
5. RoB from incomplete outcome data (assess for each outcome, as per existing RoB tool)
  - Low(1) / 2 / 3 / 4 / high(5) / unclear RoB item
  - Judgment needs to factor in:
    ∘ reasons for missing data
    ∘ whether amount of missing data balanced across groups, with similar reasons
    ∘ see Ch.8
6. RoB from selective reporting (assess for each outcome, NB different to existing Ch.8 recommendation)
  - Low(1) / 2 / 3 / 4 / high(5) /unclear RoB item
  - Judgment needs to factor in:
    ∘ existing RoB guidance on selective outcome reporting
    ∘ see Ch.8
    ∘ also, extent to which analyses (and potentially other choices) could have been manipulated to bias the findings reported, e.g. choice of method of model fitting, potential confounders considered / included
    ∘ look for evidence that there was a protocol in advance of doing any analysis / obtaining the data (difficult unless explicitly reported); QES very different from RCTs. RCTs must have a protocol in advance of starting to recruit (for REC/IRB/other regulatory approval); QES need not (especially older studies)
    ∘ Hence, separate yes/no items asking reviewers whether they think the researchers had a pre‐specified protocol and analysis plan.

**Confounding worksheet**

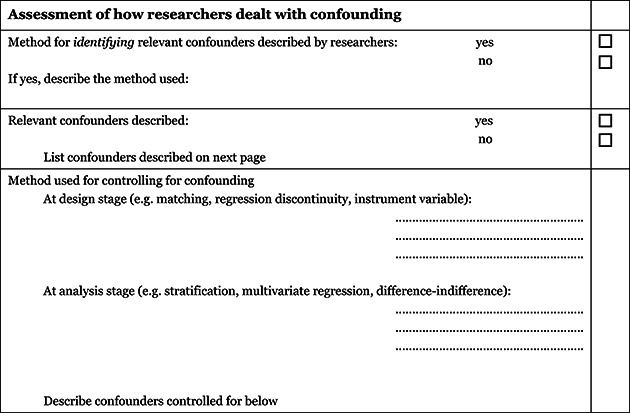

**Confounders described by researchers**

Tick (yes[0]/no[1] judgment) if confounder considered by the researchers [Cons'd?]

Score (1[good precision] to 5[poor precision]) precision with which confounder measured

Score (1[balanced] to 5[major imbalance]) imbalance between groups

Score (1[very careful] to 5[not at all careful]) care with which adjustment for confounder was carried out

**Table:** 

**Confounder**	Considered	Precision	Imbalance	Adjustment
Gender	❏	❏	❏	❏
Age	❏	❏	❏	❏
Socio‐economic status	❏	❏	❏	❏
Mental problems	❏	❏	❏	❏
History of drug misuse	❏	❏	❏	❏
Unobservables^20^	❏	Irrelevant	❏	❏
Other:	❏	❏	❏	❏
Other:	❏	❏	❏	❏

#### 9.1.3 User guide for unobservables

Selection bias is understood as systematic baseline differences between groups and can therefore compromise comparability between groups. Baseline differences can be observable (e.g. age and gender) and unobservable (to the researcher; e.g. motivation and ‘ability’). There is no single non‐randomised study design that always solves the selection problem. Different designs solve the selection problem under different assumptions and require different types of data. Especially how different designs deal with selection on unobservables varies. The “right” method depends on the model generating participation, i.e. assumptions about the nature of the process by which participants are selected into a programme.

As there is no universal correct way to construct counterfactuals we will assess the extent to which the identifying assumptions (the assumption that makes it possible to identify the counterfactual) are explained and discussed (preferably the authors should make an effort to justify their choice of method). We will look for evidence that authors using e.g. (this is NOT a complete list):

**Natural experiments:**

Discuss whether they face a truly random allocation of participants and that there is no change of behavior in anticipation of e.g. policy rules.

**Instrument variable (IV):**

Explain and discuss the assumption that the instrument variable does not affect outcomes other than through their effect on participation.

**Matching (including propensity scores):**

Explain and discuss the assumption that there is no selection on unobservables, only selection on observables.

**(Multivariate) Regression:**

Explain and discuss the assumption that there is no selection on unobservables, only selection on observables. Further discuss the extent to which they compare comparable people.

**Regression Discontinuity (RD):**

Explain and discuss the assumption that there is a (strict!) RD treatment rule. It must not be changeable by the agent in an effort to obtain or avoid treatment. Continuity in the expected impact at the discontinuity is required.

**Difference‐in‐difference (Treatment‐control‐before‐after):**

Explain and discuss the assumption that outcomes of participants and nonparticipants evolve over time in the same way.

### 9.2 TYPES OF OUTCOMES: INSTRUMENTS

#### 9.2.1 PTSD

**Table:** 

**Outcome**	**Instrument**	**Number of studies ‐ Synthesis sample**
PTSD	CIDI, CIDI 2.1	1
	Mississippi	2
	Questionnaire	2
	PC‐PTSD	4
	PCL, PCL‐C, PCL‐M, PCL‐S	16
	SCID	3
	TRICARE	2

#### 9.2.2 Depression

**Table:** 

**Outcome**	**Instrument**	**Number of studies ‐ Synthesis sample**
Depression	BDI	1
	CIDI	2
	CES‐D	2
	PHQ‐2, PHQ‐9	6
	PRIME‐MD	1
	Questionnaire	4
	SCID	2

#### 9.2.3 Substance abuse/Dependence

**Table:** 

**Outcome**	**Instrument**	**Number of studies ‐ Full Sample (Synthesis sample)**
Substance	AUDIT	4
	CIDI	4
	Questionnaire	4
	SCID	1
	PRIME‐MD	1

#### 9.2.4 Common mental disorder

**Table:** 

**Outcome**	**Instrument**	**Number of studies ‐ Synthesis sample**
Common mental disorder	GHQ‐12	5
BSI	1	
CIDI	1	
MCS	1	
	Hospitalization records	1

## 10 Figures

### 10.1 SENSITIVITY ANALYSIS

**Figure 10.1.1:**
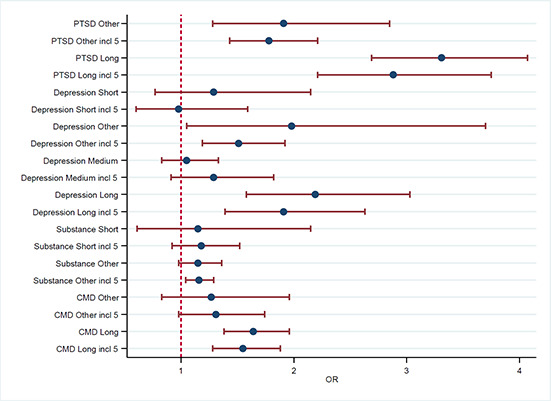
Forest plot. Absolute comparison. Including studies with score of 5 on the confounding item

**Figure 10.1.2:**
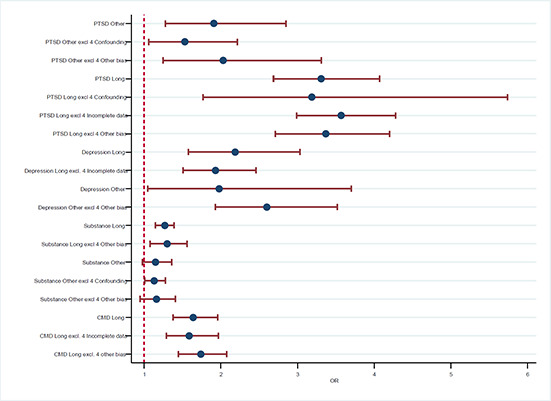
Forest plot. Absolute comparison. Excluding studies with score of 4 on the confounding, incomplete data and other risk of bias items

**Figure 10.1.3:**
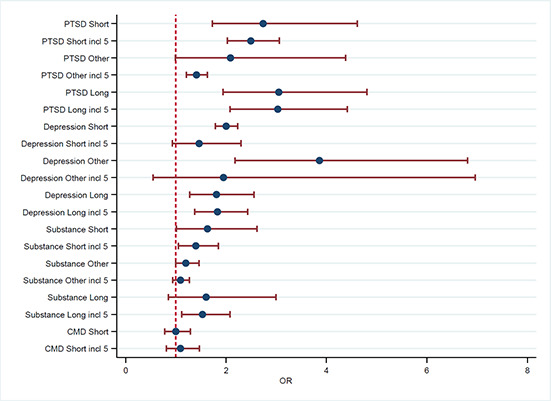
Forest plot. Relative comparison. Including studies with score of 5 on the confounding item

**Figure 10.1.4:**
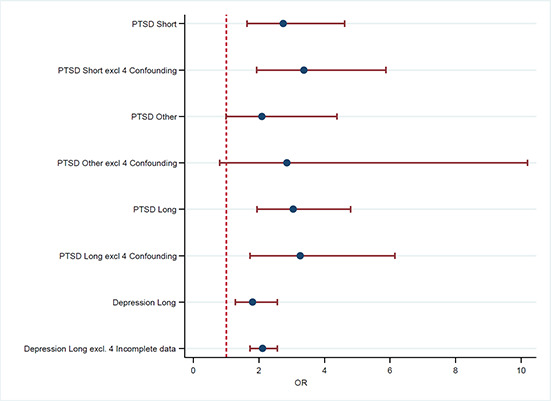
Forest plot. Relative comparison. Excluding studies with score of 4 on the confounding and incomplete data items

### 10.2 PUBLICATION BIAS

**Figure 10.2.1:**
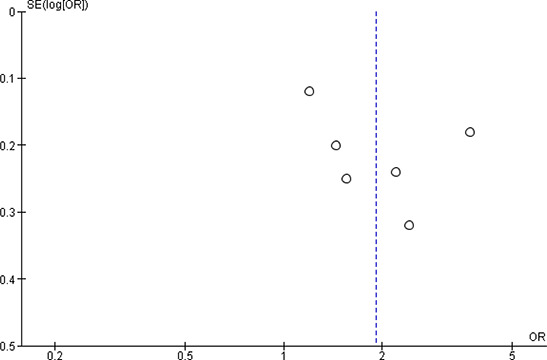
Funnel plot. Absolute comparison. PTSD, variable number of months past exposure.

**Figure 10.2.2:**
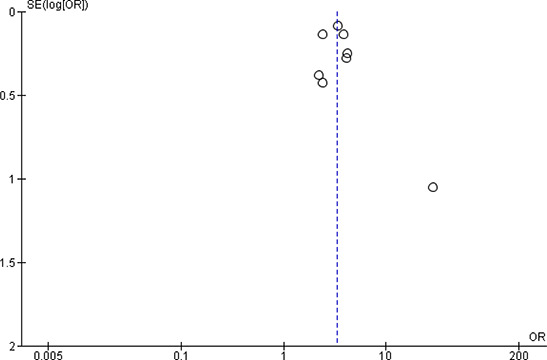
Funnel plot. Absolute comparison. PTSD, more than 24 months past exposure.

**Figure 10.2.3:**
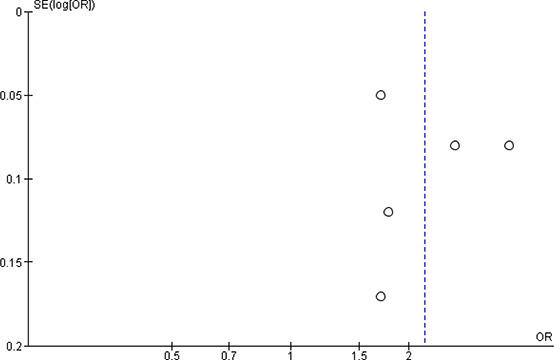
Funnel plot. Absolute comparison. Depression, more than 24 months past exposure

**Figure 10.2.4:**
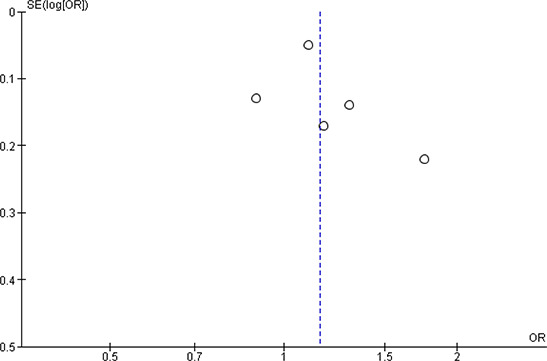
Funnel plot. Absolute comparison. Substance use, variable number of months past exposure

**Figure 10.2.5:**
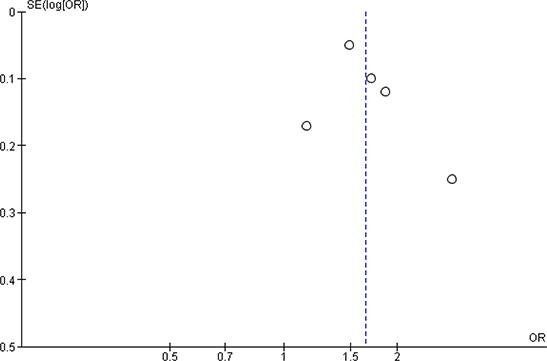
Funnel plot. Absolute comparison. Common mental disorder, more than 24 months past exposure

**Figure 10.2.6:**
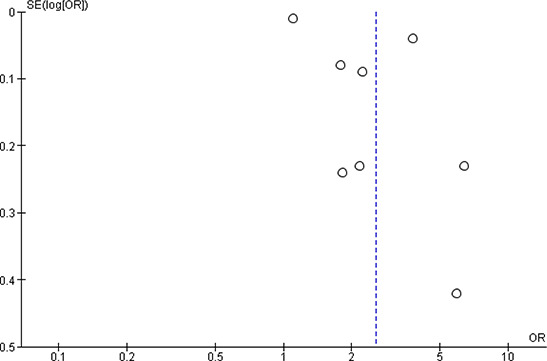
Funnel plot. Relative comparison. PTSD, 0 to 6 months past exposure.

### 10.3 FLOWCHART – LITERATURE SEARCH

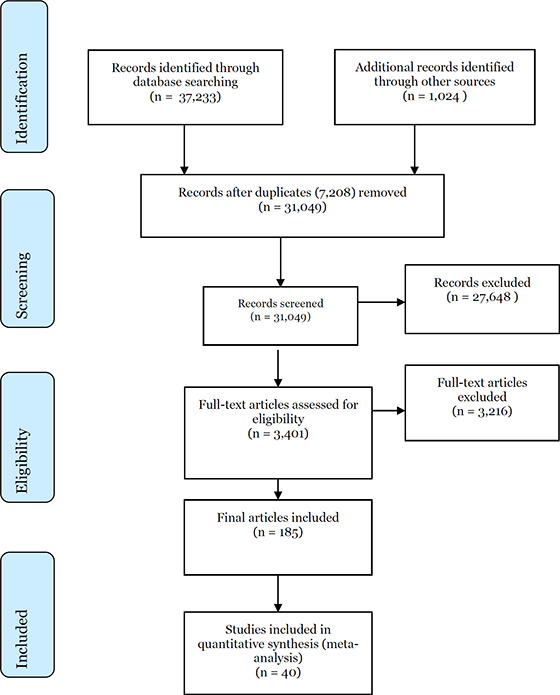

## 11 Online supplements

***List of online supplements***

1. Risk of bias tables
2. Characteristics of included studies
3. Search histories
4. Effect sizes (Excel sheet)

## Supporting information

Supplementary material

Supplementary material

Supplementary material

Supplementary material

## Footnotes

1 We note that these estimates rely on different samples and methodologies. For example some studies are restricted to looking only at deployed personnel, and thus lack a comparison group. Therefore the estimates cannot be given a causal interpretation in the sense of measuring the effect of deployment on PTSD.

2 UN peacekeeping operations include: UNTSO, UNMOGIP (since 1949), UNFICYP (since 1964), UNDOF (since 1974), UNIFIL (since 1978), MINURSO (since 1991), UNMIK (since 1999), UNMIL (since 2003), UNOCI (since 2004), MINUSTAH (since 2004), UNAMID (since 2007), MONUSCO (since 2010), UNISFA (since 2011), UNMISS (since 2011), MINUSMA (since 2013).

3 UNAMA, Afghanistan (since 2002).

4 NATO operations include: Kosovo (since 1999), Monitoring the Mediterranean Sea (since 2001), Afghanistan (since 2003), Supporting the African Union (since 2007), and Counter‐piracy off the Horn of Africa (since 2009).

5 The Multi‐National Force – Iraq (MNF–I) was a military command, led by the United States, which was responsible for Operation Iraqi Freedom. Multi‐National Force – Iraq replaced the previous force, Combined Joint Task Force 7, on 15 May 2004, and was later itself reorganized into its successor, United States Forces – Iraq, on 1 January 2010. The Force was significantly reinforced during the Iraq War troop surge of 2007. As of August 2009, all non‐U.S. coalition members had withdrawn from Iraq.

6 In the UK special forces is a separate branch, whereas in the US each branch have their own special forces component.

7 This criterion is eliminated in the current DSM‐5 (American Psychiatric Association, 2013). We retain the use of the DSM‐IV criteria here, since none of the included studies use DSM‐5 diagnostic criteria. However the careful reader should note the difference between DSM‐IV and DSM‐5.

8 In The Diagnostic and Statistical Manual of Mental Disorders (5th ed.; DSM–5; American Psychiatric Association, 2013) substance abuse/dependence has been renamed substance use. We retain the use of the DSM‐IV classifications here since all included studies used either DSM‐III, DSM‐III‐R, or DSM‐IV classification of mental disorders.

9 For example, Hotopf et al. (2006) use a variable “fitness to deploy” to adjust for confounding.

10 This risk of bias model was introduced by Prof. Reeves at a workshop on risk of bias in non‐randomised studies at SFI Campbell, February 2011. The model is a further development of work carried out in the Cochrane Non‐Randomised Studies Method Group (NRSMG).

11 Five studies were not assessed for risk of bias, because it became clear at the numerical coding stage that no effect sizes could be extracted from these studies.

12 If a study provided more than one effect size for the relevant outcome and comparison then these were averaged to create a synthetic effect size. For example Black et al. (2004) reported comparisons separately for army participants and for national guards. The two comparisons were combined to create one study level effect size.

13 Note that in DSM‐5 (American Psychological Association, 2013) PTSD is no longer classified as an Anxiety Disorder; rather it is classified under Trauma‐ and Stressor‐Related Disorders. We retain the DSM‐4 classification here to be consistent with primary studies.

14 None of the studies scored 4 on the other risk of bias item.

15 Using the conversion prevalence(deployed) = 1/[1+1/[OR*prevalence(non‐deployed)/(1‐prevalence(non‐deployed)]]

16 A total of 95 studies were judged to have too high risk of bias but the reduction due to lack of sufficient data to calculate an effect size and the reduction due to overlap of data sample preceded the reduction due to too high risk of bias.

17 Members of the review team at SFI Campbell were: the research assistants Tróndur Møller Sandoy, Ulrik Højmark Pedersen, Rasmus Henriksen Klokker, Freja Jørgensen, Julie Marie Nielsen, Amanda Weber, Sjúrdur Zachariasson, Ida Scheel Rasmussen, Pia Vang Hansen, Therese Lucia Friis, Marcel Mirzaei‐Fard, Asta Breinholt Lund, Stine Lian Olsen, Anne‐Sofie Due Knudsenand Bjørn Christian Viinholt Nielsen.

18 In the US setting downtime is typically called dwell time.

19 This risk of bias model was introduced by Prof. Reeves at a workshop on risk of bias in non‐randomised studies at SFI Campbell, February 2011. The model is a further development of work carried out in the Cochrane Non‐Randomised Studies Method Group (NRSMG).

20 See user guide for unobservables
