# Supplementary material for: Deployment of personnel to military operations: impact on mental health and social functioning
Source: Campbell Syst Rev. 2018 Jun 1;14(1):1–127. doi: 10.4073/csr.2018.6 (PMC8427986; doi:10.4073/csr.2018.6)
Supplement: Supplementary file 1 — Supplementary material [file CL2-14--s001.docx]

# Online Supplement 1: Risk of Bias Tables

This supplement contains risk of bias table for studies that are included in the review. The tables are organized in three sections. The first section (section 1.1) contains those studies that are not included in synthesis (analysis sample). A study may not be included in synthesis for the following reasons: a) we were unable to extract an effect size from the study, b) the study had sample overlap with another study, and that study was rated with a smaller risk of bias. Section 1.2 contains the studies that are part of the analysis sample, both main and sensitivity analysis. Section 1.3 contains extra studies from the updated search.

## Risk of Bias Tables: Studies not in Analysis Sample

| **Author** | Adler A. B., Britt T. W., Castro C. A., McGurk D. Bliese P. D. | Axelrod S. R., Morgan C. A., Southwick S. M. | Barrett, D. H., Doebbeling, C. C., Schwartz, D. A., Voelker, M. D., Falter, K. H., Woolson, R. F. et al. |
| --- | --- | --- | --- |
| **Year** | 2011 | 2005 | 2002 |
| **Sequence Generation** | High | High | High |
| **Allocation Concealment** | High | High | High |
| **Blinding** | 4 | 4 | 4 |
| Description |  | Non-blind |  |
| **Incomplete Outcome Data** | Unclear | Unclear | 3 |
| Description | Response rate 72% and only a subset used (those who had completed an earlier survey of which nothing further is reported). P 382 seems to indicate that 2297 were approached (full population), of which 1651 agreed to partake, but PTSD only available for 1051 of the 1651. | Not mentioned how many they attempted to survey. Unlikely they reached all. | No comparison of non-responder to responders. Response rate 76% of the eligible study subjects (91% of those contacted). Excluded 1% because of missing information on one or more of the variables of interest. |
| **Reporting Bias** | 1 | 1 | 1 |
| Description |  |  | Probably yes |
| **Other Bias** | 1 | 2 | 2 |
| Description |  | Recall bias; bias from instruments used. | Self-report bias (telephone interviews). |
| **Apriori Protocol** | Unclear | Unclear | Yes |
| Description | Page 382 says study approved by WRAIR IRB. | Not explicitly stated | Probably yes. See Doebbeling et al. (2002) |
| **Apriori Plan of Analysis** | Unclear | Unclear | Unclear |
| Description | Not stated | Not explicitly stated | "examine the relation between PTSD and perceived physical health and health-realted qualit of life … " p. 196 |
| **Confounding** | 4 | 5 | 4 |
| Description | Not considered are mental health history, ethnicity, previous deployment and age. No imbalances are shown or discussed although active duty soldiers only / 96% male. Interestingly they control for gender. Partial correlation. | None controlled for. Reason not explained. | "Each item's odds ratio has been adjusted for all other stratification variables (deployment status, age, sex, race ,rank, branch and military status) and smoking status." (footnote to table 2, p.200) |
| Relevant confounders described by researchers? | No | Discussed on page 271. | No |
| Assessment of relevant confounders | Active duty soldiers in a brigade combat team. Otherwise only rank and gender controlled for. No imbalances shown/discussed). | Nothing controlled for. They have info on: gender, age, education and ethnicity. | All but mental health history and previous deployments. In addition current smoking status which can be considered an outcome in itself of deployment. Thus a bad control as that variable introduce endogeneity on RHS. |
| **Method**  **(Design Stage)** | None | None | Stratification |
| **Method**  **(Analysis Stage)** | Adjusted correlation | OLS | Logistic regression |

| **Author** | Benda, B. B. & House, H. A. | Bramsen I., Dirkzwager A. J. E., van der Ploeg H. M. | Bray R. M., Pemberton M. R., Lane ME., Hourani L. L., Mattiko M. J., Babeu LA., |
| --- | --- | --- | --- |
| **Year** | 2003 | 2000 | 2010 |
| **Sequence Generation** | High | High | High |
| **Allocation Concealment** | High | High | High |
| **Blinding** | 4 | 4 | 4 |
| Description |  | Non-blind |  |
| **Incomplete Outcome Data** | 1 | Unclear | 3 |
| Description | Response rate women: 93% and men 90%. Apparantly no missing data. | Not mentioned how many they attempted to survey. Unlikely they reached all. | Response rate 70.6%. Missing data rate not reported but mentioned that there are missing data. |
| **Reporting Bias** | 1 | 1 | 3 |
| Description |  |  | No reports on what demographic variables are corrected for, nor the procedure for correction. I would assume that these are just stratum weights? |
| **Other Bias** | 1 | 3 | 1 |
| Description |  | They mention recall bias, and the fact that the pre-deployment psychological screen would determine deployment decision, and as such lead to attempts to manipulate the screen (p 1118). |  |
| **Apriori Protocol** | Unclear | Unclear | Unclear |
| Description |  | Not explicitly stated |  |
| **Apriori Plan of Analysis** | Unclear | Unclear | Unclear |
| Description |  | Not explicitly stated |  |
| **Confounding** | 5 | 4 | 5 |
| Description | Controls for post deployment mental health, abuse, family, friends, social support etc. | Corrects for age, proxy for mental health and education. Army, male, first deployment, voluntary fulfilment of compulsory military conscription sample only. Does not discuss plausibility of exposure being exogenously assigned. | "Estimates have been adjusted for sociodemographic differences between the three combat/theater groups". Otherwise nothing is mentioned. |
| Relevant confounders described by researchers? | No | Discussed on p1116. They have data on: gender, age, education, rank, deployment length. | No |
| Assessment of relevant confounders | Only gender, age and ethnicity and a lot of other post deployment covariates. Imbalances not mentioned. | All confounders considered except ethnicity (which may or may not be relevant here). No discussion of identification. | None |
| **Method**  **(Design Stage)** | Logistic regression | None | Some stratification is applied |
| **Method**  **(Analysis Stage)** | Logistic regression | OLS | Adjusted percentages calculated, weighting using SUDAAN. |

| **Author** | Browne T., Iversen A., Hull L., Workman L., Barker C., Horn O., Jones M., Murphy D., Greenberg N., Rona R., Hotopf M., Wessely S., Fear N. T. | Castro C. A., McGurk D. | Cesur, R., Sabia, J. J., & Tekin, E. |
| --- | --- | --- | --- |
| **Year** | 2008 | 2007 | 2012 |
| **Sequence Generation** | High | High | High |
| **Allocation Concealment** | High | High | High |
| **Blinding** | 4 | 4 | 4 |
| Description | Non-blind |  |  |
| **Incomplete Outcome Data** | 3 | Unclear | Unclear |
| Description | Response rate: 61%. Responders and non-responders differ (p.628) Stated for the total sample and not the subsample used. | No information given. 1320 soldiers (Army) and 447 Marines (Navy) surveyed. | We restrict our sample to respondents who provided non-missing information on mental health and military service at Wave IV when the respondents were young adults ages 24 and 33. Number with missing information not reported; Respondents at Wave I: 20,745; Respondents at Wave IV: 15701. Of these 1080 have military service after wave 1. |
| **Reporting Bias** | 1 | 1 | 1 |
| Description |  |  | Report results of several analyses and they perform other robustness tests as well (p. 18 ff.) |
| **Other Bias** | 2 | 1 | 1 |
| Description | Concerns between recall bias and heavy drinking. |  |  |
| **Apriori Protocol** | Unclear | Unclear | No |
| Description |  | Not stated | Add Health is not specifically for the military population. |
| **Apriori Plan of Analysis** | Unclear | Unclear | Unclear |
| Description |  | Not stated |  |
| **Confounding** | 3 | 5 | 1 |
| Description | No discussion regarding plausibility of risk events being as good as randomly assigned, conditional on deployment. Comprehensive set of confounders. | Stratified on Soldier/Marine. Otherwise nothing controlled for. Description on page 16 reveals large differences between lo/med/hi combat exposures. | We rely on evidence that deployment assignments of active-duty units are unrelated to the characteristics of soldiers or their families (Engel et al, 2010; Lyle, 2006) to identify the causal effects of combat. They test whether deployment assignment to combat zones is exogenous; only the "other" race category is significant (p. 16). |
| Relevant confounders described by researchers? | Yes; p.630 | No | Discuss selection on p. 54 ff. |
| Assessment of relevant confounders | All except ethnicity considered, but balance not reported. | Nothing considered, except stratified on Army/Marine. | Yes, except Number of previous deployments and more is added. Do not show imbalance on military variables but state they control for them. |
| **Method**  **(Design Stage)** | None | No information given | None |
| **Method**  **(Analysis Stage)** | Logistic regression | Nothing | OLS + fixed effects |

| **Author** | Ciccone D. S., Kline A. | Coughlin S. S., Kang H. K., Mahan C. M. | Di Nicola M., Occhiolini L., Di Nicola L., Vellante P., Di Mascio R., Guizzardi M., Colagrande V., Ballone E. |
| --- | --- | --- | --- |
| **Year** | 2012 | 2011 | 2007 |
| **Sequence Generation** | High | High | High |
| **Allocation Concealment** | High | High | High |
| **Blinding** | 4 | 4 | 4 |
| Description | Non-blind | Non-blind |  |
| **Incomplete Outcome Data** | 3 | 3 | Unclear |
| Description | 922/2543 used in longitudinal analyses. Compares with non-responders. | 29607 eligible. Responses obtained from 6111+3859, so less than 40% response rate. No comparison of response/non-response. | Nothing reported |
| **Reporting Bias** | 1 | 1 | 1 |
| Description |  |  |  |
| **Other Bias** | 1 | 1 | 1 |
| Description | Anonymous self-reports |  |  |
| **Apriori Protocol** | Unclear | Unclear | Unclear |
| Description | But states prospective study. | Not explictily stated |  |
| **Apriori Plan of Analysis** | Unclear | Unclear | Unclear |
| Description | Not explicitly stated | Not stated |  |
| **Confounding** | 5 | 5 | 5 |
| Description | Does not discuss covariate balance with exposure. Do not control for any confounders. | Enlistment status, number of previous deployments, and prior health not considered. Model 1 controls for (current) BMI, income, education, (current) smoking status, which must all be considered outcomes of deployment. Not clear which other measures are current. | Only age and other prior peacekeeping missions considered of the prespecified confounders considered and nothing controlled for. Other confounders considered but not controlled for, some with large imbalances. |
| Relevant confounders described by researchers? | Yes, p.2056-57 | Yes, p.3. Also table 1, p.8 | No |
| Assessment of relevant confounders | Only Guards included. | Enlistment status, number of previous deployments, and prior health not considered. Imbalance on ethnicity, rank, education (and income=correlated) and minor on enlistment status. In addition bad controls including: BMI, current smoking status. | Only age and previous peacekeeping missions considered. In addition marital status, education, family, employment, years of enrollment, task and reason for enrollment considered. Nothing controlled for. Imbalance on previous missions, years of enrollment, task and reason for enrollment. |
| **Method**  **(Design Stage)** | None | None | None |
| **Method**  **(Analysis Stage)** | Correlation | Logistic regression | None |

| **Author** | Dlugosz, L. J., Hocter, W. J., Kaiser, K. S., Knoke, J. D., Heller, J. M., Hamid, N. A. et al. | Dryden A. E. | Dutra, L. I. S. S., Grubbs, K. A. T. H., Greene, C. A. R. O., Trego, L. L., MCCartin, T. L., Kloezeman, K. A. R. E. et al. |
| --- | --- | --- | --- |
| **Year** | 1999 | 2013 | 2011 |
| **Sequence Generation** | High | High | High |
| **Allocation Concealment** | High | High | High |
| **Blinding** | 3 | 4 | 4 |
| Description | Administrative data from hospitalizations. Reliability of diagnosis reviewed by independent assessors, who are blind to whether Gulf Service. |  | Questionnaire administered in connection with post-deployment wellness visit. |
| **Incomplete Outcome Data** | 1 | Unclear | Unclear |
| Description | Full cohort observed from administrative records. | No information given | No description of how many they approached. Appears to be convenience sample. Described as pilot study. |
| **Reporting Bias** | 1 | 1 | 1 |
| Description | Probably yes |  | Probably yes |
| **Other Bias** | 2 | 1 | 2 |
| Description | From our perspective: PTSD and Depression are measured with error (they form a subset of the diagnoses reported). To the extent that these diagnoses are rare and incidence differs between control and treat this is a potential bias. Mood disorders [including depression] may have been treated on outpatient basis (p. 1274). Discharged personnel are not in the sample. If diagnoses are reason for discharge and it varies between treat and control this could also introduce bias (p 1275). |  | Recall bias |
| **Apriori Protocol** | Unclear | Unclear | Yes |
| Description | Administrative data |  | The study protocol was approved by the Human Use Committee at Tripler Army Medical Center. |
| **Apriori Plan of Analysis** | Unclear | Unclear | Unclear |
| Description |  |  |  |
| **Confounding** | 3 | 5 | 5 |
| Description | In addition to Gulf War deployment and Gulf war occupation, variables considered for inclusion in the regression model were age, race, education, length of service, branch of service, medical treatment facility catchment area, psychiatric hospitalization experience (a variable constructed from ratio of psychiatric to non-psychiatric hospitalization experience in geographic areas), prewar (October 1, 1988 through August 1, 1990) military hospitalization for mental disorders, dates of service in the Gulf War theater, Unit Identification Code, Armed Forces Qualification Test score, length of deployment to the Gulf War theater (# 120 days, 120 days), duty occupation in a health care field, gender, marital status, and military rank (p.1269). Variables that did not contribute significantly (P-value 0.05) to the model were removed. | Nothing considered / Hypothesis tests PTSD vs CES/TLEQ. Analysis is Pearson correlation. No control for confounders, although they are available. | The sample consists of women from the army. Otherwise nothing is controlled for and imbalances not shown. "Demographics measured in the study included year of birth, ethnicity, education, marital status, number of children, military branch, military affiliation (i.e., active duty, reservist, National Guard), Military Occupational Specialty, rank, length of time in the service, number of deployments, and military operation deployment (i.e., OIF, OEF)." (p.29) |
| Relevant confounders described by researchers? | "Mental disorder hospitalizations occur in the context of influential sociodemographic and individual factors. Several of the risk factors for mental disorder hospitalization in the present study (e.g., gender, marital status, age, education, military rank) are consistent with correlates of serious mental impairment in the National Comorbidity Study [35]. For example, in most summary diagnostic categories of mental disorders in the present analysis, hospitalization risk was greatest for unmarried persons; women; and persons in the lowest age, rank (E1-E4), and education categories. In the National Comorbidity study, serious mental impairment prevalence was highest among unmarried persons; women; and persons in the lowest age, income, and education categories." (p 1274) | No / Several are discussed on page 7. Including enlistment, prior trauma. | No |
| Assessment of relevant confounders | All but number of previous deployments. They additionally control education and marital status. They don’t report imbalance between treat/control (only p. 1269): Gulf War deployment status varied widely according to gender, military service branch, age, education, marital status, race, and rank. | Nothing considered | Only women from Army are included, table 2 presents F test for joint significance of the two covariates. Df = 51, meaning they have estimated a model consisting of CES, MSH and a constant (N=54). |
| **Method**  **(Design Stage)** | None (full population) | No information given | None |
| **Method**  **(Analysis Stage)** | Cox proportional hazard | None | None |
| **Author** | Edwards R. D. | Elbogen E. B., Sullivan C. P., Wolfe J., Wagner H. R., Beckham J. C. | Engdahl, R. M., Richardson, J. D., Elhai, J. D., & Frueh, B. C |
| **Year** | 2012 | 2013 | 2011 |
| **Sequence Generation** | High | High | High |
| **Allocation Concealment** | High | High | High |
| **Blinding** | 4 | 4 | 4 |
| **Description** |  |  |  |
| **Incomplete Outcome Data** | 3 | 3 | 2 |
| **Description** | The data is a subsample of a larger data set. For the larger data set the response rate is 61%. Missing data not reported. | Response rate 47%. Follow up retention rate 79%, recent homelessness at baseline very weekly related to attrition. | Response rate 71.3%. No data on non-responders exists. Only those who had served after 1990 were included and of these 4% had missing data for more than five PCL items and were excluded. Another 14% had missing data on 1-3 items and multiple imputation procedures were used. |
| **Reporting Bias** | 3 | 1 | 1 |
| **Description** | Do not report how many are lost to common support implying the effective N is unclear. |  |  |
| **Other Bias** | 1 | 1 | 1 |
| **Description** |  |  |  |
| **Apriori Protocol** | Unclear | Unclear | Unclear |
| **Description** |  |  |  |
| **Apriori Plan of Analysis** | Unclear | Unclear | Unclear |
| **Description** |  |  |  |
| **Confounding** | 5 | 5 | 5 |
| **Description** | Not considered: Mental health history, rank, enlistment status and previous deployment. Somewhat large imbalances on those considered in particular gender. Do not report on common support and the difference in employment changes dramatically due to matching (as do others outcomes as well). Surprisingly the author argues that outcomes are very stable relative to simple mean differences. | Nothing controlled for. Table 2, p.251, presents a bivariate association between combat exposure and homelessness. CE used as control variable in main analysis (table 3). | Do not report on imbalances on any confounders and do not control for anything. |
| **Relevant confounders described by researchers?** | No | No | No |
| **Assessment of relevant confounders** | Not considered: Mental health history, rank, enlistment status and previous deployment. Somewhat large imbalances on those considered in particular gender. | None | None |
| **Method**  **(Design Stage)** | None | Gender stratified | None |
| **Method**  **(Analysis Stage)** | Propensity score matching (using nearest neighbour match implemented as match in Stata). | None | Stratified sampling from a larger database of 18,443 individuals who had been identified with health conditions after serving in the Canadian armed forces and were therefore either receiving or eligible for a disability pension from Veterans Affairs Canada. |

| **Author** | Engelhard, I. M. & van den Hout, M. A. | Fear N. T., Jones M., Murphy D., Hull L., Iversen A. C., Coker B., Machell L., Sundin J., Woodhead C., Jones N., Greenberg N., Landau S., Dandeker C., Rona R. J., Hotopf M., Wessely S. | Fritch A. M., Mishkind M., Reger MA., Gahm G. A. |
| --- | --- | --- | --- |
| **Year** | 2007 | 2010 | 2010 |
| **Sequence Generation** | High | High | High |
| **Allocation Concealment** | High | High | High |
| **Blinding** | 4 | 4 | 4 |
| Description |  | Participants not blinded. Investigators collected data. | Non-blind |
| **Incomplete Outcome Data** | 3 |  | 1 |
| Description | Response/participation rate 72%. There are missing data but level not reported. 383 out of 385 were measured at baseline. 276 of 383 were followed up. | Estimates corrected for non-response. Comparison of non-response demographics to responders Table 1, p.1786. Rather low response rate: 56% (p.1794). | Full intake sample (mandatory survey upon entry). |
| **Reporting Bias** | 3 | 2 | Unclear |
| Description | Response/participation rate 72%. There are missing data but level not reported. 383 out of 385 were measured at baseline. 276 of 383 were followed up. | Many estimates left out, but reported in text (e.g. page 1790). | They do not clearly state how dependent variables are constructed. Only clue is on p250: "... Mental heal screening score." Models with full set of confounders in first step not presented. |
| **Other Bias** | 4 | Unclear | 2 |
| Description | Do not show or mention imbalances. Deployed sample only. Do not subdivide sample based on number of minor/major stressors. | Not stated whether questionnaire answers were available to sponsor (MoD) . | Possible recall bias |
| **Apriori Protocol** | 1 | Yes | Yes |
| Description |  | Ethics approval from MOD's research ethics committee and King's College Hospital local research ethics committee (p 1786). | Local Department of Clinical Investigation (p 249). |
| **Apriori Plan of Analysis** | Unclear | Unclear | Unclear |
| Description |  | Not explicitly stated | Not stated |
| **Confounding** | Unclear | 3 | 4 |
| Description |  | Some imbalances on demographics and 3 confounders not considered. | No discussion of balance across exposure. Only a few confounders are entered into the model (separately), but dropped from final model due to insignificance (stated). |
| Relevant confounders described by researchers? | 5 | Yes, p.1789. Age, sex, rank, service, engagement type (Reg/reserve). | Yes, table 1, p.250 |
| Assessment of relevant confounders | Not discussed | Main analysis does not control for prior mental health, number of deployments and ethnicity. | Gender, rank, race, age, (and education) controlled for in first model. Childhood physical abuse 'controlled' for, may act as substitute for prior mental health. Not adjusted for: number of prior deployments enlistment status and branch. |
| **Method**  **(Design Stage)** | None | Random sampling design | None |
| **Method**  **(Analysis Stage)** | Only neuroticism controlled for | Logistic regression | OLS |

| **Author** | Gackstetter, G. D., Hooper, T. I., Al Qahtani, M. S., Smith, T. C., Memish, Z. A., Schlangen, K. M. et al. | Gade, D. M. and Wenger J. B. | Garber B. G., Zamorski M. A., Jetly C. R. |
| --- | --- | --- | --- |
| **Year** | 2005 | 2011 | 2012 |
| **Sequence Generation** | High | High | High |
| **Allocation Concealment** | High | High | High |
| **Blinding** | 4 | 4 | 4 |
| Description |  |  | Non-blind |
| **Incomplete Outcome Data** | Unclear | Unclear | 4 |
| Description | "To compare the hospitalization experience of the two groups of SANG soldiers, while accounting for attrition, including resignation or discharge from military service, we used Cox proportional hazard regression analysis." However authors mention themselves that hospitalisation may have occurred at different facilities (unmeasured) and that it may vary between T and C: "Since no tertiary care facility existed near Al Khafji during our study period, all Al Khafji soldiers with health conditions requiring hospital admission were referred to KAMC in Riyadh. On the other hand, alternate health care services were available to the soldiers in the Riyadh area, a more urban and populous region. This may explain, in part, the consistently larger number of hospitalizations across all major diagnostic categories in the Al Khafji group." | Nothing stated; The extended interview response rate for RDD. Sample veterans were 76.4 percent (Source: http://www1.va.gov/VETDATA/docs/SurveysAndStudies/NSV_Methodology_Report.pdf, p. ix). | Response rate: 1572/2779. No discussion of non-responders, though they appear to have this data. |
| **Reporting Bias** | 2 | 1 | 1 |
| Description | No protocol but full population used, and all causes for hospitalisation recorded (administrative data). | Report results for the OLS regression with and without controls for physical health and perform and report the results of robustness analysis (latent class) (p. 412). |  |
| **Other Bias** | Unclear | 4 | 2 |
| Description | "Battle of Al Khafji took place just after a new contingent of SANG officers (about 300) completed their training and were immediately assigned to the Al Khafji region. A greater than expected rate of resignation from this new group of officers may have contributed to fewer overall hospitalizations since automated health data are not available for those who resign or are discharged from SANG service." | Estimate of served in combat probably negatively biased as they include exposure to dead, dying or wounded people. | They mention reporting bias due to survey. |
| **Apriori Protocol** | Unclear | Unclear | Yes |
| Description | Probably no |  | Page 739. "The research protocol was approved by the CF's Social Science Research Review Board…" |
| **Apriori Plan of Analysis** | Unclear | Unclear | Unclear |
| Description | Probably yes; "In late 1999, our collaborative research team met in Saudi Arabia at King Abdul Aziz Medical City (KAMC), formerly known as King Fahad National Guard Hospital. KAMC is a large, 560-bed, tertiary care medical centre in Riyadh, established in 1981 to provide primary care and advanced diagnostic, therapeutic, and other referral services for SANG soldiers and their family members. Two Saudi researchers from KAMC and eight from the United States, representing the Uniformed Services University of the Health Sciences (USUHS), the Centres for Disease Control and Prevention (CDC), and the Department of Defence Centre for Deployment Health Research at the Naval Health Research Centre (NHRC), comprised our research team." |  | Not explicitly stated |
| **Confounding** | 5 | 3 | 5 |
| Description | Age, length of service. Use Guards only. | Although it is true that all Gulf War veterans chose to enter military service, among this population exposure is still exogenously determined by the circumstances of combat. We lack the data to control for whether a person was drafted, but possess and use the data on whether an individual was an officer or not. Clearly, officers are systematically different than enlisted soldiers, and their selection process is more rigorous (footnote 8 p. 405). | No corrections for any confounders. Modelling involves univariate comparisons and if not significant left out of multivariate (p739). In their defence they do not give estimates a causal interpretation, i.e. they do not argue that exposure is exogenous. |
| Relevant confounders described by researchers? | Yes, "Preliminary analyses using Cox regression included estimates of crude RRs for location during the Gulf War (Al Khafji vs Riyadh), age (quartiles), length of service (quartiles), military rank (officer vs enlisted), and active-duty status (active, resigned/discharged, detained/transferred), and at least one hospitalization for any cause. Regression diagnostics revealed the absence of collinearity, but the likely presence of significant interaction between age and length of service. A manual, backward stepwise regression technique was used to construct the final model, which included age and length of service as continuous variables, age by length of service interaction term, and location." | Because the previously cited literature indicates that there are number of pre- and post-exposure factors that mitigate or exacerbate mental health outcomes, we control for a number of financial and personal characteristics. While exposure is exogenously determined, the controls for physical health are endogenous to mental health (p. 405). | No |
| Assessment of relevant confounders | Not all confounders from list are used (only age, gender, and rank). | Yes, except mental health history, Duty/Enlistment status (Active, Reserve/Guard) and number of previous deployments and more is added. | They have a host of demographic variables. Nothing used in final regression model (except marital status). They have following info: Rank, component, first language, marital status, military tenure, total number of UN/NATO tours, deployment location. No sheet completed since they do not correct for confounders. |
| **Method**  **(Design Stage)** | None, as full sample of SANG population. | None | None |
| **Method**  **(Analysis Stage)** | Cox proportional hazard model | Discrete factor maximum likelihood. Since the endogeneity of physical health may bias our OLS estimates, we estimate a series of DFML models to control for unobserved heterogeneity in the absence of our physical health controls. | Logistic regression |

| **Author** | Gray G. C., Coate B. D., Anderson C. M., Kang H. K., Berg S. W., Wignall F. S., Knoke J. D., Barrett-Connor E. | Gray G. C., Kaiser KS., Hawksworth AW., Hall F. W., Barrett-Connor E. | Han S. C., Castro F., Lee L. O., Charney M. E., Marx B. P., Brailey K., Proctor S. P., Vasterling J. J. |
| --- | --- | --- | --- |
| **Year** | 1996 | 1999 | 2014 |
| **Sequence Generation** | High | High | High |
| **Allocation Concealment** | High | High | High |
| **Blinding** | 3 | 3 | 4 |
| Description | Non-blind but administrative register data. | Assessors were blind to deployment status of participants. |  |
| **Incomplete Outcome Data** | 4 | 3 | 4 |
| Description | Only hospitalizations on military facilities. Outcomes for those who separated are missing. Large and significant differences in the separation from the military among treat and comparison, table 1, p.1507. | "Unit participation ranged from 26.1% to 71.0% of eligible Seabees." (p759). "There were no differences between participants and nonparticipants with respect to age group, race, marital status, and service entry intellectual aptitude scores" (p.759). Details not presented. | Response rate 94% at baseline enrollment. Women excluded. Of the remaining 72.1 % participated in post deployment assessment (non-participation mostly because of relocation and separation from active service). Non-participants included in analysis (using multiple imputations). |
| **Reporting Bias** | 1 | 1 | 1 |
| Description |  |  |  |
| **Other Bias** | 1 | 1 | 1 |
| Description |  |  |  |
| **Apriori Protocol** | Unclear | Yes | Unclear |
| Description | Not explicitly stated | "This study was approved by the Committee for the Protection of Human Subject at the Naval Health Research Center (San Diego, CA)." (p.758) | Not stated |
| **Apriori Plan of Analysis** | Unclear | Unclear | Unclear |
| Description | Not stated | Not explicitly stated | Not stated |
| **Confounding** | 5 | 5 | 4 |
| Description | Hospitalization rates for the ICM-9-CM diagnoses of interest are standardized with respect to age and gender distribution only. | Appear to have confounder data p.758-759, but only raw estimates presented. | Not considered: previous deployment, rank and ethnicity. In addition control for education, post deployment life events (and pre-deployment unit support, support during deployment, post-deployment social support). Outcome imputed for 28% (in total and not reported separate for Army and Guards). Table 1 shows participant/non-participant differences, not separated either. / They do control for pre-deployment PTSD which is probably the best possible control given the outcome of interest (it would basically subsume all the omitted variables). They do "overcontrol" by including post-deploy life events which PTSD might influence. I would use step 1 results (though they are stable). |
| Relevant confounders described by researchers? | Yes, p.1506-7 | Yes; table 1, p760. Confounders presented: Age, height, weight, race, marital status, sex, education, hospitalisations and pregnancies. | No |
| Assessment of relevant confounders | Only include active duty, otherwise age and gender only confounders adjusted for (other estimates for broader hospitalization categories corrected for additional confounders). State (p. 1506) that there are imbalances on age and gender and only to a lesser extent on the remaining available confounders (ethnicity, marital status, rank, branch, salary and occupation). | Table 1 suggests imbalances on confounders (age, gender). Not all listed confounders included. | Analysis separated between Army only and Guards only samples. Men only. Control for age, mental health history. No relevant imbalances shown. Not considered: previous deployment, rank and ethnicity. In addition control for education, post deployment life events (and pre deployment unit support, support during deployment, post deployment social support). |
| **Method**  **(Design Stage)** | None | None | Exclude women and divide into Army and Guards. |
| **Method**  **(Analysis Stage)** | Standardized rate ratios using direct method (wrt age and gender). | None | Regression |

| **Author** | Harmon S. C., Hoyt T. V., Jones MD., Etherage J. R., Okiishi J. C. | Heron E. A., Bryan C. J., Dougherty C. A., Chapman W. G., | Hoge, C. W., Castro, C. A., Messer, S. C., McGurk, D., Cotting, D. I., & Koffman, R. L. |
| --- | --- | --- | --- |
| **Year** | 2012 | 2013 | 2004 |
| **Sequence Generation** | High | High | High |
| **Allocation Concealment** | High | High | High |
| **Blinding** | 4 | 4 | 4 |
| Description | Non-blind |  | Non-blind. Investigators engaged directly in data collection. |
| **Incomplete Outcome Data** | 1 | 2 | 3 |
| Description | 2413 participants. 94 were excluded for missing one or more items. Of remaining 108 had partially completed demographic data (retained as missing data not related to constructs of interest). (p.369) | N = 168 pre-deploy. Lowest response is 1 mos post (95/168, 57%), and max response is 6 mos post (137/168). | Overall, 58% of the soldiers and Marines form the selected units were available to attend the recruitment briefings (79% of the soldiers before deployment ©, 58% of the soldiers after deployment in OEF Afghanistan (T1), 34% of the soldiers after deployment in OIF (T2), and 65% of the Marines after deployment in OIF (T3)" (p.15). The differential response rate is a concern although most of the non-responses were due to work and training schedules. Missing data level 0.2%-6% (own calc.). |
| **Reporting Bias** | 1 | 1 | 1 |
| Description |  |  |  |
| **Other Bias** | 2 | 1 | 1 |
| Description | Mandatory survey (PDHRA). Possible bias from underreporting of symptoms. |  | Anonymous questionnaire |
| **Apriori Protocol** | Unclear | Unclear | Yes |
| Description | "The Madigan Healthcare System IRB approved use of de-identified screening data for analysis." (p.369) | "The current study was reviewed and approved by the Wilfro Hall Medical Center IRB." (p.1036) | "The study was conducted under a protocol approved by the IRB of the WRAIR" (p.15). |
| **Apriori Plan of Analysis** | Unclear | Unclear | Unclear |
| Description | Not stated | Not stated |  |
| **Confounding** | 5 | 4 | 5 |
| Description | No confounders controlled for. Imbalances not shown or discussed. | Considered: previous exposure (proxy for previous trauma), gender, age, branch, enlistment status (Active duty Airforce). In addition: control for Depression (when considering PTSD), and vice versa. Have but do not control for: ethnicity, rank. Balance not discussed. | Only age, gender, race, and rank considered, but not controlled for. C v T3 has large imbalances and Marines are compared to Army. C v T1+T2 smaller imbalances on limited number of confounders and Army compared to Army. |
| Relevant confounders described by researchers? | No | No, only describe which variables are included in regressions. | No |
| Assessment of relevant confounders | Nothing controlled for although only included Army combat brigade soldiers. | Considered: previous exposure (proxy for previous trauma), gender, age, branch, enlistment status (Active duty Airforce). In addition: control for Depression (when considering PTSD), and vice versa. Have but do not control for: ethnicity, rank. Balance not discussed. | Age, gender, race, rank considered but not controlled for. Large imbalance on age (C v T3), and race (C v T2), and rank (C v T3) and T3 is Marines and C, T1, T2 is Army. |
| **Method**  **(Design Stage)** | None | Active Duty Air Force only | None |
| **Method**  **(Analysis Stage)** | None | "HLM" | None |

| **Author** | Hooper, R., Rona, R. J., Jones, M., Fear, N. T., Hull, L., & Wessely, S. | Horton, Jacobson, Wong, Wells, Boyko, Smith, Ryan & Smith |
| --- | --- | --- |
| **Year** | 2008 | 2013 |
| **Sequence Generation** | High | High |
| **Allocation Concealment** | High | High |
| **Blinding** | 4 | 4 |
| Description |  |  |
| **Incomplete Outcome Data** | 4 | Unclear |
| Description | 1382/2246 (61.5%) responded to first survey. Follow up: 941/1359 (69.2%) responded. No analysis of demographics for non-responders presented. Authors report: "Those who were followed up had a similar distribution of sex, rank and Service to those who were not, and to the Armed Forces as a whole, but those who were followed up were slightly older at baseline." | The study’s first panel includes 77,047 participants enrolled from 2001–2003 (36% of those able to be contacted). A second panel was enrolled from 2004–2006 and consisted of 31,110 participants (25% of those able to be contacted). Of Panel 1 participants, 71% responded to the 2004 and 2007 follow-up surveys, and 55% of Panel 2 participants responded to the 2007 follow-up survey. The present study included members of the first and second panels who completed a baseline and at least one follow-up questionnaire (n=80,524). Of the 80,524 participants with at least one follow-up assessment, 13832 (17%) were eligible (separated). Of those 588 (4%), 450 3(%), 1729 (13%) and 1975 (14%) were deleted due to being homemaker (primarily young married women), missing outcome, missing covariate and being reserve/guard. |
| **Reporting Bias** | 1 | 1 |
| Description |  |  |
| **Other Bias** | 2 | 1 |
| Description | Recall bias of combat exposures and deployments. |  |
| **Apriori Protocol** | No | Unclear |
| Description | "In 2002 we surveyed alcohol ... Use in the UK Armed Forces. Roughly 3 years later a second survey of the same cohort was carried out. In the interim a number of the participants went on deployments …" (p.1067). Cohort originally selected to test willingness of servicemen to screen. | They call it an "explorative study" p.410. |
| **Apriori Plan of Analysis** | Unclear | Unclear |
| Description | Probably yes |  |
| **Confounding** | 4 | 5 |
| Description | The measure before and after in both treat and control and analyse changes. No correction for previous mental health, ethnicity, previous deployments. Not clear whether they have reservists in their sample (probably not). Do not report on imbalances. | A lot of the covariates are most likely measured post deployment (using the survey closest to separation), among them several mental health diagnoses and disabling illness and injury. Main issue seems to be that we know deployment affects mental health which in turn affects civilian employment. This is also visible from the raw associations in the paper (table 1). Their specification includes mental health status, so the effect of deployment which they measure is conditional on mental health status. |
| Relevant confounders described by researchers? | No | No |
| Assessment of relevant confounders | None presented. Effects are adjusted for age, sex, Service and rank, imbalances not reported. | Do not consider number of previous deployments. A lot of the covariates are most likely measured post deployment (using the survey closest to separation), among them several mental health diagnoses. No imbalances shown or discussed. |
| **Method**  **(Design Stage)** | None | None |
| **Method**  **(Analysis Stage)** | OLS on changes in consumption | Logistic regression |

| **Author** | Hoyt T., Renshaw K. D., | Iowa Persian Gulf Study Group | Ishoy T., Suadicani P., Andersson A. M., Guldager B., Appleyard M., SkakkebÇÝk N. E., Gyntelberg F. |
| --- | --- | --- | --- |
| **Year** | 2014 | 1997 | 2001 |
| **Sequence Generation** | High | High | High |
| **Allocation Concealment** | High | High | High |
| **Blinding** | 4 | 4 | 4 |
| Description |  | Telephone survey | Non-blind |
| **Incomplete Outcome Data** | 4 | 2 | 3 |
| Description | Response rate 37%. 1 of 82 with missing data / n=490 approached (relevant population), 271 agreed to participate (55%), 49 of 271 excluded for not doing service in OEF/OIF, out of 222 with valid time 1 response, 140 did not respond at time 2 + 1 incomplete response. Leaving 81 valid responses for analysis. | However discussion on p. 244: "differential participation by selected demographic subgroups, although relatively minor, may limit our ability to generalize to other populations. However, given the high response rate and participation rate, nonresponse bias is unlikely to explain the observed differences." Response rate 76%. | From #1103: Response rate for treated: 83.6%, controls: 57.7%. "It is a well-established experience that refusals/non-responders in population-based follow-up studies are characterized by an excess psychiatric morbidity rate. According to the literature on attrition in population-based studies most frequent diagnoses in this connection are antisocial personality disorder and alcoholism (20, 21). In the present study we found a participation rate of approx. 84% of the veterans selected for the follow-up study, indicating that the main results must be regarded as representative for the entire group. The relatively lower participation rate (approx. 58%) among the not- deployed control individuals does not appear to have significant impact on the main results, since their lifestyle characteristics, drug and alcohol pattern and other background variables were not significantly different from those of the veterans except for minimal differences in use of medicine". |
| **Reporting Bias** | 1 | 1 | 1 |
| Description |  | Probably yes |  |
| **Other Bias** | 1 | 2 | 1 |
| Description |  | Recall bias, multiple comparisons. |  |
| **Apriori Protocol** | Unclear | Yes | Unclear |
| Description |  | See Doebbeling et al., 2002. |  |
| **Apriori Plan of Analysis** | Unclear | Unclear | Unclear |
| Description |  | "Prior to the study, a 2-tailed value was established at .05." (p.240). '"The criteria for these definitions were developed by the study investigators prior to the beginning of the data analysis. Most of the medical and psychiatric conditions were defined based on answers to multiple questions and using accepted criteria from standardized instruments and the medical literature." (p.239) |  |
| **Confounding** | 4 | 3 | 5 |
| Description | Guards and reserves only. Excluded those with prior deployment. 98% men. Otherwise nothing controlled for. | "Cochran-Mantel-Haenszel rate difference estimates of each of the primary end points were analysed for the 4 major comparisons, controlling for the stratification variables" (p.240). From 10365: Only minor imbalances on stratification variables and precision fine. | Gender, age and professionally (?) matched control group ("… personnel database of the Danish Armed Forces comprising personnel employed according to contract who could have been - but had not - been deployed in the Gulf", p. 46). No control for confounding. In addition there is a high risk of compositional confounding. Sample were deployed for 6 months between 1990-1997. |
| Relevant confounders described by researchers? | No | No | No |
| Assessment of relevant confounders | Enlistment status, gender and prior deployment are the same for all. Mental health history, age, ethnicity, rank and branch not considered. | All but mental health history and previous deployments. Nothing recorded in the paper on balance and measurement. Have to refer to other reports from same group From 10365: Only minor imbalances on stratification variables and precision fine. | Incomplete match on age, gender, profession. |
| **Method**  **(Design Stage)** | Exclude those with prior deployment and use Guards/Reserves only. | Stratified sampling Within the four domains Active GW deployed, Active non-deployed, Guard/Reserve GW deployed and Guard/Reserve non-deployed a random stratified (on Branch of service, rank, gender, race and age) sample were drawn. | Matched sampling |
| **Method**  **(Analysis Stage)** | Nothing we can use | CMH Cochran-Mantel-Haenszel adjusted rate differences. | None (for our outcomes, logistic regression for their main outcomes) |

| **Author** | Ishøy, Knop, Suadicani, Guldager, Appplyard, Gyntelberg |
| --- | --- |
| **Year** | 2004 |
| **Sequence Generation** | High |
| **Allocation Concealment** | High |
| **Blinding** | 4 |
| Description |  |
| **Incomplete Outcome Data** | 3 |
| Description | Response rate for treated: 83.6%, controls: 57.7%. "It is a well-established experience that refusals/non-responders in population-based follow-up studies are characterized by an excess psychiatric morbidity rate. According to the literature on attrition in population-based studies most frequent diagnoses in this connection are antisocial personality disorder and alcoholism (20, 21). In the present study we found a participation rate of approx. 84% of the veterans selected for the follow-up study, indicating that the main results must be regarded as representative for the entire group. The relatively lower participation rate (approx. 58%) among the not- deployed control individuals does not appear to have significant impact on the main results, since their lifestyle characteristics, drug and alcohol pattern and other background variables were not significantly different from those of the veterans except for minimal differences in use of medicine". |
| **Reporting Bias** | 1 |
| Description | No protocol, hard to judge. |
| **Other Bias** | 1 |
| Description | Data confidential |
| **Apriori Protocol** | Unclear |
| Description | Probably no |
| **Apriori Plan of Analysis** | Unclear |
| Description | They use logistic regression in analyzing main outcomes of interest, but use backward elimination. |
| **Confounding** | 5 |
| Description | Match on age, gender, profession (unclear what profession is). |
| Relevant confounders described by researchers? | No |
| Assessment of relevant confounders | incomplete match on age, gender, profession |
| **Method**  **(Design Stage)** | Matched sampling |
| **Method**  **(Analysis Stage)** | None (for our outcomes, logistic regression for their main outcomes) |

| **Author** | Ismail, K., Kent, K., Brugha, T., Hotopf, M., Hull, L., Seed, P. et al. | Iversen A. C., van S. L., Hughes J. H., Browne T., Hull L., Hall J., Greenberg N., Rona RJ., Hotopf M., Wessely S., Fear N. T. | Jacobson I.G., Horton J. L., LeardMann C. A., Ryan M. A. K., Boyko E. J., Wells T. S., Smith B., Smith T. C. |
| --- | --- | --- | --- |
| **Year** | 2002 | 2009 | 2012 |
| **Sequence Generation** | High | High | High |
| **Allocation Concealment** | High | High | High |
| **Blinding** | 4 | 4 | 4 |
| Description |  | Non-blind |  |
| **Incomplete Outcome Data** | 4 | 2 | Unclear |
| Description | Study population is those that returned completed questionnaires from the phase 1 of the British Gulf War Study. Randomly select disabled. Response rate for deployed 67%. Response rate for Era comparison 43% and for Bosnia comparison 55%. Note response rate at Phase 1, 60-70%. (p.2) | Adjusted response-rate of 76%. However that is based on a sample from another study with response rate of 61%. They do a comparison of non-responders on p.5. | The study’s first panel includes 77,047 participants enrolled from 2001–2003 (36% of those able to be contacted). A second panel was enrolled from 2004–2006 and consisted of 31,110 participants (25% of those able to be contacted). Of Panel 1 participants, 71% responded to the 2004 and 2007 follow-up surveys, and 55% of Panel 2 participants responded to the 2007 follow-up survey. The present study included members of the first and second panels who completed a baseline and at least one follow-up questionnaire (n = 80,524). Of the 80,524 participants with at least one follow-up assessment, 4,285 (5%) were in the Marine Corps and ineligible because Navy personnel assume all health care positions for this service branch, 3,197 (4%) were missing demographic or covariate data, 3,849 (5%) were missing PTSD or depression outcome data, and 4,085 (5%) screened positive for either PTSD or depression at baseline, leaving 65,108 individuals for descriptive analysis. Further restrict to health care personnel deployed, 1,492 individuals. |
| **Reporting Bias** | 1 | 3 | 1 |
| Description |  | No adjusted results presented for common mental disorder and alcohol abuse. Text is not clear on what confounders are adjusted for (footnote, table 3). |  |
| **Other Bias** | 1 | 2 | 1 |
| Description |  | Authors note that responders may have been concerned that superiors learn their responses leading to underreporting of symptoms. |  |
| **Apriori Protocol** | Unclear | Yes | Unclear |
| Description |  | Page 4 | Prospective data collection |
| **Apriori Plan of Analysis** | Unclear |  | Unclear |
| Description |  | Not explicitly stated |  |
| **Confounding** | 5 | 5 | 4 |
| Description | Only control for gender, age and rank and comparison is Era veterans and Bosnia (deployed 1-5 years later) combined. | No discussion of balance. Adjusted for: "status" (Likely regular/reserve), previous deployments, educational attainment, vulnerability factors and Service. | No imbalances shown or mentioned (the relevant treatment/comparison for us). Control for post deployment outcomes (marital status, smoking, trouble sleeping and alcohol related problems). |
| Relevant confounders described by researchers? | No | Partly on p.4 | No |
| Assessment of relevant confounders | Only age, sex, rank, and marital status using probability weights. Phase 1 disabled only. See sheet as well. | Controls for status ("pre" regular/reserve), previous deployments, educational attainment, vulnerability factors, service (branch). | All and more is added (mostly post deployment outcomes), do not show or mention imbalances on the (for us) relevant treatment/comparison. |
| **Method**  **(Design Stage)** | Logistic regression | None (sampling scheme in place but not to address confound relevant to this review). "We used a 'two-phase survey' technique to identify the prevalence of psychiatric diagnoses in the whole KCMHR military health study sample. Possible psychiatric cases were identified from the main cohort using the 12-item General Health Questionnaire (GHQ). A random sample of those who scored above the threshold for 'GHQ caseness' (score ≥ 3) were selected for interview together with a random sample of the non-GHQ cases. Cases were over-sampled; 70% of the final samples for the study were GHQ cases, and 30% were non-GHQ cases. We also included all participants who scored ≥ 50 on the Post Traumatic Stress Disorder Checklist (PCL)" (Page 2). | None |
| **Method**  **(Analysis Stage)** | Logistic regression | Logistic regression | General estimating equations |

| **Author** | Jacobson I., Horton J., LeardMann C., Ryan M., Boyko E., Wells T., Smith B. | James L. M., Van K. E., Miller R. D., Engdahl B. E. | Kang H. K., Li B., Mahan C. M., Eisen S. A., Engel C. C. |
| --- | --- | --- | --- |
| **Year** | 2012 | 2013 | 2009 |
| **Sequence Generation** | High | High | High |
| **Allocation Concealment** | High | High | High |
| **Blinding** | 2 | 4 | 4 |
| Description | Prospective study. Analysts did not engage in data collection. |  | Non-blind |
| **Incomplete Outcome Data** | Unclear | Unclear | 4 |
| Description | Not described for this particular subsample of health care professionals. | Unclear at time 1, response rate 52% at time 2 and 36% at time 3. | Response rate to follow-up 34% |
| **Reporting Bias** | 1 | 1 | 2 |
| Description |  |  | Unclear procedure for estimating adjusted relative risk. |
| **Other Bias** | 1 | 1 | 1 |
| Description |  |  |  |
| **Apriori Protocol** | Yes | Yes | Unclear |
| Description | Millenium Cohort Study | "The study protocol was approved by the local IRB" (p 160). | Not stated |
| **Apriori Plan of Analysis** | Unclear | Unclear | Unclear |
| Description | Not explicitly stated |  | Not stated |
| **Confounding** | 4 | 5 | 4 |
| Description | Overcontrolling. Controls for time varying covariates: smoking status, trouble sleeping, alcohol-related problems. Does not discuss distributional overlap exposed/non-exposed. | Pre deployment life events controlled for. Otherwise only post deployment neuroticism and post deployment social support 'controlled' for. 85% male, age 19-58, 68% caucasian (28% did not reply), mix of combat roles, mix of enlistment. 85% army. | Propensity score matching (p.404). Procedure not clearly described, ex: "A large set of background variables …." Nor is distributional overlap asserted. Some confounding variables set at the 2005 level. |
| Relevant confounders described by researchers? | Yes, table 1, p.619-620. | None | Yes, p.404 |
| Assessment of relevant confounders | All listed confounders controlled for. And more of which some are post deployment variables. | Pre deployment life events controlled for. Otherwise only post deployment neuroticism and social support 'controlled' for. | Some confounders assessed at 2005 level, including BMI (which is potentially problematic). Confounders controlled for: age, gender, race, rank, branch, enlistment status, BMI, current smoking status. Some imbalance on ethnicity, rank and enlistment status. Not considered: mental health history, prior deployment. |
| **Method**  **(Design Stage)** | None | None | None |
| **Method**  **(Analysis Stage)** | General estimating equations | Regression | The exact method is uncertain. Semi-parametric propensity score from logistic regression used as stratification variable in calculating relative risk using the Mantel-Haenszel method and maybe further adjustment of relative risks. |

| **Author** | Kang, H. K., Mahan, C. M., Lee, K. Y., Magee, C. A., & Murphy, F. M. | Kelley M. L., Runnals J., Pearson M. R., Miller M., Fairbank J. A., Brancu M. | Kelsall, H. L., Sim, M. R., Forbes, A. B., Glass, D. C., McKenzie, D. P., Ikin, J. F. et al. |
| --- | --- | --- | --- |
| **Year** | 2000 | 2013 | 2004 |
| **Sequence Generation** | High | High | High |
| **Allocation Concealment** | High | High | High |
| **Blinding** | 4 | 4 | 3 |
| Description |  |  | HSA doctor making medical assessment was blind to deployed non-deployed, p 1007. |
| **Incomplete Outcome Data** | 2 | 2 | 3 |
| Description | 15,000 deployed and 15,000 non-deployed solicited to participate. Of these 11,441 (76% Own calculations they report 75%) deployed and 9,476 (63% Own calculations they report 64%) non-deployed veterans responded. Non-responders were more likely to be younger, unmarried, non-white who had served in enlisted ranks than responders. No difference in self-perceived exposure between phase 1 responders and phase 2 (Telephone interview to increase response rates) responders. No mentioning of missing data. | Same data as 24518 where it is stated: Of 1899 participants, 230 (+2) participants had missing values (12%). In this study it is stated that data were available on 1825 participants. Due to the recruitment procedure response rate is not relevant to consider. | Partially addressed. Sensitivity analysis carried out on smaller questionnaire. Participation rate T: 80.5% and C: 56.8%. Response sensitivity analysis carried out, table 7, p.123 (in 1159). Only minor differences (2.5-5%) and in this paper also (on other outcomes than in 1159) showing only marginal differences (page 1008). Missing data rate T: 0.7% C: 8.9%. |
| **Reporting Bias** | 4 | 3 | 1 |
| Description | Do not show or mention imbalances. | They examined age and marital status as covariates in the models and found that controlling for these variables had no substantive effect on the results. Results not shown. | Probably yes |
| **Other Bias** | 2 | 1 | 1 |
| Description | Self-reported outcome variables subject to recall bias. |  |  |
| **Apriori Protocol** | Unclear | No | No |
| Description |  | "The original study was approved …" (p.616). Since secondary data analyses data generating process is irrelevant, and analysis covered by other item. | Retrospective |
| **Apriori Plan of Analysis** | Unclear | Unclear | Unclear |
| Description |  | Not specified | Probably yes |
| **Confounding** | 4 | 4 | 1 |
| Description | Stratify on gender and duty type. Non-deployed further stratified on branch to mirror the number in the same stratum of deployed. Nothing else is done or reported. | Only gender and non-combat traumatic events controlled for (could proxy for mental health history, indeed control for prior trauma). | Likely yes |
| Relevant confounders described by researchers? | No | No | 1 |
| Assessment of relevant confounders | Only gender and duty type via stratification. Non-deployed further stratified on branch to mirror the number in the same stratum of deployed. No imbalances showed or mentioned. | Not considered: Mental health, rank, branch, enlistment, age, ethnicity and previous deployment. Gender and non-combat trauma events before, during and after deployment controlled for. No imbalances on CE shown or discussed. | None |
| **Method**  **(Design Stage)** | The sampling design is a stratified random sample with unequal probabilities of selection within combinations of the strata: deployment status, gender and duty type (active service v. reserve or National Guard). Non-deployed further stratified on branch to mirror the number in the same stratum of deployed. | None | Retrospective study |
| **Method**  **(Analysis Stage)** | None | Structural equation model |  |

| **Author** | Lane M. E., Hourani L. L., Bray R. M., Williams J. | Larson, G. E., Highfill-McRoy, R. M., & Booth-Kewley, S. | LeardMann C. A., Smith T. C., Smith B., Wells T. S., Ryan M. A. K. |
| --- | --- | --- | --- |
| **Year** | 2012 | 2008 | 2009 |
| **Sequence Generation** | High | High | High |
| **Allocation Concealment** | High | High | High |
| **Blinding** | 4 | 3 | 4 |
| Description | Non-blind | Administrative data, participants were blind to the study, but obviously not to deployment. |  |
| **Incomplete Outcome Data** | 2 | 1 | 2 |
| Description | Response rate of 55.3% and 51.8% for active and reserve. | Administrative data. Complete cohort analysed. | Baseline response rate 36%, follow-up response rate 71%. Only include deployed with combat exposure and further exclude those who reported a previous physician diagnosis of PTSD or who had symptoms of PTSD at baseline, who self-reported (at follow-up) being diagnosed with PTSD before deployment (n=11) and those with missing baseline or follow-up PTSD, functional health, or covariate data (7%). "Analysis of potential responder bias to the first follow-up are ongoing" (p2) |
| **Reporting Bias** | 1 | 1 | 2 |
| Description |  |  | Do not show imbalances other than by functional status (which is the objective of the paper to analyse). |
| **Other Bias** | 1 | 2 | 2 |
| Description | Anonymous self-reports (p.1214) | Participants may avoid using TRICARE, and this may vary between treat and control particularly with regards to PTSD. | Possibly recall bias. Questionnaire measures combat exposure as self-reported exposure to witnessing death, abuse, maimed soldiers or civilians, prisoners of war, or refugees, in the past 3 years. |
| **Apriori Protocol** | Yes | Unclear |  |
| Description | IRB approval (p1213) | Since they sample an entire cohort this may be less of an issue. "We conducted a prospective study with data on both pre- and post-deployment use of psychiatric health-care services …" | Approved by the institutional review board, Naval Health Research Center, San Diego, CA (Protocol NHRC.2000.007). |
| **Apriori Plan of Analysis** | Unclear | Unclear | Unclear |
| Description | Not explicitly stated | Probably yes |  |
| **Confounding** | 4 | 4 | 2 |
| Description | No demonstration of balance or that deployment is exogenous to participants. | They have "history of mental health" but does not control for it (instead they drop observations from treatment only and this analysis shows that mental history matters except for PTSD). | All confounders (and more) controlled for but imbalances not reported. Unclear when Marital status is measured (pre/post). |
| Relevant confounders described by researchers? | Yes, table 1, p.1215 | No | Discussion on page 5-7 |
| Assessment of relevant confounders | Adjusted for: gender, race, education, age, marital status, pay grade (rank), and service. | Adjust for gender and race (dichotomous) only. Marines only, reservists excluded, they have age but does not control for it (appears balanced), they have "history of mental health" but does not control for it. (Instead they drop observations from treatment only. NB we use another estimate.) | All and more is added. Relevant (for us), imbalances not shown. |
| **Method**  **(Design Stage)** | None | Marine deployed sample to Marine non-deployed (crude). | Population based survey (Millenium Cohort) |
| **Method**  **(Analysis Stage)** | They use the predicted marginal approach. | Adjusted relative risk | Logistic regression |

| **Author** | Li, Mahan, Kang, Eisen & Engel | Lindem, K., Heeren, T., White, R. F., Proctor, S. P., Krengel, M., Vasterling, J. et al. | Litz, B. T., King, L. A., King, D. W., Orsillo, S. M., & Friedman, M. J. |
| --- | --- | --- | --- |
| **Year** | 2011 | 2003 | 1997 |
| **Sequence Generation** | High | High | High |
| **Allocation Concealment** | High | High | High |
| **Blinding** | 4 | 4 | 4 |
| Description |  |  |  |
| **Incomplete Outcome Data** | 2 | 3 | Unclear |
| Description | For the baseline survey in 1995, 11,441 deployed veterans (76%) and 9,476 non-deployed veterans (63%) participated. In 2005 6,111 (40%) deployed and 3,859 non-deployed (27%) responded (of the 29,607 living panel members). The most common reasons for nonparticipation in the postal survey were that people did not remember receiving the survey (32% of non-respondents); were not interested in participating in the survey (21%); intended to complete the survey (16%); did complete the survey and mail it back (7%); gave no particular reason for not completing the survey (7%); felt the survey did not apply to them (5%); were deployed or out of town (4%); and thought the survey was too long and involved (3%). The group of 8,822 veterans who participated in both surveys were representative of the permanent panel of 30,000 veterans in terms of demographic and military characteristics: gender, age, race, marital status, rank, branch, and unit component. | Treated: subsample of the Devens cohort (random stratified sampling based on unit designation, health symptom reporting, and gender). Original sample response rate was 79% (n: 2330). Random sampling yielded 353 veterans, of whom 261 (73.9%) could be contacted and 220 (84.3%) participated. Further reduced to 141 due to residence outside of commuting distance to the testing site. New Orleans Cohort: subsample of the original of 928. A random sample (method similar to above) of 194 veterans of whom 125 (64.4%) could be contacted. 73 (58% of those contacted) were seen for assessment, and 51 veterans participated and 37 completed the PTSD Scale. Comparison: Germany-Deployed GW-Era Cohort, 50 participants (85%) of those who could be located; 51% of the deployed unit were tested in the spring of 1995. 47 completed. | Unclear, it is stated: Data for this study were taken from Litz et ai.'s (1997) Somalia veteran survey. Of these, 60% was used for specifying and evaluating a structural model (n = 1,650). |
| **Reporting Bias** | 2 | Unclear | 1 |
| Description | They applied propensity score methods to reduce 8 covariates to a quintile (not reported which ones, they have 9 covariates and use 8 in the analysis). |  |  |
| **Other Bias** | 3 | 1 | 1 |
| Description | Gulf War onset outcomes may be biased due to recall bias as they are diagnosed 8-10 years after. |  |  |
| **Apriori Protocol** | Unclear | Unclear | Unclear |
| Description |  |  |  |
| **Apriori Plan of Analysis** | Unclear | Unclear | Unclear |
| Description |  |  |  |
| **Confounding** | 4 | 5 | 5 |
| Description | Two important confounders not considered and two post deployment outcomes (smoking and body mass index) controlled for. None or only small imbalances. | Nothing | Structural equation model with traditional combat, pressure to uphold restraint, positive aspects of peacekeeping and negative aspects of peacekeeping on PTSD. |
| Relevant confounders described by researchers? | No | No | No |
| Assessment of relevant confounders | All except mental health history and number of deployments. Current smoking and body mass index added. | Gender, age, ethnicity and Number of previous deployments are considered but no adjustment and large imbalances. | None |
| **Method**  **(Design Stage)** | From 11941: The sampling design is a stratified random sample with unequal probabilities of selection within combinations of the strata: deployment status, gender and duty type (active service v. reserve or National Guard). | Random sampling from original samples of the treated. | None |
| **Method**  **(Analysis Stage)** | Adjusted risk ratios through the Mantel-Haenszel method applying propensity score methods. | None | Structural equation model |

| **Author** | Litz B. T., Orsillo S. M., Friedman M., Ehlich P., Batres A. | Luxton D. D., Skopp N. A.,Maguen S. | MacGregor, Heltemes, Clouser, Han & Gaiarneau |
| --- | --- | --- | --- |
| **Year** | 1997 | 2010 | 2014 |
| **Sequence Generation** | High | High | High |
| **Allocation Concealment** | High | High | High |
| **Blinding** | 4 | 4 | 4 |
| Description | Non-blind | Non-blind |  |
| **Incomplete Outcome Data** | Unclear | 1 | 1 |
| Description | Only number of completed surveys reported, not number eligible for survey. | It would appear they have complete data; at least post is mandatory assessment. | Missing data level very low (12-60 of 3512) (5165 initial sample - 514 with prior MH diagnosis - 1110 more than 2 deployments - 39 women = 3502, using info on p.382). |
| **Reporting Bias** | 1 | 1 | 1 |
| Description |  |  |  |
| **Other Bias** | 1 | 2 | 1 |
| Description |  | Mandatory post-deployment questionnaires that will be used for medical assessment which may affect career paths. |  |
| **Apriori Protocol** | Unclear | Yes | Unclear |
| Description |  | Yes, p 1029. | This study was approved by the Institutional Review Board at Naval Health Research Center, San Diego, California. |
| **Apriori Plan of Analysis** | Unclear | Unclear | Unclear |
| Description |  | Not explicitly stated |  |
| **Confounding** | 4 | 5 | 3 |
| Description | No discussion regarding random assignment of exposure. | Over-controlling. Controls for PTSD score in Depression equation and vice versa. At least for our purpose this renders the interpretation of the estimates unusable. Have access to a host of demographics: age, gender, education, rank (see p.1029) but only controls for gender. | All except ethnicity controlled for. No imbalances shown or discussed. Sample restricted to: 2 times deployers, Marines, males, no prior mental health diagnosis. |
| Relevant confounders described by researchers? | Yes, but not all. P.181, see also table 1, p.180. | Yes, p.1029 | None |
| Assessment of relevant confounders | Confounders included: gender, ethnicity, prior deployments, age, education and only include active duty. Imbalances not reported. | Control for gender, prior mental health, and number of deployments. Include active duty Army soldiers only. | All except ethnicity considered and controlled for + more. In addition an interaction term of combat exposure and dwell to deployment ratio (DDR). No imbalances shown or discussed. |
| **Method**  **(Design Stage)** | None | None | Restrict sample to: Male Active duty Marines with two deployments between 2005 and 2008 to Afghanistan, Kuwait or Iraq with no mental health diagnosis before second deployment and all exposed to combat during second deployment. |
| **Method**  **(Analysis Stage)** | OLS | OLS | Logistic regression |

| **Author** | Maguen S., Litz B. T., Wang J. L., Cook M. | Maguen, S., Lucenko, B. A., Reger, M. A., Gahm, G. A., Litz, B. T., Seal, K. H. et al. | Marx, B. P., Doron-Lamarca, S., Proctor, S. P., & Vasterling, J. J. |
| --- | --- | --- | --- |
| **Year** | 2004 | 2010 | 2009 |
| **Sequence Generation** | High | High | High |
| **Allocation Concealment** | High | High | High |
| **Blinding** | 4 | 4 | 4 |
| Description |  |  |  |
| **Incomplete Outcome Data** | 3 | 2 | 2 |
| Description | Pre-deployment 1,132 agreed to participate (number of refusals not known) and 324 agreed to participate post-deployment. Were able to contact 203 post-deployment. On page 200 it is stated: Those soldiers, who completed the post-deployment survey tended to be slightly older, married, slightly more educated, and somewhat higher in rank. There were no statistically significant group differences in regards to sex and the number of previous deployments. | Missing data on race/ethnicity and mental health symptom variables in 7%. "All participants who returned from OIF deployments were eligible for participation; no information is available on rates of refusal." (p 88) | Participation rate 67%, table 1 page 844, compares responders with non-responders, less women and prior deployed in responders, otherwise only very minor differences. |
| **Reporting Bias** | 3 | 1 | 1 |
| Description | State that a number of demographic and military variables were correlated with the outcome variables. None of the correlations were significant and were therefore not included regression equation. (p. 204) Relevant data not shown. |  |  |
| **Other Bias** | 1 | 1 | 1 |
| Description |  |  |  |
| **Apriori Protocol** | Yes | Unclear | Unclear |
| Description | The Veterans Affairs Boston Institutional Review Board approved all procedures and materials. (p. 1999) |  |  |
| **Apriori Plan of Analysis** | Unclear | Unclear | Unclear |
| Description |  |  |  |
| **Confounding** | 5 | 4 | 5 |
| Description | An Army active-duty sample, otherwise only mental health history controlled for and in addition 4 (too subjective measures and correlated with outcome) appraisal and exposure variables included. Age, sex, rank, previous deployments (and marital status, education) all entered in regression step, but does not enter in final model. Criteria for eliminating confounders not clearly specified. | Have access to other variables they do not use. | Army active duty only sample, control for age and gender and include 5 different post-deployment neurocognitive performance measures. |
| Relevant confounders described by researchers? | No | No | No |
| Assessment of relevant confounders | All except ethnicity is considered. No imbalances shown or discussed. Only pre-deployment mental health (alcohol for alcohol outcome, depression for depression outcome and PTSD for PTSD outcome) controlled for. In addition 4 (post morale entered as indicated from table 7 p. 204) appraisal and exposure variables (positive military experiences, general overseas military stressors, negative aspects of peacekeeping and morale) included in regression. | Gender, age, ethnicity and three others. No reporting of imbalances. | Army active duty only sample, control for age and gender and include 5 different post-deployment neurocognitive performance measures. |
| **Method**  **(Design Stage)** | None | None | Unclear |
| **Method**  **(Analysis Stage)** | Hierarchical regression | Multiple regression | Multiple linear regression |

| **Author** | McKenzie, D. P., Ikin, J. F., McFarlane, A. C., Creamer, M., Forbes, A. B., Kelsall, H. L. et al. | Pierce M. D., Wood M. D., Reddy M., Sevin E., Shea M. T., |
| --- | --- | --- |
| **Year** | 2004 | 2012 |
| **Sequence Generation** | High | High |
| **Allocation Concealment** | High | High |
| **Blinding** | 4 | 4 |
| Description |  |  |
| **Incomplete Outcome Data** | 2 | 3 |
| Description | Here nothing reported in text. From 928: Participation rate T: 80.5% and C: 56.8%. Response sensitivity analysis carried out, table 7, p.123 (in 1159) and page 1008 in 1123. Only minor differences (0.3-5%). Here it is reported: Females excluded and among the males missing data rates: MCS: T: 3.5% C: 2.3%, GHQ: T: 0.1% C: 0.3%, PTSD: T: 6% C: 6.2%. | "The researchers were able to present this study to about 67% of the military personnel returning from the units approached with 66% of those hearing about the study agreeing to be contacted and 70% of those personnel contacted agreeing to participate." (page 14) The meaning of this sentence is a bit unclear. |
| **Reporting Bias** | 1 | 3 |
| Description | Likely yes | Does not adequately report when measurement took place and when deployment had occurred. |
| **Other Bias** | 1 | 2 |
| Description |  | Very unclear whether participants were deployed more than once before the first assessment. |
| **Apriori Protocol** | No | Unclear |
| Description | Retrospective study |  |
| **Apriori Plan of Analysis** | Unclear | Unclear |
| Description | Probably yes. Simple analysis with adjustment for confounders. |  |
| **Confounding** | 3 | 5 |
| Description | Comparison sample is matched on age, rank, gender, branch, but minor imbalances on age and rank and medium imbalance on branch (see Ikin et al. 2004). In addition estimates are controlled for marital status and education. Sample turns out to be males only. Not adjusted for duty status, previous mental health From 1159 minor imbalance), number of deployments (from 1159 medium imbalance). | Only Guards included in analysis, otherwise nothing is considered. |
| Relevant confounders described by researchers? | No | No |
| Assessment of relevant confounders | Not described here (descibed in Ikin et al 2004). From 1159 all except duty/enlistment status described. | Only National Guard troops included, otherwise nothing is considered. |
| **Method**  **(Design Stage)** | Matched sampling | None |
| **Method**  **(Analysis Stage)** | OLS, logistic regression | None |

| **Author** | Pietrzak R. H., Johnson D. C., Goldstein M. B., Malley J. C., Rivers A. J., Morgan C. A., Southwick S. M. | Polusny M. A., Kumpula M. J., Meis L. A., Erbes C. R., Arbisi P. A., Murdoch M., Thuras P., Kehle-Forbes S. M., Johnson A. K. | Proctor, S. P., Harley, R., Wolfe, J., Heeren, T., & White, R. F. |
| --- | --- | --- | --- |
| **Year** | 2010 | 2014 | 2001 |
| **Sequence Generation** | High | High | High |
| **Allocation Concealment** | High | High | High |
| **Blinding** | 4 |  | 4 |
| Description | Non-blind |  |  |
| **Incomplete Outcome Data** | 4 | 3 | 3 |
| Description | 1000 Vets surveyed (starting alphabetically), 28.5% responded. | Response rate post deployment 70%. Some difference between responders/non but not on gender, rank and baseline PTSD symptoms. | Treated: Subsample of the Devens cohort (random stratified sampling based on unit designation, health symptom reporting, and gender). Original sample response rate was 79% (n: 2330). Random sampling yielded 353 veterans, of whom 261 (73.9%) could be contacted and 220 (84.3%) participated. Further reduced to 141 due to residence outside of commuting distance to the testing site. Comparison: Germany-Deployed GW-Era Cohort, 50 participants (85%) of those who could be located; 51% of the deployed unit) were tested in the spring of 1995. 47 completed. |
| **Reporting Bias** | 1 | 1 | 1 |
| Description |  |  |  |
| **Other Bias** | Unclear | 1 | 1 |
| Description | No personal identifying information was made available to the authors, but not stated whether VA had access. |  |  |
| **Apriori Protocol** | Unclear | Yes | Unclear |
| Description |  | Page 3 |  |
| **Apriori Plan of Analysis** | Unclear | Unclear | Unclear |
| Description |  | Not stated |  |
| **Confounding** | 5 | 2 | 5 |
| Description | No corrections for confounders. Some demographics available (p.189) but not presented or controlled for. | Not considered: ethnicity and rank. Imbalances not shown or discussed. Model for combat exposure (aftermath battle exposure) also controls for aftermath of battle exposure (combat exposure) and exposure to deployment sexual stressors. | Only consider age, gender, ethnicity and service in Vietnam, large imbalances on the last two and do not control for anything. |
| Relevant confounders described by researchers? | No | No, only relating to gender differences. | No |
| Assessment of relevant confounders | None considered | Not considered: ethnicity and rank. Considered (controlled for): gender, age, marital status, prior OEF/OIF deployment status, brigade cohort, baseline symptoms (Time 1 PTSD (PCL-C)), pre-deployment risk factors (prior interpersonal victimization, unit support, preparedness, and life/family concerns. No relevant imbalances shown. Model for combat exposure (aftermath battle exposure) also controls for aftermath of battle exposure (combat exposure) and exposure to deployment sexual stressors. | Only Army included. Gender, age, ethnicity and Number of previous deployments are considered but no adjustment and large imbalances. |
| **Method**  **(Design Stage)** | None | None | Random sampling from original samples of the treated. |
| **Method**  **(Analysis Stage)** | None | Multiple regression | None |

| **Author** | Proctor, S. P., Heaton, K. J., White, R. F., & Wolfe, J. | Proctor, S. P., Heeren, T., White, R. F., Wolfe, J., Borgos, M. S., Davis, J. D. et al. | Renshaw K. D. |
| --- | --- | --- | --- |
| **Year** | 2001 | 1998 | 2011 |
| **Sequence Generation** | High | High | High |
| **Allocation Concealment** | High | High | High |
| **Blinding** | 4 | 4 | 4 |
| Description |  | Questionnaire | Non-blind |
| **Incomplete Outcome Data** | 3 | 3 | 3 |
| Description | Probably no. Random subsample from Devens Cohort. Oversampling of symptomatic and women. No reports on non-responders. Within study CAPS/SCID indicates n=143 screened but total of T=180, and C: 46 (table 1, p262). From 6777: Treated: subsample of the Devens cohort (random stratified sampling based on unit designation, health symptom reporting, and gender). Original sample response rate was 79% (n: 2330). Random sampling yielded 353 veterans, of whom 261 (73.9%) could be contacted and 220 (84.3%) participated. Further reduced to 141 due to residence outside of commuting distance to the testing site. New Orleans Cohort: subsample of the original of 928. A random sample (method similar to above) of 194 veterans of whom 125 (64.4%) could be contacted. 73 (58% of those contacted) were seen for assessment, and 51 veterans participated and 37 completed the PTSD Scale. Comparison: Germany-Deployed GW-Era Cohort, 50 participants (85%) of those who could be located; 51% of the deployed unit) were tested in the spring of 1995. 47 completed. | "The 186 Devens subjects who completed the questionnaires differed from the 353 target subjects in the sample frame in that they were more likely to be female, white, older, better educated, and to have reported >5 symptoms on the 1992/1993 survey than we would expect from the stratified random sampling. They did not differ in employment status, marital status, alcohol or drug use, or military service status (as assessed at the 1992/1993 survey). Similarly, the New Orleans cohort participants were more likely to be female and to have reported >5 symptoms on the initial survey. Thus weighting for the oversampling of females and participation bias was taken into account in the analyses comparing symptom rates across study groups." Some imputations exercise at best, since authors cannot know how responses would be. Bounding exercise would be a possibility, but not performed. | 490 troops attended, 270 participated, final sample: 207. |
| **Reporting Bias** | 1 | 2 | 1 |
| Description | Probably yes | Probably yes |  |
| **Other Bias** | 1 | 2 | 2 |
| Description | Probably yes | Recall bias | Possible recall bias (up to 7 years after exposure) |
| **Apriori Protocol** | Yes | unclear | Yes |
| Description | Probably yes qua sampling strategy. | Probably yes, as measurements taken in the 3 subsamples approximately same time. | Approved by Univ of Utah IRB (p 322). |
| **Apriori Plan of Analysis** | Unclear | Unclear | Unclear |
| Description |  | "Using a priori hypotheses about the toxicant effects of exposure to specific toxicants, the relationships between self-reported exposures and body-system symptom groupings were examined through multiple regression analyses." | Not stated |
| **Confounding** | 5 | 5 | 5 |
| Description | "Subjects in this study were 180 veterans from a larger cohort of military personnel deployed to the Gulf (Devens Cohort)". Gender and age are considered but no adjustment and large imbalance on age. | "All comparisons are weighted for sampling design, participation bias, and adjust for age, sex and education using SUDAAN." (p.1006) But very large imbalances. | No discussion of confounding, no control for confounding. |
| Relevant confounders described by researchers? | No | No | No |
| Assessment of relevant confounders | Missing confounders include: ethnicity, rank, gender, branch, reservist status, number of previous deployments. Age and gender is considered but not adjusted for, large imbalance on age. | Several confounders recorded (not rank, branch, enlistment type, mental health history) but only control for age, gender and education, not controlled for. Means/proportions tests suggest lack of overlap of covariates. | No (no sheet) |
| **Method**  **(Design Stage)** | Matched sampling | Random stratified sampling of original treated sample (but lack of overlap). | None |
| **Method**  **(Analysis Stage)** | None | SUDAAN Logistic regression | None |

| **Author** | Rona, R. J., Fear, N. T., Hull, L., & Wessely, S. | Rona, R. J., Hooper, R., Jones, M., Hull, L., Browne, Horne Murphy, D., Hotopf, and Wessely | Rona, R. J., Hooper, R., Jones, M., Iversen, A. C., Hull, L., Murphy, D. et al. |
| --- | --- | --- | --- |
| **Year** | 2007 | 2006 | 2009 |
| **Sequence Generation** | High | High | High |
| **Allocation Concealment** | High | High | High |
| **Blinding** | 4 | 4 |  |
| Description |  |  |  |
| **Incomplete Outcome Data** | Unclear | 3 |  |
| Description | Data taken from a larger sample: We selected for analysis all the women who completed a questionnaire in the Gulf and Iraq War Studies. | Table 2 p.2 compares responders and non-responders. Response rate Time 1 67%, response rate time 2 67%. Apparently no missing data. Only contacted 2820 out of 2873 possible without any explanation. |  |
| **Reporting Bias** | 4 | 1 |  |
| Description | Do not show or mention relevant imbalances. |  |  |
| **Other Bias** | 1 | 1 |  |
| Description |  |  |  |
| **Apriori Protocol** | No | Unclear |  |
| Description | Data collected as part of bigger study. | Probably not for the purpose we will use this study for. Main aim of article is to assess whether pre-screening predicts post-deployment symptoms. |  |
| **Apriori Plan of Analysis** | No | Unclear |  |
| Description |  |  |  |
| **Confounding** | 4 | 5 |  |
| Description | Compare with relevant era group. | Rather large imbalances (table 3) on branch, age, medically downgraded (description p.2) and gender. Nothing controlled for. |  |
| Relevant confounders described by researchers? | No | No |  |
| Assessment of relevant confounders | All, except ethnicity, mental history, and number of previous deployments and service only as stratification variable. Education and marital status is added. Do not show imbalances. | None |  |
| **Method**  **(Design Stage)** | Gulf War: The samples were stratified by Service [Royal Navy and Royal Marines (RN), Army, Royal Air Force (RAF)], age, enlistment type (regular or reserve), rank. Iraq War: Sampling was stratified by service and enlistment type. | None |  |
| **Method**  **(Analysis Stage)** | Logistic regression | None |  |

| **Author** | Rona R. J., Jones M., Sundin J., Goodwin L., Hull L., Wessely S., Fear N. T. | Sareen J., Belik S. L., Afifi T. O., Asmundson G. J., Cox B. J., Stein M. B. | Seelig A. D., Jacobson I. G., Smith B., Hooper T. I., Gackstetter G. D., Ryan M. A., Wells T. S., MacDermid W. S., Smith T. C., Millennium Cohort Study Team |
| --- | --- | --- | --- |
| **Year** | 2012 | 2008 | 2012 |
| **Sequence Generation** | High | High | High |
| **Allocation Concealment** | High | High | High |
| **Blinding** | 4 | 4 | 4 |
| Description | Non-blind |  |  |
| **Incomplete Outcome Data** | Unclear | 2 | 4 |
| Description | Phase 1: 59% response rate (10272); 9395 available for follow-up. 67% (6292) completed follow up. However main analysis focuses on subset of phase 1 participants that tested positive for PTSD (50+ score). Not reported how big this group was at phase 1. | Response rate regular forces 79.5% and reserves 83.5%. Missing data not mentioned. | 26% missing data. Response rates for women not reported: The first panel includes 16,876 women participants enrolled from 2001–2003 (in general 36% of those able to be contacted responded). A second panel was enrolled from 2004–2006 and consisted of 6,810 women participants (in general 25% of those able to be contacted responded). In general of Panel 1 participants, 71% responded to the 2004 and 2007 follow-up surveys, and 55% of Panel 2 participants responded to the 2007 follow-up survey. The present study included women members of the first and second panels who completed a baseline and at least one follow-up questionnaire (n =17,481). |
| **Reporting Bias** | 1 | 1 | 1 |
| Description |  |  |  |
| **Other Bias** | 2 | 3 | 2 |
| Description | Possible recall bias as follow-up takes place 3+ years later. | Possibly recall bias. Questionnaire measures combat or peacekeeping exposure more than 10 years back and "soldiers who reported deployment to both combat and peacekeeping missions could have been deployed to 1 peacekeeping mission where they were exposed to combat while peacekeeping or to separate combat and peacekeeping missions at different times" (page 2192). | Possibly recall bias. Questionnaire measures combat exposure as self-reported exposure to witnessing death, abuse, maimed soldiers or civilians, prisoners of war, or refugees, in the past 3 years. |
| **Apriori Protocol** | Yes | Unclear | Yes |
| Description | Data collected as part of British Iraq War Study (p 1192). |  | The study protocol was approved by the institutional review board at the Naval Health Research Center. |
| **Apriori Plan of Analysis** | Unclear | Unclear | Unclear |
| Description | Not stated |  |  |
| **Confounding** | 4 | 5 | 3 |
| Description | Does not show balance between deployers and non-deploying cases (purpose of the study is different than what is relevant to this review). Controls for a host of baseline information. | Four important confounders not considered and three post deployment covariates included. No imbalances shown or mentioned. Possibly four different missions are included (from 1991-1999) and it is not reported at what time the control group served. | All (except previous deployment) confounders (and more) controlled for but imbalances not reported and current alcohol problems are included. |
| Relevant confounders described by researchers? | Yes, p.1192, and table 2 (p.1194) | No | No |
| Assessment of relevant confounders | All considered except ethnicity (not sure how relevant that confounder is in UK context). | Not considered: Mental health history, previous deployment, branch and ethnicity. Post deployment marital status, income and education controlled for. No imbalances are shown, though gender separated analysis. | All except previous deployment and more is added. Current alcohol related problems included. Relevant (for us) imbalances not shown. |
| **Method**  **(Design Stage)** | None | Cross sectional / None | Population based survey (Millenium Cohort) |
| **Method**  **(Analysis Stage)** | Multinomial logistic regression | Logistic regression | Logistic regression |

| **Author** | Smith, T. C., Jacobson, I. G., Hooper, T. I., Leardmann, C. A., Boyko, E. J., Smith, B. et al. | Smith, T. C., Wingard, D. L., Ryan, M. A. K., Kritz-Silverstein, D., Slymen, D. J., & Sallis, J. F. | Smith, T. C., Zamorski, M., Smith, B., Riddle, J. R., Leardmann, C. A., Wells, T. S. et al. |
| --- | --- | --- | --- |
| **Year** | 2011 | 2009 | 2007 |
| **Sequence Generation** | High | High | High |
| **Allocation Concealment** | High | High | High |
| **Blinding** |  | 4 | 4 |
| Description |  |  |  |
| **Incomplete Outcome Data** |  | 2 | 4 |
| Description |  | First, although 71% participated in follow-up surveying between June 2004 and February 2006 (which they do not use?), the initial response to the invitation for participation was 36%. Data available for about 98%. Weight for response differences. | The invited Cohort was sampled from electronic personnel records representing approximately 11.3 percent of the 2.2 million men and women in service as of October 1, 2000 using a modified Dillman approach (design to maximise response). 36% response rate of those invited to participate and among them 97.9% had complete data. |
| **Reporting Bias** |  | 4 | 1 |
| Description |  | Do not show or mention relevant imbalances. | Do not report any imbalances. |
| **Other Bias** |  | 1 | 1 |
| Description |  |  |  |
| **Apriori Protocol** |  | Yes | Yes |
| Description |  | This research was conducted in compliance with all applicable federal regulations governing the protection of human subjects in research (Protocol NHRC.2000.007). | Protocol NHRC.2000.0007 (p.3) |
| **Apriori Plan of Analysis** |  | Unclear | Unclear |
| Description |  |  |  |
| **Confounding** |  | 4 | 4 |
| Description |  | A descriptive characteristic of the full sample and separated by the 3 outcome categories are provided but not imbalances between treated/not treated. | Report adjusted means but do not report imbalances on any of the confounders. Corrected for gender, age, race, rank, branch, and others (one of which is tenure a proxy for number of previous deployments). |
| Relevant confounders described by researchers? |  | None, except a technical: Confounding was investigated using a manual backward removal technique of variables that were not contributing significantly to the model, at p<0.05, while potentially distorting the measure of effect by more than 15%. What does that mean? | No |
| Assessment of relevant confounders |  | All, except mental health history and more is added. | Age, sex, education, marital status, race/ethnicity, length of service, military rank (this variable includes Duty/Enlistment status), branch of service, and occupational category are adjusted for but no reporting of imbalances. Do not consider mental health history. |
| **Method**  **(Design Stage)** | None | Cross sectional / None | Population based survey (Millenium Cohort) |
| **Method**  **(Analysis Stage)** | Multinomial logistic regression | Logistic regression | Logistic regression |

| **Author** | Soares H. L. | Southwick, S. M., Morgan, A. et al. | Southwick, S. M., Morgan III, C. A. et al. |
| --- | --- | --- | --- |
| **Year** | 2008 | 1993 | 1995 |
| **Sequence Generation** | High | High | High |
| **Allocation Concealment** | High | High | High |
| **Blinding** | 4 |  |  |
| Description |  |  |  |
| **Incomplete Outcome Data** | Unclear |  |  |
| Description | Nothing reported |  |  |
| **Reporting Bias** | 1 |  |  |
| Description |  |  |  |
| **Other Bias** | 1 |  |  |
| Description |  |  |  |
| **Apriori Protocol** | Unclear |  |  |
| Description |  |  |  |
| **Apriori Plan of Analysis** | Unclear |  |  |
| Description |  |  |  |
| **Confounding** | 5 |  |  |
| Description | Nothing reported except that those experiencing combat were older than those not. Include several branches and active as well as guards and men and women. |  |  |
| Relevant confounders described by researchers? | No |  |  |
| Assessment of relevant confounders | Nothing considered |  |  |
| **Method**  **(Design Stage)** | None |  |  |
| **Method**  **(Analysis Stage)** | None |  |  |

| **Author** | Stuart J. A., Halverson R. R., | Taft, C. T., Schumm, J. A., Panuzio, J., & Proctor, S. P. | Tanielian & Jaycox |
| --- | --- | --- | --- |
| **Year** | 1997 | 2008 | 2008 |
| **Sequence Generation** | High | High | High |
| **Allocation Concealment** | High | High | High |
| **Blinding** | 4 | 4 | 4 |
| Description | Assessments taken *during* deployment spell. |  |  |
| **Incomplete Outcome Data** | 4 | Unclear | 3 |
| Description | No description of eligible pool. No comparison between responders and non-responders. Non-missing data level 83%-100%. | Not specifically mentioned (original sample included 60% of Fort Devens, MA.). Time 1 N =2949, Time 2=1512 only individuals residing with family sampled. No mention of how many non-responses. | 3771 eligible, 1508 (40%) refused or/unable, 325 (9%) call-backs not complete. 1938 interviewed (p. 90). |
| **Reporting Bias** | 1 | 1 | 1 |
| Description |  | No specific mention |  |
| **Other Bias** | 1 | 1 | 1 |
| Description |  |  |  |
| **Apriori Protocol** | Yes | Yes | Unclear |
| Description | "Approval to conduct human dimensions research dealing with the psychological status of active service US Army solders was obtained through Army Medical Command channels, which included human use protocol submission and approval" (p.739). | A prospective design in which combat 'exposure and PTSD symptomatology were assessed immediately 'upon the soldier’s return from the deployment, prior to joining with family (Time 1), and family adjustment was assessed between 18 and 24 months later (Time 2). |  |
| **Apriori Plan of Analysis** | Unclear | Unclear | Unclear |
| Description | Not explicitly stated | Exploratory: examine "causal" link between PTSD and family functioning. |  |
| **Confounding** | 4 | 5 | 4 |
| Description | Balance or distributional overlap not presented or discussed but can be calculated from table 1. Large imbalance on gender, some imbalance on ethnicity and minor on age and rank. | No discussion of confounding/selection. Separate models for gender, or how combat exposure varies with e.g. Rank. | Not all considered and two additional confounders may be highly correlated. No imbalances shown or discussed. |
| Relevant confounders described by researchers? | Yes: p.739. Motivation for inclusion not given. | Gender, p.649 | No |
| Assessment of relevant confounders | Balance nor overlap in distributions, not reported or commented on but can be calculated from table 1. Large imbalance on gender, some imbalance on ethnicity and minor on age and rank. Confounders not considered: enlistment status, previous deployments, prior mental health. | Gender sep. Analysis, US army only. | Not considered: mental health history, enlistment status (at time of deployment) and number of previous deployments. No imbalances shown or discussed. In addition they control for current duty status: active, reserve/guard and discharged/retired and another variable: seriously injured. These confounders may be correlated. |
| **Method**  **(Design Stage)** | None | Prospective design in which combat exposure and PTSD symptomatology were assessed immediately upon the soldier’s return from the deployment, prior to joining with family (Time 1), and family adjustment was assessed between 18 and 24 months later (Time 2). | Random sampling strategy |
| **Method**  **(Analysis Stage)** | OLS | Sep. Analysis by gender. | Weight to improve representativeness to the total population of OEF/OIF veterans and adjusted relative risk ratio (probably a log-binomial model as they state they use the SAS proc genmod.), although RR associated with trauma exposure is the incremental risk associated with each additional trauma. Does not make sense/It is like a marginal effect. By how much does the relative risk increase for an additional trauma? (constant by model construction). |

| **Author** | Thomas J. L., Britt T. W., Odle-Dusseau H., Bliese P. D. | Vasterling, J. J., Proctor, S. P., Friedman, M. J., Hoge, C. W., Heeren, T., King, L. A. et al. | Vogt, D. S., Pless, A. P., King, L. A., & King, D. W. |
| --- | --- | --- | --- |
| **Year** | 2011 | 2010 | 2005 |
| **Sequence Generation** | High | High | High |
| **Allocation Concealment** | High | High | High |
| **Blinding** | 4 | 4 | 4 |
| Description | Participants clearly not blinded. Not stated whether investigators were. |  |  |
| **Incomplete Outcome Data** | 3 | 3 | 3 |
| Description | 2984/3500 of the unit were briefed. 2439/2984 gave written consent (p869). No comparison of non-responses. | At enrollment, 94% of 1633 invited soldiers participated voluntarily. Of the 1542 soldiers assessed at Time 1, 73% (n = 1124) participated in onsite assessment at Time 2. Soldiers most commonly were excluded from Time 2 assessment because they were no longer with their originating unit (48% relocated to another unit, 24% separated from service, 5% for unknown reasons).Only 2% declined Time 2 participation. Forty-one participants were excluded for invalid or incomplete questionnaire responses." (p.42) | Response rate 66%, women 56%, men 67%, deployed from active duty 41% and Reserve/Guard 78%. (p.274) |
| **Reporting Bias** | 1 | 1 | 4 |
| Description |  |  | Do not show imbalances, only A table containing demographic and background characteristics of the sample are available from the first author. In footnote 7 it is stated that: A supplemental set of regression analyses was conducted to evaluate associations after accounting for gender differences in demographic/background characteristics. With one exception, the interpretations of these findings did not change. Results are not shown. |
| **Other Bias** | Unclear | 1 | 1 |
| Description | Confidentiality of survey not stated. |  |  |
| **Apriori Protocol** | Yes | Unclear | Unclear |
| Description | P.869 | Not stated, but prospective data-collection. |  |
| **Apriori Plan of Analysis** | Unclear | Unclear | Unclear |
| Description | Not explicitly stated | Not stated |  |
| **Confounding** | 5 | 4 | 5 |
| Description | Describe some demographics on p.869, but no discussion on confounding, nor any argument as to under what conditions exposure intensity can be considered exogenous to the individual. | All confounders (and more considered). Does not adjust for ethnicity, rank, number of previous deployments. Controls for DRRI post-deployment events + family stressors which may be correlated with battle stress. | Only gender is controlled for. High risk of multicollinearity. Include three different combat exposure measures in the model along with gender interaction terms. Although (page 276 it is stated): For ease of interpretation, the variables representing the main effects of deployment factors were centred prior to the calculation of the product terms, (...). Centring is a procedure that involves subtracting the mean from all scores and that has the effect of reducing problems associated with multicollinearity.' They only centre the interaction terms. |
| Relevant confounders described by researchers? | No; but see mention on p 869 | No, but shown in table 1 | Uses the literature |
| Assessment of relevant confounders | None considered, although only active-duty army brigade combat team soldiers included. | All confounders (and more considered). Does not adjust for ethnicity, rank, number of previous deployments. Do not show the (for us) relevant imbalances. | Only gender is controlled for. Do not show imbalances, only report: female participants reported being deployed for a significantly longer period of time than their male counterparts. Female participants were more likely to have been deployed from the Reserves while male participants were more likely to have been deployed from the National Guard. Female veterans were, on average about 7 years younger than male veterans. Female veterans were more likely to belong to an ethnic or racial minority group than male veterans. Female veterans had fewer children, on average, compared with male veterans. However, is only useful for PTSD as the depression and anxiety measures were administered to only half of the sample. |
| **Method**  **(Design Stage)** | None | None (for the sample in table 3, they do recruit prospectively a match active duty comparison contrast, but not relevant here) | Unclear |
| **Method**  **(Analysis Stage)** | Multilevel modelling | Checks for within-battalion correlation (ICCs). Hierarchical OLS. | Hierarchical regression |

| **Author** | Vogt, D. S. & Tanner, L. R. | Vogt D. Vaughn R., Glickman M. E., Schultz M., Drainoni M. L., Elwy R., Eisen S. | Waller M., Treloar SA., Sim MR., McFarlane A. C., McGuire A. C., Bleier J., Dobson AJ. |
| --- | --- | --- | --- |
| **Year** | 2007 | 2011 | 2012 |
| **Sequence Generation** | High | High | High |
| **Allocation Concealment** | High | High | High |
| **Blinding** | 4 | 4 | 4 |
| Description |  |  | Non-blind |
| **Incomplete Outcome Data** | 3 | 3 | 2 |
| Description | Response rate 64% | Response rate 57%. They use non-response weights to make estimates for sampled population, but this effectively assumes non-response is only due to observed characteristics. | Response rate were 49% and 46% for Bougainville and East Timor. Analysis of non-response in table 2, p5. Appear to be relatively minor differences. |
| **Reporting Bias** | 1 | 4 | 2 |
| Description |  | They say they will control for confounders, but then simply notes no change in "results" (footnote 2). Surely coefficients or SE must be affected? | Does not report effect of quartiles of exposure variables. |
| **Other Bias** | 1 | 1 | 4 |
| Description |  |  | ADF faced deployments to Afghanistan (since 2001) and Iraq (since 2003). Models are not corrected for deployments to Middle East, (measurement bias). Measurements taken 8 years after deployment (recall bias). |
| **Apriori Protocol** | Unclear | Yes | Yes |
| Description | Probably convenience sample. They contact respondents who were left over from a different study (p29). | P.799 | Approved by Australian Defence Human Research Ethics Committee (p.2). |
| **Apriori Plan of Analysis** | Yes | Unclear | Unclear |
| Description | Uses the literature | Not stated | Not explicitly stated |
| **Confounding** | 5 | 5 | 4 |
| Description | A descriptive characteristic of the full sample and only gender (of the predefined confounders) are controlled for. | Do not include background factors but include several different deployment stressors in the regression (correlations of each stressor with outcomes are also available). They include "Prior life stressors" which I consider a good proxy for prior mental health. They also argue that in their sample our confounding variables do not affect "results". Do we believe them? | Study does not demonstrate that exposure is exogenous to participants, nor does it demonstrate balance on confounders (Imbalance for deployment locations which is only a proxy for exposure is reported). |
| Relevant confounders described by researchers? | The literature on the subject is discussed. | No, some discussion of this on p 801. | Yes, table 2, p.5 |
| Assessment of relevant confounders | Only gender, childhood family environment and pre-deployment number of stressful events controlled for. | State that background factors could not account for observed differences and do not include them (except gender which is their main interest). | Regression model adjusted for: age, gender, service, rank, and quartiles of traumatic and non-traumatic stress. Imbalance (note based on deployment location which is only a proxy for exposure) on age and rank, only minor on gender and branch. |
| **Method**  **(Design Stage)** | None | Stratified on gender and enlistment status and only those returning within the past year. | None |
| **Method**  **(Analysis Stage)** | Mix of measurement and structural model, exposure to warfare and effect on posttraumatic stress symptomatology (PTSS). | Regression | Logistic regression |

| **Author** | Ward, W. | White, R. F., Proctor, S. P., Heeren, T., Wolfe, J., Krengel, M., Vasterling, J. et al. | Wolfe, J., Brown, P. J., & Kelley, J. M. |
| --- | --- | --- | --- |
| **Year** | 1997 | 2001 | 1993 |
| **Sequence Generation** | High | High | High |
| **Allocation Concealment** | High | High | High |
| **Blinding** |  | 4 | 4 |
| Description |  | Questionnaire |  |
| **Incomplete Outcome Data** | Unclear | 3 | Unclear |
| Description |  | See notes to 4446 | Do not mention response rate or missing data, but from 6777: Treated: the Devens cohort. Original sample response rate was 79% (n: 2330). "Nearly all units were contacted, and examination of nonparticipants indicated absences primarily for administrative and medical purposes" (p.18). Potentially problematic if those away for medical purposes are at higher risk of PTSD, etc. |
| **Reporting Bias** | 1 | 1 | 1 |
| Description |  | Probably yes |  |
| **Other Bias** | 1 | 2 | 1 |
| Description |  | Recall bias |  |
| **Apriori Protocol** | Unclear | Yes | Unclear |
| Description |  | "The Institutional Review Board approved the protocol..." (p.44) |  |
| **Apriori Plan of Analysis** | Unclear | Unclear | Unclear |
| Description |  | The present study was aimed at (1) exploring the possibility that GW veterans would show evidence of CNS dysfunction on neuropsychological tests and (2) examining whether performance on neuropsychological tests was related to specific chemical exposures experienced in the Gulf. |  |
| **Confounding** | 5 | 5 | 4 |
| Description |  | "SUDAAN analyses: adjusted for age, education, gender, and sampling design. Comparison wise P-values not adjusted for, multiple comparisons are presented" (p.51). Raw imbalances not shown, only adjusted for sampling design, remarkably less imbalanced than raw imbalances shown in for example 4446. | High risk of multicollinearity. Include three different combat exposure measures in the model along with gender interaction terms. Correlation between Laufer and (2) is .53 so I would not be that concerned about multi. They include and discuss a wide range of background variables. |
| Relevant confounders described by researchers? | No | No | No |
| Assessment of relevant confounders | Show imbalances on age and rank but nothing is controlled for. | Several confounders recorded (not rank, branch, enlistment type, mental health history), but not controlled for. Estimates only controlled for age, education, gender and sampling design. | Control for gender, education, marital status, rank, race, prior war-zone service. No control for prior mental health. Devens is possibly Army only (I seem to recall from different study). |
| **Method**  **(Design Stage)** |  | Random stratified sampling of original treated sample (but lack of overlap). | None (attempt to sample entire Ft Devens population) |
| **Method**  **(Analysis Stage)** |  | SUDAAN adjusted means | OLS |

| **Author** | Wolfe, J., Erickson, D. J., Sharkansky, E.J., King, D.W. and King, L.A. | Wolfe J., Proctor S. P., Davis J. D., Borgos M. S., Friedman M. J., | Wood M. D., Foran H. M., Britt T. W., Wright K. M. |
| --- | --- | --- | --- |
| **Year** | 1999 | 1998 | 2012 |
| **Sequence Generation** | High | High | High |
| **Allocation Concealment** | High | High | High |
| **Blinding** | 4 | 4 | 4 |
| Description |  |  |  |
| **Incomplete Outcome Data** | 2 | 2 | 2 |
| Description | At time 1 no response rate reported but The cohort represented approximately 60% of the military personnel deployed from Ft. Devens to the Gulf region. Those not surveyed were in units that were unavailable for participation, primarily because of general administrative (e.g., out-processing) purposes. At Time 2, roughly 78% of the original sample participated. Non-responders were more likely to be younger, to be a member of a minority group and to be deployed from active duty. No mentioning of missing data. | At time 1 no response rate reported but The cohort represented approximately 60% of the military personnel deployed from Ft. Devens to the Gulf region. Those not surveyed were in units that were unavailable for participation, primarily because of general administrative (e.g., out-processing) purposes. At Time 2, roughly 78% of the original sample participated. Non-responders were more likely to be younger, to be a member of a minority group and to be deployed from active duty. No mentioning of missing data. "Nearly 79% of the original cohort completed the re-evaluation (n=2313)..." (p. 105). "We analysed the data collected from 2119 subjects ... at time 2" (p. 106). | 95% of the soldiers consented (1,762 of 1,850). However only junior enlisted soldiers (824 of 1,762) who deployed (600 of 824) and had combat experience (583 of 600) were included. |
| **Reporting Bias** | 1 | 1 | 1 |
| Description |  |  |  |
| **Other Bias** | 1 | 1 | 1 |
| Description |  |  |  |
| **Apriori Protocol** | Unclear | Unclear | Unclear |
| Description |  |  | "This study was approved by IRB of the WRAIR" (p.532). |
| **Apriori Plan of Analysis** | Unclear | Unclear | Unclear |
| Description |  |  |  |
| **Confounding** | 5 | 5 | 5 |
| Description | Correlation without adjustment for anything although only Army personnel is included. | Percent reported without adjustment for anything although only Reserve/Guard Army personnel is included. | Important confounders not considered, no imbalances shown only two discussed. They write: "Demographic covariates were selected if correlated with a predictor or outcome variable, and gender and multiple deployments met these criteria" (p.534). This is a less than satisfying procedure in terms of handling confounding. Demographics: Age, gender, education, multiple deployments, years in military (from table 1, p.533). |
| Relevant confounders described by researchers? | Prior research | No | No |
| Assessment of relevant confounders | Gender, age, ethnicity, military status, education, marital status, rank, prior combat experience used in the logistic regression which unfortunately cannot be used due to lack of information necessary to calculate SE. | Army only sample, otherwise nothing is controlled for and no imbalances reported or discussed. | Gender, multiple deployments, benefit finding (BF), non-commissioned officer (NCO) leadership and various interaction terms: Combat x BF; Combat x NCO Leadership; BF x NCO leadership and a three-way interaction term (Combat x BF x NCO Leadership). No imbalances shown but gender and multiple deployments vary significantly with PTSD. |
| **Method**  **(Design Stage)** | Unclear | None | None |
| **Method**  **(Analysis Stage)** | None | None | Multiple regression |

| **Author** | Woodhead C., Wessely S., Jones N., Fear N. T., Hatch S. L. | Wooten N. R. | Wooten N. R. |
| --- | --- | --- | --- |
| **Year** | 2012 | 2010 | 2012 |
| **Sequence Generation** | High | High | High |
| **Allocation Concealment** | High | High | High |
| **Blinding** | 4 | 4 | 4 |
| Description | Non-blind | Non-blind | Non-blind |
| **Incomplete Outcome Data** | 3 | 4 | 4 |
| Description | Response rate phase 1, 59%. They use phase 2, where they replenish phase 1 sample with HERRICK, and contact Phase 1 sample again. Response rate: 56%. Not reported how large a percentage of Phase 1 sample they were able to reconnect with. | 420 ARNG women mailed. 59 with invalid addresses + 1 duplicate listing (p 100). Out of 360, 132 were returned. Only 98 could be included in analyses (and only 87-88 used for model). Also describe using algorithm to impute missing data (p 101). Does not report how many obs. this algorithm is applied to. Compares responders to non-responders. Significant differences on demographics. | 420 ARNG women mailed. 59 with invalid addresses. Out of 361, 135 were returned. Only 101 could be included in analyses (and only 91 used for model). Also describe using algorithm to impute missing data (p 836). Does not report how many obs. this algorithm is applied to. Compares responders to non-responders. Significant differences on demographics. |
| **Reporting Bias** | 1 | 1 | 2 |
| Description |  |  | PhD Dissertation building on same data contains additional outcomes (Depression, Alcohol Misuse). Models for both these measures are insignificant. |
| **Other Bias** | 2 | 2 | 2 |
| Description | Possible recall bias, particularly for phase 1 sample. | Recall bias | Recall bias |
| **Apriori Protocol** | Unclear | Yes | Yes |
| Description | Not explicitly stated | P.80 | Stated on p.833 |
| **Apriori Plan of Analysis** | Unclear | Unclear | Unclear |
| Description | Not stated | Not explicitly stated | Not explicitly stated |
| **Confounding** | 4 | 5 | 5 |
| Description | Adjusted for age, rank, marital status, enlistment status, gender (split analysis), service type, and medical role in theater. No presentation/discussion of balance based on exposure (hi/low). | Only corrects for age, although appears to possess the confounder list, or proxies for them. Does not claim causality, studies "associations"/correlations. | Only corrects for age, although appears to possess the confounder list, or proxies for them. Does not claim causality, studies "associations"/correlations. |
| Relevant confounders described by researchers? | Yes, p.3-4 | Yes, p.95-97 | No discussion, but presentation of them. |
| Assessment of relevant confounders | Number of previous deployments and prior health not controlled for. Balance not reported/discussed. | The study contains all confounders in some form. But final model only corrects for age and prior trauma and in addition post-deployment factors and post-deployment social support. See sheet for #12539. | The study contains all confounders in some form. But final model only corrects for age and prior trauma and in addition post-deployment factors and post-deployment social support. |
| **Method**  **(Design Stage)** | None | None | None |
| **Method**  **(Analysis Stage)** | PTSD-score: negative binomial regression; CMD, Alco: logistic regression (all weighted). | OLS + logistic regression | OLS |

| **Author** | Wright K. M., Cabrera O. A., Adler A. B., Bliese P. D. |
| --- | --- |
| **Year** | 2013 |
| **Sequence Generation** | High |
| **Allocation Concealment** | High |
| **Blinding** | 4 |
| Description |  |
| **Incomplete Outcome Data** | 4 |
| Description | Of time 1 participants 54% were followed up at time 2 and/or 3 and used as time 1 participants. Time 2 FU rate (used) 46% and time 3 24% Some discussion on p.555 re. Potential bias from this (they use multiple imputation). Argue that it generally works for this population. 76% gave consent to participate. |
| **Reporting Bias** | 1 |
| Description |  |
| **Other Bias** | 1 |
| Description |  |
| **Apriori Protocol** | Yes |
| Description | P.546 |
| **Apriori Plan of Analysis** | Unclear |
| Description |  |
| **Confounding** | 4 |
| Description | Nothing controlled for except age and ethnicity in the growth model we do not use. I argue we could use ME (combat) in table 5. If not then we would only have a correlation from Table 4 to work with and I would agree on 5. HLM Model controls for combat exposure, functional impairment, age, ethnicity, and rank (p.550). Army only (Brigade Combat Team and p.547). |
| Relevant confounders described by researchers? | No |
| Assessment of relevant confounders | 98% report it is their first deployment (to Iraq). Otherwise nothing controlled for and no relevant (for us) imbalances shown. |
| **Method**  **(Design Stage)** | None |
| **Method**  **(Analysis Stage)** | Nothing (we can use) / HLM |

## Risk of Bias: Analysis Sample

| **Author** | Al-Turkait, F. A. & Ohaeri, J. U. | Baggaley, M. R., Piper, M. E., Cumming, P., & Murphy, G. | Black, D. W., Carney, C. P., Peloso, P. M., Woolson, R. F., Schwartz, D. A., Voelker, M. D. et al. |
| --- | --- | --- | --- |
| **Year** | 2008 | 1999 | 2004 |
| **Sequence Generation** | High | High | High |
| **Allocation Concealment** | High | High | High |
| **Blinding** | 4 | 4 | 4 |
| Description |  |  |  |
| **Incomplete Outcome Data** | Unclear | 3 | 2 |
| Description | Sampling strategy was to sample 200 from each exposure stratum. Then pick 50 at random and replenish in case of non-response until 50 sampled. No analysis re. How non-response varied between strata. | Appears that total population considered is 400+100 (p.13). 382 usable questionnaires returned response rate 79.4% (not separate on T/C) and missing data rate 4%. | Randomly drawn sample (N=4886). Response rate 76%. 3695 completed. |
| **Reporting Bias** | 2 | 1 | 2 |
| Description | Key statistics related to confounder analysis not presented (only F-test for full model). | Probably yes | Imbalances not shown (possibly discussed in related papers). |
| **Other Bias** | 1 | 2 | 2 |
| Description | Probably yes | Recall bias | Prior mental health 6 years after susceptible to recall bias. Also telephone interview where validation is weak. |
| **Apriori Protocol** | Yes | Unclear | Yes |
| Description | Qua sampling strategy |  | See Doebbeling et al., 2002 |
| **Apriori Plan of Analysis** | Unclear | Unclear | Yes |
| Description | Many models are tested | They collect more information than is presented or controlled for in the paper. | See Doebbeling et al., 2002 |
| **Confounding** | 5 | 5 | 2 |
| Description | Only crude counts appear usable. Subsamples appear to have very little overlap on covariates (p 936, table 1). | Only present age (which differs significantly). Mentions additional tours as confounder, and the fact that some were deployed in NI at the time measurement was taken. | Only minor imbalances on stratification variables. Imbalance on mental health history not reported. |
| Relevant confounders described by researchers? | No | No | No |
| Assessment of relevant confounders | The 4 subsamples are highly imbalanced on all presented confounders except marriage status: Only consider age, education, income, rank and marital status. | Only age presented. | All, except number of previous deployments. |
| **Method**  **(Design Stage)** | None | None | Within the four domains Active GW deployed, Active non-deployed, Guard/Reserve GW deployed and Guard/Reserve non-deployed a random stratified (on branch of service, rank, gender, race and age) sample were drawn and logistic regression used in analysis. |
| **Method**  **(Analysis Stage)** | None | None | Logistic regression + SUDAAN (weights) |

| **Author** | Booth-Kewley S., Schmied E.A., Highfill-McRoy R.M., Larson G.E., Garland C.F., Ziajko L.A. | Booth-Kewley, S., Larson, G. E., Highfill-McRoy, R. M., Garland, C. F., & Gaskin, T. A. | Breen-Lopez C. J. |
| --- | --- | --- | --- |
| **Year** | 2013 | 2010 | 2014 |
| **Sequence Generation** | High | High | High |
| **Allocation Concealment** | High | High | High |
| **Blinding** | 2 | 4 | 4 |
| Description | Outcome assessors were likely unaware of study since they are based on psychiatric diagnosis in TRICARE system. | "Participation was voluntary and military unit commanders were not present during enrollment or questionnaire completion. Study enrollment and survey administration was performed by civilian researchers." […] "The survey was not anonymous. To allow for a possible follow-up assessment, participants were asked to provide their social security numbers and names. Potential participants were assured that all data would be kept completely confidential and no one in their chain of command would ever see their data." (p71) |  |
| **Incomplete Outcome Data** | 2 | 2 | 2 |
| Description | 1576 marines in original Warfighter Status Survey (response rate of 78%). Excluded from WSS: not providing identifying information, unable to find matching data in CHAMPS and DMDC, leading to a sample of 1291. 178 were excluded because they had a pre-existing psychiatric condition at time of WSS. This study consists of subset of 1113 from WSS. | Each participant was given a small gift (either a $5 fast food gift card or a computer flash drive of comparable value) in exchange for participation. The overall response rate was 78%. (p71) | Of 1899 participants, 230 (+2) participants had missing values (12%). Due to the recruitment procedure response rate is not relevant to consider / we do not know how many they tried to recruit. Still author does not report for which variables data are missing. Also, observations where outcome is available but where a confounder is missing are excluded (p39). Since we cannot calculate a response rate (we only know how many took the survey), I find 12% missing outcomes/data to be high. |
| **Reporting Bias** | 4 | 2 | 1 |
| Description | Estimation procedure based on elimination of insignificant variables leads to exclusion of e.g. combat measure for some outcome equations. | Does not control for gender and rank and univariate analysis are not shown for these covariates. |  |
| **Other Bias** | 1 | 2 | 2 |
| Description | To ensure confidentiality, participants' data were stored separately from identifiers, and after matching, identifiers were stripped from the data. The participants' chain of command never had access to any part of their data. | Recall bias. Uses PCL-C which means that participants could have reported trauma that occurred not as result of deployment. Difficult to judge whether it plausibly varies between treatment. | State on p. 75: "Initially, participants were referred to the study by a VAMC provider. More recently, participants entered the study as volunteers, in response to the recruitment flyers, bulletins, posters, and mailings advertising the study”. |
| **Apriori Protocol** | Yes | Unclear | No |
| Description | Protocol NHRC.2007.0003 (p.3) | Probably yes, but retrospective study |  |
| **Apriori Plan of Analysis** | Unclear | Unclear | Unclear |
| Description | Not explicitly stated |  | States hypotheses / Stated in the same document, not in a separate document in advance. |
| **Confounding** | 3 | 4 | 4 |
| Description | Balance, or distributional overlap not presented or discussed. All relevant confounders considered. | Discussion of active duty/reservist (p 70). "… standard demographic variables" (p 70). Do not show/consider imbalances and do not control for gender, rank and mental health history. Control for deployment status at post deployment. | Not considered: Mental health (but controls for previous trauma), rank, branch, enlistment and previous deployment. No imbalances on CE shown or discussed. |
| Relevant confounders described by researchers? | Yes, p1-2. | Yes, from previous literature. | Yes, based on previous literature (but focus is on gender) |
| Assessment of relevant confounders | Balance nor overlap in distribution between low/med/high exposure not described (but not prime purpose of study either). All relevant confounders controlled for (in PTSD). | All except prior mental health. However only covariates that were significant in the univariate analysis were entered into the model. Does not control for gender and rank and univariate analysis are not shown for these covariates. | Not considered: Mental health, rank, branch, enlistment and previous deployment. Age, race, education, marital status, educational status and non-combat trauma events (= correlate of mental health) considered. No imbalances on CE shown or discussed. |
| **Method (Design Stage)** | None | None | None |
| **Method (Analysis Stage)** | Logistic regression | Logistic regression | Hierarchical logistic regression |

| **Author** | Cerdá, M., C. Richards, G. H. Cohen, J. R. Calabrese, I. Liberzon, M. Tamburrino, S. Galea, K. C. Koenen. | Chapman P. L., Elnitsky C., Thurman R. M., Pitts B., Figley C., Unwin B. | Coughlin S.S., Kang H.K., Mahan C.M. |
| --- | --- | --- | --- |
| **Year** | 2014 | 2014 | 2011 |
| **Sequence Generation** | High | High | High |
| **Allocation Concealment** | High | High | High |
| **Blinding** | 4 | 4 | 4 |
| Description |  |  |  |
| **Incomplete Outcome Data** | 3 | 4 | 2 |
| Description | Response rate of 43.2%. Respondents were excluded if they had not been deployed by Wave 1 or declined to report deployment status, had no follow-up data, never consumed alcohol, or were missing data on AUD timing (58% excluded) / The web appendix gives details (p.2). 2,616 agreed to participate (out of 6514 eligible). 948 did not deploy by Wave 1, 1259 had wave 2/or 3 data. | Main data (from Chapman et al. 2012): 347 deployed and 494 non-deployed. Used for this study: 196 (56%) deployed and 256 (52%) non-deployed. No explanation of why not all included, except they state that they exclude soldiers with combat-related physical injuries requiring overnight stay at a hospital during deployment. | From 12919: For the baseline survey in 1995, 11,441 deployed veterans (76%) and 9,476 nondeployed veterans (63%) participated. In 2005 6,111 (40%) deployed and 3,859 nondeployed (27%) responded (of the 29,607 living panel members). The most common reasons for nonparticipation in the postal survey were that people did not remember receiving the survey (32% of nonrespondents); were not interested in participating in the survey (21%); intended to complete the survey (16%); did complete the survey and mail it back (7%); gave no particular reason for not completing the survey (7%); felt the survey did not apply to them (5%); were deployed or out of town (4%); and thought the survey was too long and involved (3%). |
| **Reporting Bias** | 1 | 1 | 1 |
| Description |  |  |  |
| **Other Bias** | 2 | Unclear | 1 |
| Description | Respondents were excluded if they had not been deployed by Wave 1 or declined to report deployment status, had no follow-up data, never consumed alcohol, or were missing data on AUD timing (58% excluded). | Does not explain how the analysis sample was chosen (if it was chosen and not just reduced due to missing variables) |  |
| **Apriori Protocol** | Unclear | Yes | Unclear |
| Description | IRBs have approved the study, so some form of protocol must have been produced (p462). | Stated in Chapman et al 2012. |  |
| **Apriori Plan of Analysis** | No | No | Unclear |
| Description |  |  |  |
| **Confounding** | 3 | 5 | 4 |
| Description | Includes military sexual harassment, post-battle traumatic events and (p. 462): Civilian stressors during deployment, including family disruption, and stressors following deployment, including changes in employment status, problems with health insurance, and legal problems are commonplace in military families. Related characteristics, such as divorce and lower family income, have been linked with alcohol misuse in military samples. = they control for stressors that may have been caused by deployment / My reading is that this is what the fully adjusted model does. The single exposure model controls for AUD history (prior wave), study wave (essentially time), age, gender, and marital status (post deployment?). They do not display balance lo/med/hi exposure. | Excludes physical injured because of the high correlation of physical injury and mental health issues (and further time out of theatre) (p.20). Some imbalance on most confounders. | Two important confounders not considered and one not controlled for. Controls for problem: drinking, which is possibly itself an outcome of deployment. None or only small imbalances. No discussion of exogeneity assumption of deployment assignment. |
| Relevant confounders described by researchers? | The purpose is to examine the modifying role of civilian stressors following deployment. | No | No |
| Assessment of relevant confounders | Not considered: rank, ethnicity and previous deployment. Considered: Army Guard only. Controls for alcohol disorder history, age, gender, marital status, sexual harassment, post-battle traumatic events and after deployment civilian stressors. No imbalances shown / p.1 in web appendix shows descriptive stats. About 88% whites. | Not considered: mental health history. Considered: age, gender, rank, ethnicity, education and marital status. Army combat medics sample. Compares to never deployed and 'most' were returning from their second deployment. Some imbalance on all variables except ethnicity and minor on education. | All, except previous deployment and mental health history (and Enlistment status is considered with a minor imbalance but not controlled for). Only minor imbalances on covariates. Problem drinking is bad controlled. |
| **Method (Design Stage)** | All Ohio Army National Guard members who served in June 2008-February 2009 were contacted. | Unclear, nothing (except numbers) reported in Chapman et al 2012 and no explanation given to the lower number analysed. | From 11941: The sampling design is a stratified random sample with unequal probabilities of selection within combinations of the strata: deployment status, gender and duty type (active service v. reserve or National Guard). |
| **Method (Analysis Stage)** | A generalized linear mixed model with a random intercept was used to estimate Ses. | Sequential logistic regression (p. 21: after partialing out significant demographic characteristics... and depression (PTSD) screening outcomes). Not further explained. | Logistic regression |

| **Author** | David, A. S., Farrin, L., Hull, L., Unwin, C., Wessely, S., & Wykes, T. | Dedert, E. A., Green, K. T., Calhoun, P. S., Yoash-Gantz, R., Taber, K. H., Mumford, M. M. et al. | Farmer, Vaughan, Garnet & Weinick |
| --- | --- | --- | --- |
| **Year** | 2002 | 2009 | 2014 |
| **Sequence Generation** | High | High | High |
| **Allocation Concealment** | High | High | High |
| **Blinding** | 3 | 4 | 4 |
| Description | RA's were blind to participant assignment, order of assessment randomised. See p 1359. |  |  |
| **Incomplete Outcome Data** | Unclear | Unclear | Unclear |
| Description | They compare responders to non-responders, table 1, p1360. No further analysis. | Nothing reported. Self-selected paid volunteers. | State on p. 14 that 2620 completed the survey and 355 (12%) declined to participate but the number who passively declined (did not return a blank survey) is unknown. |
| **Reporting Bias** | 1 | 1 | 1 |
| Description | Probably yes |  |  |
| **Other Bias** | 1 | 2 | 1 |
| Description |  | The TLEQ is a retrospective account of trauma exposure. (p.835) |  |
| **Apriori Protocol** | No | No | Yes |
| Description | Retrospective. Phase 2 of epidemiological study (p.1358); sample selected on the basis of SF36-PF score. |  | Before the survey was administered, a survey administrator would read the Human Subjects Protection Committee (HSPC)–approved oral consent script describing the study’s purpose and relevant information about human subjects’ research protections |
| **Apriori Plan of Analysis** | Unclear | No | Unclear |
| Description | Probably no. They sampled both ill/healthy Gulf, but only ill Era and Bosnia. Then discovered case crossovers (p 1362). |  |  |
| **Confounding** | 5 | 5 | 5 |
| Description | Their main analysis controlled for age, education and IQ, but does not give effect measure hence we must rely on raw counts. | 'Sampling: Veterans who had served since 9/11/01 in the US military and were enrolled in the VA (a multi-site research study conducted through the Department of Veterans Affairs Mid-Atlantic 6 Mental Illness Research, Education and Clinical Center (MIRECC).) were sent letters inviting them to participate in a study on post-deployment mood, and mental and physical health. Participants were also referred to the study through fliers and clinical providers. (p 831). No reporting of imbalances. "Participants were self-selected volunteers who responded to parid research recruitment, and most were enrolled as VA patients, so they may have had higher trauma exposure and psychopathology and may not be representative of the entire cohort of veterans serving since 9/11/01." (p.835) | The only adjustment is weighting so the sample is representative of the current population of marines preparing for deployment (for our purpose it is the post deployment sample). |
| Relevant confounders described by researchers? | No | No | None |
| Assessment of relevant confounders | Counts not adjusted for age, education, IQ. | Only gender, age and ethnicity and intellectual functioning and imbalances not reported. | Not considered: gender, mental health history, enlistment status and number of previous deployments. No imbalances shown or discussed. |
| **Method**  **(Design Stage)** | Stratification | None | None |
| **Method**  **(Analysis Stage)** | None (their main analysis uses ANCOVA) | Logistic regression | Weighting so the sample is representative of the current population of marines preparing for deployment (for our purpose it is the post deployment sample) |

| Farmer, Vaughan, Garnet & Weinick | Foster, E. M. | Gehrman, Seelig, Jacobson, Boyko, Hooper, Gackstetter, Ulmer & Smith | Gordon, J. G. A. |
| --- | --- | --- | --- |
| 2014 | 2011 | 2013 | 2002 |
| High | High | High | High |
| High | High | High | High |
| 4 | 4 | 4 | 4 |
|  |  |  |  |
| Unclear | 3 | 3 | 2 |
| State on p. 14 that 2620 completed the survey and 355 (12%) declined to participate but the number who passively declined (did not return a blank survey) is unknown. | Response rate was 55.3%. The data were weighted to represent all Reserve component personnel, using sampling weights that are a function of age, gender, education, and race/ethnicity. | The study’s first panel includes 77,047 participants enrolled from 2001–2003 (36% of those able to be contacted). A second panel was enrolled from 2004–2006 and consisted of 31,110 participants (25% of those able to be contacted). Of Panel 1 participants, 71% responded to the 2004 and 2007 follow-up surveys, and 55% of Panel 2 participants responded to the 2007 followup survey. The present study included members of the first and second panels who completed a baseline and at least one follow-up questionnaire (n = 80,524). Of the 80,524 participants with at least one follow-up assessment, 18175 (23%) were eligible (several criterias). Of those 2971 (16%) were deleted due to missing data. Also some discussion on non-response bias in MCS in general, and in this sample in particular on p1016 | Response rate of approximately 72%, missing data not mentioned. |
| 1 | 1 | 1 | 1 |
|  | Perform and report results from sensitivity analyses. |  |  |
| 1 | 4 | 1 | 1 |
|  | Do not take into consideration that their data is a weighted sample (risk of incorrect standard errors) The data were weighted to represent all Reserve component personnel, using sampling weights that are a function of age, gender, education, and race/ethnicity. |  |  |
| Yes | Unclear | Unclear | Unclear |
| Before the survey was administered, a survey administrator would read the Human Subjects Protection Committee (HSPC)–approved oral consent script describing the study’s purpose and relevant information about human subjects’ research protections |  | This study was approved by the Naval Health Research Center IRB … (p1011) |  |
| Unclear | Unclear | Unclear | Unclear |
|  | State on page 304: 'These analyses were approved by the Institutional Review Board of the University of North Carolina.' Not clear whether that was a priori. |  |  |
| 5 | 4 | 2 | 5 |
| The only adjustment is weighting so the sample is representative of the current population of marines preparing for deployment (for our purpose it is the post deployment sample) | Discuss confounding factors page 302 and examined the sensitivity of findings to unobserved confounding. Uses post deployment employment and income as predictors of deployment. Otherwise this is a careful analysis that tries to address imbalances by propensity score weighting, and sensitivity analysis. It can be argued however that number of deployments is a poor proxy for exposure to trauma, e.g. military commanders may better observe exposure and decide whether to redeploy based on such information (or type of mission). | All considered and adjusted for + more. No imbalances shown or discussed, except mental health and previous deployment where there are no imbalances by construction. Analysis stratified by time since deployment (Group 1 and 2) | Imbalances shown for age, rank and a number of post deployment variables, nothing controlled for. |
| None | Identified variables that prior research revealed as predictive of deployment. | Based on published litterature (p1010) | None |
| Not considered: gender, mental health history, enlistment status and number of previous deployments. No imbalances shown or discussed | All, except mental health history and number of deployments. More is added (of which two, employment and income are post deployment variables). Uses number of deployment as proxy for exposure (my interpretation, the author discusses in discussion section.) | All considered and adjusted for + more. No imbalances shown or discussed, except mental health and previous deployment where there are no imbalances by construction | Only gender and age and a number of post deployment covariates. Nothing controlled for. |
| None | Full population of guards/reservists. | Select those having no deployments before their baseline questionnaire; screening negative for PTSD, depression, anxiety, or panic predeployment; and no indication of a prior diagnosis of a mental disorder or use of psychotropic medications predeployment. | None |
| Weighting so the sample is representative of the current population of marines preparing for deployment (for our purpose it is the post deployment sample) | Survey-weighted logistic regression; the IPTW (Inverse Probability of Treatment Weights) were used as sampling weights. | Logistic regression | None |
|  |  |  |  |
|  |  |  |  |
|  |  |  |  |
|  |  |  |  |
|  |  |  |  |
|  |  |  |  |
|  |  |  |  |

| **Author** | Foster, E. M. | Gehrman, Seelig, Jacobson, Boyko, Hooper, Gackstetter, Ulmer & Smith |
| --- | --- | --- |
| **Year** | 2011 | 2013 |
| **Sequence Generation** | High | High |
| **Allocation Concealment** | High | High |
| **Blinding** | 4 | 4 |
| Description |  |  |
| **Incomplete Outcome Data** | 3 | 3 |
| Description | Response rate was 55.3%. The data were weighted to represent all Reserve component personnel, using sampling weights that are a function of age, gender, education, and race/ethnicity. | The study’s first panel includes 77,047 participants enrolled from 2001–2003 (36% of those able to be contacted). A second panel was enrolled from 2004–2006 and consisted of 31,110 participants (25% of those able to be contacted). Of Panel 1 participants, 71% responded to the 2004 and 2007 follow-up surveys, and 55% of Panel 2 participants responded to the 2007 follow-up survey. The present study included members of the first and second panels who completed a baseline and at least one follow-up questionnaire (n = 80,524). Of the 80,524 participants with at least one follow-up assessment, 18175 (23%) were eligible (several criterias). Of those 2971 (16%) were deleted due to missing data. Also some discussion on non-response bias in MCS in general, and in this sample in particular on p.1016. |
| **Reporting Bias** | 1 | 1 |
| Description | Perform and report results from sensitivity analyses. |  |
| **Other Bias** | 4 | 1 |
| Description | Do not take into consideration that their data is a weighted sample (risk of incorrect standard errors) The data were weighted to represent all Reserve component personnel, using sampling weights that are a function of age, gender, education, and race/ethnicity. |  |
| **Apriori Protocol** | Unclear | Unclear |
| Description |  | This study was approved by the Naval Health Research Center IRB … (p.1011). |
| **Apriori Plan of Analysis** | Unclear | Unclear |
| Description | State on page 304: 'These analyses were approved by the Institutional Review Board of the University of North Carolina.' Not clear whether that was a priori. |  |
| **Confounding** | 4 | 2 |
| Description | Discuss confounding factors page 302 and examined the sensitivity of findings to unobserved confounding. Uses post deployment employment and income as predictors of deployment. Otherwise this is a careful analysis that tries to address imbalances by propensity score weighting, and sensitivity analysis. It can be argued however that number of deployments is a poor proxy for exposure to trauma, e.g. military commanders may better observe exposure and decide whether to redeploy based on such information (or type of mission). | All considered and adjusted for + more. No imbalances shown or discussed, except mental health and previous deployment where there are no imbalances by construction. Analysis stratified by time since deployment (Group 1 and 2). |
| Relevant confounders described by researchers? | Identified variables that prior research revealed as predictive of deployment. | Based on published literature (p.1010). |
| Assessment of relevant confounders | All, except mental health history and number of deployments. More is added (of which two, employment and income are post deployment variables). Uses number of deployment as proxy for exposure (my interpretation, the author discusses in discussion section.) | All considered and adjusted for + more. No imbalances shown or discussed, except mental health and previous deployment where there are no imbalances by construction. |
| **Method (Design Stage)** | Full population of guards/reservists. | Select those having no deployments before their baseline questionnaire; screening negative for PTSD, depression, anxiety, or panic pre-deployment; and no indication of a prior diagnosis of a mental disorder or use of psychotropic medications pre-deployment. |
| **Method (Analysis Stage)** | Survey-weighted logistic regression; the IPTW (Inverse Probability of Treatment Weights) were used as sampling weights. | Logistic regression |

| Farmer, Vaughan, Garnet & Weinick | Foster, E. M. | Gehrman, Seelig, Jacobson, Boyko, Hooper, Gackstetter, Ulmer & Smith | Gordon, J. G. A. |
| --- | --- | --- | --- |
| 2014 | 2011 | 2013 | 2002 |
| High | High | High | High |
| High | High | High | High |
| 4 | 4 | 4 | 4 |
|  |  |  |  |
| Unclear | 3 | 3 | 2 |
| State on p. 14 that 2620 completed the survey and 355 (12%) declined to participate but the number who passively declined (did not return a blank survey) is unknown. | Response rate was 55.3%. The data were weighted to represent all Reserve component personnel, using sampling weights that are a function of age, gender, education, and race/ethnicity. | The study’s first panel includes 77,047 participants enrolled from 2001–2003 (36% of those able to be contacted). A second panel was enrolled from 2004–2006 and consisted of 31,110 participants (25% of those able to be contacted). Of Panel 1 participants, 71% responded to the 2004 and 2007 follow-up surveys, and 55% of Panel 2 participants responded to the 2007 followup survey. The present study included members of the first and second panels who completed a baseline and at least one follow-up questionnaire (n = 80,524). Of the 80,524 participants with at least one follow-up assessment, 18175 (23%) were eligible (several criterias). Of those 2971 (16%) were deleted due to missing data. Also some discussion on non-response bias in MCS in general, and in this sample in particular on p1016 | Response rate of approximately 72%, missing data not mentioned. |
| 1 | 1 | 1 | 1 |
|  | Perform and report results from sensitivity analyses. |  |  |
| 1 | 4 | 1 | 1 |
|  | Do not take into consideration that their data is a weighted sample (risk of incorrect standard errors) The data were weighted to represent all Reserve component personnel, using sampling weights that are a function of age, gender, education, and race/ethnicity. |  |  |
| Yes | Unclear | Unclear | Unclear |
| Before the survey was administered, a survey administrator would read the Human Subjects Protection Committee (HSPC)–approved oral consent script describing the study’s purpose and relevant information about human subjects’ research protections |  | This study was approved by the Naval Health Research Center IRB … (p1011) |  |
| Unclear | Unclear | Unclear | Unclear |
|  | State on page 304: 'These analyses were approved by the Institutional Review Board of the University of North Carolina.' Not clear whether that was a priori. |  |  |
| 5 | 4 | 2 | 5 |
| The only adjustment is weighting so the sample is representative of the current population of marines preparing for deployment (for our purpose it is the post deployment sample) | Discuss confounding factors page 302 and examined the sensitivity of findings to unobserved confounding. Uses post deployment employment and income as predictors of deployment. Otherwise this is a careful analysis that tries to address imbalances by propensity score weighting, and sensitivity analysis. It can be argued however that number of deployments is a poor proxy for exposure to trauma, e.g. military commanders may better observe exposure and decide whether to redeploy based on such information (or type of mission). | All considered and adjusted for + more. No imbalances shown or discussed, except mental health and previous deployment where there are no imbalances by construction. Analysis stratified by time since deployment (Group 1 and 2) | Imbalances shown for age, rank and a number of post deployment variables, nothing controlled for. |
| None | Identified variables that prior research revealed as predictive of deployment. | Based on published litterature (p1010) | None |
| Not considered: gender, mental health history, enlistment status and number of previous deployments. No imbalances shown or discussed | All, except mental health history and number of deployments. More is added (of which two, employment and income are post deployment variables). Uses number of deployment as proxy for exposure (my interpretation, the author discusses in discussion section.) | All considered and adjusted for + more. No imbalances shown or discussed, except mental health and previous deployment where there are no imbalances by construction | Only gender and age and a number of post deployment covariates. Nothing controlled for. |
| None | Full population of guards/reservists. | Select those having no deployments before their baseline questionnaire; screening negative for PTSD, depression, anxiety, or panic predeployment; and no indication of a prior diagnosis of a mental disorder or use of psychotropic medications predeployment. | None |
| Weighting so the sample is representative of the current population of marines preparing for deployment (for our purpose it is the post deployment sample) | Survey-weighted logistic regression; the IPTW (Inverse Probability of Treatment Weights) were used as sampling weights. | Logistic regression | None |
|  |  |  |  |
|  |  |  |  |
|  |  |  |  |
|  |  |  |  |
|  |  |  |  |
|  |  |  |  |
|  |  |  |  |

| **Author** | Gordon, J. G. A. | Gray G. C., Reed R. J., Kaiser K. S., Smith T. C., Gastanaga V.M. | Harvey S.B., Hatch S.L., Jones M., Hull L., Jones N., Greenberg N., Dandeker C., Fear N.T., Wessely S. |
| --- | --- | --- | --- |
| **Year** | 2002 | 2002 | 2012 |
| **Sequence Generation** | High | High | High |
| **Allocation Concealment** | High | High | High |
| **Blinding** | 4 | 4 | 4 |
| Description |  |  |  |
| **Incomplete Outcome Data** | 2 | 2 | 3 |
| Description | Response rate of approximately 72%, missing data not mentioned. | Response rate 68.6% and missing data 0.1% | Random sample of T and C drawn, response rate phase 1: T: 58% and C: 43% (afterwards reclassification of 0.7% T to C and 19.7% C to T). Response rate Phase 2 of those who participated in phase 1 (used only those who responded in both phases in the analysis): T: 58%, C: 62% |
| **Reporting Bias** | 1 | 1 | 3 |
| Description |  |  | State at page 1183: "post-hoc sensitivity analyses including individuals who had redeployed did not demonstrate any significant alternations from the results presented". Results of this important sensitivity analysis is not reported. |
| **Other Bias** | 1 | 2 | 4 |
| Description |  | Possible recall bias (being asked to recollect onset since 1991 up to 8 years later). Self-reported medical conditions (which we are currently not using) are not from validated instruments. | Almost 20% percent of the non-deployed (according to the registers) were reclassified as deployed based on self-report. |
| **Apriori Protocol** | Unclear | Yes | Yes |
| Description |  | The study was approved by the institutional review board of the Naval Health Research Center (San Diego, California). | The cohort study of UK military personnel was approved by the Ministry of Defence Research Ethics Committee and King’s College Hospital’s local research ethics committee. |
| **Apriori Plan of Analysis** | Unclear | Unclear | Unclear |
| Description |  |  |  |
| **Confounding** | 5 | 4 | 4 |
| Description | Imbalances shown for age, rank and a number of post deployment variables, not controlled for. | Not considered: Mental health history, rank and previous deployment. Current alcohol and smoking controlled for, only small imbalance on alcohol but some imbalance on smoking. Some imbalance on enlistment status and minor on gender. | Random sample but 31% who deployed between phase 1 and 2 were not used in the analysis (not reported divided by T/C). Important confounders not considered. |
| Relevant confounders described by researchers? | No | No | No |
| Assessment of relevant confounders | Only gender and age and a number of post deployment covariates. Nothing controlled for. | Not considered: Mental health history, rank and previous deployment. Current alcohol and smoking controlled for. Some imbalance on enlistment status and minor on gender. | All, except ethnicity, mental health history and number of previous deployments. Some imbalance in rank. In addition response weights constructed to take account of nonresponse according to sex, rank, and age. |
| **Method**  **(Design Stage)** | None | None | Random sample of T and C at Time 1. |
| **Method**  **(Analysis Stage)** | None | Logistic regression | Logistic regression using response weights |

| Gray G. C., Reed R. J., Kaiser K. S., Smith T. C., Gastanaga V.M. | Harvey S.B., Hatch S.L., Jones M., Hull L., Jones N., Greenberg N., Dandeker C., Fear N.T., Wessely S. | Hoge, C. W., Auchterlonie, J. L., & Milliken, C. S. | Holmes, D. T., Tariot, P. N., & Cox, C. |
| --- | --- | --- | --- |
| 2002 | 2012 | 2006 | 1998 |
| High | High | High | High |
| High | High | High | High |
| 4 | 4 | 4 | 4 |
|  |  | Mandatory PDHA |  |
| 2 | 3 | 2 | 4 |
| Response rate 68.6% and missing data 0.1% | Random sample of T and C drawn, response rate Phase 1: T: 58% and C: 43% (afterwards reclassification of 0.7% T to C and 19.7% C to T). Response rate Phase 2 of those who participated in phase 1 (use only those who responded in both phases in the analysis): T: 58%, C: 62% | Study essentially contains entire population of interest. But due to start up problems test scores are missing at the start. "The 18% of service members who did not have a PDHA record (n=66 589) were very similar to those who had a PDHA by deployment location and demographics, except that service members who did not have aPDHA record were somewhat more likely to be active duty Marines (TABLE 2)." (p1027) | Response rate 46%. 57% for the treated and 36% for the comparison. |
| 1 | 3 | 1 | 1 |
|  | State at page 1183: "post-hoc sensitivity analyses including individuals who had redeployed did not demonstrate any significant alternations from the results presented". Results of this important sensitivity analysisd is not reported. | Probably yes |  |
| 2 | 4 | 1 | 2 |
| Possible recall bias (being asked to recollect onset since 1991 up to 8 years later). Self-reported medical condtions (which we are currently not using) are not from validated instruments. | Almost 20% percent of the non-deployed (according to the registers) were reclassified as deployed based on self-report. | Measurements taken shortly after end of deployment. | Recall bias (they ask for mental health history, previous deployments, etc.) |
| Yes | Yes | Unclear | Unclear |
| The study was approved by the institutional review board of the Naval Health Research Center (San Diego, California). | The cohort study of UK military personnel was approved by the Ministry of Defence Research Ethics Committee and King’s College Hospital’s local research ethics committee. | Used administrative data |  |
| Unclear | Unclear | Unclear | Yes |
|  |  |  | An a priori analysis plan is mentioned but not the content or a reference. |
| 4 | 4 | 4 | 5 |
| Not considered: Mental health history, rank and previous deployment. Current alcohol and smoking controlled for, only small imbalance on alcohol but some imbalance on smoking. Some imbalance on enlistment status and minor on gender. | Random sample but 31% who deployed between phase 1 and 2 were not used in the analysis (not reported divided by T/C). Important confounders not considered. | Logistic regression was used  to calculate ORs for outcomes adjusted  for demographic variables shown  in TABLE 1, which are noted in the article  as “adjusted OR.” p1026 which is all except prior mental health, ethnicity, and number of deployments, note large imbalance on enlistment status. | A short (only gender, age, race and rank) descriptive characteristic of the full sample. |
| No | No | No | No |
| Not considered: Mental health history, rank and previous deployment. Current alcohol and smoking controlled for. Some imbalance on enlistment status and minor on gender. | All, except ethnicity, mental health history and number of previous deployments. Some imbalance in rank. In addition response weights constructed to take account of nonresponse according to sex, rank, and age. | All except prior mental health, ethnicity, and number of deployments. | None |
| None | Random sample of T and C at Time 1. | None | None |
| Logistic regression | Logistic regression using response weights | Logistic regression | None |
|  |  |  |  |
|  |  |  |  |
|  |  |  |  |
|  |  |  |  |
|  |  |  |  |
|  |  |  |  |
|  |  |  |  |

| **Author** | Hoge, C. W., Auchterlonie, J. L., & Milliken, C. S. | Holmes, D. T., Tariot, P. N., & Cox, C. | Hotopf, M., David, A., Hull, L., Ismail, K., Unwin, C., & Wessely, S. |
| --- | --- | --- | --- |
| **Year** | 2006 | 1998 | 2003 |
| **Sequence Generation** | High | High | High |
| **Allocation Concealment** | High | High | High |
| **Blinding** | 4 | 4 | 4 |
| Description | Mandatory PDHA |  |  |
| **Incomplete Outcome Data** | 2 | 4 | 2 |
| Description | Study essentially contains entire population of interest. But due to start up problems test scores are missing at the start. "The 18% of service members who did not have a PDHA record (n=66 589) were very similar to those who had a PDHA by deployment location and demographics, except that service members who did not have a PDHA record were somewhat more likely to be active duty marines (TABLE 2)." (p1027) | Response rate 46%. 57% for the treated and 36% for the comparison. | From 7737: Treated response rate: 70.4% (reduced to 64% due to missing data, table 2), comparison response rate: 61.9% reduced to 57% due to missing data, table 2). Responders were older and more likely to be still in service. There was no significant interaction between deployment, late response, and health outcome. 200 servicemen who had not responded after two mailings were randomly chosen for intensive follow-up. The intensive follow-up group contained more discharged personnel were more likely to be married and more likely to have lower educational achievement. They were older and did not differ significantly by medical discharges, employment status, alcohol consumption, and smoking but reported slightly worse health perception (SF-36) than the main study responders. Physical function scales did not, however, differ significantly. |
| **Reporting Bias** | 1 | 1 | 4 |
| Description | Probably yes |  | Do not show imbalances on an important stratification variable (fitness) and do not control for it in the analysis. |
| **Other Bias** | 1 | 2 | 1 |
| Description | Measurements taken shortly after end of deployment. | Recall bias (they ask for mental health history, previous deployments, etc.) |  |
| **Apriori Protocol** | Unclear | Unclear | Unclear |
| Description | Used administrative data |  |  |
| **Apriori Plan of Analysis** | Unclear | Yes | Unclear |
| Description |  | An a priori analysis plan is mentioned but not the content or a reference. |  |
| **Confounding** | 4 | 5 | 3 |
| Description | Logistic regression was used to calculate ORs for outcomes adjusted for demographic variables shown in TABLE 1, which are noted in the article as “adjusted OR” p.1026 which is all except prior mental health, ethnicity, and number of deployments, note large imbalance on enlistment status. | A short (only gender, age, race and rank) descriptive characteristic of the full sample. | Imbalances on the stratification variable Fitness (not explained what it is but apparently thought to somehow relate to predeployment general health) is not shown. Era comparison selected to match Gulf sample (p.409). Hence the Era comparison in this study is a subsample of the original Era sample (compared to Bosnia only). There are big imbalances between treat and comparison. 4 for Bosnia only ES. |
| Relevant confounders described by researchers? | No | No | No |
| Assessment of relevant confounders | All except prior mental health, ethnicity, and number of deployments. | None | All, except ethnicity, mental health history and number of deployments. Imbalances on the stratification variable Fitness is not shown and not controlled for. Notice they do appear to have information on previous deployments (N.I. Tours) but do not control for it. |
| **Method**  **(Design Stage)** | None | None | Stratify on service (Royal Navy, Army, Royal Air Force), sex, age, service status (regular or reservist), rank (officer or other), and fitness (army and air force only). "Since only soldiers we included in the original Bosnia group and there were no reservists we excluded personnel from the Royal Navy and Rayal Air Force and reservist from the Era group." (p.409) |
| **Method**  **(Analysis Stage)** | Logistic regression | None | Logistic regression |

| Hotopf, M., David, A., Hull, L., Ismail, K., Unwin, C., & Wessely, S. | Hotopf, M., Hull, L., Fear, N. T., Browne, T., Horn, O., Iversen, A. et al. | Ikin, J. F., Sim, M. R., Creamer, M. C., Forbes, A. B., McKenzie, D. P., Kelsall, H. L. et al. | Ishoy, T., Suadicani, P., Guldager, B., Appleyard, M., Hein, H. O., & Gyntelberg, F. |
| --- | --- | --- | --- |
| 2003 | 2006 | 2004 | 1999 |
| High | High | High | High |
| High | High | High | High |
| 4 | 4 | 3 | 4 |
|  |  | Psychologists administering CIDI were blind to exposure status. However see p117 1st column. |  |
| 2 | 2 | 3 | 3 |
| From 7737:Treated response rate: 70.4% (reduced to 64% due to missing data, table 2), comparison response rate: 61.9% Reduced to 57% due to missing data, table 2). Responders were older and more likely to be still in service. There was no significant interaction between deployment, late response, and health outcome. 200 serviceman who had not responded after two mailings were randomly chosen for intensive follow-up. The intensive follow-up group contained more discharged personnel, were more likely to be married and more likely to have lower educational achievement. They were older and did not differ significantly by medical discharges, employment status, alcohol consumption, and smoking but reported slightly worse health perception (SF-36) than the main study responders. Physical function scales did not, however, differ significantly. | Participation rate T:62.3% and C 56.3%. Intense followup of 150 randomly selected non-responders (cant find analysis in the text). 5% difference in response rate between regular/reservist in TELIC, but 12 % for Era (comparison). Mention to have done sensitivity analysis but not how and no conclusions follow. Missing data rate varies between 1-2% | Investigated for "participation" bias (non-response). While treatment group had good response rate 1456/1808. Comparison group had lower response: 1588/2796. Additional phone administered smaller questionnaire to non-responders, and sensitivity analysis carried out, table 7, p123. Only minor differences (2.5-5%). Missing data rate T: 3% C: 11% | Response rate for treated: 83.6%, controls: 57.7%. No mentioning of missing data. |
| 4 | 1 | 1 | 1 |
| Do not show imbalances on an important stratification variable (fitness) and do not control for it in the analysis. | Uncertain, but since they report on "adverse effects" it is less likely that outcomes have been excluded. | Probably yes |  |
| 1 | 1 | 1 | 1 |
|  |  | Clinical interview + register data, probably free of recall bias. |  |
| Unclear | Yes | No | Unclear |
|  | First stage of data collection of a planned cohort study comparing mental and physical health outcomes in wot groups: individuals deployed on TELIC 1 and military personnel who did not deploy to TELIC 1. | Retrospective |  |
| Unclear | Unclear | Unclear | Unclear |
|  | Some stratification and oversampling was pre-planned. Stratification on: service, enlistment, oversampling of Era due to medical downgrade and reservists | Probably yes |  |
| 3 | 2 | 3 | 4 |
| Imbalances on the stratification variable Fitness (not explained what it is but apparently thought to somehow relate to predeployment general health) is not shown. Era comparison selected to match Gulf sample (p.409). Hence the Era comparison in this study is a subsample of the original Era sample (compared to Bosnia only). There are big imbalances between treat and comparison. 4 for Bosnia only ES. | Differences between TELIC (n=4722) and Era (n=5550) were examined on confounders. Imbalance on most confounders. Particularly age and medically downgraded but all confounders controlled for except ethnicity | Comparison sample is matched on age, rank, gender, branch, but minor imbalanced . In addition estimates are controlled for marital status and education. Sample turns out to be males only. Duty status unclear (could be all active duty?). (a) type estimates not controlled for previous mental health (but only minor imbalance), but (b) types are. Estimates not controlled for previous deployments (and medium imbalance on previous deployment) but some comparisons made (table 4, searches for specific gulf war effect by conditioning on having been deployed in control). (type a outcomes) 2 (type b outcomes). | Match on age, gender, profession (unclear what profession is) but no imbalances shown or even discussed (gender and age imbalances shown in 1103 and they are okay, but 'profession' not shown or discussed). But table 3 shows balance on mental health outcomes prior to deploy between treat/control. |
| No | p. 1736 | No | None |
| All, except ethnicity, mental health history and number of deployments. Imbalances on the stratification variable Fitness is not shown and not controlled for. Notice they do appear to have information on previous deployments (N.I. Tours) but do not control for it. | Yes (except ethnicity), and more. Imbalances between control/treat. | All considered except duty/enlistment status described. Only adjusted for age, rank, branch (and gender only males) and type b outcomes mental health history as well. | Match on age, gender, profession. |
| Stratify on service (Royal Navy, Army, Royal Air Force), sex, age, service status (regular or reservist), rank (officer or other), and fitness (army and air force only). "Since only soldiers we included in the original Bosnia group and there were no reservists we excluded personnel from the Royal Navy and Rayal Air Force and reservist from the Era group." (p.409) | Stratified sampling | Matched sampling | Matched sampling |
| Logistic regression | Logistic regression | Logistic regression | None |
|  |  |  |  |
|  |  |  |  |
|  |  |  |  |
|  |  |  |  |
|  |  |  |  |
|  |  |  |  |
|  |  |  |  |

| Jones M., Rona R. J., Hooper R., Wesseley S. | Jones N., Thandi G., Fear N. T., Wessely S, Greenberg N | Kelley, M. L., Hock, E., Jarvis, M. S., Smith, K. M., Gaffney, M. A., & Bonney, J. F. | Killgore, W. D. S., Stetz, M. C., Castro, C. A., & Hoge, C. W. |
| --- | --- | --- | --- |
| 2006 | 2014 | 2002 | 2006 |
| High | High | High | High |
| High | High | High | High |
| 4 | 4 | 4 | 4 |
|  |  |  |  |
| 2 | 1 | 2 | Unclear |
| Response rate 65% (long questionnaire), no mentioning of missing data. | Response rate 98% and very few missing data. | Response rate 68% and of these 12% had missing data. | Nothing reported other than: The data were collected as a subset of a larger ongoing longitudinal study on the effects of combat deployment on the mental health and well-being of soldiers. |
| 1 | 1 | 1 | 1 |
|  |  |  |  |
| 3 | 1 | 1 | 1 |
| It is the effect of number of countries deployed to since 1999 (separated by 1 and more than 1 compared to none), thus we do not know the number of deployments. The effects may be a mix of 1 and more deployments. |  |  |  |
| Unclear | Unclear | Unclear | Unclear |
| The study was given ethical appproval by the Defence Medical Services Clinical Research Committee and by King's College Hospital Research Ethics Committee (p 323). | p6 says Ethics approved by Ministry of Defense Research Ethics Committee. Implying they must have laid out their plans for datacollection and analysis. Is it a protocol? |  |  |
| Unclear | Unclear | Unclear | Unclear |
|  |  |  |  |
| 5 | 3 | 5 | 5 |
| Not considered: Mental health history, ethnicity, enlistment status and previous deployment. Current alcohol and smoking controlled for. Imbalances not reported. It is the effect of number of countries deployed to since 1999 (separated by 1 and more than 1 compared to none), thus we do not know the number of deployments or the number of previous deployments. In fact those not deployed since 1999 may have been deployed to the Gulf in 1991. | Not all confounders considered and no imbalances shown or discussed / descriptives not presented. | Nothing discussed | A descriptive characteristic of the full sample. |
| No | Yes, discussion p. 2, column 2. | No | No |
| Not considered: Mental health history, ethnicity, enlistment status and previous deployment. Current alcohol and smoking controlled for. Imbalances not reported. | Not considered: mental health history, branch (or maybe they are all Army?, not stated directly, referred to simply as Armed Forces), and ethnicity. Adjust for enlistment, service length (as a proxy for age and rank), gender, previuos deployment, time on current deployment, combat exposure (excl. IED) and leadership. No imbalances shown or discussed and service length may be a poor proxy for age and rank. | Only gender and branch (as they only include Navy women). Otherwise none of the prespecified confounders are controlled for, however imbalances are reported for all except mental health history, number of deployments, rank and enlistment status. Other confounders are controlled for. | None |
| Stratified by service and size (do not know what they mean by size). | Sampled Armed Forces | None | None |
| Logistic regression | Logistic regression | Regression | None |
|  |  |  |  |
|  |  |  |  |
|  |  |  |  |
|  |  |  |  |
|  |  |  |  |
|  |  |  |  |
|  |  |  |  |

| **Author** | Hotopf, M., Hull, L., Fear, N. T., Browne, T., Horn, O., Iversen, A. et al. | Ikin, J. F., Sim, M. R., Creamer, M. C., Forbes, A. B., McKenzie, D. P., Kelsall, H. L. et al. | Ishoy, T., Suadicani, P., Guldager, B., Appleyard, M., Hein, H. O., & Gyntelberg, F. |
| --- | --- | --- | --- |
| **Year** | 2006 | 2004 | 1999 |
| **Sequence Generation** | High | High | High |
| **Allocation Concealment** | High | High | High |
| **Blinding** | 4 | 3 | 4 |
| Description |  | Psychologists administering CIDI were blind to exposure status. However see p117 1st column. |  |
| **Incomplete Outcome Data** | 2 | 3 | 3 |
| Description | Participation rate T:62.3% and C 56.3%. Intense follow-up of 150 randomly selected non-responders (can’t find analysis in the text). 5% difference in response rate between regular/reservist in TELIC, but 12 % for Era (comparison). Mention to have done sensitivity analysis but not how and no conclusions follow. Missing data rate varies between 1-2% | Investigated for "participation" bias (non-response). While treatment group had good response rate 1456/1808. Comparison group had lower response: 1588/2796. Additional phone administered smaller questionnaire to non-responders, and sensitivity analysis carried out, table 7, p.123. Only minor differences (2.5-5%). Missing data rate T: 3% C: 11%. | Response rate for treated: 83.6%, controls: 57.7%. No mentioning of missing data. |
| **Reporting Bias** | 1 | 1 | 1 |
| Description | Uncertain, but since they report on "adverse effects" it is less likely that outcomes have been excluded. | Probably yes |  |
| **Other Bias** | 1 | 1 | 1 |
| Description |  | Clinical interview + register data, probably free of recall bias. |  |
| **Apriori Protocol** | Yes | No | Unclear |
| Description | First stage of data collection of a planned cohort study comparing mental and physical health outcomes in wot groups: individuals deployed on TELIC 1 and military personnel who did not deploy to TELIC 1. | Retrospective |  |
| **Apriori Plan of Analysis** | Unclear | Unclear | Unclear |
| Description | Some stratification and oversampling was pre-planned. Stratification on: service, enlistment, oversampling of Era due to medical downgrade and reservists | Probably yes |  |
| **Confounding** | 2 | 3 | 4 |
| Description | Differences between TELIC (n=4722) and Era (n=5550) were examined on confounders. Imbalance on most confounders. Particularly age and medically downgraded but all confounders controlled for except ethnicity. | Comparison sample is matched on age, rank, gender, branch, but minor imbalanced. In addition, estimates are controlled for marital status and education. Sample turns out to be males only. Duty status unclear (could be all active duty?). (a) type estimates are not controlled for previous mental health (but only minor imbalance), but (b) types are. Estimates not controlled for previous deployments (and medium imbalance on previous deployment) but some comparisons made (table 4, searches for specific gulf war effect by conditioning on having been deployed in control). (type a outcomes) 2 (type b outcomes). | Match on age, gender, profession (unclear what profession is) but no imbalances shown or even discussed (gender and age imbalances shown in 1103 and they are okay, but 'profession' not shown or discussed). But table 3 shows balance on mental health outcomes prior to deploy between treat/control. |
| Relevant confounders described by researchers? | p. 1736 | No | No |
| Assessment of relevant confounders | Yes (except ethnicity), and more. Imbalances between control/treat. | All considered except duty/enlistment status described. Only adjusted for age, rank, branch (and gender only males) and type b outcomes mental health history as well. | Match on age, gender, profession. |
| **Method**  **(Design Stage)** | Stratified sampling | Matched sampling | Matched sampling |
| **Method**  **(Analysis Stage)** | Logistic regression | Logistic regression | None |

| **Author** | Jones M., Rona R. J., Hooper R., Wesseley S. | Jones N., Thandi G., Fear N. T., Wessely S, Greenberg N | Kelley, M. L., Hock, E., Jarvis, M. S., Smith, K. M., Gaffney, M. A., & Bonney, J. F. |
| --- | --- | --- | --- |
| **Year** | 2006 | 2014 | 2002 |
| **Sequence Generation** | High | High | High |
| **Allocation Concealment** | High | High | High |
| **Blinding** | 4 | 4 | 4 |
| Description |  |  |  |
| **Incomplete Outcome Data** | 2 | 1 | 2 |
| Description | Response rate 65% (long questionnaire), no mentioning of missing data. | Response rate 98% and very few missing data. | Response rate 68% and of these 12% had missing data. |
| **Reporting Bias** | 1 | 1 | 1 |
| Description |  |  |  |
| **Other Bias** | 3 | 1 | 1 |
| Description | It is the effect of number of countries deployed since 1999 (separated by 1 and more than 1 compared to none), thus we do not know the number of deployments. The effects may be a mix of 1 and more deployments. |  |  |
| **Apriori Protocol** | Unclear | Unclear | Unclear |
| Description | The study was given ethical approval by the Defence Medical Services Clinical Research Committee and by King's College Hospital Research Ethics Committee (p.323). | p.6 says Ethics approved by Ministry of Defence Research Ethics Committee. Implying they must have laid out their plans for data collection and analysis. Is it a protocol? |  |
| **Apriori Plan of Analysis** | Unclear | Unclear | Unclear |
| Description |  |  |  |
| **Confounding** | 5 | 3 | 5 |
| Description | Not considered: Mental health history, ethnicity, enlistment status and previous deployment. Current alcohol and smoking controlled for. Imbalances not reported. It is the effect of number of countries deployed to since 1999 (separated by 1 and more than 1 compared to none), thus we do not know the number of deployments or the number of previous deployments. In fact those not deployed since 1999 may have been deployed to the Gulf in 1991. | Not all confounders considered and no imbalances shown or discussed / descriptives not presented. | Nothing discussed |
| Relevant confounders described by researchers? | No | Yes, discussion p. 2, column 2. | No |
| Assessment of relevant confounders | Not considered: Mental health history, ethnicity, enlistment status and previous deployment. Current alcohol and smoking controlled for. Imbalances not reported. | Not considered: mental health history, branch (or maybe they are all Army(?), not stated directly, referred to simply as Armed Forces), and ethnicity. Adjust for enlistment, service length (as a proxy for age and rank), gender, previous deployment, time on current deployment, combat exposure (excl. IED) and leadership. No imbalances shown or discussed and service length may be a poor proxy for age and rank. | Only gender and branch (as they only include Navy women). Otherwise none of the pre specified confounders are controlled for, however imbalances are reported for all except mental health history, number of deployments, rank and enlistment status. Other confounders are controlled for. |
| **Method**  **(Design Stage)** | Stratified by service and size (do not know what they mean by size). | Sampled Armed Forces | None |
| **Method**  **(Analysis Stage)** | Logistic regression | Logistic regression | Regression |

| Kline, A., Falca-Dodson, M., Sussner, B., Ciccone, D. S., Chandler, H., Callahan, L. et al. | Lande, R. G., Marin, B. A., Chang, A. S., & Lande, G. R. | Luxton D. D., Greenburg D., Ryan J., Niven A., Wheeler G., Mysliwiec V. | Macera C. A., Aralis H. J., Highfill-McRoy R., Rauh M. J. |
| --- | --- | --- | --- |
| 2010 | 2008 | 2011 | 2014 |
| High | High | High | High |
| High | High | High | High |
| 4 | 4 | 4 | 4 |
|  |  |  |  |
| 2 | 2 | 2 | Unclear |
| Response rate 95% of these 13% had missing data. | Response rate 84%. Missing data: T: 1-3%, C: 4-5% | All soldiers from a redeploying brigade combat team participated. Non-missing data rate 86.9% | Nothing reported |
| 3 | 3 | 1 | 1 |
| Page 278: we found little difference between those deployed once versus those deployed more than once on most measures of mental health (not shown). | Perform multiple regression analysis for binge drinking but only reports a p-value. (p < 0.001) |  |  |
| 2 | 2 | 1 | 1 |
| Possibly recall bias. Not precisely stated when deployment to Iraq happened previously, but usually 3 year rotation. | Recall bias, as questions focus on preceeding year. |  |  |
| Unclear | Unclear | No | Unclear |
|  |  | This study used existing data and was approved by the Madigan Army Medical Center Department of Clinical Investigation. |  |
| Unclear | Unclear | No | Unclear |
|  | Appears to be convenience sample from Walter Reed Army Medical Center (abstract). | Only outcomes were selected a priori: The medical comorbidities PTSD, depression, mTBI, obesity, and panic syndrome and the high-risk health behaviors abuse of tobacco, abuse of alcohol, and risk for suicide were selected a priori as outcome variables. (page 1191). |  |
| 4 | 5 | 5 | 5 |
| Two important confounders not considered and serious imbalance on previous deployment. Controls for post deployment income. | Nothing | The authors conclusion is: Soldiers who experienced combat are at increased risk for persistent SSD (Short sleep duration) and comorbidities associated with SSD. The purpose of the paper is to evaluate the effect of SSD on the outcomes relevant for the review. | Only gender controlled for (and it is a Navy Marine only sample). |
| No | No | Not really a discussion of relevant confounders but refer to Reger et al. (Reger MA, Gahm GA, Swanson RD, Duma SJ. Association between number of deployments to Iraq and mental health screening outcomes in US Army soldiers. J Clin Psychiatry 2009;70:1266-72.) for similar modeling approach. | No (except gender though) |
| All, except rank and mental history, more are added. | Gender is considered, but no adjustment and large imbalance. | All except ethnicity and mental health history. Post-deployment sleep duration and symptoms of insufficient sleep added. No imbalances are reported, although it is an Army (and probably active-duty) only sample. | Only gender / Separate analyses by gender. |
| Logistic regression | None | Total population (of the specific Brigade combat team). | None |
| Logistic regression | None | Logistic regression | Gender separated univariate logistic regression |
|  |  |  |  |
|  |  |  |  |
|  |  |  |  |
|  |  |  |  |
|  |  |  |  |
|  |  |  |  |
|  |  |  |  |

| **Author** | Killgore, W. D. S., Stetz, M. C., Castro, C. A., & Hoge, C. W. | Kline, A., Falca-Dodson, M., Sussner, B., Ciccone, D. S., Chandler, H., Callahan, L. et al. | Lande, R. G., Marin, B. A., Chang, A. S., & Lande, G. R. |
| --- | --- | --- | --- |
| **Year** | 2006 | 2010 | 2008 |
| **Sequence Generation** | High | High | High |
| **Allocation Concealment** | High | High | High |
| **Blinding** | 4 | 4 | 4 |
| Description |  |  |  |
| **Incomplete Outcome Data** | Unclear | 2 | 2 |
| Description | Nothing reported other than: The data were collected as a subset of a larger ongoing longitudinal study on the effects of combat deployment on the mental health and well-being of soldiers. | Response rate 95% of these 13% had missing data. | Response rate 84%. Missing data: T: 1-3%, C: 4-5% |
| **Reporting Bias** | 1 | 3 | 3 |
| Description |  | Page 278: we found little difference between those deployed once versus those deployed more than once on most measures of mental health (not shown). | Perform multiple regression analysis for binge drinking but only reports a p-value. (p< 0.001) |
| **Other Bias** | 1 | 2 | 2 |
| Description |  | Possibly recall bias. Not precisely stated when deployment to Iraq happened previously, but usually 3 years of rotation. | Recall bias, as questions focus on proceeding year. |
| **Apriori Protocol** | Unclear | Unclear | Unclear |
| Description |  |  |  |
| **Apriori Plan of Analysis** | Unclear | Unclear | Unclear |
| Description |  |  | Appears to be convenience sample from Walter Reed Army Medical Center (abstract). |
| **Confounding** | 5 | 4 | 5 |
| Description | A descriptive characteristic of the full sample. | Two important confounders not considered and serious imbalance on previous deployment. Controls for post deployment income. | Nothing |
| Relevant confounders described by researchers? | No | No | No |
| Assessment of relevant confounders | None | All, except rank and mental history, more are added. | Gender is considered, but no adjustment and large imbalance. |
| **Method**  **(Design Stage)** | None | Logistic regression | None |
| **Method**  **(Analysis Stage)** | None | Logistic regression | None |

| McCaroll, James E., Robert J. Ursano and Carol S. Fullerton | McCarroll J. E., Ursano R. J., Fullerton C. S. | Perconte S.T., Wilson A.T., Pontius E. B., Dietrick A. L., Spiro K. J. | Peterson, A. L., Wong, V., Haynes, M. F., Bush, A. C., & Schillerstrom, J. E. |
| --- | --- | --- | --- |
| 1993 | 1993 | 1993 | 2010 |
| High | High | High | High |
| High | High | High | High |
| 4 | 4 | 4 | 4 |
| Non-blind | Non-blind |  |  |
| Unclear | Unclear | 1 | Unclear |
| Not stated how many were approached (see #20051). Reports sample size of 55 and 56 at followup, but only uses 50 and 50 for analysis. Original sample had T=116, C=118. | Not stated how many were approached. | Response rate 95%. Missing data 2.5% | No mentioning of missing data |
| 3 | 3 | 1 | 1 |
| We used chi square analysis to test the differences between the two gropus for both marital status and parenthood. Neither difference was statiscally significant at time 1 or 2. (p 939) | We examined variables that migh have confounded our findings …. Age, gender, race, and prior experience were not determined to be confounding variables. Yet they show that inexperienced react stronger to IES scale, and sample is imbalanced. |  |  |
| 2 | 2 | 1 | 1 |
| Possible recall bias | Paper mentions recall bias. |  |  |
| Unclear | Unclear | Unclear | Unclear |
| Not explicitly stated | Not explicitly stated | Not stated |  |
| Unclear | Unclear | Unclear | Unclear |
| Not stated | Not stated | Not stated |  |
| 5 | 5 | 5 | 3 |
| Consider age and gender (there is some imbalance on gender). Does not consider or display balance between exposed/non-exposed groups for other relevant confounders (but do assert balance on 2 other confounders: marital status and parenthood). No confounders controlled for. | Imbalanced on gender, and experience (~prior trauma); but balanced on the other confounders they report on: age, marital status, number of children. Further reports (but not substantiated) on p1876 that gender, race and prior experience is not confounding. | Only gender is considered, analysis also divided on race, and prior combat exposure (vietnam vs non-vietnam). Univariate. No discussion of balance, no tables. | Compares Iraq deployed (high combat exposure) to Quatar deployed (low combat exposure)Do not adjust for anything but there are no imbalances. Some imbalance on spciality codes (p677). They exclude previously deployed to either location. |
| p939 (briefly) | p1876 (briefly) | Not stated | No |
| Reported: age, marital status, parenthood and gender. | Reported: age, maritial status, gender. Not reported: number of children, race. | Gender separated means only. | All, except ethnicity and mental health history. |
| None | None | None | None |
| None | None | ANOVA | None |
|  |  |  |  |
|  |  |  |  |
|  |  |  |  |
|  |  |  |  |
|  |  |  |  |
|  |  |  |  |
|  |  |  |  |

| **Author** | Luxton D. D., Greenburg D., Ryan J., Niven A., Wheeler G., Mysliwiec V. | Macera C. A., Aralis H. J., Highfill-McRoy R., Rauh M. J. | McCaroll, James E., Robert J. Ursano and Carol S. Fullerton |
| --- | --- | --- | --- |
| **Year** | 2011 | 2014 | 1993 |
| **Sequence Generation** | High | High | High |
| **Allocation Concealment** | High | High | High |
| **Blinding** | 4 | 4 | 4 |
| Description |  |  | Non-blind |
| **Incomplete Outcome Data** | 2 | Unclear | Unclear |
| Description | All soldiers from a redeploying brigade combat team participated. Non-missing data rate 86.9% | Nothing reported | Not stated how many were approached (see #20051). Report sample size of 55 and 56 at follow-up, but only uses 50 and 50 for analysis. Original sample had T=116, C=118. |
| **Reporting Bias** | 1 | 1 | 3 |
| Description |  |  | We used chi square analysis to test the differences between the two groups for both marital status and parenthood. Neither difference was statistically significant at time 1 or 2. (p.939) |
| **Other Bias** | 1 | 1 | 2 |
| Description |  |  | Possible recall bias |
| **Apriori Protocol** | No | Unclear | Unclear |
| Description | This study used existing data and was approved by the Madigan Army Medical Center Department of Clinical Investigation. |  | Not explicitly stated |
| **Apriori Plan of Analysis** | No | Unclear | Unclear |
| Description | Only outcomes were selected a priori: The medical comorbidities PTSD, depression, mTBI, obesity, and panic syndrome and the high-risk health behaviours, abuse of tobacco, abuse of alcohol, and risk for suicide were selected a priori as outcome variables. (page 1191). |  | Not stated |
| **Confounding** | 5 | 5 | 5 |
| Description | The authors conclusion is: Soldiers who experienced combat are at increased risk for persistent SSD (Short sleep duration) and comorbidities associated with SSD. The purpose of the paper is to evaluate the effect of SSD on the outcomes relevant for the review. | Only gender controlled for (and it is a Navy Marine only sample). | Consider age and gender (there is some imbalance on gender). Does not consider or display balance between exposed/non-exposed groups for other relevant confounders (but do assert balance on 2 other confounders: marital status and parenthood). No confounders controlled for. |
| Relevant confounders described by researchers? | Not really a discussion of relevant confounders but refer to Reger et al. (Reger MA, Gahm GA, Swanson RD, Duma SJ. Association between number of deployments to Iraq and mental health screening outcomes in US Army soldiers. J Clin Psychiatry (2009;70:1266-72) for similar modelling approach. | No (except gender though) | p.939 (briefly) |
| Assessment of relevant confounders | All except ethnicity and mental health history. Post-deployment sleep duration and symptoms of insufficient sleep added. No imbalances are reported, although it is an Army (and probably active-duty) only sample. | Only gender / Separate analyses by gender. | Reported: age, marital status, parenthood and gender. |
| **Method**  **(Design Stage)** | Total population (of the specific Brigade combat team). | None | None |
| **Method**  **(Analysis Stage)** | Logistic regression | Gender separated univariate logistic regression | None |

| Pierce, P. F. | Polusny, M. A., Erbes, C. R., Arbisi, P. A., Thuras, P., Kehle, S. M., Rath, M. et al. | Polusny, M. A., Erbes, C. R., Murdoch, M., Arbisi, P. A., Thuras, P., & Rath, M. B. | Proctor, S. P., Heaton, K. J., Dos Santos, K. D., Rosenman, E. S., & Heeren, T. |
| --- | --- | --- | --- |
| 2005 | 2009 | 2011 | 2009 |
| High | High | High | High |
| High | High | High | High |
| 4 | 4 | 4 | 4 |
|  |  |  |  |
| 4 | Unclear | Unclear | 2 |
| Treated response rate 52% and comparison response rate 45%. Otherwise missing data level not mentioned except: Only subjects with complete data are reported in this analysis. "We examined whether the characteristics of the nonrespondents differed systematically from those of the respondents. The response rate was not related to parental status or component of the Air Force. However, on average, the respondents held slighly higher ranks, compares with the nonrespondents." (p351). 2400 Air Force women sampled whereof 1164 completed the study (p351). Table II reports demographics for n=691, while analysis uses n=698. No explanation for discrepancies. | Nothing reported, in 2696 it is stated that precise participation rates could not be obtained. | Time 2 of sample used in 2695, response rate 81% | 75.3% follow-up participation rate (Response both at time 1 and 2). 171 soldiers volunteered at Time 1 (93 depl/78 non depl), 119 completed (67 depl/52 non). Responserate: depl: 72%, non: 67%. No major differences in Time 1 baseline characteristics (eg, age, education, marital status) or outcome measures were observed between the participating and non-participating groups. (Results are available on request from the first author.) |
| 4 | 1 | 1 | 3 |
| Relevant confounders only reported separated by active duty/reserve or guard, and n=691, whereas number who completed study was 1164. |  |  | Perform sensitivity analysis. Do not report results other than state it did not change the results. Do not show imbalances (in their analysis they use a subgroup of the control group). |
| 1 | 1 | 3 | 1 |
|  |  | Number of participants unsure, stated on page 689 n=424 and in table 2 and n=349. |  |
| Unclear | Yes | Yes | Yes |
|  | The RINGS study (Readiness and Resilience in National Guard Soldiers) was approved by the human subject research review boards of the Army Department of Veterans Affairs, University of Minnesota, and relevant ArmyNational Guard (ARNG) command (p.354 and #2696). | The RINGS study (Readiness and Resilience in National Guard Soldiers) was approved by the human subject research review boards of the Army Department of Veterans Affairs, University of Minnesota, and relevant ArmyNational Guard (ARNG) command (p.689). | Sample size requirements for this study were estimated a priori. (page 351) |
| Unclear | Unclear | Unclear | No |
|  |  |  | We assumed that the actual analyses would control for additional variables, resulting in increased power to detect differences or the ability to detect smaller effect sizes. (page 352) |
| 3 | 4 | 4 | 5 |
| Compares Air Force women deployed in the theater of operations with women deployed elsewhere, matched with respect to component (active duty or Reserve/Guard) and parental status (parent or not a parent). Have access to other confounders but do not adjust and do not report imbalances. | Army National Guard sample, otherwise nothing is controlled for. Age, ethnicity and rank is considered, only minor imbalances except on rank (table 1, p 355). | Army National Guard sample, of pre-specified confounders only baseline PTSD symptoms is controlled for (and in addition a number of pre-deployment factors such as prior life stressors etc.) High risk of multicollinarity. Include three different combat exposure measures in the model. | The overall control group is nondeployed (activated and nonactivated). In the analysis they use only the nonactivated (n decreases from 52 to 19. "A subset of non-deployers (N=19) served within the US on activated status ... " p351.). The nonactivated are most likely a very selective comparison (do not show separate characteristic of the two control groups). |
| Based on previous experience and recommandations of military and civilian scientific groups. | No | Yes, briefly discussed on page 688. | None, except their sensitivity analysis is inspired by what have been shown to influence aspects of mood (do not report results of the sensitivity analysis other than state it did not change the results). |
| All, except number of deployments, do not report imbalances. | Army National Guard sample. Age, ethnicity and rank is considered, only minor imbalances, except on rank. | Army National Guard all without prior PTSD sample, of pre-specified confounders only baseline PTSD symptoms is controlled for (and in addition a number of pre-deployment factors such as prior life stressors etc.) Gender, age race, rank considered, and gender age, race controlled for. Relevant balance on exposure not displayed. Include three different combat exposure measures in the model. | All, except ethnicity, mental history, rank and number of previous deployments. Education is added. Do not show the relevant imbalances. They do have information about prior deployments. Also they include Time 1 POMS score (essentially controlling for prior mental health). Problematic that they control for Time 2 unit cohesion (possibly an effect of deployment) (footnote to table 3). They have information on age, rank, education, reading score, ethnciity, marital status (see table 1, p354). But do not control in final models. And do not show balance for the smaller non-deployed comparison (N=52-19) |
| Air Force women only, match on theater/non-theater, component (active duty or Reserve/Guard) and parental status. | None | Unclear | The study incorporated a cluster-sampling design, with participants sampled within military unit groups. The biggest issue with this study is the sampling procedure: "At time 1; 171 soldiers ... volunteered [my emphasis] to participate in the study" (p351). |
| None | None | Hierarchical logistic regression | Generalized estimating equation (GEE) |
|  |  |  |  |
|  |  |  |  |
|  |  |  |  |
|  |  |  |  |
|  |  |  |  |
|  |  |  |  |
|  |  |  |  |

| **Author** | McCarroll J. E., Ursano R. J., Fullerton C. S. | Perconte S.T., Wilson A.T., Pontius E. B., Dietrick A. L., Spiro K. J. | Peterson, A. L., Wong, V., Haynes, M. F., Bush, A. C., & Schillerstrom, J. E. |
| --- | --- | --- | --- |
| **Year** | 1993 | 1993 | 2010 |
| **Sequence Generation** | High | High | High |
| **Allocation Concealment** | High | High | High |
| **Blinding** | 4 | 4 | 4 |
| Description | Non-blind |  |  |
| **Incomplete Outcome Data** | Unclear | 1 | Unclear |
| Description | Not stated how many were approached. | Response rate 95%. Missing data 2.5% | No mentioning of missing data. |
| **Reporting Bias** | 3 | 1 | 1 |
| Description | We examined variables that might have confounded our findings. Age, gender, race, and prior experience were not determined to be confounding variables. Yet they show that inexperienced react stronger to IES scale, and sample is imbalanced. |  |  |
| **Other Bias** | 2 | 1 | 1 |
| Description | Paper mentions recall bias. |  |  |
| **Apriori Protocol** | Unclear | Unclear | Unclear |
| Description | Not explicitly stated | Not stated |  |
| **Apriori Plan of Analysis** | Unclear | Unclear | Unclear |
| Description | Not stated | Not stated |  |
| **Confounding** | 5 | 5 | 3 |
| Description | Imbalanced on gender, and experience (~prior trauma); but balanced on the other confounders they report on: age, marital status, number of children. Further reports (but not substantiated) on p.1876 that gender, race and prior experience is not confounding. | Only gender is considered, analysis also divided on race, and prior combat exposure (Vietnam vs non-Vietnam). Univariate. No discussion of balance, no tables. | Compares Iraq deployed (high combat exposure) to Quatar deployed (low combat exposure). Do not adjust for anything but there are no imbalances. Some imbalance on speciality codes (p677). They exclude previously deployed to either location. |
| Relevant confounders described by researchers? | p.1876 (briefly) | Not stated | No |
| Assessment of relevant confounders | Reported: age, marital status, gender. Not reported: number of children, race. | Gender separated means only. | All, except ethnicity and mental health history. |
| **Method**  **(Design Stage)** | None | None | None |
| **Method**  **(Analysis Stage)** | None | ANOVA | None |

| Riddle J. R., Smith T. C., Smith B., Corbeil T. E., Engel C. C., Wells T. S., Hoge C. W., Adkins J., Zamorski M., Blazer D. | Riviere L. A., Kendall-Robbins A., McGurk D., Castro C. A., Hoge C. W. | Shen, Y. C., Arkes, J., Kwan, B. W., Tan, L. Y., & Williams, T. V. | Shen Y.C., Arkes, J., Pilgrim J. |
| --- | --- | --- | --- |
| 2007 | 2011 | 2009 | 2009 |
| High | High | High | High |
| High | High | High | High |
| 4 | 4 | 4 | 4 |
| Non-blind | Non-blind |  |  |
| 3 | 3 | Unclear | Unclear |
| Demographic data almost complete (76476 out of 77047). Response rate 36% | Response rate 3127/5378 at 3mths and 2449/3470 at 12 months. Restricts sample to those who had only deployed once to Iraq. Bringing sample to 2539 at 3mths, and 1495 at 12mths. No comparison with non-responders. | Not mentioned specifically, but us administrative records. TRICARE unlikely to have missing records. CTS unclear. | Nothing reported. Probably as this is a mandatory survey, missing outcome data is very uncommon. |
| 1 | 1 | 2 | 2 |
|  |  | Focuses only on PTSD. Strategy is to harvest PTSD diagnosis from TRICARE. Possible that they have also investigated other outcomes. | In a sensitivity analysis, we limit our estimation to those sailors who only appear once in the sample, and our results remain the same. (not shown) |
| 1 | 1 | 1 | 1 |
|  | Anonymously surveyed (p136). |  |  |
| Yes | Yes | Unclear | Unclear |
| Protocol NHRC.2000.0007 (p200) | Data were collected under a Walter Reed Army Institute of Research Institutional Review Board approval (p137). | Unlikely |  |
| Unclear | Unclear | Unclear | Unclear |
| Not stated | Not explicitly stated |  |  |
| 5 | 5 | 3 | 4 |
| Balance on deployment condition not demonstrated nor discussed. Conditions on measurements which can be considered outcomes. | Bad controls. Likely outcomes of deployment like jobloss is used as predictors of PTSD/Depression. The regression specification makes it difficult to interpret exposure as dose-response relationship. No discussion of confounder balance by exposure. | Control for a wide variety of characteristics; sample of active duty only and separate estimates by branch. Do not control for mental history and number of previous deployments. Do not show or discuss imbalances. | Three confounders not controlled for (age, mental health history and duty/enlistment status), not all imbalances are shown.20% have multiple deployments. Data is representative of sailors who have been deployed to field missions (which can occur both on ship or on ground). No discussion of exogeneity of deployment assignment. |
| Yes, p194 | Yes, table 1, p138. | See discussion on p.8-9 | No |
| All except: prior mental health; several additional controls; all measured post-deployment. | Final model adjusted for: age, gender, rank and social functioning outcomes. | Yes, except mental health history. Additional relevant controls. | All, except age, mental health history and duty/enlistment status, more is added. Not all imbalances shown. |
| None | None | Restricting sample to CTS sample military population. | Sailors on routine shipboard operation without field operation are exempted from filling out the survey. Data is representative of sailors who have been deployed to field missions (which can occur both on ship or on ground) |
| Logistic regression | Logistic regression | Logistic regression + year dummies | Probit model with robust SEs as 20% have multiple observations. P219 says they adjust for clustering. Not stated which option in Stata is used: cluster(var) or robust. |
|  |  |  |  |
|  |  |  |  |
|  |  |  |  |
|  |  |  |  |
|  |  |  |  |
|  |  |  |  |
|  |  |  |  |

| **Author** | Pierce, P. F. | Polusny, M. A., Erbes, C. R., Arbisi, P. A., Thuras, P., Kehle, S. M., Rath, M. et al. | Polusny, M. A., Erbes, C. R., Murdoch, M., Arbisi, P. A., Thuras, P., & Rath, M. B. |
| --- | --- | --- | --- |
| **Year** | 2005 | 2009 | 2011 |
| **Sequence Generation** | High | High | High |
| **Allocation Concealment** | High | High | High |
| **Blinding** | 4 | 4 | 4 |
| Description |  |  |  |
| **Incomplete Outcome Data** | 4 | Unclear | Unclear |
| Description | Treated response rate 52% and comparison response rate 45%. Otherwise missing data level not mentioned except: Only subjects with complete data are reported in this analysis. "We examined whether the characteristics of the nonrespondents differed systematically from those of the respondents. The response rate was not related to parental status or component of the Air Force. However, on average, the respondents held slightly higher ranks, compared with the nonrespondents." (p.351). 2400 Air Force women sampled whereof 1164 completed the study (p.351). Table II reports demographics for n=691, while analysis uses n=698. No explanation for discrepancies. | Nothing reported, in 2696 it is stated that precise participation rates could not be obtained. | Time 2 of sample used in 2695, response rate 81% |
| **Reporting Bias** | 4 | 1 | 1 |
| Description | Relevant confounders only reported separated by active duty/reserve or guard, and n=691, whereas number who completed study was 1164. |  |  |
| **Other Bias** | 1 | 1 | 3 |
| Description |  |  | Number of participants unsure, stated on page 689 n=424 and in table 2 and n=349. |
| **Apriori Protocol** | Unclear | Yes | Yes |
| Description |  | The RINGS study (Readiness and Resilience in National Guard Soldiers) was approved by the human subject research review boards of the Army Department of Veterans Affairs, University of Minnesota, and relevant Army National Guard (ARNG) command (p.354 and #2696). | The RINGS study (Readiness and Resilience in National Guard Soldiers) was approved by the human subject research review boards of the Army Department of Veterans Affairs, University of Minnesota, and relevant Army National Guard (ARNG) command (p.689). |
| **Apriori Plan of Analysis** | Unclear | Unclear | Unclear |
| Description |  |  |  |
| **Confounding** | 3 | 4 | 4 |
| Description | Compares Air Force women deployed in the theater of operations with women deployed elsewhere, matched with respect to component (active duty or Reserve/Guard) and parental status (parent or not a parent). Have access to other confounders but do not adjust and do not report imbalances. | Army National Guard sample, otherwise nothing is controlled for. Age, ethnicity and rank is considered, only minor imbalances except on rank (table 1, p.355). | Army National Guard sample, of pre-specified confounders only baseline PTSD symptoms is controlled for (and in addition a number of pre-deployment factors such as prior life stressors etc.) High risk of multicollinearity. Include three different combat exposure measured in the model. |
| Relevant confounders described by researchers? | Based on previous experience and recommendations of military and civilian scientific groups. | No | Yes, briefly discussed on page 688. |
| Assessment of relevant confounders | All, except number of deployments, do not report imbalances. | Army National Guard sample. Age, ethnicity and rank is considered, only minor imbalances, except on rank. | Army National Guard all without prior PTSD sample, of pre-specified confounders only baseline PTSD symptoms is controlled for (and in addition a number of pre-deployment factors such as prior life stressors etc.) Gender, age race, rank considered, and gender age, race controlled for. Relevant balance on exposure not displayed. Include three different combat exposure measured in the model. |
| **Method**  **(Design Stage)** | Air Force women only, match on theater/non-theater, component (active duty or Reserve/Guard) and parental status. | None | Unclear |
| **Method**  **(Analysis Stage)** | None | None | Hierarchical logistic regression |

| **Author** | Proctor, S. P., Heaton, K. J., Dos Santos, K. D., Rosenman, E. S., & Heeren, T. | Riddle J. R., Smith T. C., Smith B., Corbeil T. E., Engel C. C., Wells T. S., Hoge C. W., Adkins J., Zamorski M., Blazer D. | Riviere L. A., Kendall-Robbins A., McGurk D., Castro C. A., Hoge C. W. |
| --- | --- | --- | --- |
| **Year** | 2009 | 2007 | 2011 |
| **Sequence Generation** | High | High | High |
| **Allocation Concealment** | High | High | High |
| **Blinding** | 4 | 4 | 4 |
| Description |  | Non-blind | Non-blind |
| **Incomplete Outcome Data** | 2 | 3 | 3 |
| Description | 75.3% follow-up participation rate (response both at time 1 and 2). 171 soldiers volunteered at Time 1 (93 depl/78 non depl), 119 completed (67 depl/52 non). Response rate: depl: 72%, non: 67%. No major differences in Time 1 baseline characteristics (eg, age, education, marital status) or outcome measures were observed between the participating and non-participating groups. (Results are available on request from the first author) | Demographic data almost complete (76476 out of 77047). Response rate 36%. | Response rate 3127/5378 at 3mths and 2449/3470 at 12 months. Restricts sample to those who had only deployed once to Iraq. Bringing sample to 2539 at 3mths, and 1495 at 12mths. No comparison with non-responders. |
| **Reporting Bias** | 3 | 1 | 1 |
| Description | Perform sensitivity analysis. Do not report results other than state it. Did not change the results. Do not show imbalances (in their analysis they use a subgroup of the control group). |  |  |
| **Other Bias** | 1 | 1 | 1 |
| Description |  |  | Anonymously surveyed (p.136) |
| **Apriori Protocol** | Yes | Yes | Yes |
| Description | Sample size requirements for this study were estimated a priori. (p. 351) | Protocol NHRC.2000.0007 (p.200) | Data were collected under a Walter Reed Army Institute of Research Institutional Review Board approval (p.137). |
| **Apriori Plan of Analysis** | No | Unclear | Unclear |
| Description | We assumed that the actual analyses would control for additional variables, resulting in increased power to detect differences or the ability to detect smaller effect sizes. (page 352) | Not stated | Not explicitly stated |
| **Confounding** | 5 | 5 | 5 |
| Description | The overall control group is nondeployed (activated and nonactivated). In the analysis they use only the nonactivated (n decreases from 52 to 19. "A subset of non-deployers (N=19) served within the US on activated status ... " p.351.). The nonactivated are most likely a very selective comparison (do not show separate characteristic of the two control groups). | Balance on deployment condition not demonstrated nor discussed. Conditions on measurements which can be considered outcomes. | Bad controls. Likely outcomes of deployment like job-loss is used as predictors of PTSD/depression. The regression specification makes it difficult to interpret exposure as dose-response relationship. No discussion of confounder balance by exposure. |
| Relevant confounders described by researchers? | None, except their sensitivity analysis is inspired by what have been shown to influence aspects of mood (do not report results of the sensitivity analysis other than state it did not change the results). | Yes, p.194 | Yes, table 1, p.138 |
| Assessment of relevant confounders | All, except ethnicity, mental history, rank and number of previous deployments. Education is added. Do not show the relevant imbalances. They do have information about prior deployments. Also they include Time 1 POMS score (essentially controlling for prior mental health). Problematic that they control for Time 2 unit cohesion (possibly an effect of deployment) (footnote to table 3). They have information on age, rank, education, reading score, ethnicity, marital status (see table 1, p.354). But do not control in final models. And do not show balance for the smaller non-deployed comparison (N=52-19). | All except: prior mental health; several additional controls; all measured post-deployment. | Final model adjusted for: age, gender, rank and social functioning outcomes. |
| **Method**  **(Design Stage)** | The study incorporated a cluster-sampling design, with participants sampled within military unit groups. The biggest issue with this study is the sampling procedure: "At time 1; 171 soldiers ... volunteered [my emphasis] to participate in the study" (p.351). | None | None |
| **Method**  **(Analysis Stage)** | Generalized estimating equation (GEE) | Logistic regression | Logistic regression |

| Smith T.C., Ryan M.A., Wingard D.L., Slymen D. J., Sallis J. F., Kritz-Silverstein D., Millennium Cohort Study Team | Street A. E., Gradus J.L., Giasson H.L., Vogt D., Resick P.A. | Stuart, J. A. & Bliese, P. D. | Sundin J., Herrell R.K., Hoge C.W., Fear N.T., Adler A.B., Greenberg N., Riviere L. A., Thomas J.L., Wessely S., Bliese P.D. |
| --- | --- | --- | --- |
| 2008 | 2013 | 1998 | 2014 |
| High | High | High | High |
| High | High | High | High |
| 4 | 4 | 4 | 4 |
|  |  |  |  |
| 2 | 3 | 4 | Unclear |
| Baseline response rate 36%, followup response rate 71%. Further exclude those who where deployed before submission of baseline questionnaire (2230 or 4%) or while deployed (621 or 1%) and those who submitted follow up questionnaire while deployed (1986 or 4%). Missing data 0.01%. Further removed those with prior PTSD (1861 or 4% of those left). | Response rate 48.6%. Minor diff. To non-responders, except age, responders 4 years older. / They reweight for non-response to recover population prevalence. | Response rate 31%. Of these 50% had missing data and was not included in the analysis. No comparison of responders versus nonresponders. | US response rate 90.1% and few non-deployed excluded. UK response rate 72% (from snowball) and further some added and some deleted and further limited to regular enlisted army male personnel who had deployed to Iraq in 2007-2008 so in reality it is unclear. |
| 1 | 1 | 4 | 1 |
|  |  | Do not show or mention imbalances |  |
| 2 | 1 | 1 | 1 |
| Possibly recall bias. Questionnaire measures combat exposure as self-reported exposure to witnessing death, abuse, maimed soldiers or civilians, prisoners of war, or refugees, in the past 3 years. |  |  |  |
| Yes | Yes | Unclear | No |
| This research has been conducted in compliance with all applicable federal regulations governing the protection of human subjects in research (Protocol NHRC.2000.007). | p s557 |  | Data are from other studies. |
| Unclear | Unclear | Unclear | Unclear |
|  |  |  |  |
| 3 | 4 | 4 | 3 |
| All confounders controlled for but imbalances only shown for the total sample and not divided by branch as the analyses. For the total sample there are some imbalances for gender, age, enlistment status and occupation and further previous deployment and mental health is not very precise, but controls for smoking/drinking problems @baseline. | They have all (except prior mental health) relevant confounders but do not use them. | Only Army reservists included. Otherwise confounders controlled for in regression and imbalances not reported. | Not considered are only mental health history and ethnicity, but no adjustments are made and some imbalances on age, rank and number of deployments. UK score 5 and US 3. |
| No | From the litterature | Discussion on page 6 | No |
| It is stated on page 8: "Analyses included adjustment for baseline characteristics including sex, age, education, marital status, race/ethnicity, rank, service component, occupation, cigarette smoking, and problem alcohol drinking". Further they remove those with deployment before submission of baseline questionnaire and those with prior PTSD. So all confounders are controlled for although these two last not in a very precise manner | Gender separated analysis. State that no confounders identified (Check if confounders are significant and then do not include them) and only report non adjusted. | All, except mental health history and number of deployments + current life stressor. | Not considered: mental health history and ethnicity. Regular enlisted army male personnel only. Age, rank, number of deployments, education, marital status, years in service and career intentions staying in service shown by high/low combat exposure. Nothing controlled for. Imbalance on age, education, UK rank, years in service (minor), number of deployments (UK large, US minor) and career intentions (UK large, US small). |
| Population based survey (Millenium Cohort) | Gender stratified | Stratification on location of deployment, mission type (combat, combat support, or service). | Unclear/nothing |
| Logistic regression | Logistic regression; Check if confounders are significant and then do not include them. | Hierarchical linear regression | None |
|  |  |  |  |
|  |  |  |  |
|  |  |  |  |
|  |  |  |  |
|  |  |  |  |
|  |  |  |  |
|  |  |  |  |

| **Author** | Shen, Y. C., Arkes, J., Kwan, B. W., Tan, L. Y., & Williams, T. V. | Shen Y.C., Arkes, J., Pilgrim J. | Simmons, R., Maconochie, N., & Doyle, P. |
| --- | --- | --- | --- |
| **Year** | 2009 | 2009 | 2004 |
| **Sequence Generation** | High | High | High |
| **Allocation Concealment** | High | High | High |
| **Blinding** | 4 | 4 | 4 |
| Description |  |  |  |
| **Incomplete Outcome Data** | Unclear | Unclear | 4 |
| Description | Not mentioned specifically, but us administrative records. TRICARE unlikely to have missing records. CTS unclear. | Nothing reported. Probably as this is a mandatory survey, missing outcome data is very uncommon. | Discussion on page 7. Response rates among men were 53% for GWV and 42% for NGWV. Ninety percent of the reasons (for not responding) given by both GWV and NGWV for earlier non-response related to such things as not remembering receiving a questionnaire, thinking they had sent it back, or general 'mistrust' of the MoD. It might therefore be concluded that response bias relating to adverse health outcomes was not large in this study (this is the authors conclusion, do not fully agree). |
| **Reporting Bias** | 2 | 2 | 1 |
| Description | Focuses only on PTSD. Strategy is to harvest PTSD diagnosis from TRICARE. Possible that they have also investigated other outcomes. | In a sensitivity analysis, we limit our estimation to those sailors who only appear once in the sample, and our results remain the same. (not shown) | Probably yes |
| **Other Bias** | 1 | 1 | 3 |
| Description |  |  | They mention recall bias (p.7), coding based on free text. It is not described whether coders were blinded. Measures not validated. |
| **Apriori Protocol** | Unclear | Unclear | No |
| Description | Unlikely |  | Retrospective |
| **Apriori Plan of Analysis** | Unclear | Unclear | Unclear |
| Description |  |  | Probably no, main purpose of study was reproduction and child health |
| **Confounding** | 3 | 4 | 4 |
| Description | Control for a wide variety of characteristics; sample of active duty only and separate estimates by branch. Do not control for mental history and number of previous deployments. Do not show or discuss imbalances. | Three confounders not controlled for (age, mental health history and duty/enlistment status), not all imbalances are shown. 20% have multiple deployments. Data is representative of sailors who have been deployed to field missions (which can occur both on ship or on ground). No discussion of exogeneity of deployment assignment. | Matched comparison sample. Adjusts for age at survey, service, rank, serving status (at time of survey)*, smoking status*, current alcohol consumption*. NB! * marked can be considered effects/outcomes of deployment. |
| Relevant confounders described by researchers? | See discussion on p.8-9 | No | No |
| Assessment of relevant confounders | Yes, except mental health history. Additional relevant controls. | All, except age, mental health history and duty/enlistment status, more is added. Not all imbalances shown. | All except ethnicity, mental health history and previous deployment |
| **Method (Design Stage)** | Restricting sample to CTS sample military population. | Sailors on routine shipboard operation without field operation are exempted from filling out the survey. Data is representative of sailors who have been deployed to field missions (which can occur both on ship or on ground) | Matched sampling (sex, age, service, rank, status, fitness to deploy) |
| **Method (Analysis Stage)** | Logistic regression + year dummies | Probit model with robust SEs as 20% have multiple observations. P.219 says they adjust for clustering. Not stated which option in Stata is used: cluster(var) or robust. | Logistic regression |

| **Author** | Simms, L. J., Watson, D., & Doebbeling, B. N. | Skopp N.A., Reger M.A., Reger G.M., Mishkind M.C., Raskind M., Gahm G.A. | Skotnicka J. |
| --- | --- | --- | --- |
| **Year** | 2002 |  | 2013 |
| **Sequence Generation** | High | High | High |
| **Allocation Concealment** | High | High | High |
| **Blinding** | 4 | 4 | 4 |
| Description |  | non-blind |  |
| **Incomplete Outcome Data** | 2 | 1 | Unclear |
| Description | Response rate 91% | Mandatory surveys, less than 1% non-compliers (p 278). | Nothing reported |
| **Reporting Bias** | 3 | 1 | 1 |
| Description | Do not show imbalances |  |  |
| **Other Bias** | 1 | 1 | 1 |
| Description |  | Exposure completed shortly after return to US. |  |
| **Apriori Protocol** | Unclear | Yes | Unclear |
| Description |  | Data collected under a protocol for a different project (p.278). |  |
| **Apriori Plan of Analysis** | Unclear | Unclear | Unclear |
| Description |  | Not stated, but see page 278. |  |
| **Confounding** | 4 | 2 | 5 |
| Description | No reporting on imbalances other than: Compared with the nondeployed group, deployed participants were more likely to be younger, male, enlisted personnel (vs. officers), Army or Marines, and less educated. There were no differences between deployed and nondeployed participants in terms of race, income, current unemployment, or active/reserve status. | Adjusted for all confounders except rank (although considered). Drops non-significant confounders from final model. | Soldiers only, otherwise only mention age and education and there are imbalances. Simple t-tests used. Uses a comparison group of non-deployed, but no description of how it was chosen, and it is certainly not matched. Large imbalances. |
| Relevant confounders described by researchers? | All, except mental health history and number of deployments. | Yes, very carefully described on page 279. | No |
| Assessment of relevant confounders | None | All controlled for except rank (initially, final model not). Rank considered. | Soldiers only, otherwise only mention age and education and there are imbalances. |
| **Method (Design Stage)** | Stratified sampling on deploy/non-deploy, and National Guard/Active. Within each of these 4 domains stratified random sampling on age, sex, rank and branch. | None | None |
| **Method (Analysis Stage)** | None | Logistic regression | None |

| **Author** | Smith T.C., Ryan M.A., Wingard D.L., Slymen D. J., Sallis J. F., Kritz-Silverstein D., Millennium Cohort Study Team | Street A. E., Gradus J.L., Giasson H.L., Vogt D., Resick P.A. | Stuart, J. A. & Bliese, P. D. |
| --- | --- | --- | --- |
| **Year** | 2008 | 2013 | 1998 |
| **Sequence Generation** | High | High | High |
| **Allocation Concealment** | High | High | High |
| **Blinding** | 4 | 4 | 4 |
| Description |  |  |  |
| **Incomplete Outcome Data** | 2 | 3 | 4 |
| Description | Baseline response rate 36%, follow-up response rate 71%. Further exclude those who were deployed before submission of baseline questionnaire (2230 or 4%) or while deployed (621 or 1%) and those who submitted follow up questionnaire while deployed (1986 or 4%). Missing data 0.01%. Further removed those with prior PTSD (1861 or 4% of those left). | Response rate 48.6%. Minor diff. To non-responders, except age, responders 4 years older. / They reweight for non-response to recover population prevalence. | Response rate 31%. Of these 50% had missing data and was not included in the analysis. No comparison of responders versus nonresponders. |
| **Reporting Bias** | 1 | 1 | 4 |
| Description |  |  | Do not show or mention imbalances. |
| **Other Bias** | 2 | 1 | 1 |
| Description | Possibly recall bias. Questionnaire measures combat exposure as self-reported exposure to witnessing death, abuse, maimed soldiers or civilians, prisoners of war, or refugees, in the past 3 years. |  |  |
| **Apriori Protocol** | Yes | Yes | Unclear |
| Description | This research has been conducted in compliance with all applicable federal regulations governing the protection of human subjects in research (Protocol NHRC.2000.007). | p s557 |  |
| **Apriori Plan of Analysis** | Unclear | Unclear | Unclear |
| Description |  |  |  |
| **Confounding** | 3 | 4 | 4 |
| Description | All confounders controlled for but imbalances only shown for the total sample and not divided by branch as the analyses. For the total sample there are some imbalances for gender, age, enlistment status and occupation and further previous deployment and mental health is not very precise, but controls for smoking/drinking problems @baseline. | They have all (except prior mental health) relevant confounders but do not use them. | Only Army reservists included. Otherwise confounders controlled for in regression and imbalances not reported. |
| Relevant confounders described by researchers? | No | From the literature | Discussion on page 6 |
| Assessment of relevant confounders | It is stated on page 8: "Analyses included adjustment for baseline characteristics including sex, age, education, marital status, race/ethnicity, rank, service component, occupation, cigarette smoking, and problem alcohol drinking". Further they remove those with deployment before submission of baseline questionnaire and those with prior PTSD. So all confounders are controlled for although these two last not in a very precise manner. | Gender separated analysis. State that no confounders identified (check if confounders are significant and then do not include them) and only report non adjusted. | All, except mental health history and number of deployments + current life stressor. |
| **Method (Design Stage)** | Population based survey (Millenium Cohort) | Gender stratified | Stratification on location of deployment, mission type (combat, combat support, or service). |
| **Method (Analysis Stage)** | Logistic regression | Logistic regression; Check if confounders are significant and then do not include them. | Hierarchical linear regression |

| **Author** | Sundin J., Herrell R.K., Hoge C.W., Fear N.T., Adler A.B., Greenberg N., Riviere L. A., Thomas J.L., Wessely S., Bliese P.D. | Sutker P. B., Davis J. M., Uddo M., Ditta S. R. | Sutker, P. B., Uddo, M., Brailey, K., & Allain, J. |
| --- | --- | --- | --- |
| **Year** | 2014 | 1995 | 1993 |
| **Sequence Generation** | High | High | High |
| **Allocation Concealment** | High | High | High |
| **Blinding** | 4 | 4 | 4 |
| Description |  | Assessment protocol constituted one component of a full day of psychological debriefing exercises conducted in small groups by mental health professionals upon invitation of military units (p.418). |  |
| **Incomplete Outcome Data** | Unclear | 4 | Unclear |
| Description | US response rate 90.1% and few non-deployed excluded. UK response rate 72% (from snowball) and further some added and some deleted and further limited to regular enlisted army male personnel who had deployed to Iraq in 2007-2008 so in reality it is unclear. | Out of 1423 troops, 511 were excluded from data analyses: 365 did not complete all instruments relevant to the investigation, and 155 scored less than 85 on the measure of intelligence … (p.417). "Missing data on the relevant instruments resulted from varying time periods allotted for debriefing across units, minor difference in the assessment battery, and individual failures to complete all instruments. The final sample of 912 did not differ in the distribution of personal history characteristics from the overall sample of 1423 troops ..." (p.417) | Treated missing data rate 30%, comparison rate not reported. They state that: Subjects excluded from analysis (because of missing data) did not differ from included war-zone troops on descriptive variables such as age, ethnicity, gender, rank, level of education, or total number of days deployed to the Persian Gulf. |
| **Reporting Bias** | 1 | 3 | 2 |
| Description |  | ODS-SE (a stress exposure scale) used as instrument for combat-exposure (p.418), but no reporting. | Have non reported data on previous military service/combat exposure and location, prior mental health contacts, as well as marital status, occupation, number of children, and living arrangements currently and prior to deployment. |
| **Other Bias** | 1 | 1 | 1 |
| Description |  |  |  |
| **Apriori Protocol** | No | Unclear | Unclear |
| Description | Data are from other studies | Not explicitly stated. |  |
| **Apriori Plan of Analysis** | Unclear | Unclear | Unclear |
| Description |  | Not explicitly stated. |  |
| **Confounding** | 3 | 5 | 5 |
| Description | Not considered are only mental health history and ethnicity, but no adjustments are made and some imbalances on age, rank and number of deployments. UK score 5 and US 3. | Compares war zone deployed reservists to reservists deployed state-side. Unclear that this is a valid comparison. While the authors note a host of confounders (p.416), they do not control for these in the warzone/stateside contrast, while noting that the distribution of characteristics are not identical. | Do not control for anything but report significant differences from ANOVA analyses of the confounders, some imbalances although not shown for all confounders. Arbitrary median division of war-zone stress groups (footnote 1 page 44). Table 2, p.41 seems to indicate balance in the deployed group (low/high). |
| Relevant confounders described by researchers? | No | Yes, table 1, p 418. Confounders described: Age, Education, Ethnicity, Rank, Sex. | No |
| Assessment of relevant confounders | Not considered: mental health history and ethnicity. Regular enlisted army male personnel only. Age, rank, number of deployments, education, marital status, years in service and career intentions staying in service shown by high/low combat exposure. Nothing controlled for. Imbalance on age, education, UK rank, years in service (minor), number of deployments (UK large, US minor) and career intentions (UK large, US small). | Table 1 suggests imbalances. Confounders not considered: branch, number of previous deployments, prior mental health. | All, except mental health history and number of previous deployments are considered, some imbalances on gender, age and rank, mention significant ethnicity differences from ANOVA analysis but do not show imbalance. |
| **Method (Design Stage)** | Unclear/nothing | None | None |
| **Method (Analysis Stage)** | None | None | None |

| **Author** | Sutker, P. B., Uddo, M., Brailey, K., Vasterling, J. J., and Errera, P. | Tackett D. P. | Toomey, R., Kang, H. K., Karlinsky, J., Baker, D. G., Vasterling, J. J., Alpern, R. et al. |
| --- | --- | --- | --- |
| **Year** | 1994 | 2011 | 2007 |
| **Sequence Generation** | High | High | High |
| **Allocation Concealment** | High | High | High |
| **Blinding** | 4 | 4 | 4 |
| Description |  |  |  |
| **Incomplete Outcome Data** | 1 | Unclear | 2 |
| Description | 5% missing data | Only stated how many completed the survey (and a few of them were excluded). | Random, stratified sample and follow up to the National Health Survey of Gulf War Era Veterans and Their Families (1995). Participation rate 53% for treated and 39% for control although: Because of the lower participation rates by non-deployed veterans, more people in this category (n=799) were recruited to achieve the desired sample size of 1000 per group. Participation bias is examined: Overall, the degree of participation bias was independent of deployment status (page 387). Missing data varies but is less than 1%. |
| **Reporting Bias** | 1 | 1 | 4 |
| Description |  |  | Of the outcomes 10 years after deployment only PTSD adjusted OR is reported, for the remaining unadjusted prevalence rates are available. |
| **Other Bias** | 1 | 1 | 1 |
| Description |  |  | Gulf War onset outcomes may be biased due to recall bias as they are diagnosed 8-10 years after / On the other hand it is CAPS + CIDI. 5 may be a little harsh. 3 (only Gulf War onset outcomes) |
| **Apriori Protocol** | Unclear | Yes | Unclear |
| Description | Probably yes given quasi random sampling (every 3rd name, p. 384) and clinician administered SCID. | Page 53 |  |
| **Apriori Plan of Analysis** | Unclear | Unclear | Unclear |
| Description |  |  |  |
| **Confounding** | 3 | 5 | 4 |
| Description | Only Army reservists included. Otherwise no confounders are controlled for. Imbalance in age. Their incidence measure "controls" for prior mental health by excluding those from the base. | Include self-efficacy, social support and spirituality in the regression. Also available one with resilience included. Otherwise nothing controlled for. Balance not considered. | Imbalances shown for gender and age, the remaining just stated there were a difference. (and 5 for illicit substance dependence Table 1) |
| Relevant confounders described by researchers? | No | No | No |
| Assessment of relevant confounders | All, except number of deployments (prior deployments only in the ODS deployed subset, p.384). | Guards only sample. Otherwise none of the relevant confounders considered but self-efficacy, social support and spirituality included in the regression. | All, except mental health history and number of previous deployments and education is added. Further: Candidate covariates were deleted for particular models when they caused computational problems preventing model calculation. Education not controlled for in Gulf onset PTSD. Age and rank not controlled for in illicit substance dependence. Outcomes 10 years after, except PTSD, nothing is controlled for. |
| **Method (Design Stage)** | None | None | The sampling design is a stratified random sample with unequal probabilities of selection within combinations of the strata: deployment status, gender and duty type (active service v. reserve or National Guard). |
| **Method (Analysis Stage)** | None | Regression | Stratification and logistic regression. |

| **Author** | Trautmann S., Schönfeld S., Behrendt S., Höfler M., Zimmermann P., Wittchen H. U. | Unwin, C., Blatchley, N., Coker, W., Ferry, S., Hotopf, M., Hull, L. et al. | Unwin, C., Hotopf, M., Hull, L., Ismail, K., David, A., & Wessely, S. |
| --- | --- | --- | --- |
| **Year** | 2014 | 1999 | 2002 |
| **Sequence Generation** | High | High | High |
| **Allocation Concealment** | High | High | High |
| **Blinding** | 4 | 4 | 4 |
| Description |  |  |  |
| **Incomplete Outcome Data** | 1 | 2 | 2 |
| Description | Deployed: Response rate 92.8%. Missing data 0.4%. Non-deployed: response rate 95.4%, no missing data. | Treated response rate: 70.4% (reduced to 64% due to missing data, table 2), comparison response rate: 61.9% reduced to 57% due to missing data, table 2). Responders were older and more likely to be still in service. There was no significant interaction between deployment, late response, and health outcome. 200 servicemen who had not responded after two mailings were randomly chosen for intensive follow-up. The intensive follow-up group contained more discharged personnel, were more likely to be married and more likely to have lower educational achievement. They were older and did not differ significantly by medical discharges, employment status, alcohol consumption, and smoking but reported slightly worse health perception (SF-36) than the main study responders. Physical function scales did not, however, differ significantly. | From 7737: Treated response rate: 70.4% (reduced to 64% due to missing data, table 2), comparison response rate: 61.9% Reduced to 57% due to missing data, table 2). Responders were older and more likely to be still in service. There was no significant interaction between deployment, late response, and health outcome. 200 servicemen who had not responded after two mailings were randomly chosen for intensive follow-up. The intensive follow-up group contained more discharged personnel, were more likely to be married and more likely to have lower educational achievement. They were older and did not differ significantly by medical discharges, employment status, alcohol consumption, and smoking but reported slightly worse health perception (SF-36) than the main study responders. Physical function scales did not, however, differ significantly. |
| **Reporting Bias** | 1 | 4 | 4 |
| Description |  | Do not show imbalances on important stratification variables (fitness and branch of service) and do not control for them in the analysis (as they do with other stratification variables where also imbalances are shown. Actual numbers used in the analyses are not reported (the numbers reported are obviously incorrect for the treated and also incorrect for comparison if it is correct that the analyses only include men as stated in the text). | Do not show imbalances on an important stratification variables (fitness and branch) and do not control for it in the analysis. |
| **Other Bias** | 1 | 1 | 1 |
| Description |  |  |  |
| **Apriori Protocol** | Yes | Unclear | Unclear |
| Description | EK72022010 |  |  |
| **Apriori Plan of Analysis** | Unclear | Unclear | Unclear |
| Description |  |  |  |
| **Confounding** | 2 | 4 | 4 |
| Description | All except mental health history and ethnicity considered. Stratified comparison sample. | Imbalances on the stratification variables Fitness (not explained what it is but apparently thought to somehow relate to predeployment general health) and branch of service are not shown. In addition, they control for a number of factors which can be considered an effect of deployment: current smoking, current alcohol consumption, civilian/military status on follow-up. (see table 4) | Imbalances on the stratification variables branch and Fitness (not explained what it is but apparently thought to somehow relate to predeployment general health) are not shown and not controlled for. Large imbalance on the stratification variable enlistment status but not controlled for. Add a number of post deployment variables fi alcohol and employment. |
| Relevant confounders described by researchers? | From the literature | None, except a confusing discussion of healthy warrior effect in the 'Study limitations'-section: 'The associations between the Gulf War and Era cohorts were only slightly more robust than those between the Gulf War and Bosnia cohorts, which suggests that the “healthy warrior” effect was not strong. We controlled for predeployment fitness, a proxy for general health.' Fitness to deploy should be seen as a proxy for the unmeasured underlying health of soldiers. However, they do not control for fitness in e.g. table7. They argue that if the healthy warrior effect is present they would underestimate the effect of deployment. (p.176, col2). (relevant) Confounders (apparently) controlled for: age, rank (and gender since male only). | None |
| Assessment of relevant confounders | Not considered: mental health history and ethnicity. Soldiers only sample (guess they are all Army and not reservists) and use never deployed as comparison. Some imbalance on rank. In addition control for marital status, economic situation (OBS post variable), education, length of service and unit (combat, medical and other). | All, except ethnicity, mental health history and number of deployments. Imbalances on the stratification variables Fitness and branch of service are not shown and not controlled for. | All, except ethnicity, branch, mental health history and number of deployments. Imbalances on the stratification variables branch and Fitness is not shown and not controlled for. A number of post deployment variables added. |
| **Method (Design Stage)** | Stratified (age, gender and unit) random selection of never deployed. | Stratify on service (Royal Navy, Army, Royal Air Force), sex, age, service status (regular or reservist), rank (officer or other), and fitness (army and air force only). | Stratify on service (Royal Navy, Army, Royal Air Force), sex, age, service status (regular or reservist), rank (officer or other), and fitness (army and air force only). |
| **Method (Analysis Stage)** | Logistic regression with post-stratification weights. | Logistic regression | Logistic regression |

| **Author** | Vanderploeg R. D., Belanger H. G., Horner R. D., Spehar A. M., Powell-Cope G., Luther S.L., Scott S.G. | Vasterling, J. J., Proctor, S. P., Amoroso, P., Kane, R., Heeren, T., & White, R. F. | Voelker, M. D., Saag, K. G., Schwartz, D. A., Chrischilles, E., Clarke, W. R., Woolson, R. F. et al. |
| --- | --- | --- | --- |
| **Year** | 2012 | 2006 | 2004 |
| **Sequence Generation** | High | High | High |
| **Allocation Concealment** | High | High | High |
| **Blinding** | 3 | 3 | 4 |
| Description | Anonymous self-reports. | Assessments were conducted at military installations by a civilian examiner team. All performance-based neuropsychological measures were individually administered according to scripted, standardized instructions. Participants completed the paper and pencil surveys in small groups. Examiners and participants were typically aware of each participant’s anticipated deployment status at time 1 and actual deployment status at time 2. |  |
| **Incomplete Outcome Data** | 4 | 2 | 2 |
| Description | Of 10400 letters mailed, approximately 700 letters were returned for insufficient addresses and 4006 individuals responded for a response rate of 41.3%. "Because of anonymity it was not possible to compare respondents with non-respondents" (p.1888). We excluded 423 who did not fully complete the survey, 371 who completed the survey more than 1 time, and 113 who gave inconsistent or impossible responses, resulting in a final sample of 3098" (p.1889). | Time 1: N=1368 (out of 1457= 94%). Assessed again at time 2 if at same installation (N=1028), 75% of time 1 sample. Final sample at time 2: 961 (6.5% excluded due to missing data). Among the 661 missing values for specific items on questionnaires (occurring in 3% of cases) were replaced by the mean value of the individual’s completed items for that measure if the participant responded to at least 50% of the items. If fewer than 50% of the items on a measure were completed, summary scores were not computed. Outliers were truncated at 3 SDs from the mean. Baseline characteristics and differences between time 2 respondents and nonrespondents were examined via t test or 2 test, as appropriate. | Randomly drawn sample. Response rate 76%. Iowa Gulf War Study (analysed elsewhere). |
| **Reporting Bias** | 1 | 1 | 3 |
| Description |  | Uncertain, but since they report on "adverse effects" it is less likely that outcomes have been excluded. | Their procedure to remove covariates in a stepwise fashion is not very transparent. |
| **Other Bias** | 2 | 1 | 1 |
| Description | Web based survey (possible bias in responders/non-responders). |  |  |
| **Apriori Protocol** | Yes | Unclear | Yes |
| Description | Protocol was approved by IRB of USF (p.1888). | Prospective, cohort-controlled design measuring subjective and objective neuropsychological outcomes in US Army soldiers deploying to Iraq. But note not for PTSD. | See Doebbeling et al., 2002. |
| **Apriori Plan of Analysis** | No | No | Yes |
| Description | Analysis is exploratory (p.1889) | Analysis not designed for PTSD | See Doebbeling et al., 2002 |
| **Confounding** | 5 | 2 | 4 |
| Description | Control and treat are imbalanced and several confounders not considered. | To capture heterogeneous deployment experiences and geographic separation within the war zone, unit selection was based on a modified categorization procedure. Deploying and nondeploying units represented combat, combat support, and combat service support functions and were well matched in these attributes. Note for POMS depression (table 5) they control for time 1 value + gender, age, education, but also time 2 sleep and alcohol use (bad controls). (3 for table 5 POMS measures) | Only minor imbalances on stratification variables. Imbalance on mental health history not reported. Stepwise regression excluding relevant confounders. |
| Relevant confounders described by researchers? | Yes, table 1, p.1890. Confounders described: Gender, race, prior psych trauma and enlistment status. | No, but dedicate p. 523 and table 3 to comparing T and C. | Yes: Theoretical models provided a framework to examine contributors to and potential confounders in the relation between deployment and HRQL (page 904). |
| Assessment of relevant confounders | The deployed group differed from the nondeployed group in several characteristics … (p.1889). Age, rank, branch and prior deployment not considered. Note that a host of deployment related experiences are controlled for: physical injury, traumatic experiences, Blasts, Mild TBI. | Sampling technique ensures high balance on: Age, race, Gender, Rank, previous deployment, prior mental health, and additional: education, tenure in army, marriage, sleep, alcohol consumption, cigarettes, medication, psychoactive consumption, developmental disorder, psychiatric disorder, past alcohol use disorder, prior head injury, other neuro-medical disorder. This is established at baseline (Time 1). | All, except age, gender, rank, enlistment status and number of previous deployments. More is added. They have these variables but exclude some in a stepwise fashion. |
| **Method (Design Stage)** | None | Cohort-controlled study | Stratification |
| **Method (Analysis Stage)** | Logistic regression | None | Stratification and linear stepwise regression (factors with univariate p values of 0.25 or less included). |

| **Author** | Vinokur, A. D., Pierce, P. F., Lewandowski-Romps, L., Hobfoll, S. E., & Galea, S. | Wells, T. S., LeardMann, C. A., Fortuna, S. O., Smith, B., Smith, T. C., Ryan, M. A. K. et al. | Wolfe, J., Proctor, S. P., Erickson, D. J., Heeren, T., Friedman, M. J., Huang, M. T. et al. |
| --- | --- | --- | --- |
| **Year** | 2011 | 2010 | 1999 |
| **Sequence Generation** | High | High | High |
| **Allocation Concealment** | High | High | High |
| **Blinding** | 4 | 4 | 4 |
| Description |  |  |  |
| **Incomplete Outcome Data** | 3 | 2 | 3 |
| Description | Of the 2250 men and women who were invited to the study, 1451 completed the telephone interview, and 1009 provided data using the mailed SAQ (60%), or its equivalent online (40%). […] Of the 1009 men and women completing the initial Time 1 (June 2005) SAQ, 796 also completed the follow-up time 2 SAQ (page 7). | Response rate 71.4%, of these 27% had missing data or were excluded: We excluded participants from the final sample if, at baseline, they reported ever having a diagnosis for depression, met the criteria for mild or other depression, or reported taking medicine for anxiety, stress, or depression (12%). We also excluded those completing the baseline questionnaire after deployment (3%), completing either questionnaire while on deployment (5%), or not answering questions on depression diagnosis or symptoms or use of medication for anxiety, stress, or depression (7%). Additionally only 31% of the invited cohort members replied (but see discussion p.97). | See discussion p.533. Differences between non-responders and responders. |
| **Reporting Bias** | 1 | 1 | 4 |
| Description |  |  | Table 5 footnote. "Results remain similar after controlling for age, sex, education, active duty status, …. " |
| **Other Bias** | 1 | 2 | 1 |
| Description |  | Possibly recall bias. Questionnaire measures combat exposure as self-reported responses to. "… at least one combat experience in the past 3 years, including witnessing death, trauma, prisoners of war, or refugees" (p.92-93). |  |
| **Apriori Protocol** | Unclear | Yes | Unclear |
| Description |  | This research has been conducted in compliance with all applicable federal regulations governing the protection of human subjects in research, and was approved by the institutional review board, Naval Health Research Center, San Diego, CA (Protocol NHRC.2000.007). |  |
| **Apriori Plan of Analysis** | Unclear | Unclear | Unclear |
| Description |  |  |  |
| **Confounding** | 5 | 2 | 4 |
| Description | Correlations only. Balance not reported/discussed. | Because of known associations between smoking and depression and smoking and deployment, there were concerns these relations may interact. We investigated this by entering a first order multiplicative interaction term of deployment by smoking status into the regression model. We performed regression diagnostics, including examining covariates for multicollinearity and goodness-of-fit tests. No interaction was observed between smoking status and deployment status, nor was there interaction between baseline PTSD symptoms or diagnosis and deployment as related to depression. Since they ask about current smoking behaviour, they use an outcome of deployment as independent variable. They could have controlled for smoking status at baseline (like they do with alcohol). Rather large imbalances at baseline. Healthy warrior type argument (p.97): "Conversely, men and women who deployed and did not report combat exposures were at lower risk for depression than nondeployed men and women. These findings support hypotheses that stress associated with combat may lead to depression, and that stress related to deployment, in the absence of combat exposure, may be mitigate by selective deployment of service members who are at decreased risk for the development of depression in comparison with nondeployed men and women." | Adjust for stratification variables (weights), they do collect demographics: "gender, age, education level, race, marital status, military rank, military status, and prior military service" (p.534), but not included in regressions. |
| Relevant confounders described by researchers? | No | The literature on the subject is discussed | No |
| Assessment of relevant confounders | Age, gender, race, rank, enlistment status considered (table 1, p8), but not corrected for. | All and more is added |  |
| **Method (Design Stage)** | None | Population based survey (Millennium Cohort) | Stratification |
| **Method (Analysis Stage)** | None | Logistic regression | SUDAAN |

## Risk of Bias: Updated search

| **Author** | **Adams, Nikitin , Wooten, Larson** | **Afari ,Pittman , Floto , Owen, Hossain et al.** | **Balderrama-Durbin, Stanton, Snyder, Cigrang , Talcott et al** |
| --- | --- | --- | --- |
| **Year** | 2016 | 2015 | 2017 |
| **Sequence generation** | High | High | High |
| **Allocation concealment** | High | High | High |
| **Blinding** | 4 | 4 | 4 |
| **Incomplete outcome data** | Unclear | Unclear | Unclear |
| **Description** | Nothing reported. However because the PDHA is mandatory and taken within 60 days of return missing outcomes are likely low. In addition authors report few missing covariates (1%, p359) | Nothing reported, uses a subset of the total survey | Nothing reported, uses a subset of the total survey |
| **Selective reporting** | 1 | 1 | 1 |
| **Description** |  |  |  |
| **Other bias** | 1 | 1 | 1 |
| **Description** |  |  |  |
| **A priori protocol** | No | No | No |
| **A priori analysis plan** | No | No | No |
| **Confounding** | 3 | 5 | 5 |
| **Description** | All (and more) except mental health history controlled for and only women and separate analysis on Active Duty and National Reserve/Guard. One bad control (TBI). No imbalances shown or discussed | Gender divided analysis, otherwise only means and SD for those with (and without) combat exposure | Correlation with no covariates |
| **Relevant confounders described** | Women and Army only. Divide analysis (and results) on Active duty and National Reserve/Guard. Models adjust for fiscal-year end date of index deployment, age, race, marital status, education rank, occupation, prior deployment and TBI. | Gender divided, otherwise nothing controlled for | All have a partner |
| **Method used for controlling for confounding (At design state)** | Active duty, female, Army members returning from OEF/OIF deployments ending in fiscal years (FYs) 2008–2011. Within 60 days of the end of an index deployment. Same data as Mustillo et al. (2015); although this sample is women only, however they are included in Mustillo et al. (2015). | None | None |
| **Method used for controlling for confounding (At analysis stage)** | Multivariate logistic regression (1, 2 or 3+ combat exposures vs none) | None | None |

| **Author** | **Boasso, Steenkamp, Nash, Larson , Litz** | **Boulos, Zamorski** | **Britt, Herleman , Odle-Dusseau, Moore,Castro , Hoge** |
| --- | --- | --- | --- |
| **Year** | 2015 | 2016 | 2016 |
| **Sequence generation** | High | High | High |
| **Allocation concealment** | High | High | High |
| **Blinding** | 4 | 4 | 4 |
| **Incomplete outcome data** | 4 | 2 | 4 |
| **Description** | More than 25% (250/869) deleted list wise due to missing one or more measurements. | Response rate 80%. List wise deletion was used for missing values, resulting in the exclusion of 0.9% to 3.6% of respondents (p66S) | 98% response rate but matching per cent only 18% across time points |
| **Selective reporting** | 1 | 1 | 1 |
| **Description** |  |  |  |
| **Other bias** | 1 | 1 | 1 |
| **Description** |  |  |  |
| **A priori protocol** | No | No | No |
| **A priori analysis plan** | No | No | No |
| **Confounding** | 5 | 5 | 5 |
| **Description** | Only state there were no differences (between tertiles of combat exposure) except in age but do not show or control for any covariates (although it is male Marines only). | Some imbalances (except child abuse) and all are controlled for. Do not report the time since return for those in the control group who were deployed (elsewhere), neither the time since return for those in the treatment group (Afghanistan deployed) who were deployed elsewhere too. Include bad controls (education, marital status, income and difficulty meeting basic experiences). | Correlations (active combat vs. not, passive combat vs. not) (and a structural model with perceived benefits of deployment). No description of differences based on stratification of combat exposure. |
| **Relevant confounders described** | Only: There were no predeployment differences between the upper, middle, and lower tertiles on race, education, PTSD, depression, anxiety, or overall functional capacities and disability. At predeployment, Marines in the middle tertile were younger than those in the lower tertile, F(389)=7.12, p=.008; and had fewer prior deployments than those in the upper tertile, F(409) = 5.89, p = .016. | Individuals with an Afghanistan-related deployment were more likely to be male, older, of white racial background, in higher household income groupings, in higher ranks, in the Army, in a married or common-law relationship, and previously deployed on non-Afghanistan-related missions outside North America, and they were less likely to be in higher education groupings. Mental health history not considered but exposure to child abuse where there is no imbalance. Include bad controls (education, marital status, income and difficulty meeting basic experiences are all measured post deployment) |  |
| **Method used for controlling for confounding (At design state)** | None | Afghanistan deployed (and in addition 57% deployed elsewhere too) vs. not Afghanistan deployed (29% deployed elsewhere). |  |
| **Method used for controlling for confounding (At analysis stage)** | None, although as reported data was balanced on the tertiles | Logistic regression |  |

| **Author** | Crum-Cianflone , Powell , LeardMann , Russell, Boyko | de Silva, Varuni ,Jayasekera , Hanwella | Dursa, Barth, Schneiderman, Bossarte |
| --- | --- | --- | --- |
| **Year** | 2016 | 2016 | 2016 |
| **Sequence generation** | High | High | High |
| **Allocation concealment** | High | High | High |
| **Blinding** | 4 | 4 | 4 |
| **Incomplete outcome data** | Unclear | Unclear | 2 |
| **Description** | Of those invited, 31,110 (25% of the contacted group) joined the study. A total of 17,152 completed both baseline (June 2004–February 2006) and follow-up surveys (June 2007–December 2008). Excluded those deployed before baseline (n = 6,402). Additionally, those with missing exposure or covariate data were excluded (n = 79), resulting in a sample size of 10,671. Further removed those with prior PTSD, numbers not reported | Nothing reported | The veterans invited to participate in the 2012 follow up study were all 28,374 living panel members of the original sample of 30,000 contacted in 1995. Response from Gulf: 8,104; Gulf Era: 6,148 (50% response) |
| **Selective reporting** | 1 | 1 | 1 |
| **Description** |  |  |  |
| **Other bias** | 1 | 1 | 1 |
| **Description** |  |  |  |
| **A priori protocol** | No | No | No |
| **A priori analysis plan** | Unclear | No | Unclear |
| **Confounding** | 4 | 5 | 4 |
| **Description** | All confounders considered; some imbalances. Only control for age and gender. Report incidence rates weighted for age and sex using U.S. military population proportions in October 2003. Separate by non-deployers, deployed with combat, deployed without combat | Adjust for age, marital status and educational status, but no imbalances shown or discussed. Report results only for 10 different exposures separately |  |
| **Relevant confounders described** | All considered | Adjust for age, marital status and education but no imbalances shown or discussed | Two important confounders not considered and one not controlled for. Controls for problem drinking, which is possibly itself an outcome of deployment. None or only small imbalances. No discussion of exogeneity assumption of deployment assignment. |
| **Method used for controlling for confounding (At design state)** | Population based survey (Millennium Cohort) | None | No |
| **Method used for controlling for confounding (At analysis stage)** | Report incidence rates weighted for age and sex | Logistic regression | All, except previous deployment and mental health history. Imbalance on age; otherwise only minor imbalances on covariates. Odds ratios were adjusted for age, sex, race, service branch, unit, BMI, and smoking status. Last 2 are bad controls |

| **Author** | **Fink,Cohen , Sampson , Gifford , Fullerton et al.** | **Fissette** | **Hougsnæs , Bøe , Dahl , Reichelt** |
| --- | --- | --- | --- |
| **Year** | 2016 | 2016 | 2017 |
| **Sequence generation** |  | High | High |
| **Allocation concealment** |  | High | High |
| **Blinding** |  | 4 | 4 |
| **Incomplete outcome data** |  | 4 | 4 |
| **Description** |  | Of the 318 available, 164 provided both pre and post tests and were analysed (48% missing/attrition) | The response rate was 59%, but only the 3403 men (47.5%) who completed all items of the four different screening instruments were used to define cases with mental health problems were included |
| **Selective reporting** |  | 1 | 1 |
| **Description** |  |  |  |
| **Other bias** |  | 1 | 1 |
| **Description** |  |  |  |
| **A priori protocol** |  |  | No |
| **A priori analysis plan** |  |  | No |
| **Confounding** |  | 5 | 5 |
| **Description** |  | Age, gender, ethnicity, education considered but no imbalances shown or discussed or controlled for | No imbalances shown or discussed (but all (except mental health history) controlled for) and 16 bad controls (potential outcomes) included |
| **Relevant confounders described** |  | Age, gender, ethnicity, education considered but no imbalances shown or discussed or controlled for | All except mental health history controlled for and in addition several post-deployment covariates (a large number of potential outcomes) |
| **Method used for controlling for confounding (At design state)** |  | None | None |
| **Method used for controlling for confounding (At analysis stage)** |  | Linear regression, logistic regression | Logistic regression |

| **Author** | **Hourani, Williams , Bray , Wilk ,Hoge** | **Jacobson; Donoho; Crum-Cianflone** | **Lee, Garber** |
| --- | --- | --- | --- |
| **Year** | 2016 | 2015 | 2015 |
| **Sequence generation** | High | High | High |
| **Allocation concealment** | High | High | High |
| **Blinding** | 4 | 4 | 4 |
| **Incomplete outcome data** | 3 | 4 | 1 |
| **Description** | The overall response rate was 51.8% in 2005 and 70.6% in 2008. The response rates were high relative to most military surveys, and most items showed less than a 5% missing rate. The current study included only respondents from the Army to ensure a more homogeneous population that had the opportunity for combat duty in Iraq or Afghanistan, for a study total of 3,639 in 2005 (2,818 men; 821 women) and 5,927 in 2008 (4,320 men; 1,607 women). | Of the 55 019 participants who completed 2 follow-up surveys, 21 647 completed at least 1 deployment, and 20 255 screened negative for PTSD prior to outcome assessment, thereby making them eligible for the present study. Of those eligible, 16 754 (83%) had complete demographic, military, and behavioral data required for inclusion in the matching algorithm. Eighteen percent (2933) of this group was women. Propensity scores were used to match a man with each woman, if a match was obtainable. Eighty percent of the women were able to be matched with men, yielding a final study population of 4684 (2342 men and women). | Participants included 3319 members of the CAF Regular Force who had completed both the CAF RHQ and an Enhanced Post deployment Screening (EPDS) from 2009 to 2012 and for whom these records could be matched; numbers used for analysis is 3025 |
| **Selective reporting** | 1 | 1 | 1 |
| **Description** |  |  |  |
| **Other bias** | 1 | 1 | 1 |
| **Description** |  |  |  |
| **A priori protocol** | Unclear | Unclear | Yes |
| **A priori analysis plan** | Unclear | Unclear | Unclear |
| **Confounding** | 5 |  | 5 |
| **Description** | No imbalances shown or discussed (but all controlled for) and 7 bad controls (potential outcomes) included. In particular model is estimated conditional on other mental health outcomes post-deployment, such as depression, and alcohol abuse. |  | All considered but no imbalances shown or discussed or controlled for (in the analyses of PTSD and depression, only PCS which is not relevant for this review) |
| **Relevant confounders described** | All controlled for and in addition several post-deployment covariates (potential outcomes) | Match using propensity scores of being female and include post sexual harrassment (bad control) | All considered |
| **Method used for controlling for confounding (At design state)** | None | State they use confounders important for PTSD and gender. However the purpose of matching is not the treatment (deployed or experienced combat) we are interested in, it is the predicted probability of the dependent variable, which for the purpose of the study is being female ("the predicted probability of the dependent variable, in this case, female gender", p.32 ) | None |
| **Method used for controlling for confounding (At analysis stage)** | Logistic regression | Match on: Demographic and military data obtained from military electronic personnel files included gender, birth year, race/ethnicity, education level, marital status, service component, service branch, military pay grade, deployment experience in support of the operations in Iraq and Afghanistan, and military occupation where a combat specialist refers to individuals in occupations such as infantry, combat engineering, installation security, or aircrew. A range of Behavioral and mental health variables were obtained from responses to the baseline Millennium Cohort questionnaire and matched. Additional adjustment for sexual assault reported on both follow-up surveys. | Negative binomial regression analyses with continuous post concussive symptom scores as the dependent variable (headache, dizziness, feeling tired, trouble concentrating, forgetfulness, sleep problems, and irritability). Concerning PTSD and depression only correlation with CE is shown |

| **Author** | **Ikin, McKenzie, Gwini, Kelsall, Creamer et al.** | **Kanesarajah, Waller, Zheng, Dobson** | **Telch, Beevers, Rosenfield, Lee, Reijntjes, Ferrell** |
| --- | --- | --- | --- |
| **Year** | 2016 | 2016 | 2015 |
| **Sequence generation** | High | High | High |
| **Allocation concealment** | High | High | High |
| **Blinding** | 4 | 4 | 4 |
| **Incomplete outcome data** | 4 | 3 | 2 |
| **Description** | 1871 baseline deployed, non-deployed baseline 2924. FU 715 deployed (missing/attrition rate 62%) and 675 non-deployed (missing/attrition rate 77%). Investigated for "participation" bias (non-response), no major imbalances found | Totally, 26 239 eligible ADF members were invited to participate and 14 032 (53%) responded to the survey and 11 555 provided responses (missing data rate 18%). Analysis weighted for non-response to be representative of the population (assuming MAR) | 133 out of 184 included in analysis |
| **Selective reporting** | 1 | 1 | 1 |
| **Description** |  |  |  |
| **Other bias** | 1 | 1 | 1 |
| **Description** |  |  |  |
| **A priori protocol** | Unclear | No | Unclear |
| **A priori analysis plan** | Unclear | No | Unclear |
| **Confounding** | 5 | 5 | 5 |
| **Description** | Initial sample matched but attrition rates are high, small age, rank and branch imbalances, several confounders not considered and they do not report what they adjust for in the analysis (report 'raw' percentages as well) | Do not consider mental health history, ethnicity, and number of previously deployment. No imbalances shown or discussed but age, gender, rank, branch and duty/enlistment status controlled for (+ more of which 2: whether assigned work on deployment met their ability, their colleagues did what was expected of them, are potentially endogenous, and are likely to be correlated with the outcome of interest). | No imbalances shown or discussed, of pre specified only gender is controlled for. Army only. |
| **Relevant confounders described** | Only males deployed slightly older, small imbalance on rank and branch. Previous deployment, mental health history (pre deployment) ethnicity and duty/enlistment status not considered | Adjust for gender, age group, service, service status, and rank, whether assigned work on deployment met their ability, their colleagues did what was expected of them and whether they had any problems at home during most recent deployment. Do not consider mental health history, ethnicity, and number of previously deployment. No imbalances shown or discussed | Include CE level and change and control for gender and time since deployment and 5-HTTLPR genotype (serotonin transporter linked polymorphic region genotype) and include interaction terms (CE (both level and change) and 5-HTTLPR genotype) |
| **Method used for controlling for confounding (At design state)** | Matched sampling | None | None |
| **Method used for controlling for confounding (At analysis stage)** | Adjusted relative risk, do not report what they adjust for | Logistic regression | Regression |

| **Author** | **Mustillo, Kysar-Moon, Douglas, Hargraves, MacDermid et al.** | **Nyaronga, Toma** | **Ogle, Young** |
| --- | --- | --- | --- |
| **Year** | 2015 | 2015 | 2016 |
| **Sequence generation** | High | High | High |
| **Allocation concealment** | High | High | High |
| **Blinding** | 4 | 4 | 4 |
| **Incomplete outcome data** | 3 | 5 | 1 |
| **Description** | 20% missing data | Survey respond rate 20% (177 of 900) and of these 19% have all data missing (so end up with 144). Use EM algorithm to impute missing data from what we understand is the 144, but do not describe how many of the 144 had complete data. | Nothing reported but probably no missing data |
| **Selective reporting** | 1 | 1 | 1 |
| **Description** |  |  |  |
| **Other bias** | 1 | 1 | 1 |
| **Description** |  |  |  |
| **A priori protocol** | Unclear | Unclear | Unclear |
| **A priori analysis plan** | Unclear | Unclear | Unclear |
| **Confounding** | 5 | 5 |  |
| **Description** | Do not show or discuss imbalances, do not consider mental health history, number of deployments, rank, age, duty/enlistment status | No imbalances shown or discussed. Control for age, gender, ethnicity, branch and in addition include 4 bad controls (Divorce, family support, friend support, religiosity). Do not consider rank, duty/enlistment status, mental health history, number of deployments. | Males only. Otherwise nothing considered. Correlation analysis (CE vs depression) |
| **Relevant confounders described** | Include 5 bad controls (physical and mental health outcomes). Control for branch, ethnicity, marital status, gender, time deployed and deployment location (Iraq or Afghanistan). Do not consider mental health history, number of deployments, rank, age, duty/enlistment status | Deployed to combat zone but do not report whether the controls are non-deployed or deployed to non-combat zones, thus the study cannot be categorised as either reporting an absolute effect or a relative effect. |  |
| **Method used for controlling for confounding (At design state)** | None | None |  |
| **Method used for controlling for confounding (At analysis stage)** | Logistic regression | Regression |  |

| **Author** | **Quartana, Wilk ,Balkin , Hoge** | **Trautmann, Goodwin, Höfler, Jacobi, Strehle et al.** | **Zheng, Kanesarajah,Waller, McGuire, Treloar** |
| --- | --- | --- | --- |
| **Year** | 2015 | 2017 | 2016 |
| **Sequence generation** | High | High | High |
| **Allocation concealment** | High | High | High |
| **Blinding** | 4 | 4 | 4 |
| **Incomplete outcome data** | 3 | 2 | 2 |
| **Description** | 587 U.S. soldiers. Response rate 62% | Deployed soldiers response rate 92.8% and non-deployed response rate 95.4% | Response rate were 49% and 46% for Bougainville and East Timor. Analysis of non-response in table 2, p5. (Waller et al., 2012) appear to be relatively minor differences. |
| **Selective reporting** | 1 | 1 | 1 |
| **Description** |  |  |  |
| **Other bias** | 1 | 1 | 4 |
| **Description** |  |  | ADF faced deployments to Afghanistan (since 2001) and Iraq (since 2003). Models are not corrected for deployments to Middle East. (Measurement bias). Measurements taken 8 years after deployment (recall bias) |
| **A priori protocol** | Unclear | Unclear | Unclear |
| **A priori analysis plan** | Unclear | Unclear | Unclear |
| **Confounding** | 5 | 5 | 4 |
| **Description** | Consider age, gender, rank, ethnicity, education but do not show or discuss imbalances and do not control for them (as they are either not significant or imply any difference in a model of physical symptoms with combat exposure included as a continuous measure). Do not consider mental health history and number of deployments. All are Army active duty soldiers | Not all considered, some imbalance on those considered and include bad controls | Binary logistic regression models include both childhood adversity and traumatic exposure score variables and are adjusted for age group (≤34, 35-44, 45-54, ≥55), sex, service (Navy, Army and Air Force), service status (active regular, reserve, ex-serving) and rank (officer and enlisted). |
| **Relevant confounders described** | Age, gender, rank, ethnicity, education and whether a soldier reported being wounded or injured during combat are considered but not controlled for in reported results (do not include them because no significant partial effects of ethnicity, educational attainment and physical injury on PHQ-15 scores (Physical symptoms) and findings from a gender- and age-adjusted model did not deviate from the unadjusted model on the PHQ-15 scores). Note the non-significant and 'did not deviate' results are obtained in a model where combat exposure is included as a continuous measure as opposed to for example high vs. low (which could make a difference) | Only males included. Consider age, rank and unit, some imbalances. Also consider marital status and education at post deployment so bad controls. Presents only weighted per cents with PTSD, depression and alcohol abuse. | Age, gender, branch, duty/enlistment status and rank adjusted for. No imbalances shown or discussed. Show results for three levels of childhood trauma exposures (could serve as proxy for mental health history). Do not consider ethnicity and number of deployments. |
| **Method used for controlling for confounding (At design state)** | None | Deployed to Afghanistan in 2010. The random sample was stratified, oversampling combat personnel as an assumed high-risk population. Never deployed soldiers were drawn from the same home base locations | None |
| **Method used for controlling for confounding (At analysis stage)** | Regression | Nothing | Logistic regression but with CE as a continuous variable and interaction terms |
