# Supplementary material for: Deployment of personnel to military operations: impact on mental health and social functioning
Source: Campbell Syst Rev. 2018 Jun 1;14(1):1–127. doi: 10.4073/csr.2018.6 (PMC8427986; doi:10.4073/csr.2018.6)
Supplement: Supplementary file 2 — Supplementary material [file CL2-14--s004.docx]

# Online Supplement 2: Characteristics of Included Studies

This supplement contains charateristics of studies table for studies that are included in the review. The tables are organized in two sections. The first section (section 1.1) contains those studies that are included in synthesis (analysis sample, both main and sensitivity). Section 1.2 contains the studies that are not part of the analysis sample. A study may not be included in synthesis for the following reasons: a) we were unable to extract an effect size from the study, b) the study had sample overlap with another study, and that study was rated with a smaller risk of bias.

Abbreviations used:

- Participants/Sample size: T = Treated, C = Controls, N = total sample size
- Contrast/Comparison: A = Absolute contrast (deployed v n0t deployed), DE = Deployed Elsewhere (e.g. deployed to combat zone v deployed to German base), CE = Combat exposure (dose-response).
- Effect size extract/Type of ES: SMD = Standardised Mean Difference, OR = Odds ratio, RR = Risk ratio

## Characteristics of Studies in Analysis Sample main and sensitivity

| **Study info** |  |  |  |
| --- | --- | --- | --- |
| Author(s) | Al-Turkait, F. A. & Ohaeri, J. U. | Baggaley, M. R., Piper, M. E., Cumming, P., & Murphy, G. | Black, D. W., Carney, C. P., Peloso, P. M., Woolson, R. F., Schwartz, D. A., Voelker, M. D. et al. |
| Year | 2008 | 1999 | 2004 |
| **Participants** |  |  |  |
| Sample | 0 |  | The Iowa Gulf War Study |
| Samplesize | (1) retired group: 50; (2) active duty - rear: 50; (3) active duty - battle: 50; (4) PoWs: 50 | T: 145, C: 237 | Regular Military: T: 985, C: 968. Guard/Reserve: T: 911, C: 831 |
| Branch | NA | NA | MA |
| Rank (% Enlisted) | NA | NA | NA |
| Active Duty Status (%) | NA | NA | NA |
| **Intervention** |  |  |  |
| Deployed from | Kuwait | UK | USA |
| Deployed to | Gulf war | Bosnia | Gulf War |
| Type of deployment | War zone | Civil conflict | War zone |
| **Contrast** |  |  |  |
| Military Comparison | Yes | Yes | Yes |
| Comparison | CE | A | A |
| **Outcome** |  |  |  |
| Outcome | PTSD (CAPS), Depression | PTSD (Impact of Event Scale (IES)), CMD (GHQ-28) | PTSD (Check-list) |
| Outcome type | Dichotomous | Dichotomous | Dichotomous |
| Time point | Deployment: First Gulf War. Data collected: July 1997 - December 1998 | Deployment: Nov. 1992 - April 1993. Data collected: 36 months after | Deployment: Aug. 2, 1990 – July 31, 1991. Data collected: September 1995 – May 1996 |
| Source | Clinician interview, Hopkins | Questionnaire | Questionnaire |
| **Method** |  |  |  |
| Estimation | test | test | Logistic regression + SUDAAN (weights) |
| **Effect size extraction** |  |  |  |
| ES extracted | 2 | 2 | 2 |
| Type of ES | OR, SMD | OR | OR |
| Notes | Score 5 in confounding | Score 5 in confounding | 0 |

| **Study info** |  |  |  |
| --- | --- | --- | --- |
| Author(s) | Booth-Kewley S;Schmied EA;Highfill-McRoy RM;Larson GE;Garland CF;Ziajko LA; | Booth-Kewley, S., Larson, G. E., Highfill-McRoy, R. M., Garland, C. F., & Gaskin, T. A. | Breen-Lopez CJ; |
| Year | 2013 | 2010 | 2014 |
| **Participants** |  |  |  |
| Sample | Warfighter Status Survey |  | VISN 6 MIRECC. |
| Samplesize | N=1113 | NA | N = 1669 |
| Branch | Marines | Marines | OIF, OEF or OND |
| Rank (% Enlisted) | 90.7 | 90.1 | NA |
| Active Duty Status (%) | 1 | 83.6 | NA |
| **Intervention** |  |  |  |
| Deployed from | USA | USA | USA |
| Deployed to | OIF;OEF | OIF, OEF | OIF;OEF |
| Type of deployment | War zone | War zone | War zone |
| **Contrast** |  |  |  |
| Military Comparison | Yes | Yes | Yes |
| Comparison | CE (H, M vs L) | CE (vH/H/M vs L) | CE (continuous) |
| **Outcome** |  |  |  |
| Outcome | CDM; PTSD | PTSD (PCL-C) | PTSD |
| Outcome type | Dichotomous | Dichotomous | Dichotomous (continuous available) |
| Time point | New onset of psychiatric diagnosis between survey completion (June 2007 - January 2008) and December 2010 (p4) and OIF/OEF deployed | Deployment: 2002 - 2007 Data collected: June 2007 - Jan 2008 | Not stated. All deployed after 2001 and surveyed upon return |
| Source | CHAMPS | Questionnaire | Questionnaire: (SCID) |
| **Method** |  |  |  |
| Estimation | logistic regression | logistic regression | Hierarchical logistic regression |
| **Effect size extraction** |  |  |  |
| ES extracted | 4 | 3 | 1 |
| Type of ES | OR | OR | OR |
| Notes |  | OR by combat intensity | 0 |

| **Study info** |  |  |  |
| --- | --- | --- | --- |
| Author(s) | Cerdá, M; C. Richards; G. H. Cohen, J. R. Calabrese; I. Liberzon; M. Tamburrino; S. Galea; K. C. Koenen. | Chapman PL;Elnitsky C;Thurman RM;Pitts B;Figley C;Unwin B; | Coughlin SS;Kang HK;Mahan CM; |
| Year | 2014 | 2014 | 2011 |
| **Participants** |  |  |  |
| Sample | Sample of Ohio National Guard members | Original sample is a 3-year longitidunal study of US combat medics | National Health Survey of Gulf War Era Veterans and Their Families. |
| Samplesize | N=1095 | T: 196, C: 256 | T: 6,111 C: 3,859 |
| Branch | Army | Army | Air Force, Army, Marine Corps, Navy |
| Rank (% Enlisted) | NA | 1 | 84.3 |
| Active Duty Status (%) | 0 | NA | 35.7 |
| **Intervention** |  |  |  |
| Deployed from | NA | USA | USA |
| Deployed to | OIF;OEF | NA | Gulf war |
| Type of deployment | NA | NA | War zone |
| **Contrast** |  |  |  |
| Military Comparison | Yes | Yes | Yes |
| Comparison | CE (H vs L and M vs L) | A | A |
| **Outcome** |  |  |  |
| Outcome | Alcohol use disorder | PTSD and depression | PTSD, Depression |
| Outcome type | Dichotomous | Dichotomous | Dichotomous |
| Time point | Deployed: June 2008–Feb 2009. Data collected: 0-4 years after deployment | 12 post deployment | Gulf War (1991) survey in 2005 |
| Source | Alcohol use (MINI), (DSM-IV) | Questionnaire: PTSD( PCL), depression (PHQ-9) | Questionnaire: PTSD (PCL-C), (PHQ-9). |
| **Method** |  |  |  |
| Estimation | A generalized linear mixed model with a random intercept was used to estimate SEs | Sequential logistic regression (p. 21: after partialing out significant demographic characteristics… And depression (PTSD) screening outcomes) Not further explained | Logistic regression |
| **Effect size extraction** |  |  |  |
| ES extracted | 2 | 2 | 2 |
| Type of ES | OR | OR | OR |
| Notes | Relative exposure | Score 5 in confounding | 0 |

| **Study info** |  |  |  |
| --- | --- | --- | --- |
| Author(s) | David, A. S., Farrin, L., Hull, L., Unwin, C., Wessely, S., & Wykes, T. | Dedert, E. A., Green, K. T., Calhoun, P. S., Yoash-Gantz, R., Taber, K. H., Mumford, M. M. et al. | Farmer, Vaughan, Garnet & Weinick |
| Year | 2002 | 2009 | 2014 |
| **Participants** |  |  |  |
| Sample | The British Gulf War Study |  | 0 |
| Samplesize | C: 78; Gulf: 207; Bosnia: 54 | N=356 | T: 1062, C: 1463 |
| Branch | Army, Navy, Air Force, Marines | NA | U.S. Marines |
| Rank (% Enlisted) | 88 | NA | 86.2 |
| Active Duty Status (%) | NA | 3 | NA |
| **Intervention** |  |  |  |
| Deployed from | UK | USA | USA |
| Deployed to | Gulf War; Bosnia | NA | OIF;OEF |
| Type of deployment | War zone, Civil conflict | NA | War zone |
| **Contrast** |  |  |  |
| Military Comparison | Yes | Yes | Yes |
| Comparison | A; DE | CE | A |
| **Outcome** |  |  |  |
| Outcome | Depression (BDI), PTSD (Mississippi) | PTSD and depression (DSM-IV, SCID), Substance abuse (AUDIT) | PTSD, depression and Alcohol |
| Outcome type | Dichotomous (from continuous scale) | Dichotomous | PTSD: continuous, depression and Alcohol: dichotomous |
| Time point | Deployment: T: Sept. 1, 1990 – June 30, 1991. C: Bosnia: April 1, 1992 – Feb. 6, 1997. Era Cohort not reported | Deployment: Serving since 2001. Data collected: NA | Deployed: 2001-2011. Data collected: 0-10 years after deployment |
| Source | Questionnaire | Questionnaire | Questionnaire: PTSD (PCL-C), Depression (PHQ-2), Alcohol (AUDIT-C) |
| **Method** |  |  |  |
| Estimation | ANCOVA, test for main effects only reported | Logistic regression |  |
| **Effect size extraction** |  |  | Weighted population sample |
| ES extracted | 2 | 3 | 3 |
| Type of ES | OR | OR | SMD, OR |
| Notes | Overlapping samples. Only depression extracted. Score 5 in confounding | Score 5 in confounding | All converted to OR. Score 5 in confounding |

| **Study info** |  |  |  |
| --- | --- | --- | --- |
| Author(s) | Foster, E. M. | Gehrman, Seelig, Jacobson, Boyko, Hooper, Gackstetter, Ulmer & Smith | Gordon, J. G. A. |
| Year | 2011 | 2013 | 2002 |
| **Participants** |  |  |  |
| Sample | Department of Defense survey of health related behaviors among the Guard and Reserve Force | 0 |  |
| Samplesize | C: Women: 2632, Men: 7447; T: Women: 812, Men: 5023 | N1 = 8902 and N2= 6098 (two groups analysed separately). | T: 1,168, C: 669 |
| Branch | Army, Navy, Air Force, Marines | Army, Air Force, Navy /Coast Guard, Marine | NA |
| Rank (% Enlisted) | 85.3 | 71.8 | NA |
| Active Duty Status (%) | None | 55.9 | NA |
| **Intervention** |  |  |  |
| Deployed from | USA | USA | Canada |
| Deployed to | OIF, OEF | OIF;OEF | Gulf War, Bosnia, Croatia, Golan Heights, Haiti, Somalia |
| Type of deployment | NA | War zone | NA |
| **Contrast** |  |  |  |
| Military Comparison | Yes | Yes | Yes |
| Comparison | A | CE (Y/N) | A |
| **Outcome** |  |  |  |
| Outcome | PTSD , Alcohol and drug abuse, Depression | PTSD, depression | PTSD (PCL-M), Depression (CES-D) and Alcohol abuse |
| Outcome type | Dichotomous | Dichotomous | Continuous |
| Time point | Deployment: 2004-2006. Data collected: 2006 | Survey cycles are 2004-2006, and 2007-2008. G1 time points: 0 - 5 years, G2: 0 - 4 years | Data collected: Sept. 1999 – Dec. 1999 |
| Source | Questionnaire | Questionnaire: PTSD (PCL-C), Depression | Questionnaire |
| **Method** |  |  |  |
| Estimation | Propensity score weighting. Inverse probability of treatment (IPTW)-weighted logistic regression |  | None |
| **Effect size extraction** |  | Logistic regression |  |
| ES extracted | 8 | 4 | 3 |
| Type of ES | OR | OR | SMD |
| Notes | Multinomial logit. Males and females estimated separately. | Anxiety also available | Converted to OR. Score 5 in confounding |

| **Study info** |  |  |  |
| --- | --- | --- | --- |
| Author(s) | Gray GC;Reed RJ;Kaiser KS;Smith TC;Gastanaga VM; | Harvey SB;Hatch SL;Jones M;Hull L;Jones N;Greenberg N;Dandeker C;Fear NT;Wessely S; | Hoge, C. W., Auchterlonie, J. L., & Milliken, C. S. |
| Year | 2002 | 2012 | 2006 |
| **Participants** |  |  |  |
| Sample | Gulf War Seabees | The British Iraq War Study | Army and Marine OIF OEF |
| Samplesize | T (Gulf War deployed): 3831, C (deployed elsewhere): 4933, C (not deployed): 3104 | T: 552; C: 391 | OIF: 222,620; OEF: 16,318, Other: 64,967 |
| Branch | Navy (seabees) | Navy, Army; Air Force | Army, Marines |
| Rank (% Enlisted) | 90.8 | 80.5 | 88.1 |
| Active Duty Status (%) | 77.6 | 0 | 62.1 |
| **Intervention** |  |  |  |
| Deployed from | USA | UK | USA |
| Deployed to | Gulf war | OIF | OIF, OEF |
| Type of deployment | War zone | War zone | War zone |
| **Contrast** |  |  |  |
| Military Comparison | Yes | Yes | Yes |
| Comparison | A, CE | A | CE |
| **Outcome** |  |  |  |
| Outcome | New onset PTSD and Depression | CDM; Probable PTSD; Alcohol misuse | PTSD (Primary Care PTSD Screen), Depression (PHQ-2) |
| Outcome type | Dichotomous | Dichotomous (3) | Dichotomous |
| Time point | Deployed: 1991. Data collected: 8 years after (1999) | Data collected: 16 months after (Time 1) & 4.8 years after deployment (Time2) | Data collected: May 1, 2003 - April 30, 2004 |
| Source | Questionnaire, self-reported physician-diagnosed | CMD (GHQ-12), PTSD: PCL-C, Alcohol (AUDIT) | Questionnaire |
| **Method** |  |  |  |
| Estimation | Logistic regression | Logistic regression (with weights) | logistic regression (but only reported for outcome (1)) |
| **Effect size extraction** |  |  |  |
| ES extracted | 4 | 3 | 2 |
| Type of ES | OR | OR | OR |
| Notes | Alcohol (RoB 5). Only ask if they drink alcohol and not how much | 0 | Depression not reported. |

| **Study info** |  |  |  |
| --- | --- | --- | --- |
| Author(s) | Holmes, D. T., Tariot, P. N., & Cox, C. | Hotopf, M., David, A., Hull, L., Ismail, K., Unwin, C., & Wessely, S. | Hotopf, M., Hull, L., Fear, N. T., Browne, T., Horn, O., Iversen, A. et al. |
| Year | 1998 | 2003 | 2006 |
| **Participants** |  |  |  |
| Sample |  | British Gulf War Study | The British Iraq War Study |
| Samplesize | T: 296, C: 179 | T (Gulf-Bosnia): 570, T: (Bosnia-only): 2049 C (Era): 1785 | T: 5,481, C: 4,613 |
| Branch | Air Force | regular army, | Army, Navy, Air Force, Marines |
| Rank (% Enlisted) | 90 | 86.3 | 84 |
| Active Duty Status (%) | None | 1 | 91 |
| **Intervention** |  |  |  |
| Deployed from | USA | UK |  |
| Deployed to | Gulf War | Bosnia | OIF |
| Type of deployment | War zone | Civil conflict | Combat operation |
| **Contrast** |  |  |  |
| Military Comparison | Yes | Yes | Yes |
| Comparison | A | A | A, CE |
| **Outcome** |  |  |  |
| Outcome | PTSD (Mississippi), CMD (SCL-90-R) | GHQ-12, PTSD, alcohol () | PTSD (PCL-C); CMD (GHQ-12); Alcohol (WHO AUDIT) |
| Outcome type | Dichotomous | Dichotomous | Dichotomous |
| Time point | 11 months after return / cessation of hostilities | Deployed: 1992-1996. Data collected: 1997 - 1998, 6-7 years after return and 1-6 years after return (Bosnia) | Deployment: Jan. 18, 2003 - June 28, 2003. Data collection: June, 2004 – Mar. 2, 2006 |
| Source | Questionnaire | Questionnaire: PTSD measure is homemade, but based on the Mississippi scale, Alcohol (GHQ-12), | Questionnaire |
| **Method** |  |  |  |
| Estimation | None | Logistic regression | Oversampling of some strata. Weights used. Controlled for confounders with logistic regression |
| **Effect size extraction** |  |  |  |
| ES extracted | 2 | 2 | 6 |
| Type of ES | OR | OR | OR |
| Notes | Depression not reported other than median score. Score 5 in confounding | 0 | 0 |

| **Study info** |  |  |  |
| --- | --- | --- | --- |
| Author(s) | Ikin, J. F., Sim, M. R., Creamer, M. C., Forbes, A. B., McKenzie, D. P., Kelsall, H. L. et al. | Ishoy, T., Suadicani, P., Guldager, B., Appleyard, M., Hein, H. O., & Gyntelberg, F. | Jones M;Rona RJ;Hooper R;Wesseley S; |
| Year | 2004 | 1999 | 2006 |
| **Participants** |  |  |  |
| Sample | Same sample as 928, 1104, 1122 and 1123 | The Danish Gulf War Study | 0 |
| Samplesize | T: 1349, C: 1342; | T: 686; C: 231 | N=1382 (full questionnaires), + 1491 (abrigded) |
| Branch | Army, Navy, Air Force | NA | NA |
| Rank (% Enlisted) | 81.1 | NA | NA |
| Active Duty Status (%) | NA | NA | NA |
| **Intervention** |  |  |  |
| Deployed from |  | Denmark | UK |
| Deployed to | Australia | Gulf War | NA |
| Type of deployment | Gulf War | NA | NA |
| **Contrast** | War zone |  |  |
| Military Comparison | Yes | Yes | Yes |
| Comparison | A; DE | A | A |
| **Outcome** |  |  |  |
| Outcome | Major Depression, PTSD, Alchol abuse, Drug abuse | Alcohol, Depression | PTSD and alcohol |
| Outcome type | Dichotomous | Continuous: Alcohol Dichotomous: Depression | Dichotomous |
| Time point | Deployment: Aug. 2, 1990 – Sept. 4, 1991. Data collected: August, 2000 - April, 2002 | Deployment: Aug. 2, 1990 – Dec. 31, 1997. Data collected: January 1997 - January 1998 | Not reported |
| Source | Clinical interview (CIDI) | Questionnaire | Questionnaire: PTSD (PCL), alcohol consumption |
| **Method** |  |  |  |
| Estimation | Logistic regressions | None | Logistic regression |
| **Effect size extraction** |  |  |  |
| ES extracted | 9 | 2 | 2 |
| Type of ES | OR | OR, SMD | OR |
| Notes | Two types, we use Any case 12 months prior to measurement. | SMD converted to OR | Score 5 in confounding |

| **Study info** |  |  |  |
| --- | --- | --- | --- |
| Author(s) | Jones N;Thandi G;Fear NT;Wessely S;Greenberg N; | Kelley, M. L., Hock, E., Jarvis, M. S., Smith, K. M., Gaffney, M. A., & Bonney, J. F. | Killgore, W. D. S., Stetz, M. C., Castro, C. A., & Hoge, C. W. |
| Year | 2014 | 2002 | 2006 |
| **Participants** |  |  |  |
| Sample | Sample of UK afghanistan veterans |  |  |
| Samplesize | T: 1061, C: 1698 | T: 48, C: 72 | T: 173, C: 1895 |
| Branch | NA | Navy | Army |
| Rank (% Enlisted) | NA | All | 86.1, |
| Active Duty Status (%) | NA | NA | All |
| **Intervention** |  |  |  |
| Deployed from | UK | USA | USA |
| Deployed to | OEF | NA | OIF |
| Type of deployment | War zone | NA | War zone |
| **Contrast** |  |  |  |
| Military Comparison | Yes | Yes | Yes |
| Comparison | CE (exposure vs none) | A | A |
| **Outcome** |  |  |  |
| Outcome | PTSD and common mental disorder (CMD) | Depression (CES-D) | PTSD, Depression |
| Outcome type | Dichotomous | Continuous | Dichotomous |
| Time point | Towards the end of deployment | 3–6 weeks before and after | Unclear |
| Source | PTSD (PCL-C), CMD (GHQ-12) | Questionnaire | Questionnaire |
| **Method** |  |  |  |
| Estimation | Logistic regression | Multiple regression | None |
| **Effect size extraction** |  |  |  |
| ES extracted | 2 | 1 | 2 |
| Type of ES | OR | SMD | OR |
| Notes | 0 | Converted to OR. Score 5 in confounding | Score 5 in confounding |

| **Study info** |  |  |  |
| --- | --- | --- | --- |
| Author(s) | Kline, A., Falca-Dodson, M., Sussner, B., Ciccone, D. S., Chandler, H., Callahan, L. et al. | Lande, R. G., Marin, B. A., Chang, A. S., & Lande, G. R. | Luxton DD;Greenburg D;Ryan J;Niven A; Wheeler G; Mysliwiec V; |
| Year | 2010 | 2008 | 2011 |
| **Participants** |  |  |  |
| Sample | OIF and OEF deployed of New Jersey Army National Guard troops preparing for deployment to Iraq in 2008. | OIF deployed | Soldiers from a redeploying brigade combat team in Iraq. |
| Samplesize | T: 625, C: 1910 | T: 326, C: 640 | Total 2717 |
| Branch | Army | NA | Army |
| Rank (% Enlisted) | NA | NA |  |
| Active Duty Status (%) | None | None | 93 |
| **Intervention** |  |  | NA |
| Deployed from | USA | USA | USA |
| Deployed to | OIF, OEF | NA | OIF |
| Type of deployment | War zone | NA | War zone |
| **Contrast** |  |  |  |
| Military Comparison | Yes | Yes | Yes |
| Comparison | A | A | CE |
| **Outcome** |  |  |  |
| Outcome | PTSD, Depression, Alcohol and substance abuse (all DSM-IV) | Alcohol abuse | PTSD, depression and alcohol abuse |
| Outcome type | Dichotomous | Dichotomous | Dichotomous |
| Time point | Deployment: 2001 – 2007. Data collected: November 2007 – May 2008 | NA | Data collected: 90 - 180 days upon return |
| Source | Questionnaire | Questionnaire | PTSD (PC-PTSD); Depression (PHQ-9); Alcohol (>5) |
| **Method** |  |  |  |
| Estimation | Logistic regression | None | Logistic regression |
| **Effect size extraction** |  |  |  |
| ES extracted | 6 | 1 | 12 |
| Type of ES | OR | OR | OR |
| Notes | Additionally: PTSD (1), Depression (1) available. 3 Alcohol ES coded | Unadjusted OR (5 in confounding) | Score 5 in confounding |

| **Study info** |  |  |  |
| --- | --- | --- | --- |
| Author(s) | Macera CA;Aralis HJ;Highfill-McRoy R;Rauh MJ; | McCarroll JE;Ursano RJ;Fullerton CS; | Perconte ST;Wilson AT;Pontius EB;Dietrick AL;Spiro KJ; |
| Year | 2014 | 1993 | 1993 |
| **Participants** |  |  |  |
| Sample | PDHA and PDHRA records | Sample deployed to ODS | NA |
| Samplesize | Iraq (OIF): 24.375, Afghanistan (OEF): 3.115, Kuwait: 4.044 | T: 116; C: 118 | T: 439, C: 126 |
| Branch | Marine Corps, Navy | NA | Marine, Navy, Army |
| Rank (% Enlisted) | 89.1 | NA | NA |
| Active Duty Status (%) | 94.8 | NA | 0 |
| **Intervention** |  |  |  |
| Deployed from | USA | USA | USA |
| Deployed to | OIF;OEF | Gulf War | Gulf War |
| Type of deployment | War zone | War zone | War zone |
| **Contrast** |  |  |  |
| Military Comparison | Yes | Yes | Yes |
| Comparison | CE | CE | A |
| **Outcome** |  |  |  |
| Outcome | PTSD | PTSD, CMD | PTSD, depression,CMD |
| Outcome type | Dichotomous | continuous | Continuous |
| Time point | 90-180 days after return | 3-5 months after exposure/return to US. | Data collected: 1993. 0 - 1 years post |
| Source | PTSD (PC-PTSD) | Impact Event Scale | PTSD: Mississippi, depression: (BDI), SCL-90R |
| **Method** |  |  |  |
| Estimation | Univariate logistic regression | t-test | None |
| **Effect size extraction** |  |  |  |
| ES extracted | 4 | 2 | 3 |
| Type of ES | OR | OR | SMD |
| Notes | 2 different exposure items, by gender. Score 5 in confounding | Score 5 in confounding | Converted to OR. Score 5 in confounding |

| **Study info** |  |  |  |
| --- | --- | --- | --- |
| Author(s) | Peterson, A. L., Wong, V., Haynes, M. F., Bush, A. C., & Schillerstrom, J. E. | Pierce, P. F. | Polusny, M. A., Erbes, C. R., Arbisi, P. A., Thuras, P., Kehle, S. M., Rath, M. et al. |
| Year | 2010 | 2005 | 2009 |
| **Participants** |  |  |  |
| Sample | Active-duty U.S. Air Force Members | Women deployed to the Persian Gulf. | Cohort of National Guards |
| Samplesize | T: 4408, C: 959 | NA | T: 29, C: 493 |
| Branch | Air Force | Air Force, (p. 350) | National Guard soldiers |
| Rank (% Enlisted) | 82.6 | 68.1 | 90.2 |
| Active Duty Status (%) | All | 26 | None |
| **Intervention** |  |  |  |
| Deployed from | USA | USA | USA |
| Deployed to | OIF | Gulf War | OIF |
| Type of deployment | War zone | War zone | War zone, Combat zone |
| **Contrast** |  |  |  |
| Military Comparison | Yes | Yes | Yes |
| Comparison | DE | DE | A |
| **Outcome** |  |  |  |
| Outcome | PTSD (PC-PTSD), Depression (PHQ-2) | Depression | PTSD (PCL), Depression (BDI-II) |
| Outcome type | Dichotomous | Dichotomous | Continuous |
| Time point | Data collected: June 1, 2005 -December 31, 2007 | 6 years after return | Deployment: Not reported (2001 and onwards). Data collected: February 2006 |
| Source | Questionnaire | Questionnaire | Questionnaire |
| **Method** |  |  |  |
| Estimation | Odds ratio | None | None |
| **Effect size extraction** |  |  |  |
| ES extracted | 2 | 1 | 2 |
| Type of ES | OR | OR | SMD |
| Notes | 0 | 0 | Sample: Camp Shelby. Converted to OR. |

| **Study info** |  |  |  |
| --- | --- | --- | --- |
| Author(s) | Polusny, M. A., Erbes, C. R., Murdoch, M., Arbisi, P. A., Thuras, P., & Rath, M. B. | Proctor, S. P., Heaton, K. J., Dos Santos, K. D., Rosenman, E. S., & Heeren, T. | Riddle JR;Smith TC;Smith B;Corbeil TE;Engel CC;Wells TS;Hoge CW;Adkins J;Zamorski M;Blazer D; |
| Year | 2011 | 2009 | 2007 |
| **Participants** |  |  |  |
| Sample | Cohort of National Guards | Bosnia deployed | Millenium Cohort Study |
| Samplesize | N = 349 | T: 67, C: 52 | T: 23,182; C: 53,536 |
| Branch | Army | Army | Army, Air Force, Navy/Coast Guard, Marine. |
| Rank (% Enlisted) | 88.9 | 90.8 | 77.3 |
| Active Duty Status (%) | 0 | None | 57 |
| **Intervention** |  |  |  |
| Deployed from | USA | USA | USA |
| Deployed to | OIF | Bosnia | Gulf War;Bosnia;Kosovo |
| Type of deployment | War zone, Combat zone | Civil conflict | NA |
| **Contrast** |  |  |  |
| Military Comparison | Yes | Yes | Yes |
| Comparison | CE (CE vs not) | A | A |
| **Outcome** |  |  |  |
| Outcome | PTSD (PCL-M) | Depression (POMS) | PTSD, MDD, Alcohol abuse |
| Outcome type | Dichotomous | Continuous | Dichotomous (3) |
| Time point | Data collected: September 2007 | Data collected: March 2002 – April 2002 | Deployed: 1998- 2000. Data collected: 1- 5 years after |
| Source | Questionnaire | Questionnaire | PTSD (PCL-C), MDD + Alcohol (PHQ) |
| **Method** |  |  |  |
| Estimation | Hierarchical logistic regression | Generalized estimating equation (GEE) models with Time 2 mood as outcomes. Unit cohesion at Time 2 was also included in the GEE models | logistic regression |
| **Effect size extraction** |  |  |  |
| ES extracted | 1 | 1 | 1 |
| Type of ES | OR | SMD | OR |
| Notes | Sample: Camp Shelby. | Converted to OR. Score 5 in confounding | Sample overlap: Millenium Cohort Study. 5 in confounding, only alcohol used |

| **Study info** |  |  |  |
| --- | --- | --- | --- |
| Author(s) | Riviere LA;Kendall-Robbins A;McGurk D;Castro CA;Hoge CW; | Shen YC;Arkes J;Pilgrim J; | Shen, Y. C., Arkes, J., Kwan, B. W., Tan, L. Y., & Williams, T. V. |
| Year | 2011 | 2009 | 2009 |
| **Participants** |  |  |  |
| Sample | National Guards who deployed to Iraq. | The DD2796 Post-Deployment Health Assessment (PDHA) survey. US Navy |  |
| Samplesize | N=2539 (at 3mths), N=1459 (at 12mths) | N=112,720. Comparison is deployed to ship (74%) | C: 489,101; T1:53,527; T2:134,610 |
| Branch | NA | Navy | Army, Marines, Navy, Air Force |
| Rank (% Enlisted) | 93.4 | 1 | All |
| Active Duty Status (%) | 0 | 1 | All |
| **Intervention** |  |  |  |
| Deployed from | USA | USA |  |
| Deployed to | OIF | OIF;OEF | OIF, OEF |
| Type of deployment | War zone | War zone | Combat operation |
| **Contrast** |  |  |  |
| Military Comparison | Yes | Yes | Yes |
| Comparison | CE | DE | A |
| **Outcome** |  |  |  |
| Outcome | PTSD; Major Depression | PTSD | PTSD (diagnosed, TRICARE system) |
| Outcome type | Dichotomous (2) | Dichotomous | Dichotomous |
| Time point | Data collected: 2005 -2007, 3 and 12 months after deployment. | Deployed: 2002-2006 Data collected: 30 days after | 2001 - 2006 |
| Source | PTSD: PCL, Major Depression: PHQ-9 | Questionnaire. PTSD (PC-PTSD) | Administrative data |
| **Method** |  |  |  |
| Estimation | logistic regression | Probit model | logistic regression |
| **Effect size extraction** |  |  |  |
| ES extracted | 4 | 2 | 8 |
| Type of ES | OR | Probit estimate and control risk and sample size | OR |
| Notes | Score 5 in confounding | Calculated OR. | By service |

| **Study info** |  |  |  |
| --- | --- | --- | --- |
| Author(s) | Simmons, R., Maconochie, N., & Doyle, P. | Simms, L. J., Watson, D., & Doebbeling, B. N. | Skopp NA;Reger MA;Reger GM;Mishkind MC;Raskind M;Gahm GA; |
| Year | 2004 | 2002 | 2011 |
| **Participants** |  |  |  |
| Sample | Gulf War cohort | Iowa Gulf War Study Group | Iraq Deployers who didnt screen positive for PTSD predeployment |
| Samplesize | C: 17,730; T: 23,358 | T: 1896, C: 1799 | N = 2583 |
| Branch | Army, Navy, Air Force | NA | All army |
| Rank (% Enlisted) | 87 | NA | 91.2 |
| Active Duty Status (%) | NA | NA | 99.7 |
| **Intervention** |  |  |  |
| Deployed from | UK | USA | USA |
| Deployed to | Gulf War | Gulf War | OIF;OEF |
| Type of deployment | War zone | War zone | War zone |
| **Contrast** |  |  |  |
| Military Comparison | Yes | Yes | Yes |
| Comparison | A | A | CE (yes vs no) |
| **Outcome** |  |  |  |
| Outcome | Depression, PTSD | PTSD (PCL-M), Depression and alcohol abuse (PRIME-MD) | PTSD |
| Outcome type | Dichotomous | Continuous | Dichotomous |
| Time point | Deployment: Aug. 1990 - June 1991. Data collected: August 1998 - March 2001 | Data collected: 2006 | Predeployment : 45-120 days. Post : 90-180 days |
| Source | Questionnaire | Questionnaire | PTSD: Primary Care PTSD Screen |
| **Method** |  |  |  |
| Estimation | Logistic regresssion | None | logistic regression |
| **Effect size extraction** |  |  |  |
| ES extracted | 2 | 3 | 1 |
| Type of ES | OR | SMD | Marginal effect (OR) |
| Notes | 0 | Converted to OR. Depression, and Alcohol extracted. | Combat exposure range 0 - 4. Mean and SD reported |

| **Study info** |  |  |  |
| --- | --- | --- | --- |
| Author(s) | Skotnicka J; | Smith TC;Ryan MA;Wingard DL;Slymen DJ;Sallis JF;Kritz-Silverstein D;Millennium Cohort Study Team; | Street AE;Gradus JL;Giasson HL;Vogt D;Resick PA; |
| Year | 2013 | 2008 F | 2013 |
| **Participants** |  |  |  |
| Sample | Survey on Soldiers returned from Iraq. | Millennium Cohort Study. | DoD Manpower Data Center’s roster of OEF/OIF Veterans |
| Samplesize | T: 30, C: 30 | N = 48299 | N = 2344 |
| Branch | NA | Army, Air Force, Navy, Marine Corps | Marines, Army, Navy, Air Force, Coast Guard |
| Rank (% Enlisted) | NA | 73.6 | NA |
| Active Duty Status (%) | NA | 62 | 48.9 |
| **Intervention** |  |  |  |
| Deployed from | Poland | USA | USA |
| Deployed to | OIF | OIF;OEF | OIF;OEF |
| Type of deployment | NA | War zone | War zone |
| **Contrast** |  |  |  |
| Military Comparison | Yes | Yes | Yes |
| Comparison | A | A | CE (1 pt change in scale and 5 points change) |
| **Outcome** |  |  |  |
| Outcome | PTSD and depression | PTSD | PTSD |
| Outcome type | Continuous | Dichotomous | Dichotomous |
| Time point | 1 year after deployment | Deployed between 2001-2009. Uses Panel 1 and 2 follow-up (2004 and 2007 for panel 1, enrolled 2001-2003. 2007 for panel 2, enrolled 2004-2006). | Not reported but data from 2009 and OIF/OEF deployment |
| Source | PTSD questionnaire, Depression (BDI), Anxiety (STAI) | Questionnaire: (PCL-C) | PTSD (PCL-M) |
| **Method** |  |  |  |
| Estimation | None | Logistic regression | Logistic regression with weights to adjust for non-response |
| **Effect size extraction** |  |  |  |
| ES extracted | 1 | 8 | 2 |
| Type of ES | SMD | OR | OR |
| Notes | Converted to OR. Score 5 in confounding | By combat/non-combat and branch | Effect of 1 point change in CES |

| **Study info** |  |  |  |
| --- | --- | --- | --- |
| Author(s) | Stuart JA;Halverson RR; | Sundin J;Herrell RK;Hoge CW;Fear NT;Adler AB;Greenberg N;Riviere LA;Thomas JL;Wessely S;Bliese PD; | Sutker PB;Davis JM;Uddo M;Ditta SR; |
| Year | 1997 Nov | 2014 | 1995 |
| **Participants** |  |  |  |
| Sample | US Army Soldiers that deployed to Gulf, Somalia, Kuwait, Haiti and Bosnia | US and UK soldiers |  |
| Samplesize | C: 1339; T: 8829 | Total: UK: 313, US 1560 | T: 653, C: 259 |
| Branch | Army | All army | Army, Air force, Navy, Marine |
| Rank (% Enlisted) | NA | 90 | 88 |
| Active Duty Status (%) | 100 | NA | 0 |
| **Intervention** |  |  |  |
| Deployed from | USA | USA; UK | USA |
| Deployed to | Gulf War | OIF | Gulf war |
| Type of deployment | NA | War zone | War zone |
| **Contrast** |  |  |  |
| Military Comparison | Yes | Yes | Yes |
| Comparison | CE | CE (H vs L) | A |
| **Outcome** |  |  |  |
| Outcome | Depression | PTSD, alcohol | Depression |
| Outcome type | Continuous | Both | Continuous |
| Time point | Varies by deployment locations. | Data collected: 4 months – 1 year post | up to 1 year after return from deployment to Operation Desert Storm |
| Source | Questionnaire. BSI | PTSD (PCL), Alcohol (AUDIT-C) | Questionnaire: BDI,BSI |
| **Method** |  |  |  |
| Estimation | OLS | None | ANOVA (F-test) |
| **Effect size extraction** |  |  |  |
| ES extracted | 10 | 2 | 1 |
| Type of ES | SMD | OR | SMD |
| Notes | Unadjusted means extracted and converted to OR. Unclear whether regression adjusted coefficients are standardised. | US Data used. UK data sample overlap: British Iraq War Study. | Converted to OR. BSI Depression also available. Score 5 in confounding |

| **Study info** |  |  |  |
| --- | --- | --- | --- |
| Author(s) | Sutker, P. B., Uddo, M., Brailey, K., & Allain, J. | Sutker, P. B., Uddo, M., Brailey, K., Vasterling, J. J., and Errera, P. | Tackett DP; |
| Year | 1993 | 1994 | 2011 |
| **Participants** |  |  |  |
| Sample | Gulf reserve/guard |  | California National Guard Soldiers. |
| Samplesize | T: 215, C: 60 | T: 40, C: 20 | N = 223 |
| Branch | Army, Air Force | Army | NA |
| Rank (% Enlisted) | 87 | 97.5 | 97.5 |
| Active Duty Status (%) | None | None | 0 |
| **Intervention** |  |  |  |
| Deployed from | USA | USA | USA |
| Deployed to | Gulf War | Gulf War | OIF |
| Type of deployment | War zone | War zone | War zone |
| **Contrast** |  |  |  |
| Military Comparison | Yes | Yes | Yes |
| Comparison | CE (exposed vs not) | A; CE | CE |
| **Outcome** |  |  |  |
| Outcome | PTSD (PCL-M) | PTSD (DSM-III-R), Depression (BDI) , Drug abuse and alcohol abuse (SCID) | PTSD |
| Outcome type | Continuous | Dichotomous | Continuous |
| Time point | 4-10 months after return | NA | Data collected: 6-18 months post |
| Source | Questionnaire | Questionnaire | PTSD (PCL-M), |
| **Method** |  |  |  |
| Estimation | None (ANOVA), P.42 seems to suggest that they have tested for sex and race, and sex and race interactions. | None | Regression on log transformed variables |
| **Effect size extraction** |  |  |  |
| ES extracted | 4 | 16 | 1 |
| Type of ES | SMD | OR | Correlation with CE as dichotomous variable |
| Notes | Converted to OR. Score 5 in confounding | 0 | Converted to OR. Score 5 in confounding |

| **Study info** |  |  |  |
| --- | --- | --- | --- |
| Author(s) | Toomey, R., Kang, H. K., Karlinsky, J., Baker, D. G., Vasterling, J. J., Alpern, R. et al. | Trautmann S;Schönfeld S;Behrendt S;Höfler M;Zimmermann P;Wittchen HU; | Unwin, C., Blatchley, N., Coker, W., Ferry, S., Hotopf, M., Hull, L. et al. |
| Year | 2007 | 2014 | 1999 |
| **Participants** |  |  |  |
| Sample | Subsample of National Health Survey of Gulf War Era Veterans and Their Families |  | The British Gulf War Study |
| Samplesize | T: 1054, C: 1124 | T: 1483, C: 889 | Gulf: 3284, Bosnia: 1815, and Era: 2408. |
| Branch | NA | NA | Army, Navy, Air Force |
| Rank (% Enlisted) | NA | 87.2 | 86.6 |
| Active Duty Status (%) | NA | NA |  |
| **Intervention** |  |  | 98.8 |
| Deployed from | USA | Germany | UK |
| Deployed to | Gulf War | OEF | Gulf War |
| Type of deployment | War zone | War zone | War zone |
| **Contrast** |  |  |  |
| Military Comparison | Yes | Yes | Yes |
| Comparison | A | A | A; CE |
| **Outcome** |  |  |  |
| Outcome | PTSD (CAPS), Depression (BDI-II), Alcohol and drug abuse) | Alcohol, drugs | PTSD (Homemade, but based on Mississippi= |
| Outcome type | Continuous and dichotomous | Dichotomous | Dichotomous |
| Time point | Data collected: Jan. 1, 1991 - July 30, 1993 | 12 months post | Deployed: Sept. 1, 1990 - June 30, 1991. Data collected: August 1997 - November 1998 |
| Source |  | Interviews (DIA-X/M-CIDI) | Questionnaire |
| **Method** | Questionnaire |  |  |
| Estimation | Logistic regression | Logistic regression | Logistic regression |
| **Effect size extraction** |  |  |  |
| ES extracted | 5 | 2 | 1 |
| Type of ES | OR | OR | OR |
| Notes | Measure available for illicit substance (1) but RoB 5; MCS, PTSD Checklist mean score, Beck Depression (mean, and case) | 0 | Males only. Use Era comparison only |

| **Study info** |  |  |  |
| --- | --- | --- | --- |
| Author(s) | Unwin, C., Hotopf, M., Hull, L., Ismail, K., David, A., & Wessely, S. | Vanderploeg RD;Belanger HG;Horner RD;Spehar AM;Powell-Cope G;Luther SL;Scott SG; | Vasterling, J. J., Proctor, S. P., Amoroso, P., Kane, R., Heeren, T., & White, R. F. |
| Year | 2002 | 2012 | 2006 |
| **Participants** |  |  |  |
| Sample | The British Gulf War Study (women only) | Florida National Guard | Neurocognition Deployment Health Study |
| Samplesize | T: 236 (Gulf), 217 (Bosnia-only), C (Era): 192 | T: 1443, C:1655 | T: 654; C: 307. |
| Branch | Army, Navy, Air Force | NA | Army |
| Rank (% Enlisted) | 60.6 | NA | 97.9 |
| Active Duty Status (%) | None | 0 | 100 |
| **Intervention** |  |  |  |
| Deployed from | UK |  |  |
| Deployed to | Gulf War | USA | OIF |
| Type of deployment | War zone | OIF;OEF | NA |
| **Contrast** |  | War zone |  |
| Military Comparison | Yes |  | Yes |
| Comparison | A | Yes | A |
| **Outcome** |  | CE (H/M/L vs no) |  |
| Outcome | PTSD, CMD (GHQ-12) |  | PTSD (PTSD Checklist); Depression (POMS) |
| Outcome type | Dichotomous | Major Depression; PTSD; Excessive Drinking | Continous |
| Time point | Deployed: 1992 - 1996. Data collected: August 1997 - November 1998 | Dichotomous (3) | 1st: April 2003 - December 2003, 2nd: January 2005 - May 2005 |
| Source | Questionnaire | Surveys were completed an average of 31.8mth (SD=24.4, range=0-95) after deployment (p1887) | Questionnaire |
| **Method** |  | MDD: PHQ-9; PTSD: PCL-C; AUDIT |  |
| Estimation | Logistic regression | logistic regression | Means and Generalised Estimating Equation |
| **Effect size extraction** |  |  |  |
| ES extracted | 2 | 9 | 3 |
| Type of ES | OR | OR | SMD |
| Notes | CMD (GHQ-12) and PTSD. Use Era comparison only | Score 5 in confounding | Converted to OR. Depression ES (2). |

| **Study info** |  |  |  |
| --- | --- | --- | --- |
| Author(s) | Vinokur, A. D., Pierce, P. F., Lewandowski-Romps, L., Hobfoll, S. E., & Galea, S. | Voelker, M. D., Saag, K. G., Schwartz, D. A., Chrischilles, E., Clarke, W. R., Woolson, R. F. et al. | Wells, T. S., LeardMann, C. A., Fortuna, S. O., Smith, B., Smith, T. C., Ryan, M. A. K. et al. |
| Year | 2011 | 2004 | 2010 |
| **Participants** |  |  |  |
| Sample |  | The Iowa Gulf War Study data | Millennium Cohort Study |
| Samplesize | T: 657; C: 352 | Regular Military: T: 985, C: 968. Guard/Reserve: T: 911, C: 831 | T1: men: 3975, women: 744. T2: men: 3940, women: 891. C: men: 22126, women: 8543 |
| Branch | Air Force | Army, Navy, Air Force, Marines | Army, Navy, Air Force, Marines |
| Rank (% Enlisted) | 73.4 | 89.1 | 71.1 |
| Active Duty Status (%) | 25.5 | 53 |  |
| **Intervention** |  |  | 54.8 |
| Deployed from | USA | USA | USA |
| Deployed to | OIF, OEF | Gulf War | OIF, OEF |
| Type of deployment | War zone | War zone | War zone |
| **Contrast** |  |  |  |
| Military Comparison | Yes | Yes | Yes |
| Comparison | DE | A | A |
| **Outcome** |  |  |  |
| Outcome | PTSD (PCL-M) | CDM (MCS) | Depression (PHQ) |
| Outcome type | Continous | Continuous | Dichotomous |
| Time point | Deployed at: Oct 7, 2001 - Sept, 2004. T1: June 2005, T2: Sept. 2006. So T1: 0 - 4 years, T2: 1 - 5 years | Deployment: Aug. 2, 1990 – July 31, 1991 | Data collected: Baseline: 2001 – 2003. Follow up: 2004 - 2006 |
| Source | Questionnaire | Data collected: September 1995 – May 1996 | Questionnaire |
| **Method** |  | Questionnaire |  |
| Estimation | SEM; we use correlations | Linear regression | Logistic regression |
| **Effect size extraction** |  |  |  |
| ES extracted | 2 | 1 | 4 |
| Type of ES | Correlations with exposure vs not | SMD | OR |
| Notes | Converted to SMD to OR. Time 1 and 2. Score 5 in confounding | Raw SD available from text. Can then be converted to OR. | Separated by Combat/non-combat |

| **Study info** |  |
| --- | --- |
| Author(s) | Wolfe, J., Proctor, S. P., Erickson, D. J., Heeren, T., Friedman, M. J., Huang, M. T. et al. |
| Year | 1999 |
| **Participants** |  |
| Sample | Fort Devens |
| Samplesize | T: 178, C: 47 |
| Branch | Army |
| Rank (% Enlisted) | 86.2 |
| Active Duty Status (%) | 8.6 |
| **Intervention** |  |
| Deployed from | USA |
| Deployed to | Gulf War |
| Type of deployment | War zone |
| **Contrast** |  |
| Military Comparison | Yes |
| Comparison | CE (H vs L) |
| **Outcome** |  |
| Outcome | PTSD (CAPS, Mississippi), Depression (DSM-III-R, SCID) |
| Outcome type | Continuous |
| Time point | Deployment: 1991. Data collected: 1994 – 1996 |
| Source | Questionnaire |
| **Method** |  |
| Estimation | None / weighted to account for sampling scheme using SUDAAN |
| **Effect size extraction** |  |
| ES extracted | 8 |
| Type of ES | OR |
| Notes | 0 |

## Characteristics of Studies not in Analysis Sample

| **Study info** |  |  |  |
| --- | --- | --- | --- |
| Author(s) | Adler, Britt, Castro, McGurk and Bliese | Axelrod SR;Morgan CA;Southwick SM; | Barrett, D. H., Doebbeling, C. C., Schwartz, D. A., Voelker, M. D., Falter, K. H., Woolson, R. F. et al. |
| Year | 2011 | 2005 Feb | 2002 |
| **Participants** |  |  |  |
| Sample | NA | Connecticut National Guards deployed to Operation Desert Storm (Gulf War) | Iowa Persian Gulf Study Group/Iowa Gulf War Study |
| Samplesize | Total 1051 | N=94 (1mth); N=90 (6mths) | T: 1878, C: 1804 |
| Branch | NA | NA | Army, Navy, Air Force, Marines |
| Rank (% Enlisted) | NA | NA | 89 |
| Active Duty Status (%) | NA | 0 | NA |
| **Intervention** |  |  |  |
| Deployed from | USA | USA | USA |
| Deployed to | Iraq | Gulf war | Gulf War |
| Type of deployment | War zone | War zone | War zone |
| **Contrast** |  |  |  |
| Military Comparison | Yes | Yes | Yes |
| Comparison | CE | CE | A |
| **Outcome** |  |  |  |
| Outcome | PTSD | PTSD severity score | PTSD (PCL-M) |
| Outcome type | Continuous | Continuous | Dichotomous (from contionus scale) |
| Time point | 4 months post | 1 month and 6 months after returns (deployment to Gulf) | Deployed: Aug. 2, 1990 - July 31, 1991. Data collected: September 1995 - May 1996 |
| Source | PCL-S, 17 items. | Non-standard PTSD scale | Questionnaire |
| **Method** |  |  |  |
| Estimation | Adjusted correlation | OLS | Logistic regression (with sampling weights) |
| **Effect size extraction** |  |  |  |
| Type of ES | 0 | partial R2 | 0 |
| Notes | Sample overlap Sundin et al., 2014 (#27441) | Cannot convert to OR | Sample overlap: Iowa Persian Gulf Study Group. |

| **Study info** |  |  |  |
| --- | --- | --- | --- |
| Author(s) | Benda, B. B. & House, H. A. | Bramsen I;Dirkzwager AJE;van der Ploeg HM; | Bray RM;Pemberton MR;Lane ME;Hourani LL;Mattiko MJ;Babeu LA; |
| Year | 2003 | 2000 | 2010 |
| **Participants** |  |  |  |
| Sample |  | Dutch UN peacekeepers. | Department of Defense Health Related Behavior Surveys. |
| Samplesize | NA | N=572 | N =28,546 |
| Branch | NA | Army | Army, Navy, Marine, Air Force, Coast Guard. |
| Rank (% Enlisted) | NA |  | 82.8 |
| Active Duty Status (%) | NA | 97 | 100 |
| **Intervention** |  | NA |  |
| Deployed from | USA | Netherland | USA |
| Deployed to | NA | Yugoslavia | OIF;OEF |
| Type of deployment | NA | War zone | War zone |
| **Contrast** |  |  |  |
| Military Comparison | Yes | Yes | Yes |
| Comparison | CE | CE (continuous) | A; DE |
| **Outcome** |  |  |  |
| Outcome | PTSD (Clinican-Administered PTSD Scale) | PTSD severity score |  |
| Outcome type | Dichotomous | Continuous | PTSD, Depression, alcohol and drug use |
| Time point | Not reported | Data collected: 1996 Deployment: 1993-1995 | Dichotomous |
| Source | Questionnaire | Self-Rating Inventory for PTSD | Deployed: elsewhere or not deployed since 2001. Data collected: 2008 |
| **Method** |  |  | PTSD (PCL-C), Depression (a 3-item version-A Burnam depression screen. Alcohol (>5 drinks more than once a week) |
| Estimation | Logistic regression | OLS | Adjusted percentages |
| **Effect size extraction** |  |  |  |
| Type of ES | Marginal Effect (OR) | Marginal effect (mean) | OR |
| Notes | No SE of the estimate reported | Unclear if standardised | Number of observations by exposure not reported. |

| **Study info** |  |  |  |
| --- | --- | --- | --- |
| Author(s) | Browne T;Iversen A;Hull L;Workman L;Barker C;Horn O;Jones M;Murphy D;Greenberg N;Rona R;Hotopf M;Wessely S;Fear NT; | Castro CA;McGurk D; | Cesur, R., Sabia, J. J., & Tekin, E. |
| Year | 2008 | 2007 | 2011 |
| **Participants** |  |  |  |
| Sample | British Iraq War Study | MHAT-IV | The National Longitudinal Study of Adolescent Health |
| Samplesize | N=2916+662 | N=1320 | T: 1,110; C: 14,591 |
| Branch | NA | NA | NA |
| Rank (% Enlisted) | NA | 95 | NA |
| Active Duty Status (%) | 100 | 79 | NA |
| **Intervention** |  |  |  |
| Deployed from | UK | USA |  |
| Deployed to | OIF | OIF | Global War on Terror (GWOT) |
| Type of deployment | War zone | War zone | Combat operation |
| **Contrast** |  |  |  |
| Military Comparison | Yes | Yes | Yes |
| Comparison | CE | CE | A (combat vs. in US) DE (combat vs. non-combat outside US) |
| **Outcome** |  |  |  |
| Outcome | Heavy drinker | PTSD, depression, anxiety | Depression, PTSD |
| Outcome type | Dichotomous | Dichotomous | Dichotomous |
| Time point | 1-2 years post | While deployed | NA |
| Source | Alcohol: WHO-AUDIT; | DSM-IV-TR; PTSD checklist | Questionnaire |
| **Method** |  |  |  |
| Estimation | logistic regression | None | OLS + school fixed effects |
| **Effect size extraction** |  |  |  |
| Type of ES | 0 | OR | Risk difference |
| Notes | Sample overlap: British Iraq War Study | No post effect | Cannot convert to OR |

| **Study info** |  |  |  |
| --- | --- | --- | --- |
| Author(s) | Ciccone DS;Kline A; | Coughlin SS;Kang HK;Mahan CM; | Di Nicola M;Occhiolini L;Di Nicola L;Vellante P;Di Mascio R;Guizzardi M;Colagrande V;Ballone E; |
| Year | 2012 | 2011 | 2007 |
| **Participants** |  |  |  |
| Sample | Subsample of New Jersey National Guard Troops | National Health Survey of Gulf War Era Veterans and Thier Families. | Afghanistan Peace keeping |
| Samplesize | N=922 | T:6111, C: 3859 | T: 40 and C: 120 |
| Branch | NA | Air force, Army, Marines, Navy. | Army |
| Rank (% Enlisted) | NA | 84.3 | NA |
| Active Duty Status (%) | 0 | 35.7 | NA |
| **Intervention** |  |  |  |
| Deployed from | USA | USA | Italy |
| Deployed to | OIF | Gulf war | OEF |
| Type of deployment | War zone | War zone | NA |
| **Contrast** |  |  |  |
| Military Comparison | Yes | Yes | Yes |
| Comparison | CE | A | A |
| **Outcome** |  |  |  |
| Outcome | PTSD and Depression | PTSD | Depression, PTSD |
| Outcome type | Continuous | Dichotomous | Dichotomous |
| Time point | Data collected: approx 90 days after deployment | Data collected: 2003-2005. Deployed: 1991 | Data collected: April to June 2004 |
| Source | PTSD (PCL-C), Depression (PHQ-9 ) | PCL | Questionnaire |
| **Method** |  |  |  |
| Estimation | Correlation | Logistic regression | None |
| **Effect size extraction** |  |  |  |
| Type of ES | Correlation | 0 | OR |
| Notes | Cannot convert to OR | Sample overlap: National Health of Gulf War Era Veterans and Their Families. | 5 in confounding |

| **Study info** |  |  |  |
| --- | --- | --- | --- |
| Author(s) | Dlugosz, L. J., Hocter, W. J., Kaiser, K. S., Knoke, J. D., Heller, J. M., Hamid, N. A. et al. | Dryden AE; | Dutra, L. I. S. S., Grubbs, K. A. T. H., Greene, C. A. R. O., Trego, L. L., McCartin, T. L., Kloezman, K. A. R. E. et al. |
| Year | 1999 | 2013 | 2011 |
| **Participants** |  |  |  |
| Sample |  | MIRECC |  |
| Samplesize | NA | N=1824 | NA |
| Branch | Army, Navy, Air Force, Marines | NA | Army |
| Rank (% Enlisted) | NA | NA | 87.1 |
| Active Duty Status (%) | All | NA | All |
| **Intervention** |  |  |  |
| Deployed from | USA | USA | USA |
| Deployed to | Gulf War | OIF;OEF | OIF |
| Type of deployment | War zone | War zone | War zone |
| **Contrast** |  |  |  |
| Military Comparison | Yes | Yes | Yes |
| Comparison | A | CE | CE |
| **Outcome** |  |  |  |
| Outcome | Alcohol Disorder, Drug Disorder, Depression, PTSD | PTSD, depression, drug and alcohol | PTSD (Primary Care PTSD Screen), Depression (CES-D) |
| Outcome type | Dichotomous | Continuous | Dichotomous |
| Time point | Deployment: Aug. 2, 1990 - July 31, 1991. Data collected: June 1, 1991 – Sept. 30, 1993 | Not reported | NA |
| Source | Hospitalization records from DMDC Biometric data files. ICD-9-CM classifications converted into 10 mental disorders | Alcohol (AUDIT), Depression (BDI-II), PTSD (DTS), Drug (DAST) | Questionnaire |
| **Method** |  |  |  |
| Estimation | Cox Proportional Hazard Model | Pearson correlation | OLS (essentially Linear probability Model) |
| **Effect size extraction** |  |  |  |
| Type of ES | HR | 0 | Marginal effects (Mean) |
| Notes | Hazard ratio | Sample overlap: (VISN 6 MIRECC). Risk of Bias (5) | No SD for CES-D reported |

| **Study info** |  |  |  |
| --- | --- | --- | --- |
| Author(s) | Edwards RD; | Elbogen EB;Sullivan CP;Wolfe J;Wagner HR;Beckham JC; | Engdahl, R. M., Richardson, J. D., Elhai, J. D., & Frueh, B. C |
| Year | 2012 | 2013 | 2011 |
| **Participants** |  |  |  |
| Sample | 2010 National Survey of Veterans | Veterans from the Iraq/Afghanistan era either separated from active duty or in the reserves or national guard. |  |
| Samplesize | T: 453, C: 300 | N=1090. | T: 746 C: 320 |
| Branch | Army, Navy, Air Force, Marines, Coast Guard. | Army, Air Force, Navy, Marines, Coast Guard | NA |
| Rank (% Enlisted) | NA | 81.5 | NA |
| Active Duty Status (%) | NA | 52 | NA |
| **Intervention** |  |  |  |
| Deployed from | USA | USA | Canada |
| Deployed to | OIF;OEF | OIF;OEF | NA |
| Type of deployment | War zone | War zone | NA |
| **Contrast** |  |  |  |
| Military Comparison | Yes | Yes | Yes |
| Comparison | A | #I/T | A |
| **Outcome** |  |  |  |
| Outcome | Employment | Homelessness | PTSD (PCL-M) |
| Outcome type | Continuous | Dichotomous | Continuous |
| Time point | Deployed and survey in 2010 | Data collected: 1- 8 years after deployment | Deployment: after 1990. Data collected: 1999 |
| Source | Questionnaire | Homeless for at least one night in the past year | Questionnaire |
| **Method** |  |  |  |
| Estimation | Propensity score matching | None | None |
| **Effect size extraction** |  |  |  |
| Type of ES | Mean difference | 0 | 0 |
| Notes | No SE on the estimate reported. | 5 in confounding. | Sample overlap: Veteran Affairs Canada, 1999. Risk of Bias (5). |

| **Study info** |  |  |  |
| --- | --- | --- | --- |
| Author(s) | Engelhard, I. M. & van den Hout, M. A. | Fear NT;Jones M;Murphy D;Hull L;Iversen AC;Coker B;Machell L;Sundin J;Woodhead C;Jones N;Greenberg N;Landau S;Dandeker C;Rona RJ;Hotopf M;Wessely S; | Fritch AM;Mishkind M;Reger MA;Gahm GA; |
| Year | 2007 | 2010 | 2010 |
| **Participants** |  |  |  |
| Sample |  | British Iraq War | Veterans attending a military outpatient behavioral health clinic |
| Samplesize | N=276 | T: 5743, C: 2518; | N=1045 |
| Branch | Army | Naval Services, Army, Royal Air Force | Army |
| Rank (% Enlisted) | NA | 77.8 | 92 |
| Active Duty Status (%) | NA | 82.9 | 71.5 |
| **Intervention** |  |  |  |
| Deployed from | Netherlands | UK | USA |
| Deployed to | OIF | OIF;OEF | OIF;OEF |
| Type of deployment | NA | War zone | War zone |
| **Contrast** |  |  |  |
| Military Comparison | Yes | Yes | Yes |
| Comparison | CE | A; CE | CE |
| **Outcome** |  |  |  |
| Outcome | PTSD (DSM-IV, SCID) | CMD, PTSD, Alcohol | PTSD; Depression; Alcohol abuse |
| Outcome type | Continuous | Dichotomous (3) | Unclear |
| Time point | NA | Assessed the effect of deployment to Iraq and Afghanistan from 2003 to the end of data collection (sept 2009). (P1783). | Data collected: May 18, 2006-July 25, 2007 |
| Source | Questionnaire | CDM: GHQ-12; PTSD: PCL-C; Alcohol: WHO-AUDIT | PTSD: PCL-C; Depression: PHQ-9; Alcohol: AUDIT |
| **Method** |  |  |  |
| Estimation | Correlation | Logistic regression | OLS continuous CE |
| **Effect size extraction** |  |  |  |
| Type of ES | partial correlation | OR | Marginal effect (mean) |
| Notes | Cannot convert to OR | Sample overlap, British Iraq War Study |  |

| **Study info** |  |  |  |
| --- | --- | --- | --- |
| Author(s) | Gackstetter, G. D., Hooper, T. I., Al Qahtani, M. S., Smith, T. C., Memish, Z. A., Schlangen, K. M. et al. | Gade and Wenger | Garber BG;Zamorski MA;Jetly CR; |
| Year | 2005 | 2011 | 2012 |
| **Participants** |  |  |  |
| Sample |  | The National Survey of Veterans (NSV) | Canadian Forces |
| Samplesize | T: 8,334; C: 7,270 | T: 394, C: 641 | N=1572 (out of 2779) |
| Branch | NA | Army, Marines | NA |
| Rank (% Enlisted) | 87.3 | 89.7 | 84 |
| Active Duty Status (%) | None | NA | 86 |
| **Intervention** |  |  |  |
| Deployed from |  |  | Canada |
| Deployed to | Saudi Arabia (Specific Al Khafji and the Riyadh Area | Gulf War | OEF |
| Type of deployment | Combat Operations | NA | War zone |
| **Contrast** |  |  |  |
| Military Comparison | Yes | Yes | Yes |
| Comparison | DE | CE (combat/not combat) | CE (arranged in tertiles) |
| **Outcome** |  |  |  |
| Outcome | CMD (codes 290-319 on ICD-9-CM) | CMD (MCS) | PTSD, Depression |
| Outcome type | Dichotomous | Continuous | Dichotomous |
| Time point | Deployment: Jan. 1, 1991 – Feb. 1, 1991 | February 2001 – November 2001 | Deployed: Feb 15, 2010 - March 15, 2010. Stationed between 6-9mths |
| Source | Administrative data | Questionnaire | Questionnaire |
| **Method** |  |  |  |
| Estimation | Cox proportional hazards model | Discrete factor maximum likelihood estimation. | Logistic regression |
| **Effect size extraction** |  |  |  |
| Type of ES | OR | Adjusted Mean difference | OR |
| Notes | 5 in confounding and cannot calculate relevant ES | No unadjusted SD available | 5 in confounding and cannot calculate relevant ES |

| **Study info** |  |  |  |
| --- | --- | --- | --- |
| Author(s) | Gray GC;Coate BD;Anderson CM;Kang HK;Berg SW;Wignall FS;Knoke JD;Barrett-Connor E; | Gray GC;Kaiser KS;Hawksworth AW;Hall FW;Barrett-Connor E; | Han SC;Castro F;Lee LO;Charney ME;Marx BP;Brailey K;Proctor SP;Vasterling JJ; |
| Year | 1996 | 1999 | 2014 |
| **Participants** |  |  |  |
| Sample | US regular active duty Gulf War Veterans, and comparison sample | US Seabees (Naval construction workers) | National Guardsoldiers enrolled in the NDHS |
| Samplesize | T: 547076; C: 618335 | T: 527, C: 970 | N=1008 |
| Branch | Army, Navy, Marine Corps, Air Force | Navy | Army |
| Rank (% Enlisted) | NA | NA | 97.7 |
| Active Duty Status (%) | 1 | NA | 82.8 |
| **Intervention** |  |  |  |
| Deployed from | USA | USA | USA |
| Deployed to | Gulf War | Gulf war | OIF |
| Type of deployment | War zone | War zone | War zone |
| **Contrast** |  |  |  |
| Military Comparison | Yes | Yes | Yes |
| Comparison | Unclear | A | CE |
| **Outcome** |  |  |  |
| Outcome | Alcohol , PTSD, depression, Drug dependence | PTSD; Depression | PTSD |
| Outcome type | dichotomous | Dichotomous and Continous | Continuous |
| Time point | Hospitalisations: 1992, first 9 months of 1993 | Data collected: 1994- 1995. Deployment ended in March 1991 | Data collected: ~73 days post, and ~197.5 days post. Deployed: Dec 2003 –Feb 2005 |
| Source | ICD-9-CM codes | PTSD: not specified, Depression: Hopkins Symptom Checklist, 58-item, self-report. | PTSD (PCL-C) |
| **Method** |  |  |  |
| Estimation | Rate ratios using direct method to standardize | unadjusted odds ratios | Hierarchical multiple regression |
| **Effect size extraction** |  |  |  |
| Type of ES | Rate Ratio | 0 | 0 |
| Notes | Sample overlap (US Gulf War). 5 in confounding | Sample overlap: US Seabees, Gray et al, 2002 (#22996). Risk of Bias (5) | Sample overlap: NDHS |

| **Study info** |  |  |  |
| --- | --- | --- | --- |
| Author(s) | Harmon SC;Hoyt TV;Jones MD;Etherage JR;Okiishi JC; | Heron EA;Bryan CJ;Dougherty CA;Chapman WG; | Hoge, C. W., Castro, C. A., Messer, S. C., McGurk, D., Cotting, D. I., & Koffman, R. L. |
| Year | 2012 | 2013 | 2004 |
| **Participants** |  |  |  |
| Sample | Soldiers at Schofield Barracks, Hawaii at their PDHRA upon return from Iraq | Active duty Air Force vehicle operators, who attended basic combat convoy course in Iraq. | Random sample with overlap of population to ref 6405 |
| Samplesize | N=2319 | N=168 | C: 2530; T1: 1962; T2: 894; T3: 815 |
| Branch | All army | Air force | Army, Marines |
| Rank (% Enlisted) | 91.9 | 55.9 | 93.2 |
| Active Duty Status (%) | NA | 100 | All |
| **Intervention** |  |  |  |
| Deployed from | USA | USA | USA |
| Deployed to | OIF | OIF | OIF, OEF |
| Type of deployment | War zone | War zone | War zone |
| **Contrast** |  |  |  |
| Military Comparison | Yes | Yes | Yes |
| Comparison | CE (latent construct) | CE | A |
| **Outcome** |  |  |  |
| Outcome | PTSD; Depression; Alcohol abuse | PTSD and depression | Depression (PHQ), PTSD (PCL-C), Alcohol abuse |
| Outcome type | Unclear | Continuous | Dichotomous |
| Time point | Data collected: median time since return was 4 months | Data collected predeployment and 1,3,6 and 12 months postdeployment. | Data collected: 2003 |
| Source | PTSD (PC-PTSD), Depression (PHQ-9), Alcohol: WHO-AUDIT | PTSD (PCL-M), depression (PHQ-9) | Questionnaire |
| **Method** |  |  |  |
| Estimation | SEM | Generalized linear mixed-effects modeling with repeated measures, random intercept, and random effect of time | Odds ratios |
| **Effect size extraction** |  |  |  |
| Type of ES | Marginal effect | Marginal Effect (means) | 0 |
| Notes | No standard error or p-value reported. | Standardised regression coefficients only | Overlapping sample Hoge et al, 2006 (#6405). Risk of Bias (5). |

| **Study info** |  |  |
| --- | --- | --- |
| Author(s) | Hooper, R., Rona, R. J., Jones, M., Fear, N. T., Hull, L., & Wessely, S. | Horton, Jacobson, Wong, Wells, Boyko,Smith, Ryan & Smith |
| Year | 2008 | 2013 |
| **Participants** |  |  |
| Sample | A random sample of The British Iraq War Study population | Millennium Cohort Study. First (2001-2003) and second (2004-2006) panel. Those separated from military between baseline and FU |
| Samplesize | Era: 505; Era deployed: 106; TELIC1: 171; TELIC2: 159. | N=9099 |
| Branch | NA | Army; Navy/Coast Guard; Air Force; Marine Corps |
| Rank (% Enlisted) | NA | 78.4 |
| Active Duty Status (%) | NA | 100 |
| **Intervention** |  |  |
| Deployed from | UK | USA |
| Deployed to | OIF | OIF;OEF |
| Type of deployment |  | War zone |
| **Contrast** | War zone |  |
| Military Comparison |  | Yes |
| Comparison | Yes | A |
| **Outcome** | CE |  |
| Outcome |  | Unemployment |
| Outcome type | Alcohol | Dichotomous |
| Time point | Continuous | Not reported |
| Source | Measurements: 1st: 2002 (before treat were deployed), 2nd: "Roughly three years later"… 2005 | Questionnaire (Employment Yes/No/Homemaker) |
| **Method** | Questionnaire |  |
| Estimation |  | Logistic regression |
| **Effect size extraction** | OLS (on first differences) |  |
| Type of ES | 0 | 0 |
| Notes | Sample overlap. The British Iraq War Study population | Risk of Bias (5). Only 2 studies for this outcome (both RoB 5). No synthesis. |

| **Study info** |  |  |  |
| --- | --- | --- | --- |
| Author(s) | Hoyt T;Renshaw KD; | Iowa Persian Gulf Study Group | Ishoy T;Suadicani P;Andersson AM;Guldager B;Appleyard M;SkakkebÇÝk NE;Gyntelberg F; |
| Year | 2014 | 1997 | 2001 Mar |
| **Participants** |  |  |  |
| Sample | Sample is a subset of Hoyt, Renshaw & Pasupathi, 2013 | Iowa Persian Gulf Study Group/Iowa Gulf War Study | Danish Gulf War Study |
| Samplesize | N= 81 | Regular: T: 985, C: 968. Guard/Reserve: T: 911, C: 831 | T=661; C=215 |
| Branch | Army | Army, Navy, Air Force, Marines | NA |
| Rank (% Enlisted) | NA | 90.3 | NA |
| Active Duty Status (%) | 0 | 52 | NA |
| **Intervention** |  |  |  |
| Deployed from | USA | USA | Denmark |
| Deployed to | OIF;OEF | Gulf War | Gulf war |
| Type of deployment | War zone | War zone | War zone |
| **Contrast** |  |  |  |
| Military Comparison | Yes | Yes | Yes |
| Comparison | CE (continuous) | A | A |
| **Outcome** |  |  |  |
| Outcome | PTSD | Depression (PRIME-MD), PTSD (PCL), Alcohol Abuse (PRIME-MD, CAGE) | Alcohol Use, |
| Outcome type | Continuous | Dichotomous | Continuous (1) |
| Time point | Time 1: 3 months after deployment. Time 2: 7 - 9 months after deployment. | Deployment: Aug. 2, 1990 - July 31, 1991. Data collected: Sep 1995 - May 1996 | Data collected: 1997-1998. Deployment: 1990-1997 |
| Source | PTSD (PCL-M) | Questionnaire | questionnaire |
| **Method** |  |  |  |
| Estimation | Regression (using mean centered variables) | CMH-test Cochran-Mantel-Haenszel rate differences | t-test |
| **Effect size extraction** |  |  |  |
| Type of ES | Marginal effect | 0 | 0 |
| Notes | Marginal effect of DRRI. | Sample overlap: Iowa Persian Gulf Study Group. | Sample overlap: Danish Gulf War Study. Male subsample only. Risk of Bias (5). |

| **Study info** |  |  |  |
| --- | --- | --- | --- |
| Author(s) | Ishøy, Knop, Suadicani, Guldager, Appplyard, Gyntelberg | Ismail, K., Kent, K., Brugha, T., Hotopf, M., Hull, L., Seed, P. et al. | Iversen AC;van SL;Hughes JH;Browne T;Hull L;Hall J;Greenberg N;Rona RJ;Hotopf M;Wessely S;Fear NT; |
| Year | 2004 | 2002 | 2009 |
| **Participants** |  |  |  |
| Sample |  | The British Gulf War Study, only disabled | The British Iraq War Study |
| Samplesize | T: 686; C: 231 | T: 111, C: 133 | N=821 |
| Branch | NA | Na | NA |
| Rank (% Enlisted) | NA | 96 | NA |
| Active Duty Status (%) | NA | NA | 50 |
| **Intervention** |  |  |  |
| Deployed from | Denmark | UK | UK |
| Deployed to | Gulf War | Gulf War | OIF |
| Type of deployment | War zone | War zone | War zone |
| **Contrast** |  |  |  |
| Military Comparison | Yes | Yes | Yes |
| Comparison | A | DE | A |
| **Outcome** |  |  |  |
| Outcome | Alcohol, Drugs, Depression (SCL-90-R) | PTSD, anxiety, alcohol, Any Psychiatric (Depression, anxiety + alcohol) | PTSD symptoms |
| Outcome type | Continous: Alcohol Dichotomous: Drugs. Cardinal: Depression dimension of SCL-90-R. | Dichotomous | Dichotomous |
| Time point | Deployment: Aug. 2, 1990 - Dec. 31, 1997. Data collection: Jan 1997 - Jan 1998 | Data gathered 1999 and 2000, treated deployed 1990-1991 and controls are Bosnia deployed (1992-1997) and Gulf era non-deployed | Data collected: 2006-2007. |
| Source | Questionnaire | Questionnaire: The World Health Organization's schedule of clinical assessment in neuropsychiatry (version 2.1). | PTSD: Primary Care Screen; |
| **Method** |  |  |  |
| Estimation | Matched sample | Logistic regression | logistic regression |
| **Effect size extraction** |  |  |  |
| ES extracted | 0 | 0 | 0 |
| Notes | Sample overlap: Danish Gulf War Study (Operation Desert Peace) | Overlapping sample: British Gulf War Study. Disabled only subsample. Risk of Bias (5). | Sample overlap: British Iraq War Study. Risk of Bias (5). |

| **Study info** |  |  |  |
| --- | --- | --- | --- |
| Author(s) | Jacobson I;Horton J;LeardMann C;Ryan M;Boyko E;Wells T;Smith B; | Jacobson IG;Horton JL;LeardMann CA;Ryan MAK;Boyko EJ;Wells TS;Smith B;Smith TC; | James LM;Van KE;Miller RD;Engdahl BE; |
| Year | 2012 | 2012 | 2013 |
| **Participants** |  |  |  |
| Sample | Millenium Cohort Study | Millennium Cohort Study | OIF/OEF veterans who registered for VA healthcare subsequent to their deployment. |
| Samplesize | N=1492 | N=1,492 (table 4,p621) | N=271. |
| Branch | Army, Navy/Coast Guard, Air Force | Army, Navy / Coast Guard, Air Force | Army, Navy, Air force, Marines |
| Rank (% Enlisted) | 61.3 | 61.3 | NA |
| Active Duty Status (%) | 50.1 | 50.1 | 21 |
| **Intervention** |  |  |  |
| Deployed from | USA | USA | USA |
| Deployed to | OIF;OEF | OIF;OEF | OIF;OEF |
| Type of deployment | War zone | War zone | War zone |
| **Contrast** |  |  |  |
| Military Comparison | Yes | Yes | Yes |
| Comparison | CE | A; CE | CE (continuous) |
| **Outcome** |  |  |  |
| Outcome | Positive screen for PTSD or Depression | New onset PTSD or depression | PTSD, depression and alcohol |
| Outcome type | Dichotomous | Dichotomous | Continuous |
| Time point | New positive screen in period 2001-2008 | Deployed: 2001-2009 Data collected: 2001-2006 | Data collected: 6, 12 and 24 months post deployment |
| Source | PTSD: PCL-C, Depression: PHQ-9 | Questionnaire: PTSD (PCL-C), Depression (PHQ-9). | PTSD (PCL-C), Depression (BDI-SF), Alcohol (AUDIT), |
| **Method** |  |  |  |
| Estimation | general estimating equations | Logistic regression | regression |
| **Effect size extraction** |  |  |  |
| Type of ES | 0 | 0 | Marginal Effect |
| Notes | Sample overlap: Millenium Cohort Study. | Sample overlap: Millenium Cohort Study. | SD not reported |

| **Study info** |  |  |  |
| --- | --- | --- | --- |
| Author(s) | Kang HK;Li B;Mahan CM;Eisen SA;Engel CC; | Kang, H. K., Mahan, C. M., Lee, K. Y., Magee, C. A., & Murphy, F. M. | Kelley ML;Runnals J;Pearson MR;Miller M;Fairbank JA;Brancu M; |
| Year | 2009 | 2000 | 2013 |
| **Participants** |  |  |  |
| Sample | National Health Survey of Gulf War Era Veterans and Their Families | National Health Survey of Gulf War Era Veterans and Their Families. Subsample used in 11941 | VISN 6 MIRECC. |
| Samplesize | T: 6111; C: 3859 | T: 11441; C: 9476 | N= 1825 |
| Branch | Air Force, Army, Marine, Navy. | Army, Navy, Air Force, Marines | Army, Navy, Air Force, Marines, Coast Guard |
| Rank (% Enlisted) | 84.3 | 83.9 | NA |
| Active Duty Status (%) | 35.7 | 38.6 | 49.6 |
| **Intervention** |  |  |  |
| Deployed from | USA | USA | USA |
| Deployed to | Gulf war | Gulf War | OIF;OEF |
| Type of deployment | War zone | War zone | War zone |
| **Contrast** |  |  |  |
| Military Comparison | Yes | Yes | Yes |
| Comparison | A | A | CE (continuous) |
| **Outcome** |  |  |  |
| Outcome | Depression, PTSD, MDD, Alcohol Abuse | Depressed | PTSD, depression and alcohol |
| Outcome type | Dichotomous (5) | Dichotomous | Continuous |
| Time point | Deployed: 1991. Surveyed up until 2005. | Deployed: 1991. Data collected: 1995 | All deployed after 2001. Data collected: 2001-2012. 0 - 11 years after deployment |
| Source | Depression (Doctor): Self-report; PTSD (PCL-C), MDD (PHQ-9), Alcohol (PHQ) | Questionnaire | Alcohol (AUDIT), Depression (BDI-2), PTSD (DTS). |
| **Method** |  |  |  |
| Estimation | The exact method is uncertain. | Cochran-Mantel-Haenszel rate difference | Structural equation model |
| **Effect size extraction** |  |  |  |
| Type of ES | 0 | 0 | Marginal Effect |
| Notes | Sample overlap: National Health of Gulf War Era Veterans and Their Families | Sample overlap: National Health of Gulf War Era Veterans and Their Families | Sample overlap: Mid-Atlantic Mental Illness Research, Education, and Clinical Center (VISN 6 MIRECC) data |

| **Study info** |  |  |  |
| --- | --- | --- | --- |
| Author(s) | Kelsall, H. L., Sim, M. R., Forbes, A. B., Glass, D. C., McKenzie, D. P., Ikin, J. F. et al. | Lane ME;Hourani LL;Bray RM;Williams J; | Larson, G. E., Highfill-McRoy, R. M., & Booth-Kewley, S. |
| Year | 2004 | 2012 | 2008 |
| **Participants** |  |  |  |
| Sample | Same sample as 928, 1104, 1122 and 1159 | US Department of Defense Surveys of Health-Related Behaviors. |  |
| Samplesize | T: 1414; C: 1411 | T:16146; C: 15212 | T: 41,561, C: 59,595 |
| Branch | Army, Navy, Air Force | Army, Army and Air National Guard, Navy, Marines, Air Force. | Marines |
| Rank (% Enlisted) | 81.1 | 84.9 | NA |
| Active Duty Status (%) | NA | 51.5 | All |
| **Intervention** |  |  |  |
| Deployed from | Australia | USA | USA |
| Deployed to | Gulf War | OIF;OEF | OIF, OEF |
| Type of deployment | War zone | War zone | War zone, Combat zone |
| **Contrast** |  |  |  |
| Military Comparison | Yes | Yes | Yes |
| Comparison | CE | A | A |
| **Outcome** |  |  |  |
| Outcome | Depression, PTSD |  | Substance related disorders, Depression, PTSD (TRICARE) |
| Outcome type | Dichotomous | Depression; General Anxiety; PTSD | Dichotomous |
| Time point | Deployment: Aug. 2, 1990 – Sept. 4, 1991. Data collection: August 2000 - April 2002 | Dichotomous (3) | Deployed July 2001 - Sept. 2005 |
| Source | Questionnaire | Data collected: 2005-2006. Deployment status in last 36 months | TRICARE |
| **Method** |  | Depression: 3-item Version A Burnam depression screen, PTSD: PCL-C |  |
| Estimation | Logistic regression | OLS (with weights). | "Relative risks adjusted were estimated using Mantel-Haenszel confounder adjusted odds ratio" |
| **Effect size extraction** |  |  |  |
| Type of ES | 0 | SMD | Hazard ratio |
| Notes | Sample overlap: Australian Gulf veterans. | N's not stratified by Deployment/Not-deployed. No ES can be extracted | HR |

| **Study info** |  |  |  |
| --- | --- | --- | --- |
| Author(s) | LeardMann CA;Smith TC;Smith B;Wells TS;Ryan MAK; | Li, Mahan, Kang, Eisen & Engel | Lindem, K., Heeren, T., White, R. F., Proctor, S. P., Krengel, M., Vasterling, J. et al. |
| Year | 2009 | 2011 | 2003 |
| **Participants** |  |  |  |
| Sample | Millennium Cohort Study | National Health Survey of Gulf War Era Veterans and Their Families. | Fort Devens |
| Samplesize | T1: 1478, T2: 1044, T3: 2888 | T: 5,469, C: 3,353 | T: 178, C: 47 |
| Branch | Not reported | Air Force, Army, Marine Corps, Navy. | NA |
| Rank (% Enlisted) | NA | 17 | NA |
| Active Duty Status (%) | NA | 35 | NA |
| **Intervention** |  |  |  |
| Deployed from | USA | USA | USA |
| Deployed to | OIF;OEF | Gulf war | Gulf War |
| Type of deployment | War zone | War zone | War zone |
| **Contrast** |  |  |  |
| Military Comparison | Yes | Yes | Yes |
| Comparison | CE (2 exp vs 1 og 3 vs 1) | A | DE |
| **Outcome** |  |  |  |
| Outcome | New onset PTSD | PTSD | Depression (DSM-III-R, SCID), PTSD (CAPS) |
| Outcome type | Dichotomous | Dichotomous | Dichotomous |
| Time point | Data collected: 1-5 years after deployment. Baseline: 2001 - 2003. Followup: 2004 - 2006 | Gulf War (1991) survey in 1995 and 2005 | Deployment:1991. Data collected: 1994 and 1996. |
| Source | Questionnaire: PTSD (PCL-C), | Questionnaire: PTSD (PCL-C) | Questionnaire |
| **Method** |  |  |  |
| Estimation | Logistic regression | Mantel-Haenszel method. | None |
| **Effect size extraction** |  |  |  |
| Type of ES | 0 | 0 | 0 |
| Notes | Sample overlap: Millenium Cohort Study. | Sample overlap: National Health of Gulf War Era Veterans and Their Families. | Sample overlap: The Fort Devens Operation Desert Storm Reunion Survey. Risk of Bias (5) |

| **Study info** |  |  |  |
| --- | --- | --- | --- |
| Author(s) | Litz BT;Orsillo SM;Friedman M;Ehlich P;Batres A; | Litz, B. T., King, L. A., King, D. W., Orsillo, S. M., & Friedman, M. J. | Luxton DD;Skopp NA;Maguen S; |
| Year | 1997 | 1997 | 2010 |
| **Participants** |  |  |  |
| Sample | US Peacekeeping in Somalia | Litz et al.'s (1997) Somalia veteran survey | Soldier deployed in support of operations to OEF/OIF |
| Samplesize | N=3461 | N=1650 | N=6943 |
| Branch | NA | Army, Marines | NA |
| Rank (% Enlisted) | 93 | 92 | 90.2 |
| Active Duty Status (%) | NA | All | 100 |
| **Intervention** |  |  |  |
| Deployed from | USA | USA | USA |
| Deployed to | Somalia | Somalia | OIF;OEF |
| Type of deployment | Civil conflict | War zone | War zone |
| **Contrast** |  |  |  |
| Military Comparison | Yes | Yes | Yes |
| Comparison | CE | CE | CE |
| **Outcome** |  |  |  |
| Outcome | (probable) PTSD | PTSD (Mississippi) | PTSD, Depression |
| Outcome type | Continuous | Continuous | Continuous (2) |
| Time point | approx 5 mths after return to US | Average of 15 weeks after their return | predeployment: 45-120 days. Post: 90-180 days Time: March 2006 - July 2009 |
| Source | PTSD: PTSD checklist and Mississippi scale. Composite index used. | Questionnaire | PTSD: PTSD Primary Care Screen; Depression: PHQ-9 |
| **Method** |  |  |  |
| Estimation | OLS | Structural equation model | OLS. Raw mean and SD reported but exposure continuous |
| **Effect size extraction** |  |  |  |
| Type of ES | Marginal effect (mean) | Marginal effects (SEM) | Marginal effects |
| Notes | 0 | Latent PTSD measure. Standardised coefficients. | Cannot calculate OR |

| **Study info** |  |  |  |
| --- | --- | --- | --- |
| Author(s) | MacGregor, Heltemes, Clouser, Han & Gaiarneau | Maguen S;Litz BT;Wang JL;Cook M; | Maguen, S., Lucenko, B. A., Reger, M. A., Gahm, G. A., Litz, B. T., Seal, K. H. et al. |
| Year | 2014 | 2004 Mar | 2010 |
| **Participants** |  |  |  |
| Sample | 0 | Peacekeepers to Kosovo, | OIF soldiers |
| Samplesize | N = 3512 | N=203 | NA |
| Branch | U.S. Marines | NA | NA |
| Rank (% Enlisted) | NA | 88 | NA |
| Active Duty Status (%) | NA | 100 | NA |
| **Intervention** |  |  |  |
| Deployed from | USA | USA | USA |
| Deployed to | OIF;OEF | Kosovo | OIF |
| Type of deployment | War zone | Civil conflict | War zone |
| **Contrast** |  |  |  |
| Military Comparison | Yes | Yes | Yes |
| Comparison | CE | CE | CE (continuous) |
| **Outcome** |  |  |  |
| Outcome | PTSD, depression | PTSD, depression and alcohol | PTSD (PC-PTSD), Depression (PHQ-9), Alcohol abuse (AUDIT) |
| Outcome type | - | Continuous | Continuous |
| Time point | Deployed: 2005- 2008. Data collected: within 2 months after second deployment. | An average of 7 months after return (p199) | NA |
| Source | Questionnaire: PTSD: (PC-PTSD), Depression: (PHQ), | PTSD (PCL), Depression (BSI), alcohol consumption | Questionnaire |
| **Method** |  |  |  |
| Estimation |  | Hierarchical regression | Multiple regression |
| **Effect size extraction** | Logistic regression |  |  |
| Type of ES | Marginal effect (logistic) | Correlation | Marginal Effect (mean) |
| Notes |  | Cannot convert to OR | No SD reported. Unclear whether regression coefficients are standardised. |

| **Study info** |  |  |  |
| --- | --- | --- | --- |
| Author(s) | Marx, B. P., Doron-Lamarca, S., Proctor, S. P., & Vasterling, J. J. | McKenzie, D. P., Ikin, J. F., McFarlane, A. C., Creamer, M., Forbes, A. B., Kelsall, H. L. et al. | Pierce MD;Wood MD;Reddy M;Sevin E;Shea MT; |
| Year | 2009 | 2004 | 2012 |
| **Participants** |  |  |  |
| Sample | Neurocognition Deployment Health Study | Same sample as Ikin et al., 2004 (#1159) | Rhode Island National Guard and Reserve units. Following OEF/OIF deployments. |
| Samplesize | N=668 | T: 1374; C: 1513 | N= 159 |
| Branch | Army | Army, Navy, Air Force | NA |
| Rank (% Enlisted) | 98.1 | 81.1 | NA |
| Active Duty Status (%) | All | NA | 0 |
| **Intervention** |  |  |  |
| Deployed from | USA | Australia |  |
| Deployed to | OIF | Gulf War | USA |
| Type of deployment |  | War zone | OIF;OEF |
| **Contrast** | War zone |  | War zone |
| Military Comparison |  | Yes |  |
| Comparison | Yes | A | Yes |
| **Outcome** | CE |  | CE (H/M/L) |
| Outcome |  | CMD (MCS-12, GHQ-12); PTSD (PCL-S) |  |
| Outcome type | PTSD (PCL) | Continuous: MCS-12; Dichotomous: GHQ-12 , PCL-S | PTSD |
| Time point | Continuous | Deployment: Aug. 2, 1990 – Sept. 4, 1991. Data collection: August 2000 - April 2002 | Continuous |
| Source | Time 2= 73.5 days after tour | Questionnaire | Unclear |
| **Method** | Questionnaire |  | Interview. PTSD (CAPS) |
| Estimation |  | Logistic regression (GHQ-12, PCL-S); OLS (MCS-12) | None |
| **Effect size extraction** | Multiple linear regression |  |  |
| ES extracted |  | 0 | 1 |
| Type of ES | 0 | 0 | Mean |
| Notes | Sample overlap, Vasterling et al., 2006 | Sample overlap: Australian Gulf veterans. | Cannot convert to OR |

| **Study info** |  |  |  |
| --- | --- | --- | --- |
| Author(s) | Pietrzak RH;Johnson DC;Goldstein MB;Malley JC;Rivers AJ;Morgan CA;Southwick SM; | Polusny MA;Kumpula MJ;Meis LA;Erbes CR;Arbisi PA;Murdoch M;Thuras P;Kehle-Forbes SM;Johnson AK; | Proctor, S. P., Harley, R., Wolfe, J., Heeren, T., & White, R. F. |
| Year | 2010 | 2014 | 2001 |
| **Participants** |  |  |  |
| Sample | OIF/OEF Veterans from Connecticut | Post-deployment mental health in National Guard troops deployed to Iraq and Afghanistan. | Fort Devens |
| Samplesize | N=272 | N= 801 | T: 141, C: 46 |
| Branch | Army, Marines, Air Force | Army, i.e. Brigade Combat Teams | NA |
| Rank (% Enlisted) | NA | 88.6 | NA |
| Active Duty Status (%) | 28 | 0 | NA |
| **Intervention** |  |  |  |
| Deployed from | USA | USA | USA |
| Deployed to | OIF;OEF | OIF;OEF | Gulf War |
| Type of deployment | War zone | War zone | War zone |
| **Contrast** |  |  |  |
| Military Comparison | Yes | Yes | Yes |
| Comparison | CE | CE (continuous) | DE |
| **Outcome** |  |  |  |
| Outcome | PTSD; Depression | PTSD | Depression (DSM-III-R, SCID), PTSD (CAPS) |
| Outcome type | Continuous (2) | Continuous | Continuous: Depression. Dichotomous: PTSD |
| Time point | Time between return from depolyment and survey completion was 26.9 mths (.7 sd), p189 | Data collected: 2-3 months post | Deployment: 1991. Data collected: 1994 – 1996 |
| Source | PTSD: PCL-M, Depression: PHQ-9 | PTSD (PCL-M) | Questionnaire |
| **Method** |  |  |  |
| Estimation | SEM | Multiple regression | None |
| **Effect size extraction** |  |  |  |
| Type of ES | Pearson correlations | Marginal effects | 0 |
| Notes | Cannot convert to OR | Standardized Regression Slopes | Sample overlap: The Fort Devens Operation Desert Storm Reunion Survey. Risk of Bias (5) |

| **Study info** |  |  |  |
| --- | --- | --- | --- |
| Author(s) | Proctor, S. P., Heaton, K. J., White, R. F., & Wolfe, J. | Proctor, S. P., Heeren, T., White, R. F., Wolfe, J., Borgos, M. S., Davis, J. D. et al. | Renshaw KD; |
| Year | 2001 | 1998 | 2011 |
| **Participants** |  |  |  |
| Sample | Fort Devens | Fort Devens | Utah National Guards |
| Samplesize | Unclear | C: 48; T1: 186; T2: 66 | N=207 |
| Branch | NA | Devens Cohort: Army | Army, Air Force. |
| Rank (% Enlisted) | NA | New Orleans Cohort: Army, Navy, Air Force, Marines | NA |
| Active Duty Status (%) | NA | NA | 0 |
| **Intervention** |  | 2.5 |  |
| Deployed from | USA |  | USA |
| Deployed to | Gulf War | USA | OIF;OEF |
| Type of deployment | War zone | Gulf War | War zone |
| **Contrast** |  | War zone |  |
| Military Comparison | Yes |  | Yes |
| Comparison | CE | Yes | CE |
| **Outcome** |  | DE |  |
| Outcome | PTSD (SCID, CAPS) | Depression |  |
| Outcome type | Dichotomous | Dichotomous | PTSD |
| Time point | Deployed: Aug. 2, 1990 - July 31, 1991. Data collected: 1994 - 1996 | Deployment: December 1990 - August 1991. Data collected: Devens: Spring 1994 - Fall 1996. NO: Summer 1994 - Fall 1995 Germany: Spring 1995 | Dichotomous |
| Source | Clinical interview | Questionnaire | Deployed: 2001 - 2008. Data collected: 2007-2008 |
| **Method** |  |  | PTSD: PCL-M |
| Estimation | Fisher's exact (unadjusted) | SUDAAN Logistic regression | SEM |
| **Effect size extraction** |  |  |  |
| Type of ES | 0 | 0 | 0 |
| Notes | Sample overlap: The Fort Devens Operation Desert Storm Reunion Survey. Risk of Bias (5) | Sample overlap: The Fort Devens Operation Desert Storm Reunion Survey. Risk of Bias (5) | Sample overlap: Utah National Guards. |

| **Study info** |  |  |  |
| --- | --- | --- | --- |
| Author(s) | Rona RJ;Jones M;Sundin J;Goodwin L;Hull L;Wessely S;Fear NT; | Rona, R. J., Fear, N. T., Hull, L., & Wessely, S. | Rona, R. J., Hooper, R., Jones, M., Hull, L., Browne, Horne Murphy, D., Hotopf, and Wessely |
| Year | 2012 | 2007 | 2006 |
| **Participants** |  |  |  |
| Sample | British Iraq War Study | Combine sample from the British Gulf War study and the British Iraq War Study (women only) | The British Iraq War Study |
| Samplesize | N=230 | Gulf war: T: 453, C: 192. Iraq war: T: 395, C: 638 | Time 1: C: 2.873, T: 4.304. Time 2: C: 1885, T: 2820 |
| Branch | Naval Services, Army, Royal Air Force | Na | Army, Navy, Air Force |
| Rank (% Enlisted) | 92.6 | 66.6 | 77.4 |
| Active Duty Status (%) | 90.9 | NA | NA |
| **Intervention** |  |  |  |
| Deployed from | UK | UK |  |
| Deployed to | OIF | Gulf War, OIF | OIF |
| Type of deployment | War zone | War zone | Combat operation |
| **Contrast** |  |  |  |
| Military Comparison | Yes | Yes | Yes |
| Comparison | A | A | A |
| **Outcome** |  |  |  |
| Outcome | PTSD severity | PTSD (PCL-C), Depression (GHQ), Alcohol abuse | PTSD, Alcohol abuse |
| Outcome type | Multinomial | Dichotomous | Dichotomous |
| Time point | Baseline: 16 months after. Followup: 3 years later | Gulf War Study: Deployed: 1991. Data collected: 1997. Iraq War Study: Deployed: 2003. Data collected: 2004 – 2006 | 1st: 2002 (prior to deployment); |
| Source | PTSD (PCL-C ) | Questionnaire | Questionnaire |
| **Method** |  |  |  |
| Estimation | Multinomial logistic regression | Logistic regression | None |
| **Effect size extraction** |  |  |  |
| Type of ES | 0 | 0 |  |
| Notes | Sample overlap: British Iraq War Study. | Sample overlap: British Gulf War Study and British Iraq War Study. | Sample overlap: British Iraq War Study. Risk of Bias (5) |

| **Study info** |  |  |  |
| --- | --- | --- | --- |
| Author(s) | Rona, R. J., Hooper, R., Jones, M., Iversen, A. C., Hull, L., Murphy, D. et al. | Sareen J;Belik SL;Afifi TO;Asmundson GJ;Cox BJ;Stein MB; | Schultz M;Glickman ME;Eisen S; |
| Year | 2009 | 2008 | 2014 |
| **Participants** |  |  |  |
| Sample | The British Iraq War Study | Canadian Community Health Survey Cycle 1.2 Canadian Force Supplement. | A national random sample of OIF/OEF military personnel, stratified by component of service . |
| Samplesize | C: 1003; T: 870 (from table 1, p14) | C: 5587; T:2846 | N= 512 |
| Branch | Army, Navy, Air Force | NA | Army, Navy, Air force, Marines |
| Rank (% Enlisted) |  | 79.7 | NA |
| Active Duty Status (%) | 77.5 | 66 | 39.6 |
| **Intervention** | NA |  |  |
| Deployed from |  | Canada | USA |
| Deployed to | UK | NA | OIF;OEF |
| Type of deployment | OIF | NA | War zone |
| **Contrast** | War zone |  |  |
| Military Comparison | Yes | Yes | Yes |
| Comparison | A | Unclear | CE |
| **Outcome** |  |  |  |
| Outcome | PTSD (PCL-C) | PTSD, Depression, Alcohol dependence | PTSD and alcohol, and MCS |
| Outcome type | Dichotomous | Dichotomous | Dichotomous |
| Time point | Baseline data collected: 2002 Exposure to Iraq war: 2003 Outcome data collected: June 2004 - March 2006 | Survey in 2002. Participants were deployed to the Gulf War, Rwanda, Somalia, Yugoslavia (0 – 11 y) | Time 1: 3-12 months post, Time 2: 6 months after time 1 |
| Source | Questionnaire | Questionnaire: PTSD | PTSD (PCL-M), Alcohol (AUDIT), Mental health (MCS). |
| **Method** |  |  |  |
| Estimation | Logistic regression | Logistic regression | Logistic regression with sampling and non-response weights |
| **Effect size extraction** |  |  |  |
| Type of ES | 0 | OR | Marginal effect (OR) |
| Notes | Sample overlap: British Iraq War Study. No ES extractable. | Unclear if all have been deployed or not or even if those exposed to combat have been so during deployment | No raw SD for outcome and exposure |

| **Study info** |  |  |  |
| --- | --- | --- | --- |
| Author(s) | Seelig AD;Jacobson IG;Smith B;Hooper TI;Gackstetter GD;Ryan MA;Wells TS;MacDermid WS;Smith TC; | Smith, T. C., Jacobson, I. G., Hooper, T. I., Leardmann, C. A., Boyko, E. J., Smith, B. et al. | Smith, T. C., Wingard, D. L., Ryan, M. A. K., Kritz-Silverstein, D., Slymen, D. J., & Sallis, J. F. |
| Year | 2012 Jul 15 | 2011 | 2009 |
| **Participants** |  |  |  |
| Sample | Millennium Cohort Study. | Millennium Cohort Study | Millennium Cohort Study |
| Samplesize | N = 17,481 | Not reported | T: 19841, C: 46747 |
| Branch | Army, Air Force, Navy /Coast Guard, Marine. | Army, Air force, Navy, Marine corps (table 1, p. 4) | Army, Navy, Air Force, Marines |
| Rank (% Enlisted) | 75.6 | 77 | 76.9 |
| Active Duty Status (%) | 50.6 | 57 | 56.9 |
| **Intervention** |  |  |  |
| Deployed from | USA | USA | USA |
| Deployed to | OIF;OEF | OIF;OEF | Gulf War |
| Type of deployment | War zone | War zone | War zone |
| **Contrast** |  |  |  |
| Military Comparison | Yes | Yes | Yes |
| Comparison | A; CE | A | A |
| **Outcome** |  |  |  |
| Outcome | PTSD, depression and anxiety | PTSD, depression and alcohol | PTSD (PCL-C) |
| Outcome type | Dichotomous | Percents only | Dichotomous |
| Time point | Deployed: 2001-2009 Baseline: 2001 - 2003. Followup: 2004 - 2006 | Varies, deployed between 2001-2009 and uses Panel 1 first follow-up but no dates reported | Deployed: 1998 – 2000. Data collected: 2001-2003 |
| Source | Questionnaire: PTSD (PCL-C) and (PHQ-9). | Questionnaire: PTSD Checklist–Civilian Version (PCL-C), Primary Care Evaluation of Mental Disorders Patient Health Questionnaire | Questionnaire |
| **Method** |  |  |  |
| Estimation | Logistic regression | None | Multinomial logistic regression with response weights |
| **Effect size extraction** |  |  |  |
| Type of ES | 0 | 0 | 0 |
| Notes | Sample overlap: Millenium Cohort Study. | Sample overlap: Millenium Cohort Study | Sample overlap: Millenium Cohort Study |

| **Study info** |  |  |  |
| --- | --- | --- | --- |
| Author(s) | Smith, T. C., Zamorski, M., Smith, B., Riddle, J. R., Leardmann, C. A., Wells, T. S. et al. | Soares HL; | Southwick, S. M., Morgan III, C. A. et al. |
| Year | 2007 | 2008 | 1995 |
| **Participants** |  |  |  |
| Sample | Millennium Cohort Study | Soldiers who completed at least one tour of duty in Operation Enduring Freedom, Operation Iraqi Freedom, or a combination of both. | Same sample as Southwick, S. M., Morgan, A. et al. (1993) |
| Samplesize | T: 52590, C: 22823 | N=69 | NA |
| Branch | Army, Navy, Air Force, Marines | Air Force, Army, Marines, Navy | NA |
| Rank (% Enlisted) | 77.2 | NA | NA |
| Active Duty Status (%) | 57 | NA | NA |
| **Intervention** |  |  |  |
| Deployed from | USA | USA | USA |
| Deployed to | Bosnia, Kosovo or Soutwest Ásia | OIF;OEF | Gulf War |
| Type of deployment |  | War zone | War zone |
| **Contrast** | War zone, Civil conflict |  |  |
| Military Comparison | Yes | Yes | Yes |
| Comparison | A | CE (y/n) |  |
| **Outcome** |  |  |  |
| Outcome | CMD (MCS) | PTSD, depression | PTSD |
| Outcome type | Continuous | Continuous | Continuous |
| Time point | Deployment: Jan. 1, 1998 – Sept. 1, 2000. Data collected: July 2001- June 2003 | Not reported but study from 2007 |  |
| Source | Questionnaire | PTSD(IES-R), Depression (BDI), |  |
| **Method** |  |  |  |
| Estimation | ANCOVA adjusted means | None | OLS |
| **Effect size extraction** | 0 |  |  |
| Type of ES | Mean | SMD | t-value |
| Notes | No SD available | N by Combat exposure not reported | No ES extractable. |

| **Study info** |  |  |  |
| --- | --- | --- | --- |
| Author(s) | Southwick, S. M., Morgan, A. et al. | Stuart, J. A. & Bliese, P. D. | Taft, C. T., Schumm, J. A., Panuzio, J., & Proctor, S. P. |
| Year | 1993 | 1998 | 2008 |
| **Participants** |  |  |  |
| Sample | Same sample as Southwick, S. M., Morgan III, C. A. et al. (1995) | Gulf, Army reservists who have returned to civilian life | The Fort Devens Operation Desert Storm Reunion Survey |
| Samplesize | NA | T: 991, C: 279 | T: 1.512, C: 1.437 |
| Branch | NA | NA | Army |
| Rank (% Enlisted) | NA | NA | 91.6 |
| Active Duty Status (%) | None | None | 21.5 |
| **Intervention** |  |  |  |
| Deployed from | USA | USA |  |
| Deployed to | Gulf War | Gulf War | ODS |
| Type of deployment | War zone | War zone | NA |
| **Contrast** |  |  |  |
| Military Comparison | Yes | Yes | Yes |
| Comparison | CE | A | CE |
| **Outcome** |  |  |  |
| Outcome |  | Depression (BSI) | PTSD (Mississippi scale) |
| Outcome type | Continuous | Continuous | Continuous |
| Time point | 2 year follow-up | Data collected: January 1993 - February 1993 | 5 days after return to Fort Devens, MA. (Time 1); 18-24 months after again (Time 2). Estimated models use Time 2 measurements |
| Source |  | Questionnaire | Questionnaire |
| **Method** |  |  |  |
| Estimation |  | Hierarchical linear regression | Structural Equation Modeling |
| **Effect size extraction** |  |  |  |
| Type of ES | t-value | 0 |  |
| Notes | No ES extractable. | No ES extractable. | Sample overlap: The Fort Devens Operation Desert Storm Reunion Survey. Risk of Bias (5) |

| **Study info** |  |  |  |
| --- | --- | --- | --- |
| Author(s) | Tanielian & Jaycox | Thomas JL;Britt TW;Odle-Dusseau H;Bliese PD; | Vasterling, J. J., Proctor, S. P., Friedman, M. J., Hoge, C. W., Heeren, T., King, L. A. et al. |
| Year | 2008 | 2011 | 2010 |
| **Participants** |  |  |  |
| Sample |  | Soldiers from active-duty brigade unit | Neurocognition Deployment Health Study |
| Samplesize | N=1938 | N=2439 | T: 774, C: Not reported |
| Branch | Army, Navy, Air Force, Marines Corps. | Army | Army |
| Rank (% Enlisted) | 85.9 | 51 | 72 |
| Active Duty Status (%) | 38.3 | NA | 86.6 |
| **Intervention** |  |  |  |
| Deployed from | USA | USA | USA |
| Deployed to | OIF;OEF | OIF;OEF | OIF |
| Type of deployment | War zone | War zone | War zone |
| **Contrast** |  |  |  |
| Military Comparison | Yes | Yes | Yes |
| Comparison | CE | CE | CE |
| **Outcome** |  |  |  |
| Outcome | PTSD, depression | PTSD score, Depression score | PTSD (PCL-C) |
| Outcome type | Dichotomous | Continuous (2) | Continuous |
| Time point | Not reported | Measured 3 days after return from deployment (p869) | Deployment: April 2003 - Sept 2006 |
| Source | Questionnaire: PTSD (PCL-M), Depression (PHQ-8), | Questionnaire. PTSD: (PCL), Depression: (PHQ-9) | Questionnaire |
| **Method** |  |  |  |
| Estimation | Weighted and adjusted relative risk ratio (probably a log-binomial model as they state they use the SAS proc genmod) | 3 level random intercept mixed effect model | OLS on individual change. In addition they consider ICC among units from same battalion (and finds no significant ICC). |
| **Effect size extraction** |  |  |  |
| Type of ES | Marginal Effect (RR) | Marginal effects | 0 |
| Notes | Cannot calculate relevant ES | Cannot calculate relevant ES | Sample overlap: Neurocognition Deployment Health Study. Risk of Bias (5) |

| **Study info** |  |  |  |
| --- | --- | --- | --- |
| Author(s) | Vogt D;Vaughn R;Glickman ME;Schultz M;Drainoni ML;Elwy R;Eisen S; | Vogt, D. S. & Tanner, L. R. | Vogt, D. S., Pless, A. P., King, L. A., & King, D. W. |
| Year | 2011 | 2007 | 2005 |
| **Participants** |  |  |  |
| Sample | A national stratified random sample of 2,000 OEF/OIF military personnel was randomly selected from the DMDC roster | Same sample as 7788 |  |
| Samplesize | Total 595 | N=308 | N=317. |
| Branch | Army, Navy, Air Force, Marines | Army, Navy, Air Force, Marines | Army, Navy, Air Force, Marines, Coastal Guard |
| Rank (% Enlisted) | NA | NA | NA |
| Active Duty Status (%) | 41.6 | 26 |  |
| **Intervention** |  |  | NA |
| Deployed from | USA | USA | USA |
| Deployed to | OIF;OEF | Gulf War | Gulf War |
| Type of deployment | War zone | War zone | War zone |
| **Contrast** |  |  |  |
| Military Comparison | Yes | Yes | Yes |
| Comparison | CE | CE | CE |
| **Outcome** |  |  |  |
| Outcome | PTSD, depression and substance abuse | PTSD (PCL) | PTSD, depression |
| Outcome type | Continuous | Continuous | Continuous |
| Time point | Within a year | Unclear | NR |
| Source | PTSD (PCL-M), depression and substance abuse: (BASIS-24) subscales | Questionnaire | Questionnaire: PTSD (PCL-M), depression (BDI) |
| **Method** |  |  |  |
| Estimation | Uses sample design weights adjusted to also account for non-response. Multiple regression | Mix of measurement and structurel model | Hierarchical regression |
| **Effect size extraction** |  |  |  |
| Type of ES | Partial Correlation | 0 | Marginal effects (mean) |
| Notes | Cannot convert to OR | Overlapping sample Vogt et al., 2005 (#7785). Risk of Bias (5) | Cannoy calculate relevant ES |

| **Study info** |  |  |  |
| --- | --- | --- | --- |
| Author(s) | Waller M;Treloar SA;Sim MR;McFarlane AC;McGuire AC;Bleier J;Dobson AJ; | Ward, W. | White, R. F., Proctor, S. P., Heeren, T., Wolfe, J., Krengel, M., Vasterling, J. et al. |
| Year | 2012 | 1997 | 2001 |
| **Participants** |  |  |  |
| Sample | Australian Defence Force deployment to East Timor and Bougainville | 0 | Fort Devens |
| Samplesize | - | T: 117, C: 77 | Gulf: 193, Germany: 47 |
| Branch | Navy, Army, RAAF | NA | Devens Cohort: Army |
| Rank (% Enlisted) | 69.5 | 97 | New Orleans Cohort: Army, Navy, Ari Force, Marines. |
| Active Duty Status (%) | NA | NA | NA |
| **Intervention** |  |  | 12 |
| Deployed from | Australia | Australia |  |
| Deployed to | Bougainville;East Timor | Somalia | USA |
| Type of deployment | NA | Civil war | Gulf War |
| **Contrast** |  |  | War zone |
| Military Comparison | Yes | Yes |  |
| Comparison | CE (1, 2-3, 4 stressors) | Unclear | Yes |
| **Outcome** |  |  | DE |
| Outcome | PTSD; Psychological Distress | Depression (GHQ-28) | Depression (POMS) |
| Outcome type | Dichotomous (2) | Continuous | Continuous |
| Time point | Data collected: 2008, ~8 years after | 15 months after return | Deployment: December 1990 - August 1991. Data collected: Devens: Spring 1994 - Fall 1996. NO: Summer 1994 - Fall 1995 Germany: Spring 1995 |
| Source | PTSD (PCL-C), Psychological Distress (K10) | Questionnaire | Questionnaire |
| **Method** |  |  |  |
| Estimation | Logistic regression | None | SUDAAN Adjusted means |
| **Effect size extraction** |  |  |  |
| Type of ES | 0 | 0 | 0 |
| Notes | Both treatment and control deployed. Traumatic stressors do not uniformly favour one deployment over the other (Table 3). | No SD available. Cannot extract ES. | Sample overlap: The Fort Devens Operation Desert Storm Reunion Survey. Risk of Bias (5) |

| **Study info** |  |  |  |
| --- | --- | --- | --- |
| Author(s) | Wolfe J;Proctor SP;Davis JD;Borgos MS;Friedman MJ; | Wolfe, J., Brown, P. J., & Kelley, J. M. | Wolfe, J., Erickson, D. J., Sharkansky, E.J., King, D.W. and King, L.A. |
| Year | 1998 | 1993 | 1999 |
| **Participants** |  |  |  |
| Sample | The Fort Devens Operation Desert Storm Reunion Survey | The Fort Devens Operation Desert Storm Reunion Survey | The Fort Devens Operation Desert Storm Reunion Survey |
| Samplesize | N=2,119 | N= 2344 | N=2313 |
| Branch | Army | NA | Army |
| Rank (% Enlisted) | NA | 93.5 | NA |
| Active Duty Status (%) | 20.4 | NA | 21 |
| **Intervention** |  |  |  |
| Deployed from | USA | USA | USA |
| Deployed to | Gulf war | Gulf War | Gulf War |
| Type of deployment | War zone | War zone | War zone |
| **Contrast** |  |  |  |
| Military Comparison | Yes | Yes | Yes |
| Comparison | CE | CE (continuous) | CE |
| **Outcome** |  |  |  |
| Outcome | PTSD | PTSD (DSM-3), Depression (BSI) | PTSD (Mississippi) |
| Outcome type | Dichotomous | Continuous | Continuous |
| Time point | Time 1: Within 5 days of their return, Time 2: 18-24 months following the initial survey. | Data collected: April 1991 – July 1991 | Time 1: Within 5 days of their return, Time 2 18-24 months following the initial survey |
| Source | Questionnaire: PTSD: the Mississippi Scale for Combat-Related PTSD | Questionnaire | Questionnaire |
| **Method** |  |  |  |
| Estimation | None | Multiple regression | Bivariate correlation |
| **Effect size extraction** |  |  |  |
| Type of ES | 0 | Marginal effects (Mean) | 0 |
| Notes | Sample overlap: The Fort Devens Operation Desert Storm Reunion Survey. Risk of Bias (5). | Combat exposure (Laufer scale). Raw SD recoverable. | Sample overlap: The Fort Devens Operation Desert Storm Reunion Survey. Risk of Bias (5) |

| **Study info** |  |  |  |
| --- | --- | --- | --- |
| Author(s) | Wood MD;Foran HM;Britt TW;Wright KM; | Woodhead C;Wessely S;Jones N;Fear NT;Hatch SL; | Wooten NR; |
| Year | 2012 | 2012 | 2012 |
| **Participants** |  |  |  |
| Sample | - | TELIC and HERRICK subsamples | Army National Guard Women (OEF/OIF) |
| Samplesize | 552 | males = 4554; females = 432 | N=91 |
| Branch | Cavalry | Naval Services, Army, | Army |
| Rank (% Enlisted) | 100 | Royal Air Force | 73 |
| Active Duty Status (%) | 100 | 80.4 | 0 |
| **Intervention** |  | 93 |  |
| Deployed from | USA | UK | USA |
| Deployed to | OIF | OIF;OEF | OIF;OEF |
| Type of deployment | War zone | War zone | War zone |
| **Contrast** |  |  |  |
| Military Comparison | Yes | Yes | Yes |
| Comparison | CE | CE (H vs L) | CE |
| **Outcome** |  |  |  |
| Outcome | PTSD | PTSD; CDM; Hazardous Drinking | PTSS |
| Outcome type | Continuous | Continuous (1), dichotomous (2) | Dichotomous |
| Time point | Deployed: 2007-2008. Data collection: 4 months following a 15-month | Data collected: Nov, 2007 – Sept, 2009. HERRICK deployed: April 2006 - April 2007. TELIC deployed: Jan - April 2003 | Data collected: Jan-March 2009. |
| Source | Questionnaire | PTSD: PCL-C; CMD: GHQ-12; Alcohol: AUDIT | PTSD(PCl-M) |
| **Method** |  |  |  |
| Estimation | Linear regression | PTSD: negative binomial regression; CMD, Alco: logistic regression | OLS |
| **Effect size extraction** |  |  |  |
| Type of ES | Pearson correlation | RR, OR | Correlation |
| Notes | Cannot convert to OR | CE division cannot be used and overlap to British Iraq War sample | Cannot convert to OR |

| **Study info** |  |  |
| --- | --- | --- |
| Author(s) | Wooten NR; | Wright KM;Cabrera OA;Adler AB;Bliese PD; |
| Year | 2010 | 2013 |
| **Participants** |  |  |
| Sample | Army National Guard Women | Brigade Combat Team returning from Iraq |
| Samplesize | N=87-88 (tables 6,7, 8) | N = 1233 |
| Branch | Army | Army |
| Rank (% Enlisted) | 72 | 94.3 |
| Active Duty Status (%) | 0 | NA |
| **Intervention** |  |  |
| Deployed from | USA | USA |
| Deployed to | OIF;OEF | OIF |
| Type of deployment | War zone | War zone |
| **Contrast** |  |  |
| Military Comparison | Yes | Yes |
| Comparison | CE | CE |
| **Outcome** |  |  |
| Outcome | PTSS, Depression, Alcohol Misuse | PTSD, depression |
| Outcome type | Dichotomous (3) | Continuous |
| Time point | Data collected: 2009 | Time 1: few days post, Time 2: 4 months post, Time 3 12 months post |
| Source | PTSS (PCL-M), Depression (PHQ-9), Alchohol (TICS). | PTSD (PCL-S), depression(PHQ-D) |
| **Method** |  |  |
| Estimation | OLS, Logistic regression | Growth model (which we do not use) and correlation coefficients |
| **Effect size extraction** |  |  |
| Type of ES | Marginal effect (mean) | Marginal effects (Means) |
| Notes | No SD reported for exposure or outcome variables. Cannot extract ES. | Marginal effect of combat exposure at time 3 |

## Characteristics of studies from updated search

| **Author** | **Adams, Nikitin , Wooten, Larson** | **Afari ,Pittman , Floto , Owen, Hossain et al.** | **Balderrama-Durbin, Stanton, Snyder, Cigrang , Talcott et al** |
| --- | --- | --- | --- |
| Year | 2016 | 2015 | 2017 |
| **Participants** |  |  |  |
| Sample | Psychological Injury Combat Study (SUPIC), outcome data is from PDHA. Army enlisted women returning from OEF/OIF 2008-2011 | A subset of OIF and OEF veterans who enrolled for Veterans Affairs health care in San Diego between May 1, 2009 and December 3, 2010 | A subset (those with partners) of active-duty service members from a larger longitudinal investigation of U.S. Air Force Security Forces. |
| Samplesize | 42,397 | 554 | 76 |
| Branch | Army | NR | Air Force |
| Rank (% Enlisted) | 100% | NR | NR |
| Active Duty Status (%) | 66% | NR | 100% |
| **Intervention** |  |  |  |
| Deployed from | USA | USA | USA |
| Deployed to | Iraq and Afghanistan (OIF and OEF) | Iraq and Afghanistan (OIF and OEF) | Iraq |
| Type of deployment | War zone | War zone | War zone |
| **Contrast** |  |  |  |
| Comparison | CE (0 vs 1 exposure, vs 2 and 3+ exposures also available). | CE (with/without) | CE (continuous) |
| **Outcome** |  |  |  |
| Outcome | PTSD, Depression, at-risk drinking | PTSD, Depression, Alcohol consumption | PTSD, Depression, Alcohol consumption |
| **Outcome type** | Dichotomous | Continuous | Continuous |
| **Time point** | Within 60 days of the end of an index deployment | NR | 6-9 months post deployment |
| **Source** | Primary Care-PTSD, PHQ-2, AUDIT-C | PCL-C, PHQ-9, AUDIT | PCL-M, PHQ, AUDIT |
| **Method** |  |  |  |
| **Estimation** | Logistic regression | Gender divided means | Correlations |
| **Effect size extraction** |  |  |  |
| **Type of ES** | OR | Means | Correlations |
| **Notes** | Used in main analysis | 5 in confounding | Also used in Fissette, 2016. 5 in confounding. Cannot extract ES with SE |

| **Author** | **Boasso, Steenkamp, Nash, Larson , Litz** | **Boulos, Zamorski** | **Britt, Herleman , Odle-Dusseau, Moore,Castro , Hoge** |
| --- | --- | --- | --- |
| **Year** | 2015 | 2016 | 2016 |
| **Participants** |  |  |  |
| Sample | The Marine Resiliency Study (MRS; Baker et al., 2012), a 4-wave longitudinal study of four Iraq- or Afghanistan-deployed cohorts of male infantry Marines. | The 2013 Canadian Forces Mental Health Survey. Use only Regular Force (RegF) personnel who were serving in September 2012 | Same data as Hoge et al., 2004 |
| Samplesize | 617 | 6696 (3384 Afghanistan deployed) |  |
| Branch | Marines | Army: 65%, Navy: 14% , Air Force: 22% |  |
| Rank (% Enlisted) | 100% | 80% |  |
| Active Duty Status (%) | 100% | 100% |  |
| **Intervention** |  |  |  |
| Deployed from | USA | Canada |  |
| Deployed to | Iraq and Afghanistan (OIF and OEF) | Afghanistan |  |
| Type of deployment | War zone | War zone |  |
| **Contrast** |  | 1 |  |
| Comparison | CE (tertiles) | DE |  |
| **Outcome** |  |  |  |
| Outcome | PTSD | PTSD, Depression, Alcohol (abuse and dependece) |  |
| **Outcome type** | NR | Dichotomous |  |
| **Time point** | NR | Mean 5.4 years |  |
| **Source** | CAPS and PCL-S | WHO-CIDI |  |
| **Method** |  |  |  |
| **Estimation** | None | Logistic regression |  |
| **Effect size extraction** |  |  |  |
| **Type of ES** | None | OR |  |
| **Notes** | 5 in confounding. Do not report results that can be used for analysis (They employed second-order GMM (SOGMM; Grimm & Ram, 2009) to test whether distinct trajectories of posttraumatic stress symptoms were present in the tertiles (of combat exposure)) | 5 in confounding | 5 in confounding and data already used (Same data as Hoge et al 2004) |

| **Author** | Crum-Cianflone , Powell , LeardMann , Russell, Boyko | de Silva, Varuni ,Jayasekera , Hanwella | Dursa, Barth, Schneiderman, Bossarte |
| --- | --- | --- | --- |
| **Year** | 2016 | 2016 | 2016 |
| **Participants** |  |  |  |
| Sample | Millennium Cohort Study | Navy personnel from Sri Lanka | National Health of Gulf War Era Veterans and Their Families. Same data as Coughlin, Kang & Mahan, 2011 |
| Samplesize | 10,671 (7,292 non-deployed) | 259 Special Forces and 412 regular navy personnel | T: 8,104 C: 6,148 |
| Branch | Army: 62%, Navy: 11% , Air Force: 21%, Marines: 6% | Navy | Air Force= 12,2 % Army= 64,4 % Marine Corps= 10,5 % Navy= 12,9 % |
| Rank (% Enlisted) | NR | NR | Enlisted=84,3 % Officer=14,3 % Warrant officer=1,4 % |
| Active Duty Status (%) | 43% | 100% | Active= 35,7 % National Guard=29 % Reserve=35,3 % |
| **Intervention** |  |  |  |
| Deployed from | USA | Sri Lanka | USA |
| Deployed to | OIF and OEF | NR | Gulf |
| Type of deployment | War zone | War zone | War zone |
| **Contrast** |  |  |  |
| Comparison | Non-deployed | CE (10 different separately) | Non-deployed |
| **Outcome** |  |  |  |
| Outcome | PTSD, Depression, Alcohol | Cannabis use | PTSD and depression |
| **Outcome type** | Dichotomous | Dichotomous | Dichotomous |
| **Time point** | NR | 3 months | More than 20 years |
| **Source** | PCL-C, PHQ, questionnaire | AUDIT | PCL-C, PHQ-9 |
| **Method** |  |  |  |
| **Estimation** | Report insidence rates weighted for age and sex using U.S. military population proportions in October 2003. Separate by non-deployers, deployed with combat, deployed without combat | Logistic regression | Logistic regression |
| **Effect size extraction** |  |  |  |
| **Type of ES** | OR | OR | OR |
| **Notes** | Data sample already used in review (Millenium) | 5 in confounding | Data sample already used in review (National Health of Gulf War Era Veterans and Their Families) |

| **Author** | **Fink,Cohen , Sampson , Gifford , Fullerton et al.** | **Fissette** | **Hougsnæs , Bøe , Dahl , Reichelt** |
| --- | --- | --- | --- |
| **Year** | 2016 | 2016 | 2017 |
| **Participants** |  |  |  |
| Sample | US Reserve and National Guard service members. Called the Reserve National Guard (RNG) study | Subset of Active-duty sample of United States Air Force (USAF) Security Forces Airmen. Trained Iraqi Police Transition Team ,during 2009 and 2010. Also used in Balderrama-Durbin et al., 2017 | Norwegian soldiers at a mean of 4 years following deployment to Afghanistan in 2001–2011 |
| Samplesize | 2003 | 164 |  |
| Branch | NR | Air Force |  |
| Rank (% Enlisted) | NR | NR |  |
| Active Duty Status (%) | 0 | 100% |  |
| **Intervention** |  |  |  |
| Deployed from | USA |  | Norway |
| Deployed to | NR | Iraq | Afghanistan |
| Type of deployment | NR | War zone | War zone |
| **Contrast** |  |  |  |
| Comparison | Non-deployed | CE (yes or no) | CE (yes or no) |
| **Outcome** |  |  |  |
| Outcome | PTSD and depression | Alcohol | PTSD, Depression, Alcohol |
| **Outcome type** | Dichotomous | Dichotomous | Dichotomous |
| **Time point** | NR | 6-9 months | Mean of 4 years |
| **Source** | PCL-C, PHQ | AUDIT | PCL-M-17, HADS-D, AUDIT |
| **Method** |  |  |  |
| **Estimation** | Weighted incidence rates | Linear regresion, logistic regression | Logistic regression |
| **Effect size extraction** |  |  |  |
| **Type of ES** | Weighted incidence rates of post-traumatic stress disorder (PTSD) (and depression) per 100 person-years (also report CI), by sensitive and specific criterion, | OR | OR |
| **Notes** | Report Weighted incidence rates of post-traumatic stress disorder (PTSD) (and depression) per 100 person-years (also report CI), by sensitive and specific criterion. Cannot be used in meta analysis | 5 in confounding. Data also used in Balderrama-Durbin et al., 2017 | 5 in confounding |

| **Author** | **Hourani, Williams , Bray , Wilk ,Hoge** | **Jacobson; Donoho; Crum-Cianflone** | **Lee, Garber** |
| --- | --- | --- | --- |
| **Year** | 2016 | 2015 | 2015 |
| **Participants** |  |  |  |
| Sample | Two large, population-based Army samples obtained as part of the 2005 and 2008 U.S. Department of Defense Surveys of Health Related Behaviors among Active Duty Military Personnel. The 2008 data also used in Bray et al., 2010 | Millennium Cohort Study | The 2013 Canadian Forces Mental Health Survey. Use only Regular Force (RegF) personnel who were serving in September 2012 |
| Samplesize | A total of 3,639 in 2005 and 5,927 in 2008 | 4684 | 3025 |
| Branch | Army | NR | Army: 65%, Navy: 14% , Air Force: 22% |
| Rank (% Enlisted) | NR | NR | 80% |
| Active Duty Status (%) | 100% | NR | 100% |
| **Intervention** |  |  |  |
| Deployed from | USA | USA | Canada |
| Deployed to | OIF and oEF | OIF and oEF | Afghanistan |
| Type of deployment | War zone | War zone | War zone |
| **Contrast** |  |  |  |
| Comparison | Non-deployed and CE (high, moderate) | Combat (yes or no) | CE (tertiles) |
| **Outcome** |  |  |  |
| Outcome | PTSD | PTSD | PTSD |
| **Outcome type** | Dichotomous | Dichotomous | Dichotomous |
| **Time point** | NR | NR | Mean 4.6 years |
| **Source** | PCL-C | PCL-C | WHO-CIDI |
| **Method** |  |  |  |
| **Estimation** | Logistic regression | Propensity score adjustment using matched sample | Regression |
| **Effect size extraction** |  |  |  |
| **Type of ES** | OR | Relative risk | incidence rate ratio |
| **Notes** | 5 in confounding. The 2008 data also used in Bray et al., 2010 | Data sample already used in review (Millenium) | 5 in confounding. Same data as Boulos & Zamorski, 2016. |

| **Author** | **Ikin, McKenzie, Gwini, Kelsall, Creamer et al.** | **Kanesarajah, Waller, Zheng, Dobson** | **Telch, Beevers, Rosenfield, Lee, Reijntjes, Ferrell** |
| --- | --- | --- | --- |
| **Year** | 2016 | 2016 | 2015 |
| **Participants** |  |  |  |
| Sample | Same sample as 928, 1104, 1122, 1123 and 1159 (used) but a new follow up In 2011–2012, about 20 years after the Gulf War, depression only | Australian military personnel deployed to Iraq or Afghanistan between 2001 and 2009. | 133 participants from eight combat and two combat support units at Fort Hood Texas. |
| Samplesize | T: 715, C: 675 | 11 411 | 133 |
| Branch | Army, Navy, Air Force | NA | Army |
| Rank (% Enlisted) | 81% | NA | NA |
| Active Duty Status (%) | NA | NA |  |
| **Intervention** |  |  |  |
| Deployed from | Australia | Australia |  |
| Deployed to | Gulf | Iraq and Afghanistan (OIF and OEF) | Iraq |
| Type of deployment | War zone | War zone | War zone |
| **Contrast** |  |  |  |
| Comparison | Non-deployed | CE | CE |
| **Outcome** |  |  |  |
| Outcome | Depression | PTSD, alcohol | PTSD, Depression, Alcohol |
| **Outcome type** | Dichotomous | Dichotomous | Continuous |
| **Time point** |  | Median time 3 years | Depression while deployed and PTSD varies (0-at least 16 months) |
| **Source** | PHQ-9 | PCL-C, AUDIT | PCL-Short, 10-item version of the Center for Epidemiologic Studies Depression Scale (CES-D) |
| **Method** |  |  |  |
| **Estimation** | Logistic regression | Logistic regression | Multilevel, mixed-effects random coefficient regression models. Include CE level and change |
| **Effect size extraction** |  |  |  |
| **Type of ES** | OR | OR | Regression coefficient |
| **Notes** | 5 in confounding. Data sample already used (Ikin et al, 2004) | 5 in confounding. Cannot extract relevant ES (only marginal efffect) | 5 in confounding and cannot use Es as analysis includes level and change of CE |

| **Author** | **Mustillo, Kysar-Moon, Douglas, Hargraves, MacDermid et al.** | **Nyaronga, Toma** | **Ogle, Young** |
| --- | --- | --- | --- |
| **Year** | 2015 | 2015 | 2016 |
| **Participants** |  |  |  |
| Sample | Data from the Defense Medical Surveillance System, The Armed Forces Health Surveillance Center, U.S. Department of Defense, Silver Spring, Maryland (January 2008 to March 2009; The sample was then further limited to active duty service members who served in Iraq or Afghanistan only. Data already used, although Marines and Navy only (Macera, Aralis, Highfill-McRoy & Rauh, 2014) and in Adams, Nikitin , Wooten, Larson, 2015, although Army women only. | Service members/veterans actively enrolled with SUNY Empire State College in 2011 | Air Force Special Tactics sample, use Active duty only |
| Samplesize | 41,351 | 144 | 55 |
| Branch | Army, Navy, Marine and Air Force | Army, Navy, Marine, Coast Guard and Air Force | Air Force |
| Rank (% Enlisted) | NA | NR | NR |
| Active Duty Status (%) | 100% | NR | 100% |
| **Intervention** |  |  |  |
| Deployed from | USA | USA | USA |
| Deployed to | Iraq and Afghanistan (OIF and OEF) | NR | Iraq, Afghanistan and other |
| Type of deployment | War zone | War zone | War zone |
| **Contrast** |  |  |  |
| Comparison | CE (traumatic yes or no) | NR | CE (and some not deployed) |
| **Outcome** |  |  |  |
| Outcome | PTSD, Depression, Alcohol | PTSD | PTSD, Depression |
| **Outcome type** | Dichotomous | Continuous | Continuous |
| **Time point** | Less than 180 days after return | NR | NR |
| **Source** | Primary Care 4-item post-traumatic stress disorder screen (PC-PTSD) and depression self-reported, Risk for alcohol issues was assessed with a modified Two-Item Conjoint screen. | Mississippi Short Form | PCL-M and Depression Anxiety Stress Scale 21 (DASS-21) |
| **Method** |  |  |  |
| **Estimation** | Logistic regression | Regression | Correlation |
| **Effect size extraction** |  |  |  |
| **Type of ES** | OR | Regression coefficient | Correlation |
| **Notes** | 5 in confounding. Data already used, although Marines and Navy only (Macera, Aralis, Highfill-McRoy & Rauh, 2014) and in Adams, Nikitin , Wooten, Larson, 2016, although Army women only | 5 in confounding. Cannot be used in meta-analysis as it is not reported if control is deployed or not | 5 in confounding. Cannot extract ES and SE |

| **Author** | **Quartana, Wilk ,Balkin , Hoge** | **Trautmann, Goodwin, Höfler, Jacobi, Strehle et al.** | **Zheng, Kanesarajah,Waller, McGuire, Treloar** |
| --- | --- | --- | --- |
| **Year** | 2015 | 2017 | 2016 |
| **Participants** |  |  |  |
| Sample | Land Combat Study (LCS). OIF in 2003–2004, 3 months post | German deployed soldiers | Same data as Waller et al., 2012. Data were collected in 2008 from 3,564 Australian military veterans who deployed to East Timor or Bougainville |
| Samplesize | 587 | T: 1439, C: 779 | 3,564 |
| Branch | Army | Army | Bougainville Navy= 22% Army= 73 % RAAF= 5 % ; East Timor Navy= 13% Army= 77 % RAAF= 10 %  (table 2, p. 5) |
| Rank (% Enlisted) | NR | 36% | 70% |
| Active Duty Status (%) | 100% | 100% | NR |
| **Intervention** |  |  |  |
| Deployed from | USA | Germany | Australia |
| Deployed to | OIF and OEF | OEF | Bougainville and East timor. |
| Type of deployment | War zone | War zone | NR |
| **Contrast** |  |  |  |
| Comparison | CE | Non-deployed | CE |
| **Outcome** |  |  |  |
| Outcome | PTSD, Depression | PTSD, depression and alcohol abuse | PTSD, Alcohol |
| **Outcome type** | Continuous | Dichotomous | Dichotomous |
| **Time point** | 3 months | 12 months | NR |
| **Source** | The 17-item National Center for PTSD Checklist — (PCL), PHQ-9 | The Munich-Composite International Diagnostic Interview (DIA-X/M-CIDI) | PCL-C, AUDIT |
| **Method** |  |  |  |
| **Estimation** | Regression | Weighted percents | Logistic regression but with CE as a continuous variable |
| **Effect size extraction** |  |  |  |
| **Type of ES** | Regression coefficient | Weighted percents (with CIs) separately by deployed and non-deployed. | OR |
| **Notes** | 5 in confounding. Cannot extract relevant ES and SE (only marginal) | 5 in confounding. Data already used (Trautman et al., 2014) | Cannot extract relevant ES (only marginal and with interaction terms). Data already used (Waller et al., 2012) |
