# Supplementary material for: Deployment of personnel to military operations: impact on mental health and social functioning
Source: Campbell Syst Rev. 2018 Jun 1;14(1):1–127. doi: 10.4073/csr.2018.6 (PMC8427986; doi:10.4073/csr.2018.6)
Supplement: Supplementary file 3 — Supplementary material [file CL2-14--s003.docx]

# Online Supplement 3: Search Histories

## Academic Search Premier, Last Search: January 2015

| Search number | Term | Totals |
| --- | --- | --- |
| S1 | DE "RANDOMIZED controlled trials" | 35,533 |
| S2 | DE "RANDOMIZATION (Statistics)" | 400 |
| S3 | AB randomized | 131,838 |
| S4 | AB placebo | 59,215 |
| S5 | AB randomly | 104,231 |
| S6 | AB trial | 336,750 |
| S7 | AB groups | 1,565,493 |
| S8 | S1 OR S2 OR S3 OR S4 OR S5 OR S6 OR S7 | 1,921,733 |
| S9 | Review | 4,101,093 |
| S10 | S8 AND S9 | 146,063 |
| S11 | DE "COHORT analysis" | 19,387 |
| S12 | DE "CROSS-sectional method" | 31,661 |
| S13 | TI (case control) or AB (case control) | 51,305 |
| S14 | TI cohort OR AB cohort | 142,365 |
| S15 | TI ( cross sectional or cross-sectional or point in time or point-in-time) or AB (cross sectional or cross-sectional or point in time or point-in-time) | 150,953 |
| S16 | TI (epidemiologic N2 study) or AB (epidemiologic N2 study) | 6,701 |
| S17 | Ti ((follow up or followup) N2 study )OR AB ((follow up or followup) N2 study) | 17,316 |
| S18 | TI longitudinal or AB longitudinal | 105,706 |
| S19 | TI observational OR AB observational | 54,091 |
| S20 | TI (prospective n2 study) or AB (prospective n2 study) | 81,824 |
| S21 | TI retrospective OR AB retrospective | 112,204 |
| S22 | TI Intervention* N1 Stud* OR AB Intervention* N1 Stud* | 13,224 |
| S23 | Ti (quasi-experiment* or quasiexperiment* or experiment*) OR AB (quasi-experiment* or quasiexperiment* or experiment*) | 1,341,814 |
| S24 | TI assign* N3 (subject* or patient* or client*) or AB assign* N3 (subject* or patient* or client*) | 10,894 |
| S25 | TI (Propensity score* or heckman or difference in difference or regression discontinuity or (match* N1 control*) or (match* N1 compar* ) or assessment only or comparison samp* or propensity match*) OR AB (Propensity score* or heckman or difference in difference or regression discontinuity or (match* N1 control*) or (match* N1 compar* ) or assessment only or comparison samp* or propensity match*) | 1,050,979 |
| S26 | TI Non-random* or nonradom* or (non N1 random*) OR AB Non-random* or nonradom* or (non N1 random*) | 7,969 |
| S27 | TI ((random* N2 trial*) or RCT) OR AB ( (random* N2 trial*) or RCT) | 95,428 |
| S28 | (DE "Military Personnel" OR DE "Air Force Personnel" OR DE "Army Personnel" OR DE "Commissioned Officers" OR DE "Enlisted Military Personnel" OR DE "Marine Personnel" OR DE "Navy Personnel" OR DE "Volunteer Military Personnel") | 16,177 |
| S29 | TI ( (Soldier* or Marine* or airm#n* or Veteran* or militarian* or officer*) ) OR AB ( (Soldier* or Marine* or airm#n* or Veteran* or militarian* or officer*) ) | 338,912 |
| S30 | TI ( Army or troop* or military* or navy) OR AB ( Army or troop* or military* or navy) | 277,305 |
| S31 | TI ((armed or naval or navy or air or defen#e* or national) N1 force* ) OR AB ( (armed or naval or navy or air or defen#e* or national) N1 force1 ) | 5,863 |
| S32 | DE "War" | 22,767 |
| S33 | TI (war or wars or warfare*) OR AB (war or wars or warfare*) | 349,563 |
| S34 | TI (combat* or deploy* or post-deploy* or postdeploy*) OR AB (combat* or deploy* or post-deploy* or postdeploy*) | 110,761 |
| S35 | TI ((peace-keep* or peacekeep* or peace keep* or military) N1 mission*) OR AB ((peace-keep* or peacekeep* or peace keep* or military) N1 mission*) | 2,035 |
| S36 | TI (active N1 duty) OR AB (active N1 duty) | 2,580 |
| S37 | S32 OR S33 OR S34 OR S35 OR S36 | 457,404 |
| S38 | DE "VETERANS" | 4,295 |
| S39 | S28 OR S29 OR S30 OR S31 OR S38 | 583,910 |
| S40 | S37 AND S39 | 100,584 |
| S41 | (S11 OR S12 OR S13 OR S14 OR S15 OR S16 OR S17 OR S18 OR S19 OR S20 OR S21 OR S22 OR S23 OR S25 OR S26 OR S27) | 2,814,848 |
| S42 | S10 OR S41 | 2,911,057 |
| S43 | S40 AND S42 | **4,569** |

## Bibliotek.dk. Last search: January 2015

| Search number | Term | Totals |
| --- | --- | --- |
| S1 | ”væbnede styrker” eller tropper eller militær eller ”militære styrker” eller flåden eller marinen eller søværnet eller luftvåben eller flyvevåben eller luftstyrke ikke ma=(th eller nh) ikke ma=lb ikke ma=lm | 11798 |
| S2 | em=mandskab ikke ma=(th eller nh) ikke ma=lb ikke ma=lm | 20 |
| S3 | soldat eller marinesoldat? Eller militærtjeneste eller tjeneste eller (militært og personale) eller ”militære tropper” eller ”søværns personel” eller ”marine soldater” eller ”luftværns  personel” ikke ma=(th eller nh) ikke ma=lb ikke ma=lm | 8263 |
| S4 | (”væbnede styrker” eller tropper eller militær eller ”militære styrker” eller flåden eller marinen eller søværnet eller luftvåben eller flyvevåben eller luftstyrke ikke ma=(th eller nh) ikke ma=lb ikke ma=lm) eller (em=mandskab ikke ma=(th eller nh) ikke ma=lb ikke ma=lm) | 11817 |
| S6 | ((”væbnede styrker” eller tropper eller militær eller ”militære styrker” eller flåden eller marinen eller søværnet eller luftvåben eller flyvevåben eller luftstyrke ikke ma=(th eller nh) ikke ma=lb ikke ma=lm) eller (em=mandskab ikke  ma=(th eller nh) ikke ma=lb ikke ma=lm)) og ((fredsbevarende og mission?) eller ”aktiv tjeneste” eller  ”kamp tjeneste” eller ”militær tjeneste” eller ”aktiv tjeneste” eller ”kamp tjeneste” eller ”militær tjeneste” eller  deployeret eller udstationer? Eller hjemsendelse eller hjemsendt? Ikke ma=(th eller nh) ikke ma=lb ikke  ma=lm) | 62 |
| S7 | veteran? ikke ma=(th eller nh) ikke ma=lb ikke ma=lm | 2786 |
| S8 | em=veteraner ikke ma=(th eller nh) ikke ma=lb ikke ma=lm | 33 |
| S9 | (veteran? ikke ma=(th eller nh) ikke ma=lb ikke ma=lm) eller (em=veteraner ikke ma=(th eller nh) ikke ma=lb ikke  ma=lm) | 2789 |
| S10 | krig eller kamp eller ”aktiv tjeneste” eller”kamp tjeneste” eller ”militær tjeneste” eller ”aktiv militær tjeneste” ikke ma=(th eller nh) ikke ma=lb ikke ma=lm | 33958 |
| S11 | ((veteran? ikke ma=(th eller nh) ikke ma=lb ikke ma=lm) eller (em=veteraner ikke ma=(th eller nh) ikke ma=lb ikke  ma=lm)) og (krig eller kamp eller ”aktiv tjeneste” eller ”kamp tjeneste” eller ”militær tjeneste” eller ”aktiv militær tjeneste” ikke ma=(th eller nh) ikke ma=lb ikke ma=lm) | 43 |
| S12 | (((”væbnede styrker” eller tropper eller militær eller ”militære styrker” eller flåden eller marinen eller søværnet eller luftvåben eller flyvevåben eller luftstyrke ikke ma=(th eller nh) ikke ma=lb ikke ma=lm) eller (em=mandskab ikke ma=(th eller nh) ikke ma=lb ikke ma=lm)) og ((fredsbevarende og  mission?) eller ”aktiv tjeneste” eller ”kamp tjeneste” eller ”militær tjeneste” eller ”aktiv tjeneste” eller ”kamp tjeneste” eller ”militær tjeneste” eller deployeret eller udstationer? eller  hjemsendelse eller hjemsendt? Ikke ma=(th eller nh) ikke ma=lb ikke ma=lm)) eller (((veteran? ikke ma=(th  eller nh) ikke ma=lb ikke ma=lm) eller (em=veteraner ikke ma=(th eller nh) ikke ma=lb ikke ma=lm)) og (krig eller  kamp eller ”aktiv tjeneste” eller ”kamp tjeneste” eller ”militær tjeneste” eller ”aktiv militær tjeneste” ikke ma=(th eller nh) ikke ma=lb ikke ma=lm)) | 104 |

## Bibsys. Last Search: January 2015

| Search number | Term | Totals |
| --- | --- | --- |
| S1 | væpnede styrker eller tropper eller militær eller militære styrker eller sjøforsvaret eller marinen eller luftforsvaret eller flyvåpen | 0 |
| S2 | væpnede styrker eller tropper eller militær eller militære styrker eller sjøforsvaret eller marinen eller luftforsvaret eller flyvåpen | 5387 |
| S3 | emne = militær eller frie emneord = militær | 1122 |
| S4 | soldat eller marinesoldat? og militærtjeneste og tjeneste og militært personell og militære styrker og marine personell og sjøforsvarets personell og luftforsvarspersonell | 0 |
| S5 | soldat eller marinesoldat? eller militærtjeneste eller tjeneste eller militært personell eller militære styrker eller marine personell eller sjøforsvarets personell eller luftforsvarspersonell | 2661 |
| S6 | væpnede styrker eller tropper eller militær eller militære styrker eller sjøforsvaret eller marinen eller luftforsvaret eller flyvåpen eller emne = militær eller frie emneord = militær eller soldat eller marinesoldat? eller militærtjeneste eller tjeneste eller militært personell eller militære styrker eller marine personell eller sjøforsvarets personell eller luftforsvarspersonell | 7843 |
| S7 | fredsbevarende oppdrag eller aktiv tjeneste eller kamp tjeneste eller militærtjeneste eller utstasjonert eller utstasjonering eller hjemsendelse eller hjemsendt | 98 |
| S8 | væpnede styrker eller tropper eller militær eller militære styrker eller sjøforsvaret eller marinen eller luftforsvaret eller flyvåpen eller emne = militær eller frie emneord = militær eller soldat eller marinesoldat? eller militærtjeneste eller tjeneste eller militært personell eller militære styrker eller marine personell eller sjøforsvarets personell eller  luftforsvarspersonell og fredsbevarende oppdrag eller aktiv tjeneste eller kamp tjeneste eller militærtjeneste eller utstasjonert eller utstasjonering eller hjemsendelse eller  hjemsendt | 80 |
| S9 | veteran? eller emne = veteraner eller frie emneord = veteran | 695 |
| S10 | veteran? eller emne = veteraner eller frie emneord = veteraner | 695 |
| S11 | krig eller kamp eller aktiv tjeneste eller kamp tjeneste eller militærtjeneste eller aktiv militær tjeneste | 12916 |
| S12 | veteran? eller emne = veteraner eller frie emneord = veteraner og krig eller kamp eller aktiv tjeneste eller kamp tjeneste eller militærtjeneste eller aktiv militær tjeneste | 18 |
| S13 | væpnede styrker eller tropper eller militær eller militære styrker eller sjøforsvaret eller marinen eller luftforsvaret eller flyvåpen eller emne = militær eller frie emneord = militær  eller soldat eller marinesoldat? eller militærtjeneste eller tjeneste eller militært personell eller militære styrker eller marine personell eller sjøforsvarets personell eller  luftforsvarspersonell og fredsbevarende oppdrag eller aktiv tjeneste eller kamp tjeneste eller militærtjeneste eller utstasjonert eller utstasjonering eller hjemsendelse eller  hjemsendt eller veteran? eller emne = veteraner eller frie emneord = veteraner og krig eller kamp eller aktiv tjeneste eller kamp tjeneste eller militærtjeneste eller aktiv militær tjeneste | 98 |

## Cochrane Library. Last Search: January 2015

| Search number | Term | Totals |
| --- | --- | --- |
| S1 | Soldier* or Marine* or airm?n* or Veteran* or militarian* or officer*:ti,ab,kw or (Army or troop* or militar* or navy or ((armed or naval or air or defen?e or national) near/1 Force*)):ti,ab,kw or Military Personnel or veterans:kw (Word variations have been searched) | 5084 |
| S2 | war:kw or war or wars or warfare* or (combat* or deploy* or post-deploy* or postdeploy*) or ((peace-keep* or peacekeep* or peace keep* or military) near/1 mission*) or (active near/1 duty) (Word variations have been searched) | 2245 |
| S3 | S1 and S2 | 531 |
| S4 | Randomized Controlled Trial or random allocation:kw or (randomized or placebo or randomly):ab or trials or groups:ab or (randomized controlled trial or controlled clinical trial):pt (Word variations have been searched) | 603869 |
| S5 | MeSH descriptor: [Case-Control Studies] explode all trees | 11472 |
| S6 | MeSH descriptor: [Cohort Studies] explode all trees | 113162 |
| S7 | S5 OR S6 | 115884 |
| S8 | "comparative study":pt (Word variations have been searched) | 140836 |
| S9 | MeSH descriptor: [Cross-Sectional Studies] this term only | 3124 |
| S10 | MeSH descriptor: [Epidemiologic Studies] this term only | 53 |
| S11 | (case control or cohort or cross sectional or cross-sectional or point in time or point-in-time) (Word variations have been searched) | 122690 |
| S12 | ((epidemiolog* near/2 study) or ((follow up or followup or follow-up) near/2 study)) (Word variations have been searched) | 56542 |
| S13 | (longitudinal or observational or (prospective near/2 study) or retrospective) | 72315 |
| S14 | MeSH descriptor: [Intervention Studies] this term only | 2186 |
| S15 | quasi-experiment* or quasiexperiment* or experiment* | 65569 |
| S16 | (assign* near/5 (subject* or patient* or client*)) | 93891 |
| S17 | (Propensity score* or heckman or difference in difference or regression discontinuity (compar* near/1 group*) or (match* near/1 control*) or (match* near/1 compar*) or assessment only or comparison samp* or propensity-matched):ti,ab,kw (Word variations have been searched) | 214865 |
| S18 | (Non-random* or nonradom* or (non adj1 random*)) or rct | 220696 |
| S19 | S4 OR S7 OR S8 OR S9 OR S10 OR S11 OR S12 OR OR13 OR S14 OR S15 OR S16 OR S17 | 690261 |
| S20 | S3 AND S19 | 490 |

## ERIC. Last Search: January 2015

| Search number | Term | Totals |
| --- | --- | --- |
| S1 | AB randomized | 3,118 |
| S2 | AB placebo | 669 |
| S3 | AB randomly | 11,948 |
| S4 | AB trial | 9,671 |
| S5 | AB groups | 192,169 |
| S6 | S1 OR S2 OR S3 OR S4 OR S5 | 205,887 |
| S7 | Review | 161,261 |
| S8 | S6 AND S7 | 18,144 |
| S9 | DE "COHORT analysis" OR DE "Case Studies" | 42,143 |
| S10 | DE "CROSS-sectional method" OR DE "Comparative Analysis" | 61,712 |
| S11 | TI (case control) or AB (case control) | 590 |
| S12 | TI cohort OR AB cohort | 8,234 |
| S13 | TI (cross sectional or cross-sectional or point in time or point-in-time) or AB ( cross sectional or cross-sectional or point in time or point-in-time) | 6,367 |
| S14 | TI (epidemiologic N2 study) or AB (epidemiologic N2 study) | 139 |
| S15 | Ti ((follow up or followup or follow-up) N2 study )OR AB ((follow up or followup or follow-up) N2 study) | 4,099 |
| S16 | TI longitudinal or AB longitudinal | 20,915 |
| S17 | TI observational OR AB observational | 3,738 |
| S18 | TI (prospective n2 study) or AB (prospective n2 study) | 1,065 |
| S19 | TI retrospective OR AB retrospective | 2,869 |
| S20 | TI Intervention* N1 Stud* OR AB Intervention* N1 Stud* | 2,587 |
| S21 | Ti (quasi-experiment* or quasiexperiment* or experiment*) OR AB (quasi-experiment* or quasiexperiment* or experiment*) | 65,342 |
| S22 | TI assign* N3 (subject* or patient* or client*) or AB assign* N3 (subject* or patient* or client* ) | 1,076 |
| S23 | TI (Propensity score* or heckman or difference in difference or regression discontinuity or (match* N1 control*) or (match* N1 compar* ) or assessment only or comparison samp* or propensity match*) OR AB (Propensity score* or heckman or difference in difference or regression discontinuity or (match* N1 control*) or (match* N1 compar* ) or assessment only or comparison samp* or propensity match*) | 115,455 |
| S24 | TI Non-random* or nonradom* or (non N1 random*) OR AB Non-random* or nonradom* or (non N1 random*) | 220 |
| S25 | TI ((random* N2 trial*) or RCT) OR AB ( (random* N2 trial*) or RCT) | 2,112 |
| S26 | (DE "Military Personnel" OR DE "Air Force Personnel" OR DE "Army Personnel" OR DE "Commissioned Officers" OR DE "Enlisted Military Personnel" OR DE "Marine Personnel" OR DE "Navy Personnel" OR DE "Volunteer Military Personnel") | 2,537 |
| S27 | TI ( (Soldier* or Marine* or airm#n* or Veteran* or militarian* or officer*) ) OR AB ( (Soldier* or Marine* or airm#n* or Veteran* or militarian* or officer*) ) | 14,312 |
| S28 | TI ( Army or troop* or military* or navy) OR AB ( Army or troop* or military* or navy) | 7,777 |
| S29 | TI ((armed or naval or navy or air or defen#e* or national) N1 force* ) OR AB ( (armed or naval or navy or air or defen#e or national) N1 force1 ) | 401 |
| S30 | DE "War" | 2,907 |
| S31 | TI (war or wars or warfare*) OR AB (war or wars or warfare*) | 10,394 |
| S32 | TI (combat* or deploy* or post-deploy* or postdeploy*) OR AB (combat* or deploy* or post-deploy* or postdeploy*) | 5,226 |
| S33 | TI ((peace-keep* or peacekeep* or peace or keep* or military) N1 mission*) OR AB ((peace-keep* or peacekeep* or peace keep* or military) N1 mission*) | 19 |
| S34 | TI (active N1 duty) OR AB (active N1 duty) | 153 |
| S35 | S30 OR S31 OR S32 OR S33 OR S34 | 16,276 |
| S36 | DE "VETERANS" | 23 |
| S37 | S26 OR S27 OR S28 OR S29 OR S36 | 21,364 |
| S38 | S35 AND S37 | 2,161 |
| S39 | (S9 OR S10 OR S11 OR S12 OR S13 OR S14 OR S15 OR S16 OR S17 OR S18 OR S19 OR S20 OR S21 OR S23 OR S24 OR S25) | 282,455 |
| S40 | S8 OR S39 | 295,508 |
| S41 | S38 AND S40 | 350 |

## EMBASE. Last Search: January 2015

| Search number | Term | Totals |
| --- | --- | --- |
| s1 | Military Personnel/ | 22339 |
| s2 | Veterans/ | 9354 |
| s3 | (Soldier* or Marine* or airm#n or Veteran* or militarian* or officer*).ti,ab. | 114775 |
| s4 | (Army or troop* or military* or navy or ((armed or naval or navy or air or defen#e or national) adj1 Force*)).ti,ab. | 55205 |
| s5 | or/1-4 | 169638 |
| s6 | exp War/ | 25987 |
| s7 | (war or wars or warfare*).tw. | 35197 |
| s8 | (combat* or deploy* or post-deploy* or postdeploy*).tw. | 59722 |
| s9 | ((peace-keep* or peacekeep* or peace keep* or military) adj1 mission*).tw. | 201 |
| s10 | (active adj1 duty).tw. | 2157 |
| s11 | or/6-10 | 105377 |
| s12 | 5 and 11 | 20223 |
| s13 | Randomized Controlled Trial/ or random allocation/ | 413485 |
| s14 | (randomized or placebo or randomly).ab. | 752419 |
| s15 | (trial or groups).ab. | 2122848 |
| s16 | exp case control studies/ or exp cohort studies/ | 269577 |
| s17 | Comparative Study.tw. or Comparative Study/ | 691586 |
| s18 | Cross-sectional studies/ or Epidemiologic studies/ | 307412 |
| s19 | (case control or cohort or cross sectional or cross-sectional or point in time or point-in-time).tw. | 685891 |
| s20 | ((epidemiologic adj2 study) or ((follow up or followup or follow-up) adj2 study)).tw. | 49726 |
| s21 | (longitudinal or observational or (prospective adj2 study) or retrospective).tw. | 885507 |
| s22 | Intervention Studies/ | 21855 |
| s23 | (quasi-experiment* or quasiexperiment* or experiment*).tw. | 1646046 |
| s24 | (assign* adj5 (subject* or patient* or client)).ab,ti. | 42353 |
| s25 | (Propensity score* or heckman or difference in difference or regression discontinuity or (compar* adj1 group*) or (match* adj1 control*) or (match* adj1 compar*) or assessment only or comparison samp* or propensity-matched).ab,ti. | 189677 |
| s26 | (Non-random* or nonradom* or (non adj1 random*)).ab,ti. | 16387 |
| s27 | rct.tw. | 15521 |
| s28 | or/13-27 | 5770857 |
| s29 | 12 and 28 | 5638 |
| s30 | limit 29 to human | 4937 |
|  |  |  |

## LIBRIS. Last Search: January 2015

| Search number | Term | Totals |
| --- | --- | --- |
| S1 | Enkel sökning > ((”väpnade styrkor” OR trupper OR militära OR ”militära styrkor” OR marinen OR flottan OR navy OR flygvapen OR flygvapnet OR (zamn:"^Militär^") OR Soldat  OR marinsoldat* OR militärtjänst OR tjänst OR (militärt adj1 personal) OR “militära trupper” OR ”marin manskap” OR ”marin personell” OR (flygvapen adj1 (manskap eller  personell))) AND (”fredsbevarande uppdrag” OR ”aktiv tjänst” OR ”kamp tjänst” OR ”militär tjänst” OR utlandstjänst* OR utstationering OR repatriering OR repatrier*)) OR (veteran*  OR (zamn:"^Veterans^")) AND (Krig OR kamp OR ”aktiv tjänst” OR ”kamp tjänst” OR ”militär tjänst” OR ”aktiv militär tjänst”) | 55 |
| S2 | Enkel sökning > (veteran* OR (zamn:"^Veterans^")) AND (Krig OR kamp OR ”aktiv tjänst”  OR ”kamp tjänst” OR ”militär tjänst” OR ”aktiv militär tjänst”) | 27 |
| S3 | Enkel sökning > (Krig OR kamp OR ”aktiv tjänst” OR ”kamp tjänst” OR ”militär tjänst” OR ”aktiv militär tjänst”) | 11923 |
| S4 | Enkel sökning > (veteran* OR (zamn:"^Veterans^")) | 1068 |
| S5 | Enkel sökning > (zamn:"^Veterans^") | 6 |
| S6 | Enkel sökning > veteran* | 1068 |
| S7 | Enkel sökning > ((”väpnade styrkor” OR trupper OR militära OR ”militära styrkor” OR marinen OR flottan OR navy OR flygvapen OR flygvapnet OR (zamn:"^Militär^") OR Soldat  OR marinsoldat* OR militärtjänst OR tjänst OR (militärt adj1 personal) OR “militära trupper” OR ”marin manskap” OR ”marin personell” OR (flygvapen adj1 (manskap eller  personell))) AND (”fredsbevarande uppdrag” OR ”aktiv tjänst” OR ”kamp tjänst” OR ”militär tjänst” OR utlandstjänst* OR utstationering OR repatriering OR repatrier*)) | 28 |
| S8 | Enkel sökning > (”fredsbevarande uppdrag” OR ”aktiv tjänst” OR ”kamp tjänst” OR ”militär tjänst” OR utlandstjänst* OR utstationering OR repatriering OR repatrier*) | 271 |
| S9 | Enkel sökning > (”väpnade styrkor” OR trupper OR militära OR ”militära styrkor” OR marinen OR flottan OR navy OR flygvapen OR flygvapnet OR (zamn:"^Militär^") OR Soldat  OR marinsoldat* OR militärtjänst OR tjänst OR (militärt adj1 personal) OR “militära trupper” OR ”marin manskap” OR ”marin personell” OR (flygvapen adj1 (manskap eller  personell))) | 15953 |
| S10 | Enkel sökning > Soldat OR marinsoldat* OR militärtjänst OR tjänst OR (militärt adj1 personal) OR “militära trupper” OR ”marin manskap” OR ”marin personell” OR (flygvapen  adj1 (manskap eller personell)) | 4997 |
| S11 | Enkel sökning > (zamn:"^Militär^") | 19 |
| S12 | Boolesk > ”väpnade styrkor” OR trupper OR militära OR ”militära styrkor” OR marinen OR flottan OR navy OR flygvapen OR flygvapnet | 10991 |

## MEDLINE. Last Search: January 2015

| Search number | Term | Totals |
| --- | --- | --- |
| 1 | Military Personnel/ | 25231 |
| 2 | Veterans/ | 9892 |
| 3 | (Soldier* or Marine* or Veteran* or militarian* or officer*).ti,ab. | 85547 |
| 4 | (Army or troop* or military* or ((armed or naval or navy or air or defence or national) adj1 Force*)).ti,ab. | 41901 |
| 5 | or/1-4 | 132126 |
| 6 | exp War/ | 30927 |
| 7 | (war or wars or warfare*).tw. | 29333 |
| 8 | (combat* or deploy* or post-deploy* or postdeploy*).tw. | 41783 |
| 9 | ((peace-keep* or peacekeep* or military) adj1 mission*).tw. | 164 |
| 10 | (active adj1 duty).tw. | 1741 |
| 11 | or/6-10 | 89520 |
| 12 | 5 and 11 | 17368 |
| 13 | Randomized Controlled Trial/ or random allocation/ | 444644 |
| 14 | (randomized controlled trial or controlled clinical trial).pt. | 464239 |
| 15 | (randomized or placebo or randomly).ab. | 528688 |
| 16 | (trial or groups).ab. | 1488046 |
| 17 | 13 or 14 or 15 or 16 | 1892593 |
| 18 | humans.sh. | 13614781 |
| 19 | 17 and 18 | 1433998 |
| 20 | exp case control studies/ or exp cohort studies/ | 1529814 |
| 21 | Comparative Study.pt. | 1681987 |
| 22 | Cross-sectional studies/ or Epidemiologic studies/ | 189142 |
| 23 | (case control or cohort or cross sectional).tw. | 446774 |
| 24 | ((epidemiologic adj2 study) or ((follow up or followup) adj2 study)).tw. | 37756 |
| 25 | (longitudinal or observational or (prospective adj2 study) or retrospective).tw. | 581483 |
| 26 | Intervention Studies/ | 7115 |
| 27 | (quasi-experiment* or quasiexperiment* or experiment*).tw. | 1280529 |
| 28 | (assign* adj5 (subject* or patient*)).ab,ti. | 30419 |
| 29 | (Propensity score* or (compar* adj1 group*) or (match* adj1 control*) or (match* adj1 compar*) or assessment only or comparison samp* or propensity-matched).ab,ti. | 130549 |
| 30 | (Non-random* or nonradom* or (non adj1 random*)).ab,ti. | 10845 |
| 31 | rct.tw. | 7934 |
| 32 | limit 19 to "reviews (maximizes specificity)" | 40501 |
| 33 | or/20-32 | 4529038 |
| 34 | 12 and 33 | 4667 |
| 35 | limit 34 to humans | 4462 |

## PsycINFO. Last Search: January 2015

| Search number | Term | Totals |
| --- | --- | --- |
| S1 | DE "RANDOMIZED controlled trials" | 0 |
| S2 | DE "RANDOMIZATION (Statistics)" | 0 |
| S3 | AB randomized | 41,613 |
| S4 | AB placebo | 31,503 |
| S5 | AB randomly | 53,300 |
| S6 | AB trial | 119,790 |
| S7 | AB groups | 653,727 |
| S8 | S1 OR S2 OR S3 OR S4 OR S5 OR S6 OR S7 | 780,069 |
| S9 | Review | 449,134 |
| S10 | S8 and s9 | 66,705 |
| S11 | DE "COHORT analysis" OR DE "Case Studies" | 1,077 |
| S12 | DE "CROSS-sectional method" OR DE "Comparative Analysis" | 0 |
| S13 | TI (case control) or AB (case control) | 11,563 |
| S14 | TI cohort OR AB cohort | 45,074 |
| S15 | (TI cross sectional or cross-sectional or point in time or point-in-timeor) or (AB cross sectional or cross-sectional or point in time or point-in-time) | 60,832 |
| S16 | TI (epidemiologic N2 study) or AB (epidemiologic N2 study) | 3,902 |
| S17 | Ti ((follow up or followup or follow-up) N2 study ) OR AB ((follow up or followup or follow-up) N2 study) | 12,631 |
| S18 | TI longitudinal or AB longitudinal | 72,918 |
| S19 | TI observational OR AB observational | 17,222 |
| S20 | TI (prospective n2 study) or AB (prospective n2 study) | 20,906 |
| S21 | TI retrospective OR AB retrospective | 23,334 |
| S22 | TI Intervention* N1 Stud* OR AB Intervention* N1 Stud* | 9,452 |
| S23 | Ti (quasi-experiment* or quasiexperiment* or experiment*) OR AB (quasi-experiment* or quasiexperiment* or experiment*) | 329,925 |
| S24 | TI assign* N3 (subject* or patient* or client* ) or AB assign*S N3 (subject* or patient* or client*) | 4,872 |
| S25 | TI (Propensity score* or heckman or difference in difference or regression discontinuity or (match* N1 control*) or (match* N1 compar* ) or assessment only or comparison samp* or propensity match*) OR AB (Propensity score* or heckman or difference in difference or regression discontinuity or (match* N1 control*) or (match* N1 compar* ) or assessment only or comparison samp* or propensity match*) | 505,619 |
| S26 | TI Non-random* or nonradom* or (non N1 random*) OR AB Non-random* or nonradom* or (non N1 random*) | 1,580 |
| S27 | TI ((random* N2 trial*) or RCT) OR AB ( (random* N2 trial*) or RCT) | 30,467 |
| S28 | (DE "Military Personnel" OR DE "Air Force Personnel" OR DE "Army Personnel" OR DE "Commissioned Officers" OR DE "Enlisted Military Personnel" OR DE "Marine Personnel" OR DE "Navy Personnel" OR DE "Volunteer Military Personnel") | 12,639 |
| S29 | TI ( (Soldier* or Marine* or airm#n or Veteran* or militarian* or officer*) ) OR AB ( (Soldier* or Marine* or airm#n or Veteran* or militarian* or officer*) ) | 33,997 |
| S30 | TI ( Army or troop* or military* or navy ) OR AB ( Army or troop* or military* or navy) | 24,573 |
| S31 | TI ((armed or naval or navy or air or defence or national) N1 force* ) OR AB ( (armed or naval or navy or air or defence or national) N1 force1 ) | 1,022 |
| S32 | DE "War" | 10,936 |
| S33 | TI (war or wars or warfare*) OR AB (war or wars or warfare*) | 27,923 |
| S34 | TI (combat* or deploy* or post-deploy* or postdeploy*) OR AB (combat* or deploy* or post-deploy* or postdeploy*) | 19,755 |
| S35 | TI ((peace-keep* or peacekeep* or peace keep* or military) N1 mission*) OR AB ((peace-keep* or peacekeep* or peace keep* or military) N1 mission*) | 172 |
| S36 | TI (active N1 duty) OR AB (active N1 duty) | 1,254 |
| S37 | S32 OR S33 OR S34 OR S35 OR S36 | 46,987 |
| S38 | DE "VETERANS" OR DE "Military Veterans" | 9,923 |
| S39 | S28 OR S29 OR S30 OR S31 OR S38 | 54,746 |
| S40 | S37 AND S39 | 13,457 |
| S41 | (S11 OR S12 OR S13 OR S14 OR S15 OR S16 OR S17 OR S18 OR S19 OR S20 OR S21 OR S22 OR S23 OR S25 OR S26 OR S27) | 973,514 |
| S42 | S10 OR S41 | 1,018,108 |
| S43 | S40 AND S42 | 3,145 |

## Science Citation Index (SCI). Last Search: January 2015

| Search number | Term | Totals |
| --- | --- | --- |
| S1 | Topic=((Soldier* or Marine* or Airm?n or Veteran or Militarian* or Officer* or Army or Troop or Military* or Navy or (Armed or Naval or Navy or Air or Defen?e or NaTopic=onal) near/1 Force*) | 334,889 |
| S2 | Topic=((Peace-keep* or Peacekeep* or ( Peace NEAR/1 keep*)) NEAR/1 mission* ) or (war or wars or warfare or combat* or deploy* or post-deploy* or postdeploy*) or (active near/1 duty)) | 137,179 |
| S3 | #2 and #1 | [17,445](http://apps.webofknowledge.com/summary.do?product=WOS&doc=1&qid=60&SID=R2hKb8JK3dF3dHOe7jp&search_mode=CombineSearches) |
| S4 | Topic=(Randomized or Placebo or Randomly or Trial or Group) | 3,951324 |
| S5 | Topic=(Review) | 1,362,818 |
| S6 | S4 AND S9 | 311,464 |
| S7 | Topic=( (case control) or cohort) | 710,284 |
| S8 | Topic=(Comparative study) | 223,331 |
| S9 | Topic= (cross sectional or cross-sectional or point in time or point-in-time) | 193,466 |
| S10 | Topic= (epidemiological near/2 study) | 20,034 |
| S11 | Topic= ((follow up or followup) near study )) | 49,589 |
| S12 | Topic= (longitudinal ) | 223,757 |
| S13 | Topic=( observational) | 123,036 |
| S14 | Topic=( (prospective near/2 study)) | 221,377 |
| S15 | Topic=( retrospective ) | 276,860 |
| S16 | Topic=( Intervention* near Stud* ) | 24,636 |
| S17 | Topic=( (quasi-experiment* or quasiexperiment* or experiment*) ) | 3,208,541 |
| S18 | Topic=( assign* near (subject* or patient* or client*) | 30,851 |
| S19 | Topic=( (Propensity score* or heckman or difference in difference or regression discontinuity or (match* near control*) or (match* near compar* ) or assessment only or comparison samp* or propensity matched))) | 2,161,390 |
| S20 | Topic=( Non-random* or nonradom* or (non random*) ) | 16,640 |
| S21 | Topic= ((random* near trial*) or RCT) | 353,265 |
| S22 | S4-S21/ | 6,978,619 |
| S23 | S3 and S22 | 5,922 |

## Social Science Citation Index (SSCI). Last Search: January 2015

| Search number | Term | Totals |
| --- | --- | --- |
| S1 | Topic=((Soldier* or Marine* or Airm?n or Veteran or Militarian* or Officer* or Army or Troop or Military* or Navy or (Armed or Naval or Navy or Air or Defen?e or NaTopic=onal) near/1 Force*) | 80,658 |
| S2 | Topic=((Peace-keep* or Peacekeep* or ( Peace NEAR/1 keep*)) NEAR/1 mission* ) or (war or wars or warfare or combat* or deploy* or post-deploy* or postdeploy*) or (active near/1 duty)) | 120,192 |
| S3 | #2 and #1 | [18,454](http://apps.webofknowledge.com/summary.do?product=WOS&doc=1&qid=60&SID=R2hKb8JK3dF3dHOe7jp&search_mode=CombineSearches) |
| S4 | Topic=(Randomized or Placebo or Randomly or Trial or Group) | 630,071 |
| S5 | Topic=(Review) | 237,121 |
| S6 | S4 AND S5 | 52,019 |
| S7 | Topic=( (case control) or cohort) | 111,426 |
| S8 | Topic=(Comparative study) | 34,400 |
| S9 | Topic= (cross sectional or cross-sectional or point in time or point-in-time) | 71,889 |
| S10 | Topic= (epidemiological near/2 study) | 7,038 |
| S11 | Topic= ((follow up or followup) near study )) | 11,253 |
| S12 | Topic= (longitudinal ) | 80,183 |
| S13 | Topic=( observational) | 17,316 |
| S14 | Topic=( (prospective near/2 study)) | 26,527 |
| S15 | Topic=( retrospective ) | 27,446 |
| S16 | Topic=( Intervention* near Stud* ) | 10,792 |
| S17 | Topic=( (quasi-experiment* or quasiexperiment* or experiment*) ) | 193,540 |
| S18 | Topic=( assign* near (subject* or patient* or client*) | 5,369 |
| S19 | Topic=( (Propensity score* or heckman or difference in difference or regression discontinuity or (match* near control*) or (match* near compar* ) or assessment only or comparison samp* or propensity matched))) | 409,163 |
| S20 | Topic=( Non-random* or nonradom* or (non random*) ) | 2,022 |
| S21 | Topic= ((random* near trial*) or RCT) | 69,367 |
| S22 | S4-S21/ | 905,176 |
| S23 | S3 and S22 | 3,865 |

## SocINDEX. Last Search: January 2015

| Search number | Term | Totals |
| --- | --- | --- |
| S1 | DE "RANDOMIZED controlled trials" | 1,103 |
| S2 | DE "RANDOMIZATION (Statistics)" | 13 |
| S3 | AB randomized | 6,129 |
| S4 | AB placebo | 1,911 |
| S5 | AB randomly | 9,542 |
| S6 | AB trial | 27,695 |
| S7 | AB groups | 210,073 |
| S8 | S1 OR S2 OR S3 OR S4 OR S5 OR S6 OR S7 | 239,567 |
| S9 | review | 757,779 |
| S10 | S8 AND S9 | 33,956 |
| S11 | DE "COHORT analysis" OR DE "Case Studies" | 6,306 |
| S12 | DE "CROSS-sectional method" OR DE "Comparative Analysis" | 2,916 |
| S13 | TI (case control) or AB (case control) | 2,725 |
| S14 | TI cohort OR AB cohort | 14,425 |
| S15 | (TI cross sectional or cross-sectional or point in time or point-in-time) or (AB cross sectional or cross-sectional or point in time or point-in-time) | 16,039 |
| S16 | TI (epidemiologic N2 study) or AB (epidemiologic N2 study) | 803 |
| S17 | Ti ((follow up or followup or follow-up ) N2 study )OR AB ((follow up or followup or follow-up ) N2 study) | 2,472 |
| S18 | TI longitudinal or AB longitudinal | 23,744 |
| S19 | TI observational OR AB observational | 3,241 |
| S20 | TI (prospective n2 study) or AB (prospective n2 study) | 4,015 |
| S21 | TI retrospective OR AB retrospective | 5,247 |
| S22 | TI Intervention* N1 Stud* OR AB Intervention* N1 Stud* | 1,664 |
| S23 | Ti (quasi-experiment* or quasiexperiment* or experiment*) OR AB (quasi-experiment* or quasiexperiment* or experiment*) | 46,214 |
| S24 | TI assign* N3 (subject* or patient* or client*) or AB assign* N3 (subject* or patient* or client*) | 1,070 |
| S25 | TI (Propensity score* or heckman or difference in difference or regression discontinuity or (match* N1 control*) or (match* N1 compar* ) or assessment only or comparison samp* or propensity match*) OR AB (Propensity score* or heckman or difference in difference or regression discontinuity or (match* N1 control*) or (match* N1 compar* ) or assessment only or comparison samp* or propensity match*) | 134,299 |
| S26 | TI Non-random* or nonradom* or (non N1 random*) OR AB Non-random* or nonradom* or (non N1 random*) | 361 |
| S27 | TI ((random* N2 trial*) or RCT) OR AB ( (random* N2 trial*) or RCT) | 4,981 |
| S28 | (DE "Military Personnel" OR DE "Air Force Personnel" OR DE "Army Personnel" OR DE "Commissioned Officers" OR DE "Enlisted Military Personnel" OR DE "Marine Personnel" OR DE "Navy Personnel" OR DE "Volunteer Military Personnel") | 1,175 |
| S29 | TI ( (Soldier* or Marine* or airm#n* or Veteran* or militarian* or officer*) ) OR AB ( (Soldier* or Marine* or airm#n* or Veteran* or militarian* or officer*) ) | 46,424 |
| S30 | TI ( Army or troop* or military* or navy) OR AB ( Army or troop* or military* or navy) | 34,957 |
| S31 | TI ((armed or naval or navy or air or defe#ce or national) N1 force* ) OR AB ( (armed or naval or navy or air or defe#ce or national) N1 force1 ) | 662 |
| S32 | DE "War" | 8,160 |
| S33 | TI (war or wars or warfare*) OR AB (war or wars or warfare*) | 78,528 |
| S34 | TI (combat* or deploy* or post-deploy* or postdeploy*) OR AB (combat* or deploy* or post-deploy* or postdeploy*) | 16,061 |
| S35 | TI ((peace-keep* or peacekeep* or peace keep* or military) N1 mission*) OR AB ((peace-keep* or peacekeep* or peace keep* or military) N1 mission*) | 373 |
| S36 | TI (active N1 duty) OR AB (active N1 duty) | 407 |
| S37 | S32 OR S33 OR S34 OR S35 OR S36 | 94,029 |
| S38 | DE "VETERANS" OR DE "Military Veterans" | 3,203 |
| S39 | S28 OR S29 OR S30 OR S31 OR S38 | 78,144 |
| S40 | S37 AND S39 | 16,082 |
| S41 | (S11 OR S12 OR S13 OR S14 OR S15 OR S16 OR S17 OR S18 OR S19 OR S20 OR S21 OR S22 OR S23 OR S25 OR S26 OR S27) | 232,038 |
| S42 | S10 OR S41 | 258,654 |
| S43 | S40 AND S42 | 1,348 |

## Academic Search Premier 01/01 2015 - 31/3 2017

| **Search** | **Terms** | **Results** |
| --- | --- | --- |
| S44 | S40 AND S42 **Limiters** - Published Date: 20150101-20171231 | 1,171 |
| S43 | S40 AND S42 | 6,186 |
| S42 | S10 OR S41 | 3,721,420 |
| S41 | S11 OR S12 OR S13 OR S14 OR S15 OR S16 OR S17 OR S18 OR S19 OR S20 OR S21 OR S22 OR S23 OR S25 OR S26 OR S27 | 3,606,501 |
| S40 | S37 AND S39 | 127,305 |
| S39 | S28 OR S29 OR S30 OR S31 OR S38 | 687,974 |
| S38 | SU "veterans" | 16,866 |
| S37 | S32 OR S33 OR S34 OR S35 OR S36 | 548,465 |
| S36 | TI (active N1 duty) OR AB (active N1 duty) | 3,010 |
| S35 | TI ( ((peace-keep* or peacekeep* or peace keep* or military) N1 mission*) ) OR AB ( ((peace-keep* or peacekeep* or peace keep* or military) N1 mission*) ) | 2,248 |
| S34 | TI ( (combat* or deploy* or post-deploy* or postdeploy*) ) OR AB ( (combat* or deploy* or post-deploy* or postdeploy*) ) | 134,689 |
| S33 | TI ( (war or wars or warfare*) ) OR AB ( (war or wars or warfare*) ) | 383,598 |
| S32 | SU "War" | 163,052 |
| S31 | TI ( ((armed or naval or navy or air or defen#e* or national) N1 force* ) ) OR AB ( ((armed or naval or navy or air or defen#e* or national) N1 force* ) ) | 54,173 |
| S30 | TI ( ( Army or troop* or military* or navy) ) OR AB ( ( Army or troop* or military* or navy) ) | 306,647 |
| S29 | TI ( ( (Soldier* or Marine* or airm#n* or Veteran* or militarian* or officer*) ) ) OR AB ( ( (Soldier* or Marine* or airm#n* or Veteran* or militarian* or officer*) ) ) | 391,737 |
| S28 | SU ("Military Personnel" OR "Air Force Personnel" OR "Army Personnel" OR "Commissioned Officers" OR "Enlisted Military Personnel" OR "Marine Personnel" OR "Navy Personnel" OR "Volunteer Military Personnel") | 33,231 |
| S27 | TI ( ((random* N2 trial*) or RCT) ) OR AB ( ((random* N2 trial*) or RCT) ) | 128,478 |
| S26 | TI ( Non-random* or nonradom* or (non N1 random*) OR ) OR AB ( Non-random* or nonradom* or (non N1 random*) OR ) | 10,194 |
| S25 | TI ( (Propensity score* or heckman or difference in difference or regression discontinuity or (match* N1 control*) or (match* N1 compar* ) or assessment only or comparison samp* or propensity match*) ) OR AB ( (Propensity score* or heckman or difference in difference or regression discontinuity or (match* N1 control*) or (match* N1 compar* ) or assessment only or comparison samp* or propensity match*) ) | 1,332,800 |
| S24 | TI ( assign* N3 (subject* or patient* or client*) ) OR AB ( assign* N3 (subject* or patient* or client*) ) | 14,119 |
| S23 | TI ( (quasi-experiment* or quasiexperiment* or experiment*) ) OR AB ( (quasi-experiment* or quasiexperiment* or experiment*) ) | 1,703,640 |
| S22 | TI Intervention* N1 Stud* OR AB Intervention* N1 Stud* | 17,760 |
| S21 | TI retrospective OR AB retrospective | 157,002 |
| S20 | TI (prospective n2 study) OR AB (prospective n2 study) | 107,627 |
| S19 | TI observational OR AB observational | 76,736 |
| S18 | TI longitudinal OR AB longitudinal | 137,108 |
| S17 | TI ( ((follow up OR followup) N2 study) ) OR AB ( ((follow up OR followup) N2 study) ) | 21,850 |
| S16 | TI epidemiologic N2 study OR AB epidemiologic N2 study | 8,097 |
| S15 | TI ( (cross sectional) or (cross-sectional) or (point in time) or (point-in-time) ) OR AB ( (cross sectional) or (cross-sectional) or (point in time) or (point-in-time) ) | 210,437 |
| S14 | TI cohort OR AB cohort | 197,383 |
| S13 | TI case control OR AB case control | 68,388 |
| S12 | SU CROSS-sectional method | 46,712 |
| S11 | SU COHORT analysis | 26,343 |
| S10 | S8 AND S9 | 182,093 |
| S9 | Re | 4,446,322 |
| S8 | S1 OR S2 OR S3 OR S4 OR S5 OR S6 OR S7 | 2,407,962 |
| S7 | AB groups | 1,969,010 |
| S6 | AB trial | 416,856 |
| S5 | AB randomly | 141,990 |
| S4 | AB placebo | 71,520 |
| S3 | AB randomized | 175,162 |
| S2 | SU "RANDOMIZATION (Statistics)" | 802 |
| S1 | SU "randomized controlled trials" | 53,542 |

## ERIC - Limiters - Date Published: 20150101-20161231

**Search modes** - Boolean/Phrase

| **Search** | **Terms** | **Results** |
| --- | --- | --- |
| S42 | S38 AND S40 | (15) |
| S41 | S38 AND S40 | (389) |
| S40 | S8 OR S39 | (337,822) |
| S39 | S9 OR S10 OR S11 OR S12 OR S13 OR S14 OR S15 OR S16 OR S17 OR S18 OR S19 OR S20 OR S21 OR S23 OR S24 OR S25 | (323,722) |
| S38 | S35 AND S37 | (2,349) |
| S37 | S26 OR S27 OR S28 OR S29 OR S36 | (22,751) |
| S36 | DE "VETERANS" | (1,226) |
| S35 | S30 OR S31 OR S32 OR S33 OR S34 | (17,881) |
| S34 | TI (active N1 duty) OR AB (active N1 duty) | (171) |
| S33 | TI ((peace-keep* or peacekeep* or peace or keep* or military) N1 mission*) OR AB ((peace-keep* or peacekeep* or peace keep* or military) N1 mission*) | (23) |
| S32 | TI (combat* or deploy* or post-deploy* or postdeploy*) OR AB (combat* or deploy* or post-deploy* or postdeploy*) | (6,199) |
| S31 | TI (war or wars or warfare*) OR AB (war or wars or warfare*) | (10,939) |
| S30 | DE "War" | (3,219) |
| S29 | TI ((armed or naval or navy or air or defen#e* or national) N1 force* ) OR AB ( (armed or naval or navy or air or defen#e or national) N1 force1 ) | (413) |
| S28 | TI ( Army or troop* or military* or navy) OR AB ( Army or troop* or military* or navy) | (8,192) |
| S27 | TI ( (Soldier* or Marine* or airm#n* or Veteran* or militarian* or officer*) ) OR AB ( (Soldier* or Marine* or airm#n* or Veteran* or militarian* or officer*) ) | (15,318) |
| S26 | (DE "Military Personnel" OR DE "Air Force Personnel" OR DE "Army Personnel" OR DE "Commissioned Officers" OR DE "Enlisted Military Personnel" OR DE "Marine Personnel" OR DE "Navy Personnel" OR DE "Volunteer Military Personnel") | (2,719) |
| S25 | TI ((random* N2 trial*) or RCT) OR AB ( (random* N2 trial*) or RCT) | (2,842) |
| S24 | TI Non-random* or nonradom* or (non N1 random*) OR AB Non-random* or nonradom* or (non N1 random*) | (310) |
| S23 | TI (Propensity score* or heckman or difference in difference or regression discontinuity or (match* N1 control*) or (match* N1 compar* ) or assessment only or comparison samp* or propensity match*) OR AB (Propensity score* or heckman or difference in difference or regression discontinuity or (match* N1 control*) or (match* N1 compar* ) or assessment only or comparison samp* or propensity match*) | (130,784) |
| S22 | TI assign* N3 (subject* or patient* or client*) or AB assign* N3 (subject* or patient* or client* ) | (1,126) |
| S21 | Ti (quasi-experiment* or quasiexperiment* or experiment*) OR AB (quasi-experiment* or quasiexperiment* or experiment*) | (73,660) |
| S20 | TI Intervention* N1 Stud* OR AB Intervention* N1 Stud* | (3,419) |
| S19 | TI retrospective OR AB retrospective | (3,250) |
| S18 | TI (prospective n2 study) or AB (prospective n2 study) | (1,227) |
| S17 | TI observational OR AB observational | (4,288) |
| S16 | TI longitudinal or AB longitudinal | (23,650) |
| S15 | Ti ((follow up or followup or follow-up) N2 study )OR AB ((follow up or followup or follow-up) N2 study) | (4,266) |
| S14 | TI (epidemiologic N2 study) or AB (epidemiologic N2 study) | (143) |
| S13 | TI (cross sectional or cross-sectional or point in time or point-in-time) or AB ( cross sectional or cross-sectional or point in time or point-in-time) | (7,791) |
| S12 | TI cohort OR AB cohort | (10,148) |
| S11 | TI (case control) or AB (case control) | (672) |
| S10 | DE "CROSS-sectional method" OR DE "Comparative Analysis | (72,227) |
| S9 | DE "COHORT analysis" OR DE "Case Studies" | (53,291) |
| S8 | S6 AND S7 | (20,148) |
| S7 | Re | (172,767) |
| S6 | S1 OR S2 OR S3 OR S4 OR S5 | (229,670) |
| S5 | AB groups | (213,999) |
| S4 | AB trial | (11,032) |
| S3 | AB randomly | (13,985) |
| S2 | AB placebo | (709) |
| S1 | AB randomized | (4,113) |

## PsycINFO Limiters - Publication Year: 2015-2017

**Search modes** - Boolean/Phrase

| **Search Terms** | **Search Options** | **Results** |
| --- | --- | --- |
| S44 | S40 AND S42  **Limiters** - Publication Year: 2015-2017 | (687) |
| S43 | S40 AND S42 | (4,044) |
| S42 | S10 OR S41 | (1,192,647) |
| S41 | S11 OR S12 OR S13 OR S14 OR S15 OR S16 OR S17 OR S18 OR S19 OR S20 OR S21 OR S22 OR S23 OR S25 OR S26 OR S27 | (1,141,350) |
| S40 | S37 AND S39 | (15,982) |
| S39 | S28 OR S29 OR S30 OR S31 OR S38 | (63,253) |
| S38 | DE "VETERANS" OR DE "Military Veterans" | (11,726) |
| S37 | S32 OR S33 OR S34 OR S35 OR S36 | (54,725) |
| S36 | TI (active N1 duty) OR AB (active N1 duty) | (1,619) |
| S35 | TI ((peace-keep* or peacekeep* or peace keep* or military) N1 mission*) OR AB ((peace-keep* or peacekeep* or peace keep* or military) N1 mission*) | (201) |
| S34 | TI (combat* or deploy* or post-deploy* or postdeploy*) OR AB (combat* or deploy* or post-deploy* or postdeploy*) | (24,207) |
| S33 | TI (war or wars or warfare*) OR AB (war or wars or warfare*) | (31,305) |
| S32 | DE "War" | (12,251) |
| S31 | TI ((armed or naval or navy or air or defence or national) N1 force* ) OR AB ( (armed or naval or navy or air or defence or national) N1 force1 ) | (1,150) |
| S30 | TI ( Army or troop* or military* or navy ) OR AB ( Army or troop* or military* or navy) | (28,200) |
| S29 | TI ( (Soldier* or Marine* or airm#n or Veteran* or militarian* or officer*) ) OR AB ( (Soldier* or Marine* or airm#n or Veteran* or militarian* or officer*) ) | (39,661) |
| S28 | (DE "Military Personnel" OR DE "Air Force Personnel" OR DE "Army Personnel" OR DE "Commissioned Officers" OR DE "Enlisted Military Personnel" OR DE "Marine Personnel" OR DE "Navy Personnel" OR DE "Volunteer Military Personnel") | (16,264) |
| S27 | TI ((random* N2 trial*) or RCT) OR AB ( (random* N2 trial*) or RCT) | (40,028) |
| S26 | TI Non-random* or nonradom* or (non N1 random*) OR AB Non-random* or nonradom* or (non N1 random*) | (2,000) |
| S25 | TI (Propensity score* or heckman or difference in difference or regression discontinuity or (match* N1 control*) or (match* N1 compar* ) or assessment only or comparison samp* or propensity match*) OR AB (Propensity score* or heckman or difference in difference or regression discontinuity or (match* N1 control*) or (match* N1 compar* ) or assessment only or comparison samp* or propensity match*) | (576,997) |
| S24 | TI assign* N3 (subject* or patient* or client* ) or AB assign*S N3 (subject* or patient* or client*) | (268) |
| S23 | Ti (quasi-experiment* or quasiexperiment* or experiment*) OR AB (quasi-experiment* or quasiexperiment* or experiment*) | (371,070) |
| S22 | TI Intervention* N1 Stud* OR AB Intervention* N1 Stud* | (11,948) |
| S21 | TI retrospective OR AB retrospective | (28,497) |
| S20 | TI (prospective n2 study) or AB (prospective n2 study) | (25,284) |
| S19 | TI observational OR AB observational | (21,191) |
| S18 | TI longitudinal or AB longitudinal | (88,658) |
| S17 | Ti ((follow up or followup or follow-up) N2 study ) OR AB ((follow up or followup or follow-up) N2 study) | (14,235) |
| S16 | TI (epidemiologic N2 study) or AB (epidemiologic N2 study) | (4,548) |
| S15 | (TI cross sectional or cross-sectional or point in time or point-in-timeor) or (AB cross sectional or cross-sectional or point in time or point-in-time) | (103,184) |
| S14 | TI cohort OR AB cohort | (58,580) |
| S13 | TI (case control) or AB (case control) | (14,415) |
| S12 | DE "CROSS-sectional method" OR DE "Comparative Analysis" | (0) |
| S11 | DE "COHORT analysis" OR DE "Case Studies" | (1,212) |
| S10 | S8 AND S9 | (78,994) |
| S9 | Review | (509,893) |
| S8 | S1 OR S2 OR S3 OR S4 OR S5 OR S6 OR S7 | (896,698) |
| S7 | AB groups | (743,820) |
| S6 | AB trial | (141,584) |
| S5 | AB randomly | (61,504) |
| S4 | AB placebo | (35,382) |
| S3 | AB randomized | (52,921) |
| S2 | DE "trial*" | (368) |
| S1 | DE "RANDOM*" | (16,394) |

## SocIndex Limiters - Date of Publication: 20150101-20171231

| **Search** | **Terms** | **Results** |
| --- | --- | --- |
| S44 | S40 AND S42 **Limiters** - Date of Publication: 20150101-20171231 | (126) |
| S43 | S40 AND S42 | (1,459) |
| S42 | S10 OR S41 | (277,183) |
| S41 | S11 OR S12 OR S13 OR S14 OR S15 OR S16 OR S17 OR S18 OR S19 OR S20 OR S21 OR S22 OR S23 OR S25 OR S26 OR S27 | (249,965) |
| S40 | S37 AND S39 | (16,727) |
| S39 | S28 OR S29 OR S30 OR S31 OR S38 | (79,249) |
| S38 | DE "VETERANS" OR DE "Military Veterans" | (1,594) |
| S37 | S32 OR S33 OR S34 OR S35 OR S36 | (97,795) |
| S36 | TI (active N1 duty) OR AB (active N1 duty) | (449) |
| S35 | TI ((peace-keep* or peacekeep* or peace keep* or military) N1 mission*) OR AB ((peace-keep* or peacekeep* or peace keep* or military) N1 mission*) | (400) |
| S34 | TI (combat* or deploy* or post-deploy* or postdeploy*) OR AB (combat* or deploy* or post-deploy* or postdeploy*) | (17,534) |
| S33 | TI (war or wars or warfare*) OR AB (war or wars or warfare*) | (81,004) |
| S32 | DE "War" | (7,759) |
| S31 | TI ((armed or naval or navy or air or defence or national) N1 force* ) OR AB ( (armed or naval or navy or air or defence or national) N1 force1 ) | (714) |
| S30 | TI ( Army or troop* or military* or navy ) OR AB ( Army or troop* or military* or navy) | (36,421) |
| S29 | TI ( (Soldier* or Marine* or airm#n or Veteran* or militarian* or officer*) ) OR AB ( (Soldier* or Marine* or airm#n or Veteran* or militarian* or officer*) ) | (47,902) |
| S28 | (DE "Military Personnel" OR DE "Air Force Personnel" OR DE "Army Personnel" OR DE "Commissioned Officers" OR DE "Enlisted Military Personnel" OR DE "Marine Personnel" OR DE "Navy Personnel" OR DE "Volunteer Military Personnel") | (1,169) |
| S27 | TI ((random* N2 trial*) or RCT) OR AB ( (random* N2 trial*) or RCT) | (5,751) |
| S26 | TI Non-random* or nonradom* or (non N1 random*) OR AB Non-random* or nonradom* or (non N1 random*) | (403) |
| S25 | TI (Propensity score* or heckman or difference in difference or regression discontinuity or (match* N1 control*) or (match* N1 compar* ) or assessment only or comparison samp* or propensity match*) OR AB (Propensity score* or heckman or difference in difference or regression discontinuity or (match* N1 control*) or (match* N1 compar* ) or assessment only or comparison samp* or propensity match*) | (143,809) |
| S24 | TI assign* N3 (subject* or patient* or client* ) or AB assign*S N3 (subject* or patient* or client*) | (44) |
| S23 | Ti (quasi-experiment* or quasiexperiment* or experiment*) OR AB (quasi-experiment* or quasiexperiment* or experiment*) | (49,078) |
| S22 | TI Intervention* N1 Stud* OR AB Intervention* N1 Stud* | (1,857) |
| S21 | TI retrospective OR AB retrospective | (5,887) |
| S20 | TI (prospective n2 study) or AB (prospective n2 study) | (4,412) |
| S19 | TI observational OR AB observational | (3,564) |
| S18 | TI longitudinal or AB longitudinal | (26,766) |
| S17 | Ti ((follow up or followup or follow-up) N2 study ) OR AB ((follow up or followup or follow-up) N2 study) | (2,562) |
| S16 | TI (epidemiologic N2 study) or AB (epidemiologic N2 study) | (873) |
| S15 | (TI cross sectional or cross-sectional or point in time or point-in-timeor) or (AB cross sectional or cross-sectional or point in time or point-in-time) | (18,517) |
| S14 | TI cohort OR AB cohort | (16,276) |
| S13 | TI (case control) or AB (case control) | (2,843) |
| S12 | DE "CROSS-sectional method" OR DE "Comparative Analysis" | (4,180) |
| S11 | DE "COHORT analysis" OR DE "Case Studies" | (6,038) |
| S10 | S8 AND S9 | (34,981) |
| S9 | Review | (737,331) |
| S8 | S1 OR S2 OR S3 OR S4 OR S5 OR S6 OR S7 | (255,202) |
| S7 | AB groups | (224,106) |
| S6 | AB trial | (29,187) |
| S5 | AB randomly | (10,238) |
| S4 | AB placebo | (1,991) |
| S3 | AB randomized | (6,983) |
| S2 | DE "RANDOMIZATION (Statistics)" | (14) |
| S1 | DE "RANDOMIZED controlled trials" | (1,548) |

## Search Name: EMBASE deployment 2015-2017 Update

Date Run: 19/04/17 10:56:53.487

Description:

ID Search Hits

#1 Soldier* or Marine* or airm?n* or Veteran* or militarian* or officer*:ti,ab,kw or (Army or troop* or militar* or navy or ((armed or naval or air or defen?e or national) near/1 Force*)):ti,ab,kw or Military Personnel or veterans:kw 10936

#2 war:kw or war or wars or warfare* or (combat* or deploy* or post-deploy* or postdeploy*) or ((peace-keep* or peacekeep* or peace keep* or military) near/1 mission*) or (active near/1 duty) 3794

#3 S1 and S2 1687

#4 Randomized Controlled Trial or random allocation:kw or (randomized or placebo or randomly):ab or trials or groups:ab or (randomized controlled trial or controlled clinical trial):pt 1080419

#5 MeSH descriptor: [Case-Control Studies] explode all trees 14039

#6 MeSH descriptor: [Cohort Studies] explode all trees 133679

#7 #5 or #6 136931

#8 "comparative study":pt 155537

#9 MeSH descriptor: [Cross-Sectional Studies] explode all trees 4161

#10 MeSH descriptor: [Epidemiologic Studies] explode all trees 139841

#11 ("case control" or "cohort" or "cross sectional" or "cross-sectional" or "point in time" or "point-in-time") 56238

#12 ((epidemiolog* near/2 study) or ((follow up or followup or follow-up) near/2 study)) 12615

#13 (longitudinal or observational or (prospective near/2 study) or retrospective) 105813

#14 MeSH descriptor: [Clinical Trial] explode all trees 399

#15 quasi-experiment* or quasiexperiment* or experiment* 88430

#16 (assign* near/5 (subject* or patient* or client*)) 109471

#17 (Propensity score* or heckman or difference in difference or regression discontinuity (compar* near/1 group*) or (match* near/1 control*) or (match* near/1 compar*) or assessment only or comparison samp* or propensity-matched):ti,ab,kw 182371

#18 (Non-random* or nonradom* or (non adj1 random*)) or rct 251179

#19 S4 or S7 or S8 or S9 or S10 or S11 or S12 or OR13 or S14 or S15 or S16 or S17 4587

#20 S3 and S19 879

Limited to 01/01-2015 - 01/04-2017 – 193 references.

| # 26 | [543](http://apps.webofknowledge.com/summary.do?product=WOS&doc=1&qid=44&SID=V1TXuBwpeNP27yczH5o&search_mode=CombineSearches&update_back2search_link_param=yes) | #3 AND #1  *Indexes=SCI-EXPANDED, SSCI Timespan=2015-2017* |
| --- | --- | --- |
| # 25 | [279](http://apps.webofknowledge.com/summary.do?product=WOS&doc=1&qid=43&SID=V1TXuBwpeNP27yczH5o&search_mode=CombineSearches&update_back2search_link_param=yes) | #24 AND #7  *Indexes=SCI-EXPANDED, SSCI Timespan=2015-2017* |
| # 24 | [1,726,384](http://apps.webofknowledge.com/summary.do?product=WOS&doc=1&qid=42&SID=V1TXuBwpeNP27yczH5o&search_mode=CombineSearches&update_back2search_link_param=yes) | #23 OR #22 OR #21 OR #20 OR #19 OR #18 OR #17 OR #16 OR #15 OR #14 OR #13 OR #12 OR #11 OR #10 OR #9 OR #8  *Indexes=SCI-EXPANDED, SSCI Timespan=2015-2017* |
| # 23 | [97,766](http://apps.webofknowledge.com/summary.do?product=WOS&doc=1&qid=41&SID=V1TXuBwpeNP27yczH5o&search_mode=AdvancedSearch&update_back2search_link_param=yes) | TS=((random* NEAR/5 trial*) OR RCT)  *Indexes=SCI-EXPANDED, SSCI Timespan=2015-2017* |
| # 22 | [30,671](http://apps.webofknowledge.com/summary.do?product=WOS&doc=1&qid=40&SID=V1TXuBwpeNP27yczH5o&search_mode=AdvancedSearch&update_back2search_link_param=yes) | TS=(Non-random* or nonradom* or (non random*))  *Indexes=SCI-EXPANDED, SSCI Timespan=2015-2017* |
| # 21 | [427,207](http://apps.webofknowledge.com/summary.do?product=WOS&doc=1&qid=38&SID=V1TXuBwpeNP27yczH5o&search_mode=AdvancedSearch&update_back2search_link_param=yes) | TS=(((Propensity score* or heckman or difference in difference or regression discontinuity or (match* near control*) or (match* near compar* ) or assessment only or comparison samp* or propensity matched)))  *Indexes=SCI-EXPANDED, SSCI Timespan=2015-2017* |
| # 20 | [5,538](http://apps.webofknowledge.com/summary.do?product=WOS&doc=1&qid=36&SID=V1TXuBwpeNP27yczH5o&search_mode=AdvancedSearch&update_back2search_link_param=yes) | TS=(assign* NEAR/5 (subject* OR patient* OR client*))  *Indexes=SCI-EXPANDED, SSCI Timespan=2015-2017* |
| # 19 | [491,431](http://apps.webofknowledge.com/summary.do?product=WOS&doc=1&qid=34&SID=V1TXuBwpeNP27yczH5o&search_mode=AdvancedSearch&update_back2search_link_param=yes) | TS=((quasi-experiment* OR quasiexperiment* OR experiment*))  *Indexes=SCI-EXPANDED, SSCI Timespan=2015-2017* |
| # 18 | [14,858](http://apps.webofknowledge.com/summary.do?product=WOS&doc=1&qid=33&SID=V1TXuBwpeNP27yczH5o&search_mode=AdvancedSearch&update_back2search_link_param=yes) | TS=(Intervention* NEAR/5 stud*)  *Indexes=SCI-EXPANDED, SSCI Timespan=2015-2017* |
| # 17 | [70,245](http://apps.webofknowledge.com/summary.do?product=WOS&doc=1&qid=31&SID=V1TXuBwpeNP27yczH5o&search_mode=AdvancedSearch&update_back2search_link_param=yes) | TS=(retrospective)  *Indexes=SCI-EXPANDED, SSCI Timespan=2015-2017* |
| # 16 | [40,813](http://apps.webofknowledge.com/summary.do?product=WOS&doc=1&qid=30&SID=V1TXuBwpeNP27yczH5o&search_mode=AdvancedSearch&update_back2search_link_param=yes) | TS=((prospective near/2 study))  *Indexes=SCI-EXPANDED, SSCI Timespan=2015-2017* |
| # 15 | [34,387](http://apps.webofknowledge.com/summary.do?product=WOS&doc=1&qid=29&SID=V1TXuBwpeNP27yczH5o&search_mode=AdvancedSearch&update_back2search_link_param=yes) | TS=(observational)  *Indexes=SCI-EXPANDED, SSCI Timespan=2015-2017* |
| # 14 | [49,554](http://apps.webofknowledge.com/summary.do?product=WOS&doc=1&qid=28&SID=V1TXuBwpeNP27yczH5o&search_mode=AdvancedSearch&update_back2search_link_param=yes) | TS=(longitudinal)  *Indexes=SCI-EXPANDED, SSCI Timespan=2015-2017* |
| # 13 | [6,088](http://apps.webofknowledge.com/summary.do?product=WOS&doc=1&qid=26&SID=V1TXuBwpeNP27yczH5o&search_mode=AdvancedSearch&update_back2search_link_param=yes) | TS=(epidemiological near/2 study)  *Indexes=SCI-EXPANDED, SSCI Timespan=2015-2017* |
| # 12 | [114,849](http://apps.webofknowledge.com/summary.do?product=WOS&doc=1&qid=25&SID=V1TXuBwpeNP27yczH5o&search_mode=AdvancedSearch&update_back2search_link_param=yes) | TS=(cross sectional or cross-sectional or point in time or point-in-time)  *Indexes=SCI-EXPANDED, SSCI Timespan=2015-2017* |
| # 11 | [40,782](http://apps.webofknowledge.com/summary.do?product=WOS&doc=1&qid=24&SID=V1TXuBwpeNP27yczH5o&search_mode=AdvancedSearch&update_back2search_link_param=yes) | TS=(Comparative study)  *Indexes=SCI-EXPANDED, SSCI Timespan=2015-2017* |
| # 10 | [176,224](http://apps.webofknowledge.com/summary.do?product=WOS&doc=1&qid=23&SID=V1TXuBwpeNP27yczH5o&search_mode=AdvancedSearch&update_back2search_link_param=yes) | TS=((case control) or cohort)  *Indexes=SCI-EXPANDED, SSCI Timespan=2015-2017* |
| # 9 | [277,886](http://apps.webofknowledge.com/summary.do?product=WOS&doc=1&qid=20&SID=V1TXuBwpeNP27yczH5o&search_mode=AdvancedSearch&update_back2search_link_param=yes) | TS=(Review)  *Indexes=SCI-EXPANDED, SSCI Timespan=2015-2017* |
| # 8 | [687,738](http://apps.webofknowledge.com/summary.do?product=WOS&doc=1&qid=19&SID=V1TXuBwpeNP27yczH5o&search_mode=AdvancedSearch&update_back2search_link_param=yes) | TS=(Randomized or Placebo or Randomly or Trial or Group)  *Indexes=SCI-EXPANDED, SSCI Timespan=2015-2017* |
| # 7 | [556](http://apps.webofknowledge.com/summary.do?product=WOS&doc=1&qid=18&SID=V1TXuBwpeNP27yczH5o&search_mode=CombineSearches&update_back2search_link_param=yes) | #6 OR #5 OR #4  *Indexes=SCI-EXPANDED, SSCI Timespan=2015-2017* |
| # 6 | [543](http://apps.webofknowledge.com/summary.do?product=WOS&doc=1&qid=17&SID=V1TXuBwpeNP27yczH5o&search_mode=CombineSearches&update_back2search_link_param=yes) | #3 AND #1  *Indexes=SCI-EXPANDED, SSCI Timespan=2015-2017* |
| # 5 | [4](http://apps.webofknowledge.com/summary.do?product=WOS&doc=1&qid=16&SID=V1TXuBwpeNP27yczH5o&search_mode=CombineSearches&update_back2search_link_param=yes) | #4 AND #1  *Indexes=SCI-EXPANDED, SSCI Timespan=2015-2017* |
| # 4 | [17](http://apps.webofknowledge.com/summary.do?product=WOS&doc=1&qid=15&SID=V1TXuBwpeNP27yczH5o&search_mode=CombineSearches&update_back2search_link_param=yes) | #3 AND #2  *Indexes=SCI-EXPANDED, SSCI Timespan=2015-2017* |
| # 3 | [41,606](http://apps.webofknowledge.com/summary.do?product=WOS&doc=1&qid=14&SID=V1TXuBwpeNP27yczH5o&search_mode=AdvancedSearch&update_back2search_link_param=yes) | TS=((war OR wars OR warfare OR combat* OR deploy* OR post-deploy* OR postdeploy*) OR (active NEAR/1 duty))  *Indexes=SCI-EXPANDED, SSCI Timespan=2015-2017* |
| # 2 | [28](http://apps.webofknowledge.com/summary.do?product=WOS&doc=1&qid=10&SID=V1TXuBwpeNP27yczH5o&search_mode=AdvancedSearch&update_back2search_link_param=yes) | TS=((Peace-keep* OR Peacekeep* OR (Peace NEAR/1 keep*)) NEAR/1 mission*)  *Indexes=SCI-EXPANDED, SSCI Timespan=2015-2017* |
| # 1 | [1,727](http://apps.webofknowledge.com/summary.do?product=WOS&doc=1&qid=5&SID=V1TXuBwpeNP27yczH5o&search_mode=AdvancedSearch&update_back2search_link_param=yes) | TS=((Soldier* OR Marine* OR Airm?n OR Veteran or Militarian* OR Officer* OR Army OR Troop OR Military* OR Navy OR (Armed OR Naval OR Navy OR Air OR Defence)) NEAR/1 Force*)  *Indexes=SCI-EXPANDED, SSCI Timespan=2015-2017* |
